# Body dysmorphic disorder of female genitalia: a qualitative study of Swiss obstetrician–gynecologists’ experiences and practices

**DOI:** 10.1007/s00404-021-06270-w

**Published:** 2021-09-30

**Authors:** Olenka Dworakowski, Marie Drüge, Michelle Schlunegger, Birgit Watzke

**Affiliations:** Department of Psychology, University of Zurich, Zurich, Switzerland

**Keywords:** Body dysmorphic disorder, Mental health, Female genitalia, Gynecology, Qualitative research

## Abstract

**Purpose:**

This work focuses on the experiences and practices of obstetrician–gynecologists (ob–gyns) with patients suffering from body dysmorphic disorder (BDD) and issues with their aesthetics, specifically focusing on female genitalia. Ob–gyns are likely to play an important role in the recognition and treatment of women facing such issues.

**Methods:**

This study took a qualitative, explorative approach. Semi-structured interviews were conducted with 11 ob–gyns about their experiences with patients who presented symptoms of BDD of female genitalia, their treatments, and interest in further education and supportive material. Interviews were analyzed through qualitative content analysis.

**Results:**

A categorization system was created. The results showed that the participating ob–gyns are often confronted with genital dissatisfaction of patients. The study sample demonstrated a lack of mental health literacy concerning BDD. The treatments that the ob–gyns of this sample suggested for BDD of female genitalia were not in line with what evidence suggests. Finally, interest in further education and supportive material for consultation was evidenced in this sample.

**Conclusions:**

The findings encourage further studies to identify the recognition of BDD concerning genitalia or etiological factors. Furthermore, practical implications (e.g., need of supportive material) can be derived from the results.

**Supplementary Information:**

The online version contains supplementary material available at 10.1007/s00404-021-06270-w.

## Introduction

Body dysmorphic disorder is a psychological disorder defined by distressing preoccupation with a perceived defect in one’s appearance [1]. The criteria in the Diagnostic and Statistical Manual of Mental Disorders (DSM) V are: preoccupation with appearance, distress about appearance, repetitive behaviors, or mental acts (e.g., comparing or asking for reassurance) in response to concerns, and concerns cannot be explained by any other mental disorder [2]. BDD shows a prevalence of nearly 2% [3]. Associated features include strong feelings of shame [4], excessive safety behaviors [5], mirror checking, heavy make-up, or aesthetic surgery [6], sexual problems including decreased libido [4], and poor insight [7]. In some cases, BDD can also lead to suicidal ideation [1]. Sociocultural influences, such as the media, and bullying—for example comments on one’s body by peers or family members—have been associated with the development of BDD [8]. Studies show effective treatment of BDD with cognitive behavioral therapy (CBT) [9]. Furthermore, surgical treatment is contraindicated [10], even though beneficial effects of aesthetic surgery have been found for patients without mental disorders [11]. BDD can concern any specific body part. Here, we will focus on BDD concerning female genitalia.

The term ‘female genitalia’ is defined here as female external genitalia. Even though this term will be used, the authors of this work recognize that ‘female genitalia’ is not an organ only cisgender women have. Literature on BDD concerning female genitalia is rather scarce [5; 6], research on dissatisfaction with genitalia that might be subclinical is related to BDD [1] and can give insight to this topic.

General dissatisfaction with genitalia has been found to be related to sexual function or sexual esteem [12], general self-esteem, and body satisfaction [13]. The literature on genital dissatisfaction implies that many of women experience negative feelings toward their own genitalia [14], which is mirrored in the rising number of labiaplasty [15]. Explanations for these large numbers have not been researched extensively, but initial research shows that many women seeking labiaplasty suffer from BDD of female genitalia [6].

Developmental factors of BDD specific to female genitalia might be a little different than of other body parts. Sociocultural factors such as bullying are likely to only occur from close family members, medical staff, or sexual partners, since not many other people see one’s genitalia. In the case of media, pornography is most likely to influence genital satisfaction [16]. Pornographic media do not depict natural variance of female genitalia but show only one ideal, giving women unrealistic standards [17]. Women are likely to be uninformed about natural variances of female genitalia, also because not many women speak about their genitalia openly to friends and peers [16].

The vulva is still a societal taboo [16], one of the only people with whom women can openly discuss worries or questions concerning the appearance of their genitalia might be obstetrician–gynecologists (ob–gyns). Recently, the importance of psychosocial aspects of obstetrics and gynecology has been prominently discussed [18–20]. Unfortunately, studies show that ob–gyns sometimes do not recognize psychological disorders as such [21–23]. The Swiss association of gynecologists does warn that women seeking labiaplasty surgery might be suffering from some kind of psychological distress; however, they do not specifically mention BDD [24]. It seems of vital importance that ob–gyns are well informed about BDD, so that they can recommend correct treatment [25].

This work focuses on the viewpoint of ob–gyns for two main reasons. First, as mentioned, ob–gyns may play an important role in recognition and treatment of patients suffering from BDD of female genitalia [22]. Second, they might also have valuable insights into patients’ experiences. Patients are hard to reach, because BDD often stays unrecognized [3] and there are no current statistics on patients of BDD nor labiaplasty in Switzerland. The aims of this work are to investigate (1) the knowledge ob–gyns show concerning BDD, (2) their experiences with it, (3) treatments they offer these women, and (4) their interest in further education on BDD. We chose a qualitative approach because of lacking specific previous research.

## Methods

### Participants and recruitment

Participants were recruited via telephone or email. Inclusion criteria was an active practice of gynecology and exclusion criteria was non-fluency in German. Thirty-six ob–gyns were personally contacted and asked to participate in the study. These were chosen through personal contacts and internet research. To ensure as much variation as possible in the job description, the following aspects were taken into account in the selection process: age, gender, work experience, work location, practice vs. clinic, additional training, and surgery performance. Two shared practices with a total of seven ob–gyns were reached out to as well. Thirty four of these total 43 contacted ob–gyns were not interested in participation, most of them because they did not have any time. Nine of them agreed to participate in the study. This renders in a participation rate of 20.93%. Additionally, two large hospitals were contacted of which another two further ob–gyns agreed to participate. Finally, 11 ob–gyns participated. Two participants knew the first author (OD) before the study, but there was no close relationship that would influence the results of the study. With all other participants, the only contact before the interview was via email or telephone to set a date. The participants were told that the interviews were about women and their attitude toward their own body, and the term ‘BDD’ was purposefully not mentioned prior to the interview. The mean age of participants was *M* = 46.30 (SD = 9.76). The mean years of practicing work were *M* = 21.36 (SD = 11.13). Five of them worked in shared practices, three of them in their own practice and four in a large clinic. A table containing all demographic information of participants can be found in table S1 supplementary material.

### Interviews

Semi-structured expert interviews were conducted. The two first authors and the third author of this paper constructed the questions. The questions were tested in a pilot interview with a medical doctor who used to practice gynecology. Interview questions were categorized into four topics: general confrontation with psychological disorders and BDD; symptoms of BDD concerning female genitalia; treatments; and interest in further education and materials. The first two sections each included an example case which were derived from cases found in the literature [26–29] and validated by seven psychotherapists, in that they all correctly diagnosed the cases with BDD with no prior knowledge about the aims of this study. Interview questions are displayed in table S2 in the supplementary material. Full transcripts can be found in the supplementary material. The first (OD) and third (MS) authors, who were Masters students of psychology at the time, conducted all interviews together. Both these researchers are female and it was their first experience in scientific interviews. They were coached and supervised by the first author (MD) who is a postdoctoral researcher with prior experience in qualitative research. The data were collected in the offices of the participants, except for one interview that was held at a café. Only the researchers and the participants were present at the interview. After the interview, participants were informed about the background and purpose of the study and received a declaration of intentions as well as a sheet on information about BDD. Participants knew that the study was conducted by the University of Zurich and signed an informed consent. All interviews were recorded and transcribed by the first (OD) and third author (MS). Transcripts were not returned to participants for comments and no additional field notes were held. The interviews had a mean length of *M* = 28.27 (SD = 7.8, range = 17–41) min. The interviews were conducted in May and June 2019.

### Data analysis

The interview transcripts were analyzed using summarizing content analysis [30]. The categorization system for this work was first proposed deductively, leaning on prior research and literature (e.g., [6, 10, 21]). For each question of the interview, possible answers were thought of and proposed as category codes. After analyzing all the transcripts for the first time, many new categories appeared. Therefore, the system was expanded inductively. After the first analysis, the categories were reviewed, and some were deleted or incorporated into a different category. Transcripts were each analyzed by two coders using RQDA software. Differences in coding were discussed and an agreement was met. Findings were sent to participants, but none responded with specific feedback.

## Results

The complete categorization system can be found in the supplementary material. Table 1 gives a quick overview of the main results.

**Table 1:** Summary of results

Topic	Main results
Confrontation with BDD	Most participants recognized symptoms
	None of the participants correctly diagnosed BDD
Experiences with BDD of fem. genitalia	All participants had some experience
	Influencing factors: media, comments, uninformed, unrealistic ideals
	Symptoms: distorted perception, shame, lacking insight, comparing
	Labiaplasty: different opinions on pros and cons
	Further topics: medical issues, psych. issues disguised as somatic issues, subclinical dissatisfaction, ob–gyn as person of trust
Treatments	Transfer to psychotherapy
	Transfer to surgical treatment
	Advise against surgery
	Educate
	Lacking education in psychological issues
	Stronger interest of physicians in settled practices
Further Education	All at least medium interest or more
Diagnostic Material	Participants working in clinics tended to stronger interest
	Practical, helpful to standardize and raise awareness
	Impractical, unfitting, unnecessary
Supportive Material	Most medium-to-strong interest
	Visual material
	Already in use
	Information to hand out

### Confrontation with BDD

All participants mentioned some type of psychological disorders or issues with which they were confronted in their work. When reading the first example case, all but one recognized issues of self-worth, and five participants mentioned her having body perception issues. None of the participants correctly diagnosed BDD, five claimed not to have heard of this diagnosis.

*Participant 6:*

*“But I think this does catch your attention, this body image, this [low] self-esteem in this area. I don’t know the technical term for this now. (laughs)”*

*Participant 7:*

*“Yes, she avoids social activities, does not go out. She suffers from her flaw, much too much. She withdraws, that is the beginning of the end, before the destruction comes. If she can’t change this, she will get sick. […] I am not a psychologist. I don’t know how you say that psychologically.”*

### Experiences with symptoms of BDD concerning female genitalia

Statements about the participants’ experiences with symptoms of BDD of female genitalia were categorized into: influencing factors, symptoms, labiaplasty, and further topics. Each category had further subcategories.

Five participants mentioned that issues with the aesthetics of one’s genitalia have been increasing.

Some assumed influencing factors were: media, comments, being uninformed, and unrealistic ideals.

*Participant 2:*

*“The norm [of appearance of genitalia] is not only like what they see maybe in a magazine or so.”*

*Participant 3:*

*“And often this question [about aesthetics of genitalia] is only triggered by some encounter or by some comment.”*

*Participant 6:*

*“Well, I think that here, the media world really does have a big part in it.”*

*Participant 11:*

*“Aesthetic ideas [of genitalia] that are given, that quasi are seen as normal, it really is not normal.”*

Specific symptoms of BDD were mentioned: distorted perception, shame, lacking insight, and comparing oneself.

*Participant 2:*

*“Very often I would now say, is that there really [is] a discrepancy to their subjective perception. (…) And that what I think objectively.”*

*Participant 10:*

*“Because, when that is something the person is suffering from, she is ashamed in the changing room, she is ashamed with her boyfriend (…)”*

*Participant 11:*

*“Or they compare themselves, that is often when that comes up.”*

Participants expressed different opinions on labiaplasty. Some argued pro surgery, and others were more opposed. Some mentioned high risks, and others said that it is a simple procedure.

*Participant 2:*

*“Surgery normally does not make it [dissatisfaction with genital area] better.”*

*Participant 7:*

*“When it clearly is something, that just has to be taken off, then I directly sign them up for surgery.”*

*Participant 10:*

*“(…) sometimes then it helps an actually small surgery, so that she just feels more self-secure”.*

*Participant 11:*

*“I say: ‘when you have complications you can come to me’. That also exists right.”*

Some further topics arose during the interviews. Participants spoke about medical issues in the genital area.

*Participant 3:*

*“And then there also really exist the very obvious malformations (…).”*

On the other hand, participants also talked about how some women might find it easier to talk about somatic problems instead of their appearance issues. Participants suspected that women come up with medical issues to justify a labiaplasty, for example. Or they suspected a larger issue to lie behind the wish for surgery.

*Participant 11:*

*“(…) or that one realizes that maybe more lies behind it [the medical problem], right.”*

The topics of sexuality and relationships and their association with genital dissatisfaction or ideals also often came up.

*Participant 9:*

*“[quoting a patient:] ‘he won’t want to sleep with me anymore, if I am not shaven’”*

Participants also spoke of many situations where the patients were very likely to not be suffering from BDD but merely subclinical dissatisfaction.

*Participant 7:*

*“Actually good [experiences]. When they really are normal, they really accept it, the most of them. I have never had anyone yet, who always came back to it and came back to it again.”*

*Participant 11:*

*“Well that [dissatisfaction with genitalia] can’t only be seen as a disorder.”*

Finally, participants also talked about being a person of trust to their patients and how many of them talk about personal issues.

*Participant 7:*

*“Because these [psychological problems that patients want to talk about] come from the first*

*day on, I can guarantee you that.”*

*Participant 11:*

*“Well I would say, as gynecologists one is a doctor of trust”*

### Treatments

Many different answers came up when asking participants about treatments. Only one participant spoke of psychotherapy specifically, few others mentioned group therapy or sex therapy.

*Participant 1:*

*“Then, I would maybe also again, like before, explain that it [the genitalia] is normal. (…) Now, when it is only psychological suffering then, as I said before, it is in any case important that first a therapy takes place. Psychotherapy, or yes”*

Further treatments were transfer patients to surgery, or advise against surgery. Many participants also spoke about educating their patients on the normal variety of female genitalia.

*Participant 4:*

*“Where does she really need support, and I think the surgery of the genitalia is a secondary issue here, and I tried to advise the woman against it.”*

*Participant 7:*

*“‘No you are normal’ [I tell the patient] and finished. ‘Please accept that’. Here I also don’t offer any psychological consultation.”*

*Participant 8:*

*“(…) if a woman really insists on it then it [the labiaplasty] will be done (…)”*

*Participant 11:*

*“And say yes but that it [the labia] has a function. Then I explain here that that is a protection.”*

### Interest in further education, diagnostic material, and supportive material

The interests of the participants were categorized by strength. Additional topics came up in each area.

Participants all exhibited at least medium interest in further education. Additional topics were: lacking education in psychological issues and stronger interest of physicians in settled practices.

*Participant 8:*

*“Often because there is also an undersupply in the further education, right.”*

Most of those who expressed strong interest in diagnostic material worked in a clinic. Diagnostic material was said to be practical and helpful to standardize a diagnosis and raise awareness.

*Participant 8:*

*“That is surely ah, that is surely very interesting, because it also helps, or standardizes, right.”*

On the other hand, some said that it is impractical, unfitting, or even unnecessary.

*Participant 5:*

*“I don’t think, (…), that I need that because one notices yes, that, one sees the congruence.”*

All but one participant showed medium-to-strong interest in supportive material. Further comments were made that it depended on the exact format of the supportive material, that information to hand out would be practical, or that they already used such materials. The majority of the participants showed strong interest in supportive visual materials. Some participants had already used such materials.

*Participant 6:*

*“Well, I think that it often helps in a conversation when one also has some kind of guidelines to hand out to the patient.”*

*Participant 8:*

*“We have a very interesting, (…) a plaster poster. There plaster casts of, ahm, female genitalia were made.”*

## Discussion

The qualitative results of this study are not to be interpreted as representative, but reflect individual experiences of ob–gyns in different settings and from different backgrounds. The aim of this qualitative study was not to collect representative data, but to explore where future research should be directed at. Still, many issues come up that can be connected with the previous literature. The data are local to Swiss ob–gyns only, but since previous data on issues of BDD and labiaplasty are largely missing the data will be discussed with reference to global data. Because many topics that can be related to previous findings as well as additional themes and different viewpoints of participants from different backgrounds came up during the interviews the data saturation was deemed as sufficient.

### Confrontation with BDD

All participants spoke of psychological issues being present in the gynecological setting. This result is in line with the previous studies [21]. Most participants correctly recognized symptoms of BDD in the example case, but none correctly diagnosed the patient as suffering from BDD. Furthermore, only half of the participants claimed to have heard of BDD at all. This is further in line with the previous literature, showing that ob–gyns do not always recognize psychological issues as such [21, 23]. For BDD, this effect might be stronger as it is somewhat of a lesser known diagnosis. These results might lead to the hypothesis that ob–gyns in Switzerland generally exhibit low mental health literacy concerning BDD that could be further investigated.

### Experiences with symptoms of BDD concerning female genitalia

All participants had at least some experience with women facing issues with the aesthetic of their genitalia, and this shows that it is a topic with some relevance in ob–gyns practices. Moreover, participants stated that these issues have been increasing. This corresponds to recent statistics concerning labiaplasty [15].

#### Influencing factors

The ob–gyns participating in this study suggested several factors that might influence women’s satisfaction with their genitalia. Several etiological factors were mentioned, which have previously been connected to BDD. Participants in the current study also suspected that misinformation and media exposure are connected to dissatisfaction with one’s genitalia. This is in line with the previous findings that media can influence the development of BDD [8, 12, 16]. Pornographic media only display a very narrow variance of vulvas [14, 17], and since women mostly do not speak about their issues or questions openly, this might further keep women from being well informed [16].

Participants mentioned that many women might experience such insecurities due to comments by sexual partners or parents. This can be a type of bullying experience, which studies have also found to influence the development of BDD and the decision to undergo labiaplasty [6].

#### Symptoms

Participants mentioned specific symptoms that women who have issues with the aesthetics of their genitalia might present. These included feelings of shame, distorted perceptions, poor insight, and comparing yourself. These are all symptoms described in the literature on BDD [1, 4]. This result further underlines the assumption that at least some patients of these ob–gyns are suffering from BDD or symptoms that might develop into BDD.

Interview results point toward the wish for aesthetic surgery being a common symptom of BDD at ob–gyns’ offices, and a tangible one. Many participants expressed skepticism in sending their patients to plastic surgeons without a medical indication. Then again, most participants also mentioned situations in which they thought it made sense. Since these ob–gyns did not recognize BDD, it does not seem as if they would consider BDD as a contraindication for surgery. More likely, it seems that their professional opinion of what is medically relevant would influence this decision. Researchers have found that how physicians rate the attractiveness of a vulva influences their decision to perform surgery on a patient [25]. What is medically in a range of normality does not always correspond with the feelings someone might harbor toward their body.

#### Further topics

Another topic that came up in the interviews was sexuality. Patients suffering from BDD commonly have decreased libido and a hard time maintaining intimate relationships [1, 4]. Sexual issues might be another more tangible topic through which BDD might present itself at the gynecologist’s office and therefore could be a reason to ask targeted screening questions.

Patients who show insecurities about the appearance of their genitalia might suffer only subclinical issues. It is impossible to know how many patients of the participating ob–gyns truly do suffer from BDD. Comments of participants indicated that some of their patients might not be suffering from full-blown BDD. Nevertheless, all issues must be taken seriously. Subclinical dissatisfaction is likely to develop into BDD [1]. Furthermore, genital dissatisfaction can also have an effect on mental health as it might influence sexual function [12] or self-esteem [13].

### Treatments

Many different categories treatments of women who present issues with the aesthetics of their genitalia were mentioned. Studies have shown good results treating patients suffering from BDD with CBT [9]. Only one participant directly articulated referring such patients to a psychotherapist. Others spoke of sex therapy or group therapy, both of which are not what the literature suggests as the best treatment for BDD.

Most participants claimed that they would advise against surgery. This is in line with what is known about surgical treatments of BDD [10]. However, these ob–gyns did not advise against surgery, because they suspected BDD, but generally when they did not think surgery was necessary.

Most participants also described situations where they thought it appropriate to send their patients to surgery. For healthy patients with slight appearance issues, aesthetic surgery can be beneficial for their wellbeing [11]. However, careful diagnostics are necessary to determine which treatment option is best suited for patients. The Swiss gynecologists association mentions psychological issues, but does not give specific guidelines of how to diagnose patients specifically [24]. Also, in the guidelines for plastic surgeons in Switzerland, there is no article about screening for mental health [31]. Therefore, once patients are transferred to surgery, it is likely they are not diagnosed further. It does not seem as though the participants of this study were well informed about the best treatment options available for patients with such issues or how to best diagnose women with BDD.

Participants spoke of educating patients on surgery or generally on the normality of their genitalia. Some spoke about emphasizing the natural variety a vulva can have. Since many women seem to be misinformed about female genitalia [14] and this is also suspected to influence their dissatisfaction, such education of women presenting insecurities about the appearance of their genitalia seems very appropriate and might prevent further development of symptoms. However, in cases where BDD is already in a more advanced state, these treatments are not likely to be sufficient. Once again, it seems important that ob–gyns are able to differentiate between patients suffering from BDD and those who are still at a subclinical stage of dissatisfaction.

### Interest in further education, diagnostics, and supportive material

The results of the interviews suggest that ob–gyns have a large interest in further education on BDD. Participants also mentioned that such education would be very helpful, since ob–gyns are rather undereducated in mental health issues. This is in line with what has been found in the previous literature [20] and is consistent with what has been described in this paper.

Results of interest in diagnostic material were more diverse. Although the results of this study show that ob–gyns are unlikely to correctly recognize BDD as such, some participants were of the opinion that diagnostic support would be impractical or unnecessary in their daily practice. Others said that it was unsuitable, because people in reality are too variable. Though this might harbor some truth, it seems as though screening material would be very helpful. For example, when asked about recommendations for labiaplasty, ob–gyns could use the COPS-L [32], a screening instrument for BDD of the genital area specifically designed to identify such patients. A short screening test could already point the patients to more specific treatment options. A recent review also gave recommendations on how to better recognize patients suffering from BDD when they are asking about labiaplasty [22].

### Limitations and strengths

The interview questions had to be composed with little literature to base them on. Two questions were added after the first interview. At the same time, the greatest strength of this research is that it is pioneering work in a still under investigated field.

Another limitation is the small sample size. This qualitative study did not aim at a representative sample; no conclusions can be drawn for a larger population. All participants are Swiss, so the results must be interpreted in this context. Further research could investigate the same issues in other countries as basic gynecological care varies. Participants were informed that the interviews would be about women and body issues and that the study was conducted by psychologists, so a self-selection of participants that show a basic interest in topics of mental health possibly occurred. Still, through the qualitative nature of the study, many important insights can be drawn from this sample which point the way for further research. Based on the lack of knowledge this sample presents, it is likely that a sample that has less interest in psychological topics might present even less knowledge. Of course, this must be investigated further.

One of the primary findings of this research is that ob–gyns show a lack of sufficient knowledge of BDD. This also presents a limitation of the sample. It is impossible to know which patients of ob–gyns truly are suffering from BDD and which face subclinical issues.

### Future research and practical implications

There is still a lack of basic research in this field. Future studies could replicate our interviews in other countries or contexts (e.g., after giving birth). Large-scale studies should explore how women generally feel about their own genitalia, whether these issues are increasing and what influences these feelings. Furthermore, one could explore how subclinical dissatisfaction is connected to BDD and how the development of BDD could be prevented. General etiological factors of BDD concerning genitalia should be explored, and these factors might differ from other body parts as they are rarely exposed to the gaze of others.

Since BDD might stay unrecognized by the people suffering themselves, it seems important to find more evidence to how it might present itself. This would be additional work toward improving recognition and proper treatment of people suffering from BDD. Future research should also be conducted in fields beyond obstetrics and gynecology, such as surgical professions.

Subsequent studies could also further research how patients presenting BDD of female genitalia are treated in a larger scope or the efficacy of these treatments, as well as in cases of subclinical satisfaction.

Further education for ob–gyns should be implemented.

## Conclusions

This pioneering study revealed many interesting aspects of how the participating gynecologists are confronted with mental health issues, specifically BDD concerning genitalia. The conclusions drawn here only represent this sample. Nonetheless, interesting hypotheses for future, more large-scale research can be derived.

Even though participants did display understanding of issues with aesthetic of genitalia and where these might come from, a lack of mental health literacy concerning BDD was identified in this sample. This has consequences for ensuring the proper treatment of women suffering from these issues. Concerns of genital appearance seem to arise with some regularity at the gynecologist’s office, although the data from this study are not able to shed any light on how many of these patients might be diagnosed with BDD or how many have subclinical concerns. It is important to point out that subclinical concerns are of large importance as well, since treating these concerns can work preventively against the development BDD. Gynecologists are likely to be the professionals people turn to when facing these issues. Moreover, interest in further education in BDD and supportive materials for consultation on genital appearance was expressed by participants.

## Supplementary Information

Below is the link to the electronic supplementary material.

Supplementary file1 (PDF 1938 KB)

## Data Availability

Data is available in the supplementary material.
