# Supplementary material for: Body dysmorphic disorder of female genitalia: a qualitative study of Swiss obstetrician–gynecologists’ experiences and practices
Source: Arch Gynecol Obstet. 2021 Sep 30;305(2):379–87. doi: 10.1007/s00404-021-06270-w (PMC8840894; doi:10.1007/s00404-021-06270-w)
Supplement: Supplementary file 1 — Supplementary file1 (PDF 1938 KB) [file 404_2021_6270_MOESM1_ESM.pdf]

# Supplementary Material to:

# Body Dysmorphic Disorder of Female Genitalia: A Qualitative Study of Swiss Obstetrician-gynecologists' Experiences and Practices

**in**

**Archives of Gynecology and Obstetrics**

**Contents:**

Checklist to Self-Assess Studies Concerning Their Ethical Safety

COREQ-Checklist

Table S1: Demographic variables of participants

Table S2: Interview questions

Table S3: Full categorization system of content analysis

Full Transcript

**Checklist**

## Checklist to Self-Assess Studies Concerning Their Ethical Safety

Each person responsible for a study should fill out this checklist before data acquisition and decide, based on the outcome(s), if an application for approval to the Ethics Committee is necessary..

Title of the Study: Body Dysmorphic Disorder of Female Genitalia: A Qualitative Exploration of Obstetrician-gynecologists' Experiences and Practices

Principal Investigator (more than one possible): Dr. Marie Drüge

Responsible Person: Olenka Dworakowski

| | | **Yes** | **No** |
|---|---|---|---|
| 1 | Is there a possibility of disadvantages resulting for participants either through their behavior shown during the study or for not partaking in the study? | ☐ | ☒ |
| 2 | Is participation of persons with limited capacity/incapability of judgment or the participation of minors possible or intended? | ☐ | ☒ |
| 3 | Will it be necessary for people to partake in the study without consciously knowing so and without previously having given informed consent (e.g. covert observation of people in non-public spaces)? | ☐ | ☒ |
| 4 | Will participants be deliberately misinformed or falsely informed about the goals and execution of the project (e.g. by manipulated feedback about their performance)? | ☐ | ☒ |
| 5 | Will participants be asked to disclose personal experiences (e.g. stressful incidents), sensitive information (e.g. sexual behavior, drug use) or attitudes (e.g. political preferences)? | ☐ | ☒ |
| 6 | In case the physical integrity of participants is affected (e.g. by taking drugs, giving blood samples): Is there a possibility for adverse effects? | ☐ | ☒ |
| 7 | In case the psychological integrity of participants is affected (e.g. ability to concentrate, induction of negative emotions): Is there a possibility of adverse psychological effects? | ☐ | ☒ |
| 8 | In case the social integrity of participants is affected (e.g. group experiments): Is it possible for participation to cause adverse effects on the social level (e.g. reputation damage)? | ☐ | ☒ |

| | | | |
|---|---|---|---|
| 9 | Is there an additional financial incentive offered next to the usual compensation for participation in the study? | ☐ | ☒ |
| 10 | Does the research funding body require an assessment from an ethics committee? | ☐ | ☒ |
| 11 | Does the research funding body or the law require a project registration? | ☐ | ☒ |
| 12 | Has the study already been submitted to an ethics committee for assessment? | ☐ | ☒ |

Name of the committee:
Date of submission:
File reference number of approval (if known):

In case of "yes"-answers to any one question 1 through 11, it is mandatory to submit an application for approval of the study to the Ethics Committee (for psychological and related research)..

**Explanatory Comments on the Questions**

**Question 1**: Oftentimes study participants are students who are in dependent relationships with the principal investigator or their superiors (because the students will have to take exams with them and/or because the students are employed by them as tutors or assistants). In this case it is very important to make sure that there are no negative consequences due to a participation in the study – e.g. a worse grade if they don't complete the study in a satisfactory fashion. This can be achieved by ensuring the anonymity of the participant from the person whom they're dependent on. For example lecturers who grade students' performances should have none to minimal knowledge of students' identities who partake in their studies. If anonymity is guaranteed, question 1 is answered with NO.

**Question 2**: Examples for persons of limited capacity of judgment are children, mentally disabled people or people suffering from dementia or other psychiatric illnesses.

**Question 3**: This question refers to studies that observe or experimentally influence the behavior of people without their knowledge (for example in field studies or observations of people in non-public spaces).

**Question 4**: This question refers to studies that deliberately deceive participants ("deception"). That means that participants are purposefully misinformed or falsely informed about integral aspects of the study concerning them, such that they would reasonably perceive themselves as having been lied to once they find out the truth. Among other things, this can include incorrect feedback about their performance, incorrect information about the goals of the study, interactions with a co-worker of the researcher who is falsely introduced as "another study participant". This question does not refer to the fact that usually study participants aren't fully informed about the scientific background or the hypotheses of a study.

**Question 5**: This question refers to data acquisition that is sensitive due to one of two reasons. On the one hand, it's about information that needs to be treated highly confidentially because its distribution

could lead to disadvantages for the person (e.g. political views). On the other hand, it's about disclosed information that can be connected with strong emotions for the person (e.g. traumatic experiences) in a way that could cause the assessment to be an unacceptable emotional strain.

**Question 6:** This question refers to physical interventions, for example taking drugs (including alcohol) as well as invasive measures like taking blood samples, injection of contrasting agents. Unproblematic physical interventions are things like drinking non-alcoholic beverages, moderate exercise, measuring blood pressure.

**Question 7**: Just as in question 6, it's about distinguishing between negative and neutral consequences of the intervention. For example mood induction with happy or sad music is harmless because this type of music is common in everyday life and no adverse effects are to be expected. What would be disquieting or disturbing would be showing images of war or mutilation – it's true that those types of pictures can also be found in everyday life but usually one is not forced to look at them and they can be cause for very strong emotional reactions.

**Question 8:** Not every group experiment is ethically questionable but under certain conditions group experiments can possess a risk of placing people in uncomfortable situations, for example if an experiment creates a competitive situation in which individual participants will clearly lose, if aggression is induced, or if people consider the situation to be embarrassing. Just like in the two previous questions, here one must also draw a line between a small discomfort which can be considered comparable to an everyday occurrence and thus acceptable (e.g. nervousness some people always feel when talking in front of a group) and a discomfort that oversteps the boundary of what can be considered acceptable (e.g. being yelled at).

**Question 9:** Here the distinction is to be made between the standard compensation for a respective field of research for study participation and a financial incentive used specifically in the study to reach a certain goal of the study (e.g. particularly high performance motivation).

**Questions 10 and 11:** If the research funding body requires an assessment from an ethics committee the first place of contact is the Ethics Committee of the Faculty of Philosophy, provided that no other ethics committee is explicitly requested. The requirement to register is independent thereof.

**Question 12:** This question refers to ethics committees other than the one of the Faculty of Philosophy, e.g. out-of-canton ethics committees involved in multi-centered studies.

**In general: If in doubt, consult a member of the Ethics Committee of the Faculty of Philosophy.**

# COREQ (COnsolidated criteria for REporting Qualitative research) Checklist

A checklist of items that should be included in reports of qualitative research. You must report the page number in your manuscript where you consider each of the items listed in this checklist. If you have not included this information, either revise your manuscript accordingly before submitting or note N/A.

| Topic | Item No. | Guide Questions/Description | Reported on Page No. |
|---|---|---|---|
| **Domain 1: Research team and reflexivity** | | | |
| *Personal characteristics* | | | |
| Interviewer/facilitator | 1 | Which author/s conducted the interview or focus group? | 5 |
| Credentials | 2 | What were the researcher's credentials? E.g. PhD, MD | 5 |
| Occupation | 3 | What was their occupation at the time of the study? | 5 |
| Gender | 4 | Was the researcher male or female? | 5 |
| Experience and training | 5 | What experience or training did the researcher have? | 5 |
| *Relationship with participants* | | | |
| Relationship established | 6 | Was a relationship established prior to study commencement? | 4 |
| Participant knowledge of the interviewer | 7 | What did the participants know about the researcher? e.g. personal goals, reasons for doing the research | 4 |
| Interviewer characteristics | 8 | What characteristics were reported about the inter viewer/facilitator? e.g. Bias, assumptions, reasons and interests in the research topic | 4 |
| **Domain 2: Study design** | | | |
| *Theoretical framework* | | | |
| Methodological orientation and Theory | 9 | What methodological orientation was stated to underpin the study? e.g. grounded theory, discourse analysis, ethnography, phenomenology, content analysis | 6 |
| *Participant selection* | | | |
| Sampling | 10 | How were participants selected? e.g. purposive, convenience, consecutive, snowball | 4 |
| Method of approach | 11 | How were participants approached? e.g. face-to-face, telephone, mail, email | 4 |
| Sample size | 12 | How many participants were in the study? | 4 |
| Non-participation | 13 | How many people refused to participate or dropped out? Reasons? | 4 |
| *Setting* | | | |
| Setting of data collection | 14 | Where was the data collected? e.g. home, clinic, workplace | 5 |
| Presence of non-participants | 15 | Was anyone else present besides the participants and researchers? | 5 |
| Description of sample | 16 | What are the important characteristics of the sample? e.g. demographic data, date | 5 / supplement |
| *Data collection* | | | |
| Interview guide | 17 | Were questions, prompts, guides provided by the authors? Was it pilot tested? | 5 |
| Repeat interviews | 18 | Were repeat inter views carried out? If yes, how many? | - |
| Audio/visual recording | 19 | Did the research use audio or visual recording to collect the data? | 5 |
| Field notes | 20 | Were field notes made during and/or after the inter view or focus group? | 5 |
| Duration | 21 | What was the duration of the inter views or focus group? | 5 |
| Data saturation | 22 | Was data saturation discussed? | 11 |
| Transcripts returned | 23 | Were transcripts returned to participants for comment and/or | 5 |

| Topic | Item No. | Guide Questions/Description | Reported on Page No. |
|---|---|---|---|
| | | correction? | |
| **Domain 3: analysis and findings** | | | |
| *Data analysis* | | | |
| Number of data coders | 24 | How many data coders coded the data? | 6 |
| Description of the coding tree | 25 | Did authors provide a description of the coding tree? | 6 |
| Derivation of themes | 26 | Were themes identified in advance or derived from the data? | 6 |
| Software | 27 | What software, if applicable, was used to manage the data? | 6 |
| Participant checking | 28 | Did participants provide feedback on the findings? | 6 |
| *Reporting* | | | |
| Quotations presented | 29 | Were participant quotations presented to illustrate the themes/findings? Was each quotation identified? e.g. participant number | 6-10 |
| Data and findings consistent | 30 | Was there consistency between the data presented and the findings? | 6-10 |
| Clarity of major themes | 31 | Were major themes clearly presented in the findings? | 6-10 |
| Clarity of minor themes | 32 | Is there a description of diverse cases or discussion of minor themes? | 6-15 |

Developed from: Tong A, Sainsbury P, Craig J. Consolidated criteria for reporting qualitative research (COREQ): a 32-item checklist for interviews and focus groups. *International Journal for Quality in Health Care*. 2007. Volume 19, Number 6: pp. 349 – 357

**Once you have completed this checklist, please save a copy and upload it as part of your submission. DO NOT include this checklist as part of the main manuscript document. It must be uploaded as a separate file.**

Table S1

*Demographic variables of participants*

| IP | f /m | Age | Title | Further education | Work experienc e | Work location | Conducts surgeries |
|---|---|---|---|---|---|---|---|
| 1 | f | 32 | Assistant OB-GYN | None | 5 | Clinic | Yes |
| 2 | f | 51 | OB-GYN | Operative OB-GYN | 25 | Practice | Yes |
| 3 | f | 39 | General inner medicine | None | 13 | Practice | No |
| 4 | f | 57 | General inner medicine | Psychosomatic and psychosocial medicine sports medicine sexual medicine | 31 | Shared practice | No |
| 5 | m | 49 | OB-GYN | Operative OB-GYN | 20 | Practice | Yes |
| 6 | f | 43 | OB-GYN | Operative OB-GYN and senology SGGG senior-mamma surgeon | 18 | Clinic | Yes |
| 7 | m | 65 | OB-GYN | None | 39 | Clinic and shared practice | Yes |
| 8 | f | 39 | OB-GYN | Operative OB-GYN | 12 | Clinic | Yes |
| 9 | f | 48 | Practicing physician | Child and adolescent gynecology | 20 | Shared practice | No |
| 10 | f | 40 | General inner medicine | None | 13 | Shared practice | No |
| 11 | m | *NA* | OB-GYN | Operative OB-GYN and senology SGGG body psychotherapy sexual therapy | 39 | Shared practice | Yes |

*Note*. IP stands for interview partner. f is an abbreviation for female, m is an abbreviation for male. OB-GYN stands for obstetrician-gynecologist, this is a professional doctor specializing in female reproductive health. Work experience is stated in years

Table S2

*Interview Questions*

| Questions |
|---|
| 1. With what kind of mental health issues do patients come to your office? |
| 2. Please read the following case vignette: *Lea is a 25-year old woman. Since she was 13 years old, her thoughts have circled around her ‚big a misshapen' nose, her ‚small hanging breasts' and her ‚ugly' hair. But, in reality Lea is an attractive woman with none such defects. ‚Every second' she thinks about her defects and spends hours checking her appearance in the mirror. She's always comparing herself to others and constantly asks her mother if she looks normal. Lea suffers strongly from her insecurities and avoids social activities, such as parties or dates because of them and does not go to university regularly anymore.* What psychiatric disorders come to mind reading this example case? |
| 3. What are your experiences with patients that ask questions to the aesthetics or normality of their own genitals? |
| 4. What is your professional procedure with such questions? |
| 5. Do you ask your patients how they feel about the aesthetic of their genitalia? |
| 6. What are your experiences with patients that with operations of their genitalia? |
| 7. What is your professional procedure with such wishes? |
| 8. Please read the following case vignette: *Mia is a 21-year-old Patient worries a lot about the appearance of her genitalia: her labia minora is too long, as they are visible over the labia majora. Also, they are asymmetrical. This worries her a lot. In an examination her labia show a normal length. The patient reports checking her labia daily, sometimes more than once, with help of a mirror. She is very ashamed of her genitalia. She avoids undressing in public changing rooms, or letting anyone see her naked. Often she asks her boyfriend if her labia look normal. He always reassures her that they are normal. Slowly the questions are starting to annoy him.* What are your experiences with patients who show such symptoms? |
| 9. What would your professional procedure be with such a patient? |
| 10. How strong do you estimate is the interest of gynecologists in further education of BDD? |
| 11. How strong do you estimate is the interest of gynecologists or the benefit of aid in diagnostics of BDD, for example in form of a screening instrument? |
| 12. How strong do you estimate is the interest of gynecologists in aid for consulting women who show symptoms of BDD of their genitalia? |

*Note*. Questions were originally in German and have been translated for this publication

Table S3

Full categorization system of content analysis

| RQ | Broader category | Category | I/D | Explanation | Example | *N* | In *N* Files |
|---|---|---|---|---|---|---|---|
| RQ1 | Disorders which gynecologists are confronted with<br><br>(*Genannte Störungen mit denen Gynäkolog_innen in Kontakt kommen)* | Depressive episode (F32) and depressive disorder (F33) (*Depressive Episode und rezidivierende depressive Störung*) | D | | Transkript_IP01_060519 [1251:1264]:<br>Depressions<br>*(Depressionen)* | 8 | 7 |
| | | Remaining category disorders (*Restkategorie Störungen*) | D | | Transkript_IP03_090519 [468:502]:<br>[My patients may present] just basic overburden.<br>*([Meine Patientinnen können] einfach grundsätzlich Überlastung [zeigen].)* | 12 | 6 |
| | | Phobic disorder (F40) and other anxiety disorders (F41) (*Phobische Störungen und andere Angststörungen*) | D | | Transkript_IP01_060519 [1238:1252]:<br>Anxiety disorders<br>*(Angststörungen)* | 6 | 5 |
| | | Nonorganic sleep disorders (F51) (*Nichtorganische Schlafstörungen* ) | D | | Transkript_IP03_090519 [999:1038]:<br>(…) a lot, often [depressions] connected with sleep disorders.<br>*((…) viel, oft [Depressionen] verknüpft mit Schlafstörungen.)* | 4 | 4 |
| | | Reaction to severe stress, and adjustment disorders (F43) (*Reaktionen auf schwere Belastungen und Anpassungsstörungen*) | D | | Transkript_IP05_160519 [324:402]:<br>(…) currently I have a patient with an adjustment disorder.<br>*((…) aktuell habe ich eine Patientin mit einer Anpassungsstörung.)* | 3 | 3 |

| | | | | | | |
|---|---|---|---|---|---|---|
| | Somatoform disorders (F45) (*Somatoforme Störungen*) | D | | Transkript_IP01_060519 [823:1038]:<br>[Psychological disorders in gynecology] Are just often psychosomatic complaints (…).<br>*([Psychische Störungen in der Gynäkologie] Sind halt häufig psychosomatische Beschwerden (...).)* | 3 | 3 |
| | Sexual dysfunction, not caused by organic disorder or disease (F52) (*Sexuelle Funktionsstörungen, nicht verursacht durch eine organische Störung oder Krankheit*) | D | | Transkript_IP04_100519 [866:895]:<br>(…) often I hear [from my patients] lack of libido.<br>*((...) häufig hör ich Libidomangel [von meinen Patientinnen].)* | 5 | 3 |
| | Eating disorders (F50) (*Essstörungen*) | D | | Transkript_IP07_240519 [715:727]:<br>[I am confronted with] Eating disorders.<br>*([Ich treffe] Essstörungen [an].)* | 2 | 2 |
| | Schizophrenia (F20) (*Schizophrenie*) | D | | New Transkript_IP01_060519 [1272:1302]:<br>Or [I have] also already [experienced patients with] schizophrenia.<br>*(Oder [ich habe] auch schon [Patientinnen erlebt mit] Schizophrenie.)* | 1 | 1 |
| | Mental and behavioural disorders associated with the puerperium, not elsewhere classified (F53) (*Psychische oder Verhaltensstörungen im Wochenbett, anderenorts nicht klassifiziert*) | D | | New Transkript_IP01_060519 [1328:1462]:<br>And what then naturally also comes up [with my patients] in puerperium: Depressions in puerperium or also then in puerperium that a psychosis developes.<br>*(Und was dann natürlich noch dazu kommt [bei meinen Patientinnen] im Wochenbett: Wochenbettdepression oder auch dann im Wochenbett sich eine Psychose entwickelt.)* | 1 | 1 |
| | Body dysmorphic disorder (F45.2) (*Körperdysmorphe Störung*) | D | | None | 0 | 0 |
| Symptoms identified after | Decreased feeling of self-worth and self-confidence | D | | New Transkript_IP01_060519 [3943:3993]:<br>And ehm, in any case [the woman from case vignette is] missing self-confidence. | 12 | 10 |

| | | | | | | |
|---|---|---|---|---|---|---|
| reading case vignette 1<br><br>*(Genannte Symptome nach dem Lesen der Vignette 1)* | *(vermindertes Selbstwertgefühl und Selbstvertrauen)* | | | *(Und ähm, auf jeden Fall [hat die Frau aus der Fallvignette] fehlendes Selbstvertrauen.)* | | |
| | Issues with body perception *(Körperwahrnehmungsstörung)* | D | | Transkript_IP03_090519 [2474:2546]:<br>Okay, yes this is yes actually really a wrong body perception. [is talking about case vignette]<br>*(Okay, ja das ist ja eigentlich wirklich eine falsche Körperwahrnehmung [spricht über Fallvigentte])* | 5 | 5 |
| Syndromes identified after reading case vignette 1<br><br>*(Genannte Syndrome nach dem Lesen der Vignette 1)* | Depressive episode (F32) and depressive disorder (F33) *(Depressive Episode und rezidivierende depressive Störung)* | D | | New Transkript_IP01_060519 [4103:4276]:<br>Could surely [be] something depressive.<br>*((...) könnte sicher irgendwas, depressives [sein].)* | 4 | 3 |
| | Phobic disorder (F40) and other anxiety disorders (F41) *(Phobische Störungen und andere Angststörungen)* | D | | Transkript_IP07_240519 [5035:5085]:<br>She [from case vignette 1] is becoming phobic, right? Phobias from life.<br>*(Sie [aus der Fallvignette 1] wird phobisch, oder? Phobien, vor dem Leben.)* | 2 | 2 |
| | Eating disorders (F50) *(Essstörungen)* | D | | Transkript_IP09_060619 [2682:2716]:<br>No idea if she [from case vignette 1] also [has] an eating disorder<br>*(Keine Ahnung ob sie [aus Fallvignette 1] noch eine Essstörung [hat])* | 2 | 1 |
| | Obsessive compulsive disorders (F42) *(Zwangsstörung)* | D | | Transkript_IP09_060619 [6444:6458]:<br>Obsessive compulsive disorder [comes to my mind with this case vignette 1].<br>*([Ich komme bei dieser Fallvignette 1 auf] Zwangsstörung.)* | 1 | 1 |
| Naming of correct diagnosis for vignette | Reasoning (*Begründung)* | I | Reasoning of IP why a diagnosis cannot be given. | Transkript_IP02_080519 [3459:3559]:<br>I don’t make any psychological diagnosis. Because I find I am not a psychiatrist, not a psychologist. | 7 | 7 |

| | | | | | | | |
|---|---|---|---|---|---|---|---|
| | (*Korrekte Nennung der Diagnose der Vignette)* | | | | *(Ich mache keine psychischen Diagnosen. Weil ich finde ich bin keine Psychiaterin, keine Psychologin.)* | | |
| | | Body dysmorphic disorder (F45.2) *(Körperdysmorphe Störung)* | D | Explicit naming of the correct disorder (BDD) after reading case vignette. | None | 0 | 0 |
| | BDD known or unknown (*KDS bekannt oder nicht bekannt)* | BDD known *(KDS bekannt)* | D | IP knows BDD or has heard of it. | Transkript_IP05_160519 [4363:4390]: I have heard of it [BDD] already. *([KDS] Habe ich auch schon gehört.)* | 5 | 5 |
| | | BDD unknown *(KDS unbekannt)* | D | IP does not know BDD or has never heard of it. | Transkript_IP02_080519 [5040:5298]: Well. No, well I, I really don't know all these diagnosis. *(Also. Nein, eben ich, ich kenn die ganzen Diagnosen wirklich nicht.)* | 5 | 5 |
| RQ2 | Experiences with questions regarding the aesthetics or normality of genital area *(Erfahrungen mit Fragen zur Ästhetik oder Normalität des Genitalbereichs)* | Many questions *(Viele Fragen)* | D | Many experiences with patients who ask questions about the aesthetics or normality of genitalia. | Tranksript_IP05_160519 [8664:8904]: So it [aesthetics of genitalia] is definitely a topic, always and always again. *(Also das [Ästhetik des Genitalbereichs] ist schon ein Thema, also immer und immer wieder.)* | 5 | 5 |
| | | Several questions *(Einige Fragen)* | D | Several experiences with patients who ask questions about the aesthetics or normality of genitalia. | None | 0 | 0 |
| | | Seldom questions *(Seltene Fragen)* | D | Few experiences with patients who ask questions to the aesthetics or normality of genitalia. | Transkript_IP01_060519 [11615:11670]: Seldom. So, really seldom that it [aesthetics of genitalia] is brought up. *(Selten. Also wirklich selten, dass es [Ästhetik des Genitalbereichs] thematisiert wird.)* | 3 | 3 |
| | | No questions *(Keine Fragen)* | D | No experiences with patients who ask questions to the aesthetics or normality of genitalia. | Transkript_IP07_240519 [17929:18040]: With the rest [aesthetics of genitalia] I don't have any questions [of patients], unless it concernes the somatic area but psychologically I do not have any. *(Beim Rest [Ästhetik des Genitalbereichs] habe ich keine Fragen [von Patientinnen], ausser es betrifft den somatischen Bereich aber psychologisch habe ich keine.)* | 1 | 1 |
| | Experiences with wishes for plastic surgery of genital area | Many operations *(Viele OP)* | D | Many experiences with patients who present wishes for plastic surgery of genital area. | Transkript_IP04_100519 [13373:13484]: Yes. Ehm. Yes, there are, there are also many different stories [about plastic surgery of genitalia] that come to my mind like this. | 1 | 1 |

| | | | | | | |
|---|---|---|---|---|---|---|
| *(Erfahrungen mit Wünschen nach Schönheitsoperationen des Genitalbereichs)* | | | | *(Ja. Ehm. Ja, da gibt es, gibt es auch viele ganz verschiedene Geschichten [zu Schönheitsoperationen des Genitalbereichs] die mir so durch den Kopf gehen.)* | | |
| | Several operations *(Einige OP)* | D | Several experiences with patients who present wishes for plastic surgery of genital area. | Tranksript_IP05_160519 [10188:10245]: Well it [plastic surgery of genitalia] exists, yes (.) I can only tell you examples. *(Also [Schönheitsoperationen des Genitalbereichs] gibt es, ja (.) Da kann ich nur Beispielhaft sagen.)* | 1 | 1 |
| | Seldom operations *(Seltene OP)* | D | Seldom experiences with patients who present wishes for plastic surgery of genital area. | Transkript_IP03_090519 [13640:13687]: Ehm, I don't have many that wish that [plastic surgery of genitalia]. *(Ehm, ich hab nicht sehr viele die das [Schönheitsoperationen des Genitalbereichs] wünschen)* | 6 | 5 |
| | No operations *(Keine OP)* | D | No experiences with patients who present wishes for plastic surgery of genital area . | None | 0 | 0 |
| Experiences with symptoms of BDD of genitalia | Many BDD *(Viele KDS)* | D | Many experiences with patients who show symptoms of BDD of genitalia. | Transkript_IP09_060619 [19817:19851]: Many [experiences], now exactly in this [genital-]area. *(Viele [Erfahrungen], jetzt genau in dem [Genital-] Bereich.)* | 3 | 3 |
| *(Erfahrungen mit Symptomatik KDS des Genitalbereichs)* | Several BDD *(Einige KDS)* | D | Several experiences with patients who show symptoms of BDD of genitalia. | Transkript_IP06_160519 [31456:31845]: Concerning the genital area, I think I see a patient with such a question every three months [about dissatisfaction with it]. *((...) was den Genitalbereich angeht, denk ich, ich seh' alle drei Monate 'ne Patientin mit der Fragestellung [zur Unzufriedenheit damit].)* | 1 | 1 |
| | Seldom BDD *(Seltene KDS)* | D | Seldom experiences with patients who show symptoms of BDD of genitalia. | Transkript_IP06_160519 [26642:26793]: It comes up seldom that they [patients] bring it up directly and say 'I have the feeling that it, it [my genitalia] isn't normal and ahm, how do you see it?' *(Es kommt seltener vor, dass sie [Patientinnen] direkt das ansprechen und sagen 'Ich hab das Gefühl, das sie, sie [mein Genital] ist nicht normal und ähm, wie stehen Sie dazu?'.)* | 5 | 4 |
| | No BDD *(Keine KDS)* | D | No experiences with patients who show symptoms of BDD of genitalia. | Transkript_IP03_090519 [15407:15537]: But I don't know it [patients with symptoms of BDD of genitalia] from my own experience. *(Aber ich kenn das [Patientinnen mit Symptomen der KDS des Genitalbereichs] jetzt nicht aus eigener Erfahrung.)* | 1 | 1 |

| | | | | | | |
|---|---|---|---|---|---|---|
| Frequencies of experiences<br><br>*(Häufigkeiten der Erfahrungen)* | Weekly<br>*(Wöchentlich)* | I | At least every week such issues are presented by patients. | Transkript_IP08_040619 [14447:14524]:<br>The questions [about dissatisfaction with genital appearance] with us are for sure two or three times a week.<br>*(Die Anfragen [zu Unzufriedenheit mit Aussehen des Genitals] bei uns, sicher zwei, drei pro Woche.)* | 2 | 2 |
| | Monthly<br>*(Monatlich)* | I | At least every month, or a few times a month, such issues are presented. | Transkript_IP03_090519 [15715:15754]:<br>Every month one, two, three times [I am confronted with Symptoms of BDD concerning genitalia]<br>*(Jeden Monat eins, zwei, drei mal [werde ich mit Symptomen der KDS betreffend des Genitalbereichs konfrontiert])* | 3 | 3 |
| | Yearly<br>*(Jährlich)* | I | Such issues are presented only a few times a year. | Transkript_IP09_060619 [15386:15424]:<br>Well, that someone comes [because of genital dissatisfaction] every half a year.<br>*(Also, dass jemand kommt [wegen genitaler Unzufriedenheit], all Halbjahr.)* | 2 | 2 |
| | 5 % | I | An estimate of 5% of patients present such issues. | Transkript_IP04_100519 [18587:18707]:<br>I would say maximum five percent, so five in a hundred women [that show symptoms of BDD concerning Genitalia].<br>*(Ich würde sagen maximal fünf Prozent, also das wären fünf Frauen von hundert [die KDS Symptome betreffend Genitalbereich zeigen].)* | 1 | 1 |
| Tendencies<br><br>*(Tendenzen)* | Increasing<br>*(Zunehmend)* | D | Such problems seem to be increasing compared to the past. | Transkript_IP06_160519 [19644:19720]:<br>For one, that it [genital dissatisfaction] is increasing.<br>*(Zum einen, dass es [genitale Unzufriedenheit] zu nimmt.)* | 8 | 5 |
| | Decreasing<br>*(Abnehmend)* | I | Such problems seem to be decreasing compared to the past. | Transkript_IP02_080519 [16677:16883]:<br>Interestingly, I would say now in my office hours in the last one, two years almost already less often than, than still before.<br>*(Interessanterweise würde ich jetzt sagen in meiner Sprechstunde in den letzten ein, zwei Jahren weniger häufig schon fast wieder als, als noch davor.)* | 1 | 1 |
| Areas of Concern<br><br>*(Problembereiche)* | Labia *(Labien)* | D | Labia are mentioned as being of issue for patients. Labia minora are perceived as too large. | Transkript_IP06_160519 [20912:20950]:<br>(…) very large labia for example.<br>*((…) sehr grosse Schamlippen beispielsweise)* | 15 | 11 |
| | Labia mentioned<br>*(Labien erwähnt)* | I | Labia are mentioned by IP before Interviewers ask about it specifically. | Transkript_IP06_160519 [640:687]:<br>[Patients] are not so satisfied, right, in the genital area.<br>*([Patientinnen ]nicht so zufrieden sind, oder, im Genitalbereich.)* | 7 | 7 |
| | Breasts *(Brüste)* | I | Breasts are mentioned as being of issue for patients. | New Transkript_IP01_060519 [13705:14023]:<br>(…) when the breasts are large or so.<br>*((…) wenn die Brüste gross sind oder so.)* | 9 | 5 |

| | | | | | | |
|---|---|---|---|---|---|---|
| | Other operations *(Andere Operationen)* | I | Other operations or other body parts, which patients could be concerned with are mentioned. | Transkript_IP07_240519 [19734:19875]: Rejuvenations, renewing operations I have none, because there I also do not participate. *(Verjungfräulich- Erneuerungsoperationen habe ich keine, weil da mache ich auch nicht mit.)* | 3 | 3 |
| Influencing factors *(Einflussfaktoren)* | Younger *(Jüngere)* | I | Younger women are mentioned specifically. | Transkript_IP09_060619 [13784:13827]: (…) I also already have had very young women [with issues]. *((...) ich hatte auch schon ganz junge Frauen [mit Problemen].)* | 11 | 6 |
| | Media *(Medien)* | D | Media as an influencing factor is mentioned. | Transkript_IP06_160519 [31772:31849]: Well I think, that, here the medial world really does have a big part in it. *(Also ich glaube, dass, da hat die mediale Welt schon ein grossen Anteil dran.)* | 9 | 6 |
| | Pregnancy/Birth *(Schwangerschaft/Geburt)* | I | | Transkript_IP10_060619 [10934:11025]: I know that with older women it [genital dissatisfaction] is almost a bigger problem after the births. *(Ich weiss, dass es [Unzufriedenheit mit Genitalien] bei den älteren Frauen fast ein grösseres Problem ist nach den Geburten.)* | 7 | 6 |
| | Culture *(Kultur)* | I | Cultural aspects were taken into consideration. | Transkript_IP08_040619 [12605:13724]: Here, that is really interesting, because that really goes back to the culture. *(Da, das ist wirklich ganz interessant, weil das geht ja wirklich zurück auf die Kultur.)* | 8 | 4 |
| | Shaving of genital area *(Intimrasur)* | I | Topic of shaving the genital area was mentioned. | Transkript_IP10_060619 [8399:8492]: It is also about the shaving. I mean there I only saw shaved, fully shaved women. *(Es geht auch um die Rasur. Ich meine dort hab ich nur rasierte, vollrasierte Frauen gesehen.)* | 7 | 4 |
| | Comment *(Bemerkung)* | I | Women are influenced by comments of others. This may often be a sexual partner, but does not have to be. | Transkript_IP03_090519 [10493:10738]: And often this question [about aesthetics of genitalia] is only triggered by some encounter or by some comment, (…). *(Und oft wird diese Frage [über die Ästhetik des Genitalbereichs] auch erst ausgelöst durch irgend eine Begegnung oder durch Bemerkungen, (...).)* | 7 | 4 |
| | Uninformed *(Uninformiert)* | D | Patients are not well informed about female genitalia. | Transkript_IP09_060619 [19939:20014]: (…) that is like not really clear that that [their genitalia] [is normal], because they [patients] don't know what is normal. | 7 | 4 |

| | | | | | |
|---|---|---|---|---|---|
| | | | *((…) das ist ja wie nicht klar, dass das [ihr Genital] [normal ist], weil sie [die Patientinnen] nicht wissen, was normal ist.)* | | |
| Insecure *(Unsicher)* | I | Idea that insecure women are unsatisfied about the aesthetics of their genitalia. | Transkript_IP03_090519 [10446:10492]:<br>That they are basically insecure patients [that ask about the aesthetics of genitalia].<br>*(Das grundsätzlich unsichere Patientinnen sind [die über der Ästhetik der Genitalien fragen].)* | 4 | 4 |
| Unrealistic ideal *(Unrealistisches Ideal)* | I | Women have an unrealistic ideal what their genital is supposed to look like. | Transkript_IP11_280619 [12795:12941]:<br>Aesthetic ideas [of genitalia] that are given, that quasi are seen as normal, it really is not normal.<br>*(Ästhetische Vorstellungen [des Genitalbereichs] die gegeben werden, die man so quasi als normal sieht, ist gar nicht normal.)* | 4 | 3 |
| Traumatic experiences *(Traumatische Erfahrungen)* | I | | Transkript_IP07_240519 [24903:25046]:<br>Sure then one can still go searching, if she had an unfortunate defloration or abuse developed, then it is not only this problem.<br>*(Klar kann man dann noch suchen gehen, wenn sie eine unglückliche Defloration oder Missbrauch entwickelt, dann ist es nicht nur dieses Problem.)* | 3 | 3 |
| Age *(Alter)* | I | Age as an influencing factor is mentioned. | Tranksript_IP05_160519 [13590:13755]:<br>I would say that it [genital dissatisfaction] exists also with ehm somewhat older women that they, that they are bothered by it.<br>*(Ich würde sagen das [Unzufriedenheit mit dem Genitalbereich] gibt es auch bei ehm etwas reiferen Frauen, dass sie das, dass sie sich darüber stören.)* | 3 | 3 |
| Changes *(Veränderungen)* | I | General biological changes. | Transkript_IP11_280619 [12074:12148]:<br>Especially like, yes after puberty it comes of often, these, these questions [to the aesthetics of genitals].<br>*(Gerade so, ja, nach der Pubertät kommt das häufig so, so Fragestellungen [zur Ästhetik der Genitalien].)* | 3 | 3 |
| Type *(Typ)* | I | Idea that a specific type of woman is unsatisfied about the aesthetics of her genitalia | Transkript_IP02_080519 [25265:25437]:<br>(…) it is a specific type of woman [that show symptoms of BDD concerning genitalia].<br>*((…) es ist ein bestimmter Typ Frauen [die Symptome der KDS betreffend Genitalien zeigen].)* | 2 | 2 |
| Gender role *(Rollenbild)* | I | Gender specific stereotypes of societal roles were mentioned. | Tranksript_IP05_160519 [8249:8363]:<br>The role distribution, the woman has, she has to be liked and that is somehow something really old, they make themselves pretty. | 1 | 1 |

| | | | | | | |
|---|---|---|---|---|---|---|
| | | | | *(Die Rollenverteilung, die Frau hat, sie muss gefallen und das ist irgendwie etwas uraltes, sie machen sich schön.)* | | |
| Symptoms<br><br>*(Symptome)* | Distorted perception *(Verzerrte Wahrnehmung)* | D | A discrepancy between how the patient perceives the situation and what the IP sees is experienced, | Transkript_IP07_240519 [22491:22520]:<br>An imagined [dysmorphia].<br>*(Eine Eingebildete [Dysmorphie].)* | 5 | 4 |
| | Shame *(Scham)* | D | The feeling of shame. | Transkript_IP10_060619 [12447:12674]:<br>(…) she is ashamed in the changing room, she is ashamed with her boyfriend (…)<br>*((...) sie schämt sich in der Umkleidekabine, sie schämt sich mit dem Freund, (...))* | 4 | 4 |
| | Lacking insight *(Fehlende Einsicht)* | D | It appears difficult to change the patients mind or they insist on their point of view. | Transkript_IP01_060519 [16102:16318]:<br>(…) here I am probably talking against a wall if I tell her [a patient] that.<br>*((...) da red ich wahrscheinlich gegen eine Wand wenn ich ihr [einer Patientin] das sag.)* | 4 | 2 |
| | Comparing *(Vergleichen)* | D | Patients compare their genitalia to others. | Transkript_IP11_280619 [11906:12073]:<br>Or they [patients] compare themselves, that is often then when that comes up.<br>*(Oder sie [Patientinnen] vergleichen sich, das ist ja häufig dann wenn das kommt.)* | 1 | 1 |
| Opinions on and experiences with plastic surgery of genitalia<br><br>(Meinungen und Erfahrungen zu Schönheitsoperationen des Genitalbereichs) | Mention operation *(Erwähnen OP)* | I | IP mentions operations without being asked about it directly. | Transkript_IP02_080519 [17234:17285]:<br>An operation normally does not make it [unsatisfaction with genital area] better.<br>*(Eine Operation mach das [Unzufriedenheit mit Genitalbereich] in der Regel nicht besser.)* | 8 | 8 |
| | Makes sense *(Sinnvoll)* | I | Opinion that plastic surgery of genitalia can make sense in certain situations. | Transkript_IP09_060619 [19083:19180]:<br>When it clearly is something, that just has to be taken off [of the genitalia], then I sign them [patients] directly up for an operation.<br>*(Wenns klar etwas ist, das man einfach [vom Genital] wegnehmen muss, dann melde ich sie [Patientinnen] direkt zur Operation an.)* | 12 | 7 |
| | Money *(Gelder)* | I | Costs of operations are mentioned, mostly in connection to health insurances. | Transkript_IP03_090519 [13862:14018]:<br>(…) if that [the operation] somehow will be covered by a [medical] insurance and that is yes well that needs a mostly a pretty good indication so that there something gets covered.<br>*((...) ob das [die Operation] irgendwie übernommen wird von einer [kranken-]Kasse und das ist ja ehm also das braucht ja meistens eine ziemlich gute Indikation dass da etwas übernommen wird.)* | 10 | 6 |

| | | | | | | |
|---|---|---|---|---|---|---|
| | Aversion towards surgery *(Abneigung Chirurgie)* | I | Skepticism or aversion toward plastic surgery or plastic surgeons. | Transkript_IP11_280619 [19704:19845]:<br>(…) then exactly also invite [to the further education] these aesthetes [plastic surgeons] who profit from it [surgery], because me, I have, anger always comes up in me, they take advantage of it [women who wish plastic surgery].<br>*((...) dann gleich noch diese Ästheten [plastische Chirurgen] noch einladen [zur Fortbildung], die davon [Operationen] profitieren, weil mich, ich hab, mir kommt immer Wut dann rauf, die nutzen das [Frauen die OP wünschen] aus.)* | 5 | 4 |
| | Complications *(Komplikationen)* | I | Experiences with complications after a labiaplasty surgery. | Transkript_IP11_280619 [17028:17117]:<br>I say:,when you have complications [with a labiaplasty] you can come to me'. That also exists right.<br>*(Sage ich ,wenn Sie Komplikationen [mit einer Labienverkleinerung] haben, können Sie dann zu mir kommen'. Gibts ja auch.)* | 4 | 4 |
| | Directly operation *(Direkt OP)* | I | Patients have already been operated on when they come go gynecologists office. | Transkript_IP06_160519 [27637:28140]:<br>(…) they [patients] then go directly to the aesthetic surgeon who offers this in this portfolio, genital improvement and ehm, because they of course then also have less ehm, hurdles to go through.<br>*((...) die [Patientinnen] gehen dann zum ästhetischen Chirurg, der das in seinem Portefeuille anbietet, Genitalverschönerung und ähm, weil sie natürlich dann auch weniger ähm, Hürden zu durchlaufen haben.)* | 4 | 4 |
| | Complicated *(Kompliziert)* | I | Opinion that labiaplastic surgery is complicated. | Transkript_IP02_080519 [21147:21220]:<br>Well as banal as it is, it [labiaplasty] is a difficult aesthetic operation.<br>*(Also so banal dass das ist, es [Labienverkleinerung] ist eine schwierige ästhetische Operation.)* | 3 | 2 |
| | Simple *(Simpel)* | I | Opinion that labiaplastic surgery is simple. | Tranksript_IP05_160519 [12977:13173]:<br>[a labiaplasty ]Shouldn't be a problem, it is not a big deal.<br>*([eine Labienverkleinerung] Sollte eigentlich kein Problem sein, es ist keine grosse Geschichte.)* | 2 | 2 |
| | Satisfied *(Zufriedenheit)* | I | Experiences with satisfied patients after a labiaplasty surgery. | Transkript_IP07_240519 [19597:19649]:<br>(…) [I experience] only like labiaplasty and those are often satisfied.<br>*((...) [Ich erfahre] nur so Labienverkleinerungen und die sind zufrieden.)* | 2 | 2 |
| Further Topics<br><br>*(Sonstige Themen)* | Medical issues *(Medizinische Beschwerden)* | I | Possible medical issues of the genital area are mentioned. | Transkript_IP03_090519 [10976:11164]:<br>And then there also really exist the very obvious malformations [of the genital area] (…). | 19 | 10 |

| | | | | | |
|---|---|---|---|---|---|
| | | | *(Und dann gibt es aber schon auch die ganz eindeutige Fehlbildungen [des Genitalbereichs] (...).)* | | |
| Sexuality<br>*(Sexualität)* | I | | Transkript_IP08_040619 [14249:14389]:<br>(…) that it [labia] bothers during sex, that they are pulled in, (…).<br>*((...) dass es [Labien] beim Sex stört, dass die reingezogen werden, (...).)* | 10 | 7 |
| Subclinical dissatisfaction<br>*(Subklinische Unzufriedenheit)* | D | IP does not rate symptoms as pathological but rather as a subclinical dissatisfactions. | Transkript_IP11_280619 [23646:23693]:<br>Well that [dissatisfaction with genitalia] can't only be seen as a disorder.<br>*(Also das [Unzufriedenheit mit dem Genitalbereich] kann man nicht nur als Störung sehen.)* | 9 | 7 |
| Psychological issues in the background<br>*(Psychische Probleme im Hintergrund)* | D | Behind somatic issues there often lie psychological problems. | Transkript_IP11_280619 [15511:15574]:<br>(…) or that one realizes that maybe more lies behind it [the medical problem], right.<br>*((...) oder das man merkt, dass da [beim medizinischen Problem] vielleicht etwas dahintersteckt oder.)* | 9 | 5 |
| Relationship<br>*(Beziehung)* | I | Romantic relationships or relationship issues are talked about. | Transkript_IP04_100519 [17932:18117]:<br>But she [patient] of course also burdens the relationship with her insecurity.<br>*(Aber sie [Patientin] belastet natürlich auch die Beziehung mit ihrer Unsicherheit.)* | 6 | 4 |
| Person of trust<br>*(Vertrauens-person)* | D | Gynecologists are portrayed as a person of trust to their patients. | Transkript_IP07_240519 [27106:27329]:<br>Because these [psychological problems that patients want to talk about] come from the first day on, I can guarantee you that.<br>*(Weil diese [psychischen Probleme, die Patientinnen besprechen möchten] kommen vom ersten Tag an, das kann ich Ihnen garantieren.)* | 5 | 4 |
| Mutilation<br>*(Verstümmelung)* | I | | Transkript_IP10_060619 [13744:14066]:<br>We see of course many with genital mutilation at our place right, because we have all the asylum seekers.<br>*(Wir sehen natürlich viele mit Genital Mutilation bei uns oder, weil wir die ganzen Asylanten haben.)* | 3 | 3 |
| Rather open themselves to women<br>*(Öffnen sich eher Frauen gegenüber)* | I | Patients would rather talk to female gynecologists about their issues. | Transkript_IP08_040619 [19490:19625]:<br>There are women, who do open themselves up to men but eh, that, the empathy is maybe, is really an advantage, mhm.<br>*(Es gibt Frauen, die öffnen sich schon Männern gegenüber aber äh, das, die Nachempfindung ist vielleicht schon, ist schon Vorteil, mhm.)* | 2 | 2 |

| | | | | | | |
|---|---|---|---|---|---|---|
| | Comments on Experiences<br><br>*(Bemerkungen zu Erfahrungen)* | Specific example *(spezifisches Beispiel)* | D | Interviewed person (IP) talks about one specific example | Transkript_IP10_060619 [7487:7513]:<br>I had this [a question to aesthetics of genitalia] just today.<br>*(Das [eine Frage zur Ästhetik des Genitalbereichs] hatte ich gerade heute.)* | 7 | 4 |
| | | Most women satisfied *(Meisten Frauen zufrieden)* | I | Experience that most women are satisfied with the aesthetics of their genitalia | Transkript_IP04_100519 [11612:11661]:<br>I think most of the women are very satisfied [with their genitalia].<br>*(Ich denke die meisten Frauen sind sehr zufrieden [mit ihren Genitalien].)* | 3 | 2 |
| RQ3 | Ask about satisfaction with aesthetics of genitalia<br><br>*(Nachfragen über Zufriedenheit mit Aussehen des Genital)* | Do not ask *(Fragen nicht nach)* | D | IP does not actively ask patients if they are satisfied with aesthetics of their genitalia. | Transkript_IP02_080519 [18868:18968]:<br>No. Because I don't want to bring her [the patients] attention to that (…))<br>*(Nein. Weil ich sie [die Patientin] gar nicht auf, darauf aufmerksam machen möchte (...))* | 7 | 7 |
| | | Ask medically *(Fragen medizinisch)* | D | IP asks patients if there are satisfied with the aesthetics of their genitalia only if there is a medical reason for it. | Transkript_IP06_160519 [24990:25233]:<br>Except of course I see a change in the genital area that indeed is illness-related, (…) then I actively ask her [the patient] for example about sexuality.<br>*(Ausser ich sehe natürlich Veränderungen im Genitalbereich, die tatsächlich krankheitsbedingt sind, (...) da spreche ich [die Patientin] zum Beispiel aktiv die Sexualität an.)* | 3 | 3 |
| | | Ask *(Fragen nach)* | | IP does actively ask patients if they are satisfied with aesthetics of their genitalia. | Transkript_IP04_100519 [12817:13036]:<br>Or are you satisfied with your [the patients] external? Or with your sexual, with the appearance of your sexual organ, like that yes.)<br>*(Oder sind sie zufrieden mit ihrem [der Patientin] äusseren? Oder mit ihrem sexuellen, mit ihrem, äusseren ihres Sexualorgans, so, ja.)* | 1 | 1 |
| | Professional treatments<br><br>*(Professionelles Vorgehen)* | Emphasize normality *(Normalität Betonen)* | D | The normality of the genitalia of patients is emphasized. | Transkript_IP01_060519 [16006:16102]:<br>Then I would maybe also again, like before also already, explain that it [the genitalia] is normal<br>*(Dann würde ich, vielleicht ich auch wieder, wie vorhin auch schon, erklären dass es [die Genitalien] normal ist.)* | 19 | 9 |
| | | Talk *(Gespräch)* | I | Have a talk about issues with patient or encourage patient to talk about it with others. | Transkript_IP03_090519 [14714:14853]:<br>(…) well my experience is that the most [of the patients] with which one really also talks about it [issues with genital appearance] and looks at it actually can deal with it.<br>*((...) also meine Erfahrung ist, dass die meisten [Patientinnen] mit denen man das [Unzufriedenheit mit Aussehen des Genitals] wirklich auch bespricht und anschaut eigentlich damit umgehen können.)* | 22 | 8 |

| | | | | | |
|---|---|---|---|---|---|
| Diversity<br>*(Vielfalt)* | D | Diversity and variations of female genitalia are talked about. | Transkript_IP02_080519 [24157:24316]:<br>I (…) then really emphasize that the normal [genital] has many facet's and so forth and.<br>*(Ich (...) betone dann wirklich, dass das normale [genital] viele Facetten hat und so weiter und.)* | 11 | 8 |
| Transfer to surgery<br>*(Überweisung an Chirurgie)* | D | | Transkript_IP08_040619 [14942:15004]:<br>(…) if a women really insists on it then it [the labiaplasty] will be done, (…)<br>((…) wenn eine Frau darauf insistiert, dann wird das [die Labienverkleinerung] schon gemacht, (…)) | 11 | 7 |
| Advise against operation<br>*(Abratung von OP)* | D | IP would advise against an operation. | Transkript_IP02_080519 [17234:17285]:<br>An operation normally does not make it [genital dissatisfaction] better.<br>*(Eine Operation macht das [Unzufriedenheit mit dem Genital] in der Regel nicht besser.)* | 11 | 7 |
| Visual<br>*(Visuell)* | D | Work with visual material. Show patient their own genitalia in a mirror or show them pictures of other genitalia. | Transkript_IP03_090519 [11394:11630]:<br>I then look at it [genitalia] with the young women with a mirror, so that we can look at where the problem is together.<br>*((...) ich schau das [Genital] dann auch mit den jungen Frauen mit dem Spiegel an, dass wir zusammen schauen wo ist das Problem, was stört.)* | 8 | 7 |
| Individuality<br>*(Individualität)* | I | Treatments are different for each individual. | Transkript_IP01_060519 [12070:12143]:<br>I think that is of course always a little individual who is sitting in front of me.<br>*(Ich glaub ist natürlich immer ein bisschen individuell wer vor mir sitzt.)* | 6 | 6 |
| Inclusion<br>*(Miteinbeziehen)* | I | Patients are included in whole process. | Transkript_IP01_060519 [12594:12628]:<br>But I would actually try to include the woman like that (…)<br>*(Aber ich würde eigentlich probieren so die Frau einzubinden (...))* | 8 | 6 |
| Education on operations<br>*(Aufklärung OP)* | I | Patients are educated specifically on operations and possible complications. | Transkript_IP09_060619 [18192:18217]:<br>(…) tell them my worries [about operations of genitalia]<br>*((...) sag ihnen meine Bedenken [über Operationen des Genitalbereichs].)* | 11 | 6 |
| Take advantage of expert status<br>*(Expertise nutzen)* | I | IP take advantage of their expert status to be able to convince patients. | Transkript_IP07_240519 [28911:29093]:<br>And let the authority work like that.<br>*(Und die Autorität so wirken lassen.)* | 9 | 6 |
| Take seriousely<br>*(Ernstnehmen)* | I | | Transkript_IP02080519 [16563:16621]:<br>Then I do try, I exactly don't say [to the patient]: 'you are crazy'.<br>*(Das versuch ich schon, ich sag eben nicht [der Patientin]: ‚Sie spinnen'.)* | 6 | 6 |

| | | | | | |
|---|---|---|---|---|---|
| Examination *(Untersuchung)* | I | Patients are examined medically. | Transkript_IP04_100519 [15299:15341]:<br>And, ehm, then I think [I do] an examination.<br>*(Und, ähm, dann denk ich [mache ich] eine Untersuchung.)* | 7 | 5 |
| Anamnesis *(Anamnese)* | I | | Tranksript_IP05_160519 [11557:11587]:<br>(…) look at the life situation [of the patient].<br>*((…) die Lebenssituation [der Patientin] anschauen.)* | 10 | 5 |
| Education *(Aufklärung)* | D | Patients are educated on female genitalia. | Transkript_IP09_060619 [20049:20146]:<br>(…) with education and explanation and calming I have much more success [with symptoms of BDD of genitalia] than with the other example [BDD].<br>*((…) hab ich mit Aufklärung und Erklärung und Beruhigung viel mehr Erfolg [bei Symptomen von KDS des Genitalbereichs] als beim anderen Beispiel [KDS].)* | 11 | 4 |
| Transfer to psychotherapy *(Überweisung Psychotherapie)* | D | IP would suggest psychotherapy in some cases. | New Transkript_IP01_060519 [14659:14825]:<br>(…) that first [before an operation] a therapy takes place. Psychotherapy or yes.<br>*((…) dass zu erst [vor einer Operation] eine Therapie statt findet. Psychotherapie, oder ja.)* | 5 | 4 |
| Collaboration *(Zusammenarbeit)* | I | Collaboration with other specialists (e.g. a psychologist) | Transkript_IP06_160519 [32576:33041]:<br>And I think that in this area [BDD] because it is harder, that interdisciplinary collaborations probably have more need for information.<br>*(Und ich glaube, dass in dem Bereich [KDS] dadurch, dass es schwieriger ist, das interdisziplinäre Zusammenarbeiten wahrscheinlich mehr Informationsbedarf ist.)* | 6 | 3 |
| Make little of *(Wenig eingehen)* | I | Not give the problem too much attention. | Transkript_IP07_240519 [23991:24371]:<br>'No you are normal' [I tell the patient] and finished. 'Please accept that'. Here I also don't offer any psychological consultation.<br>*('Nein, sie sind normal' [sage ich der Patientin] und fertig. 'Akzeptieren Sie das, bitte'. Da biete ich auch keine psychologische Beratung an.)* | 3 | 3 |
| Leave decision up to them *(Entscheidung überlassen)* | I | The decision of an operation is left up to the patient. | Transkript_IP09_060619 [18848:18966]:<br>There I can take myself out [of the decision to undergo surgery].<br>*(Da kann ich mich herausnehmen [aus der Entscheidung eine Operation zu machen])* | 2 | 2 |
| Strategies *(Strategien)* | I | IP would try to teach patient strategies to handle their difficulties. | Transkript_IP04_100519 [17475:17700]:<br>(…) maybe there are some techniques to get changed, so that she [the patient] still can for example go excersize or go swimming. | 2 | 2 |

| | | | | | | |
|---|---|---|---|---|---|---|
| | | | | | *((…) vielleicht gibt es gewisse Techniken sich umziehen, damit sie [die Patientin] trotzdem zum Beispiel zum Sport oder zum Schwimmen gehen kann.)* | | |
| | | Expose *(Exponieren)* | I | IP would try to expose patient to their problems to be able to handle them better. | Transkript_IP04_100519 [17366:17460]: But I would see how can she [the patient] maybe still slowly expose herself a little. *(Aber ich würde schauen wie kann sie [die Patientin] vielleicht, doch langsam sich ein bisschen exponieren.)* | 1 | 1 |
| | | Drugs *(Medikamente)* | D | IP mentioned medication as possible treatment. | Transkript_IP11_280619 [22281:22483]: Yesyes. One here couldn't just give drugs. *(Jaja. Man könnte da nicht einfach Medikamente abgeben.)* | 1 | 1 |
| | | Screening for BDD *(Screening auf KDS)* | D | | None | 0 | 0 |
| RQ4 | Interest in further education<br><br>*(Interesse an Fortbildungen)* | Large interest in further education *(Grosses Interesse an Fortbildung)* | D | | Transkript_IP07_240519 [25919:25933]: Well rather large [the interest in further education]. *(Als eher gross [das Interesse an Fortbildung].)* | 9 | 7 |
| | | Medium interest in further education *(Mittel Interesse an Fortbildung)* | D | | Transkript_IP01_060519 [17305:17438]: I think that there is some interest [in further education] around, but probably not in the width… *(Ich denk dass schon Interesse [an Fortbildung] da ist, aber wahrscheinlich nicht in der Breiten…)* | 4 | 4 |
| | | Little interest in further education *(Wenig Interesse an Fortbildung)* | D | | None | 0 | 0 |
| | | No interest in further education *(Kein Interesse an Fortbildung)* | D | | None | 0 | 0 |
| | | Under educated *(Untergebildet)* | I | Gynecologists are not well educated in psychological topics. | Transkript_IP08_040619 [18081:18153]: Often because there is also an undersupply in the further education [of gynecologists], right. *(Häufig da auch eine Unterversorgung in der Weiterbildung [der Gynäkologen und Gynäkologinnen] besteht, oder.)* | 3 | 2 |
| | | Practices *(Niedergelassene)* | I | Gynecologists in settled practices would be more interested in further education than those in clinics. | Transkript_IP01_060519 [17316:17415]: (…) in the outpatient, well in the practices, the gynecologists that they there could have more interest [in further education]. | 2 | 2 |

| | | | | | | |
|---|---|---|---|---|---|---|
| | | | | *((...) in den ambulanten, also in den Praxen, die Gynäkologinnen dass die da mehr Interesse haben könnten [an Fortbildungen].)* | | |
| Interest in diagnostic instruments of BDD<br><br>*(Interesse an Instrumente der Diagnostik der KDS)* | Large interest in diagnostics *(Grosses Interesse an Diagnostik)* | D | | Transkript_IP06_160519 [33808:33848]:<br>I think that that [diagnostic instruments] are very helpful (…)<br>*(Ich glaube, dass es [diagnostische Instrumente] sehr hilfreich ist (...))* | 4 | 4 |
| | Medium interest in diagnostics *(Mittel Interesse an Diagnostik)* | D | | Transkript_IP07_240519 [28482:28580]:<br>Yes, that [diagnostic instruments] can be helpful in the sense that it defines normality better for the younger [doctors].<br>*(Ja, das [diagnostische Instrumente] kann in dem Sinne hilfreich sein, dass es Jüngeren [Ärzten und Ärztinnen] die Normalität besser definieren kann.)* | 2 | 2 |
| | Little interest in diagnostics *(Wenig Interesse an Diagnostik)* | D | | Transkript_IP09_060619 [23847:23988]:<br>Ehm, well honestly I would probably read it [the diagnostic instrument], put in a file and think I have it and then never think of it again.<br>*(Ähm, also ehrlich gesagt, würde ichs [das diagnostische Instrument] wahrscheinlich durchlesen, in ein Fach tun und denken ich hab das und nachher nicht mehr daran denken.)* | 4 | 4 |
| | No interest in diagnostics *(Kein Interesse an Diagnostik)* | D | | Transkript_IP02_080519 [26288:26313]:<br>Here [with diagnostic instruments] I am very skeptical.<br>*(Da [bei diagnostischen Instrumenten] bin ich sehr skeptisch.)* | 1 | 1 |
| | Standardize *(Objektivieren)* | I | Diagnostic material is interesting because it can objectify and standardize the symptoms. | Transkript_IP08_040619 [18350:18456]:<br>That [diagnostic instrument] is surely ah, that is surely very interesting, because it also helps, or standardizes, right.<br>*(Das [diagnostische instrument] ist sicher äh, das ist sicher sehr interessant, weil das erleichterts auch, oder standardisierts oder.)* | 3 | 3 |
| | Awareness *(Bewusstsein)* | I | Diagnostic material can help raise awareness of issue. | Transkript_IP10_060619 [14849:14913]:<br>(…) that one [with support of the diagnostic instrument] simply doesn't forget to ask about some aspect.<br>*((...) dass man [mit Hilfe des diagnostischen Instruments] einfach nicht vergisst, irgendeinen Aspekt zu erfragen.)* | 3 | 3 |
| | Unpractical *(Unpraktisch)* | I | Diagnostic material is unpractical in daily work of gynecologist. | Transkript_IP06_160519 [33845:34865]:<br>(…) the problem is that mostly that one needs a certain amount of time so that one can use them [the diagnostic instruments], well one has to deal with it and that is always a question of lack of time. | 3 | 3 |

| | | | | | | |
|---|---|---|---|---|---|---|
| | | | | *((...) das Problem ist meistens, dass man 'ne gewisse Zeit braucht, damit man die [diagnostischen instrumente] benutzen kann, also man muss sich damit beschäftigen und das ist immer 'ne Frage des Zeitmangels.)* | | |
| | Practical *(Praktisch)* | I | Diagnostic material is practical in daily work of gynecologist. | Transkript_IP08_040619 [18458:18743]: Any underassistant can be ordered to, or to find it out [with the diagnostic instrument]. That is surely good. *((...) kann jeder Unterassistent beauftragt werden, oder, das herauszufinden [mit dem diagnostischen Instrument]. Das ist sicher gut.)* | 3 | 3 |
| | Unfitting *(Unpassend)* | I | Diagnostic material is not perceived as fitting for a diagnosis. | Transkript_IP02_080519 [26427:26553]: I think the reality is so often more complex [than portrayed in an diagnostic instrument]. *(Ich glaube die Wirklichkeit ist so häufig viel komplexer [als im diagnostischen Hilfsmittel dargestellt].)* | 3 | 2 |
| | Unnecessary *(Unnötig)* | I | Diagnostic material is unnecessary. | Tranksript_IP05_160519 [14363:14582]: I don't think, that the [diagnostic instruments] not, that I need that because one notices yes, that, one sees the congruence. *(Ich glaube nicht, dass nicht das [diagnostische Instrumente], dass ich das brauche weil man merkt ja, dass, man sieht ja die Kongruenz.)* | 1 | 1 |
| Interest in support for consultation on BDD of genitalia *(Interesse an Hilfsmittel zur Beratung der KDS des Genitalbereichs)* | Large interest in support *(Grosses Interesse an Hilfsmittel)* | D | | Transkript_IP02_080519 [29002:29036]: Well good boards [as support for consultation] large interest (…). *(Also gute Tafeln [als Hilfsmittel für die Beratung] grosses Interesse (...).)* | 7 | 7 |
| | Medium interest in support *(Mittel Interesse an Hilfsmittel)* | D | | Tranksript_IP05_160519 [15136:15167]: Well it [supportive material for consultation] is definitely interesting *(Also [Hilfsmittel für Beratung] ist definitiv interessant.)* | 2 | 2 |
| | Little interest in support *(Wenig Interesse an Hilfsmittel)* | D | | None | 0 | 0 |
| | No interest in support *(Kein Interesse an Hilfsmittel)* | D | | Transkript_IP04_100519 [20538:20759]: Don't we already have too many pictures [of genitalia]? ((laughs)). *(Haben wir nicht zu viel Bilder [von Genitalien] schon? ((lacht)).)* | 1 | 1 |

| | | | | | |
|---|---|---|---|---|---|
| Depending on format *(Abhängig von Form)* | I | Depending on what kind of support material is presented it could be more or less interesting. | Transkript_IP02_080519 [28120:28160]:<br>Yes one now would have to look at the boards [as support for consultation].<br>*(Ja müsste man jetzt die Tafeln [als Hilfsmittel für die Beratung] anschauen.)* | 1 | 1 |
| Information to hand out *(Info zum Mitgeben)* | I | | Transkript_IP06_160519 [35714:36379]:<br>Well I think that it often helps in a conversation when one also has some kind of guidelines to hand out to the patient.<br>*(Also ich glaube, das hilft häufig im Gespräch, wenn man eben auch den Patienten irgendwelche Leitfäden auch aushändigen kann.)* | 1 | 1 |
| Already in usage (Bereits in Benutzung) | | IP already had supportive materials for consultation of women with issues with the aesthetics of their genitalia. | Transkript_IP08_040619 [15178:15388]:<br>We have a very interesting, ahm, plaster- ah, a plaster poster. There plaster casts of, ahm, female genitalia were made.<br>(Wir haben eine ganz interessante, äh, Gips-,äh, ein Gipsposter. Da wurden Gipsabdrücke von ähm, weiblichen Genitalien gemacht.) | 1 | 1 |

*Note***.** I stands for inductively found categories, D stands for deductively found categories. All categories and example quotes were translated from German to English by the author. In () and *italic* are the German original terms and sentences. In [] are comments by the author for clarification of the quotes.

# Appendix G: Transcripts

Interview mit: IP1
Datum des Interviews: 06.05.2019
Dauer: 25 min
Anonymisierte Begriffe: keine
IP- Nummer: 1

MS: Vielen Dank haben Sie sich Zeit genommen für das Interview.

IP1: Gerne.

MS: Ähm. Dann beginn ich mal mit der ersten Frage und zwar: Mit welchen psychischen Stö-rungen kommen denn Frauen zu Ihnen in die Klinik, oder was haben Sie schon erlebt?

IP1: Ähm, meinen Sie mit der Frage, dass die psychische Störung dazu kommt, dass sie zu uns kommt oder als Begleit- also Nebendings.

MS: Beides, also was bekommen Sie mit, also sagen Ihnen die Patientinnen das oder merken Sie das bei Ihren Patientinnen, einfach was Sie schon gesehen haben.

IP1: Mh, also es gibt ganz unterschiedliche Krankheitsbilder in Bezug auf Gynäkologie tatsächlich. |**<< Somatoforme Störungen (F45)**|Sind halt häufig psychosomatische Beschwerden, wie zum Beispiel Missempfindungen im Genitalbereich also Juckreiz vor allem am Genital oder ähm |**<< Sexuelle Funktionsstörungen, nicht verursacht durch eine organische Störung oder Krankheit (F52)**|Schmerzen beim Geschlechtsverkehr, das sind so die somatischen Probleme|*Sexuelle Funktionsstörungen, nicht verursacht durch eine organische Störung oder Krankheit (F52) >>*|.|*Somatoforme Störungen (F45) >>*| Und ansonsten gibt's natürlich in der Geburtshilfe auch immer wieder Patientinnen. Also jede Frau kann, also gebärt und dann gibt's natürlich Patientinnen mit psychiatrischen Diagnosen, zum Beispiel |**<< Phobische Störung (F40) und andere Angststörungen (F41)**|Angststörungen|*Phobische Störung (F40) und andere Angststörungen (F41) >>*|, |**<< Depressive Episode (F32) und rezidivierende depressive Störung (F33)**|Depressionen|*Depressive Episode (F32) und rezidivierende depressive Störung (F33) >>*|, ähm |**<< Schizophrenie (F20)**|oder auch schon Schizophrenie |*Schizophrenie (F20) >>*|oder andere Erkrankungen. |**<< Psychische oder Verhaltensstörungen im Wochenbett, anderenorts nicht klassifiziert (F53)**|Und was dann natürlich noch dazu kommt im Wochenbett: Wochenbettdepression oder auch dann im Wochenbett sich eine Psychose entwickelt.|*Psychische oder Verhaltensstörungen im Wochenbett, anderenorts nicht klassifiziert (F53) >>*| Das hab' ich jetzt schon so erlebt.

MS: Mhm, Okay. Gut. Ähm. Was würden Sie sagen welche Rolle spielt dann die Zufriedenheit mit dem äusseren Erscheinungsbild, ähm bei Ihren Patientinnen?

IP1: Darüber hab' ich mich schon Gedanken gemacht bevor, und da muss ich sagen so als rein, also fast ausschliesslich somatische, also ich als Ärztin behandle ja vor allem das somatische Leiden oder das, ähm, ja. Und da hab' ich mich irgendwie bisher nie darauf geachtet ob das äussere Erscheinungsbild irgendwie 'nen Zusammenhang mit dem könnte, oder auch das, also ich muss ehrlich sagen, dass ich da jetzt nie auf die Zufriedenheit vom äusseren Erscheinungsbild irgendwie ein Kontakt geknüpft hab oder dass ich die Frauen gefragt hab' und so-gar ehrlich gesagt denk ich jetzt gerade: ganz häufig Frauen mit Angststörungen oder Depres-sion oder so würde ich nie im Leben denken, wenn ich sie seh', vom Erscheinungsbild, dass die Frauen 'ne psychiatrische Erkrankung haben. Das klingt fast so stigmatisierend, aber also, mach schnell 'ne Pause.

[kurze Pause im Interview]

MS: Also das heisst, es kommen auch keine Patientinnen zu Ihnen und fragen, und ähm, äussern etwas über ihr Aussehen oder so? Haben Sie das jetzt noch nicht erlebt?

IP1: Ich hab' das noch nicht erlebt. Aber dazu muss man sagen: Ich kann mir vorstellen, dass ich als Spitalärztin, hab ja nicht so meine Patientinnen. Sondern es sind meistens Einzelkon-takte: Die kommen als Notfall zu mir, oder sie sind einmal für 'ne Operation da, oder sie sind einmal für 'ne Geburt da und ich kann mir vorstellen, dass solche Problematiken eher in der Praxis auftauchen, wo die Ärztin die Bezugsperson ist und die Frauen regelmässig zu ihr kommen.

MS: Okay.

IP1: Ist ja viel anonymer bei uns sozusagen.

MS: Guter Punkt, mhm. Okay. Gut. Ähm. Dann kommen wir jetzt zu einer Fallvignette, die ich Ihnen gerne zeigen würde.

IP1: Mhm.

MS: Ich bitte Sie einfach die durchzulesen. Und dann hab' ich eine Frage dazu.

[MS legt Fallvignette 1 hin. IP1 liest Fallvignette 1 durch.]

IP1: Mhm. Okay.

MS: Mhm. Ähm. Jetzt die Frage dazu ist: Was denken Sie, was für eine psychische Störung, Auffälligkeit, kommt Ihnen hierzu in den Sinn?

IP1: (2). Also muss ich jetzt ein ICD 10 -

MS: Nein, nein also einfach, keine Kodierung was auch immer. Einfach ähm -

IP1: Ja.

MS: Einfach Ihre Gedanken dazu, Sie können mal laut überlegen.

IP1:|**<< Körperwahrnehmungsstörung**| Hmm. Ja also, es ist vielleicht eine, wie heisst das, Körperwahrnehmungsstörung,|*Körperwahrnehmungsstörung >>*| oder so was. |**<< vermindertes Selbstwertgefühl und Selbstvertrauen**|Und ähm, auf jeden Fall fehlendes Selbstvertrauen.|*vermindertes Selbstwertgefühl und Selbstvertrauen >>*| Und, ähm, ist natürlich die Frage. Mit den Informationen kann ich natürlich nicht sagen was dahinter steckt, |**<< Depressive Episode (F32) und rezidivierende depressive Störung (F33)**|könnte sicher irgendwas, depressives, oder (1). |**<< Begründung**|Ich war schon, bin schon bissl raus aus der Sache. ((lacht)).|*Begründung >>*| Depressiv oder ja, alles, alles, kann noch viele andere Sachen.|*Depressive Episode (F32) und rezidivierende depressive Störung (F33) >>*|

MS: Wenn Sie mal zeigen können, worauf basieren Sie ihre Annahmen. Oder was waren die ausschlaggebenden Satzteile, die Sie jetzt angesprochen haben?

IP1: Ja einfach, dass sozusagen die Aussenwelt nimmt sie anders wahr als sie selbst, und dass sie jede Sekunde daran denkt, also dass es sozusagen ein mächtiges, was Übermächtiges ist und sie nicht da raus kommt irgendwie und dass es auch ihr Sozialleben beeinträchtigt. Also die Lebensqualität leidet darunter, sie meidet soziale Aktivitäten. So.

MS: Mhm, und wenn jetzt das eine Patientin wäre von Ihnen, die so zu Ihnen kommt. Was würden Sie denn noch für weitere Informationen jetzt von Ihr brauchen?

IP1: (1). Ich würde auf jeden Fall, mich würde noch mehr so die soziale Anamnese interessie-ren also, so ähm ob sie überhaupt ihr Haus verlässt. Ob sie ähm, eine Arbeitsstelle hat. Ob sie sich da wohl fühlt. Ähm. Dann so die, bei einer Frau in dem Alter die so, würde ich auch im-mer noch an Ess- an Essverhalten oder so was, würde mich noch interessieren. Und dann ich mein natürlich, wenn man dann noch tiefer gehen möchte ist dann die Frage woher kommt es und ähm aus was für 'nem Haus kommt sie, was hat sie erlebt in der Kindheit, oder hatte sie irgendwelche traumatische Erlebnisse. Ja. Dann würde ich sie natürlich noch gut anschauen ob da irgendwelche, ob's vielleicht irgendwelche Narben oder irgendwas gibt die auf irgendwelche anderen ähm Störungen hinweisen könnten.

MS: Mhm. Ok. Ähm. Ich hab' eine Auflösung.

IP1: ((lacht)) Oh mein Gott.

[MS legt die DSM 5-Diagnosekriterien der KDS hin.]

MS: Also ähm, genau. Sie haben gesagt Körperwahrnehmungsstörung das ist eigentlich gar nicht so weit davon entfernt. Was wir hier darstellen wollten ist eine Körperdysmorphe Störung.

IP1: Mhm.

MS: Ähm, ich habe hier die DSM-5. Sie arbeiten ja mit dem ICD-10 in der Klinik?

IP1: Mhm.

MS: Genau. Im DSM-5 ist ein bisschen schöner dargestellt, was die Symptome sind. [zeigt DSM-5 Kriterien]. Und zwar haben diese Patientinnen und Patienten eine übermässige Beschäftigung mit einem Mangel oder Defekt an ihrem Körper der eben für andere nicht erkennbar oder nur geringfügig ist. Ähm. Dann haben sie eben diese wiederholenden Verhaltensweisen, wie sie überprüfen sich ständig im Spiegel, übermässige Körperpflege, Rückversicherungsverhalten bei ihren Freunden. Genau. Ist halt mit einem hohen Leidensdruck ver-bunden. Sozial, beruflich aber auch in anderen wichtigen Funktionsbereichen. Und, das was Sie eben fragen würden, es ist eben keine Essstörung. Also es ist eine andere Störung. Weil dort beschäftigen sie sich ja meistens mit Gewicht und Körperfett. Und bei dieser Störung geht es wirklich um Körperteile also Gesicht, Brüste und so weiter. Und einfach noch als In-formation, weil Sie eben mit dem ICD10 arbeiten [zeigt ICD10 Kriterien]. Da ist es bei den Hypochondrischen Störungen klassifiziert. Als Dysmorphophobie. Aber das bekommen Sie nachher noch, wenn es Sie weiter interessiert. Genau. Haben Sie noch eine Frage zu dem, oder war das verständlich?

IP1: Mhm.

MS: Genau. Jetzt wissen Sie schon ein bisschen um was es geht. Ähm. Damit kommen wir zur nächsten Frage und zwar: Wenn Sie jetzt dieses Fallbeispiel gesehen haben, haben Sie denn schon irgendwelche Erfahrungen mit Patientinnen gemacht, die etwas ähnliches berich-tet haben?

IP1: (2). Also schwierig zu sagen. Mir kommt jetzt gerade nur eine Situation in den Sinn. Da war ich selbst noch Studentin. Und da war ich bei einer Spezialsprechstunde für eigentlich, eigentlich war es so was wie genitale Fehlbildungen. |**<< Labien erwähnt**|Und da kam 'ne

Patientin, und sie hatte halt, ähm, ihrer Ansicht nach waren ihre inneren Labien zu gross.|*Labien erwähnt >>*| Aber für uns Untersuche-rinnen waren das nicht der Fall. Sie war total verunsichert und wollte die verkleinert haben. Dass ist jetzt vielleicht eine Situation, die in die Richtung gehen könnte. Ähm und ansonsten fällt mir jetzt ehrlich gesagt nichts ein.

MS: Mhm. Okay. Die nächste Frage wäre gewesen auf welche Herausforderungen treffen Sie, wenn Sie in Kontakt sind mit solchen Patientinnen. Also wenn wir vielleicht das Beispiel nehmen, also was war dort schwierig?

IP1: Ja in dem Fall wars eigentlich schwierig, weil wir beide Untersucherinnen die Intervention oder Operation nicht für Sinnvoll gehalten haben, die sie sich gewünscht hat. Und zwar nicht auch, es ist nicht nur nicht sinnvoll, sondern es ist auch, also immer mit 'nem Risiko verbunden eine Operation zu machen. Und sowieso Operationen im Genitalbereich, weiss man auch nicht sicher was es zur Folge hat was die Sensibilität oder so danach angeht. Ich hab' da jetzt nicht grosse Erfahrungen aber einfach so, wenn man sich das so logisch überlegt. Und, ja eigentlich war auch schon eine Herausforderung, sozusagen die Frau von ihrer Über-zeugung herunter zu holen, oder ja das bisschen zu nehmen das Gefühl, dass da etwas nicht gut ist, oder dass es zu gross ist, was ja nicht, genau. Ich weiss auch ehrlich gesagt nicht wie's dann mit ihr weiter ging, weil ich war eben nur Studentin und war nur an dem Tag da.

MS: Mhm. Okay. Gut. Die nächste Frage ist dann eben wie sind Sie da vorgegangen mit dieser Patientin, also was haben Sie, einfach gesagt es ist alles okay, oder haben Sie's ihr noch irgendwie gezeigt oder wie war da die Interaktion?

IP1: Ich war ja Beobachterin. Und ähm die Ärztin hat ihr gesagt, dass es, dass es normal ist und dass sie ganz viel Vergleiche hat, viel mehr als die Patientin selber wahrscheinlich. Ähm und dass sie sich, dass es völlig normal ist, ähm, und dann wars eigentlich auch noch eine Interessante Situation, weil die Patientin dann gesagt hat: «Ah, sieht's bei Ihnen genauso aus?» ((lacht)) Ähm, dann ist die Ärztin selber bisschen in Stress gekommen. Und ich glaub dann hat sie auch noch, sie ist dann nicht so richtig drauf eingegangen und dann hat sie auch noch ein Spiegel geholt, glaub ich. Und dann haben sie sich's auch noch mal gemeinsam an-gekuckt. Aber ganz genau kann ich mich, also was dann konkret noch war, weiss ich jetzt auch nicht mehr genau.

MS: Aber dann auch eher abgeraten von -

IP1: Ja.

MS: Der OP.

IP1: Jaja, auf jeden Fall.

MS: Ähm, ja, diese Frage muss ich dann halt hypothetisch stellen. Also wenn Sie sich jetzt vorstellen würden, dass so eine Patientin zu Ihnen kommt. Jetzt mit dem kleinen Hintergrundwissen, dass Sie jetzt haben zu dieser Störung, wie würden Sie da vorgehen, wenn jetzt eine Patientin diese Beschwerden äussert?

IP1: Also ich denke, dass ich auf jeden Fall medizinische Interventionen, ähm, verhindern, oder nicht einwilligen würde. Zumindest nicht ohne Gutachten, oder ohne dass die Patientin mit einem Psychologen oder Psychologin in Kontakt war. Und es ist natürlich vielleicht nicht so ganz einfach die Patientin davon zu überzeugen, ähm, dass es vielleicht sinnvoll wäre sich vielleicht psychologische Unterstützung zu holen. Ja. Aber ich wäre sehr zurückhaltend eben mit medizinischen Interventionen und würde versuchen die Frau da in eine andere Richtung zu lenken.

MS: Mhm. Glaub ist schon fertig.

OD: Gut dann übernehm ich jetzt. Ähm. Ja meine Fragen wurden ein bisschen vorgegriffen.

IP1: Okay.

OD: Also es ist kein Problem. Ähm aber eben Sie haben ja das Beispiel genannt über die Frau die eben unzufrieden war mit der Ästhetik ihres Genitalbereichs. Ähm. Haben Sie sonst noch Erfahrungen, also stellen Frauen manchmal Fragen vielleicht ob es normal aussieht oder sonst zur Ästhetik ihres Genitalbereichs?

IP1: (2). |**<< Seltene Fragen** |Selten. Also wirklich selten, dass es thematisiert wird.|*Seltene Fragen >>*| |**<< Vertrauensperson**|Bei mir, glaub ich. Was aber auch wieder daran liegen könnte, dass ich ‘ne fremde Person bin. Wenn die Frauen all Halbjahr oder einmal im Jahr zu mir kommen würden, könnte sich das ändern. Denke ich.

|*Vertrauensperson >>*|OD: Ja. Und ja wie, wenn das vorkommt wie gehen Sie dann vor oder, Sie haben ja schon ein bisschen gesagt aber, hätten Sie noch irgendwas hinzu zu fügen oder würden Sie das immer so machen?

IP1: |**<< Individualität**|Ich glaub ist natürlich immer ein bisschen individuell wer vor mir sitzt.|*Individualität >>*| |**<< Vielfalt**|Aber ich glaub, wenn eine Frau über ihr Genital Unsicherheiten hätte, würde ich wahrscheinlich so was erzählen wie: dass es eine grosse Variation an weiblichen Genitalen gibt|*Vielfalt >>*| und |**<< Expertise nutzen**|dass ich ganz viele gesehen, also so Vergleiche hab.|*Expertise nutzen >>*| |**<< Normalität betonen**|Und dass, dass es einfach so normal ist.|*Normalität betonen >>*| |**<< Medizinische Beschwerden**|Also ich mein gibt natürlich sicher auch Dinge die jetzt nicht normal sind|*Medizinische Beschwerden >>*| aber, also normal, was ist normal? Ich will mich nicht unkorrekt ausdrücken. |**<< Miteinbeziehen**|Aber ich würde eigentlich probieren so die Frau einzubinden |*Miteinbeziehen >>*|und zu |**<< Normalität betonen**|sagen dass es einfach so, so die Natur ist und wir auch, ja.

|*Normalität betonen >>*|OD: Ähm, und fragen Sie auch manchmal bei Ihren Patientinnen nach wie sie, wie sie die Ästhetik ihres Genitalbereichs finden?

IP1: |**<< Fragen nicht nach**|Nein.|*Fragen nicht nach >>*|

OD: Ja die nächste Frage haben wir eigentlich auch schon ein bisschen besprochen. Es geht, also haben Sie sonst noch Erfahrungen, ausser dieses Beispiel, mit Patientinnen die Schön-heitsoperationen am weiblichen Genitalbereich wollten?

IP1: Gehört die Brust auch dazu? Ja oder?

OD: Ähm.

IP1: Oder nein? Oder?

OD: Eigentlich nicht, aber wenn Sie erzählen wollen dann gerne.

IP1: (1) |**<< Seltene OP**|**<< Labien**|Also ich hab’ das natürlich schon mitgekriegt, dass Frauen sich die Labien verklei-nern lassen haben|*Labien >>*|, ähm aber meistens sind die dann in den Sprechstunden bei Oberärzten oder leitenden Ärzten davor. Deshalb ist es dann eher so, dass ich tatsächlich bei der Operation oder so, dass dann eher mitkriege. Aber im Gespräch war ich dann nicht dabei.|*Seltene OP >>*| |**<< Brüste**|Und ich mein die Brüste sind ein anderes Thema, also, wenn man sich da umkuckt wer da alles Narben an den Brüsten hat, dann, dann ist es ein weit verbreitetes Ding. |**<< Medizinische Beschwerden**|Wobei da

natürlich da nicht nur die Ästhetik im Vordergrund steht, sondern da gibt's ja auch körperliche Beschwerden wenn die Brüste gross sind oder so. |*Brüste >>*|*Medizinische Beschwerden >>*|

OD: Mhm. Ja die nächste Frage haben wir eigentlich auch schon gehabt ((lacht)). Was Ihr Vorgehen da wäre, also ja haben Sie eigentlich schon ein bisschen gesagt aber?

IP1: Ich denk generell ist es immer so, wenn das in der Medizin. Jetzt red ich aber nicht ganz persönlich von mir, |**<< Medizinische Beschwerden**|wenn jetzt irgendwelche körperlichen Beschwerden vorhanden sind, |**<< Brüste**|aufgrund von irgend-, also zum Beispiel grosse Brüste und Rückenschmerzen.|*Brüste >>*| |**<< Labien**|Oder manchmal gibt's den Fall, dass die Labien so gross sind, dass es Beschwerden |*Labien >>*|macht beim Sitzen oder Fahrradfahren oder so, dann ist es immer einfacher Interventionen zu planen oder Einverständnis zu geben.|*Medizinische Beschwerden >>*| |**<< Überweisung Psychotherapie**|Wenn es jetzt rein, psychisches Leiden ist, dann wie ich vorhin gesagt hab ist auf jeden Fall wichtig, dass zuerst eine Therapie stattfindet. Psychotherapie, oder ja.

|*Überweisung Psychotherapie >>*|OD: Ja. Dann haben wir noch mal ein Fallbeispiel. Können Sie das kurz durchlesen.

[OD legt Fallvignette 2 hin. IP1 liest Fallvignette 2 durch.]

IP1: Mhm. Gut.

OD: Das ähm ist ein Beispiel für eine Körperdysmorphe Störung des weiblichen Genitalbereichs. Also es kann eigentlich jedes Körperteil betroffen sein von der Körperdysmorphen Störung und das wär' ein Beispiel für den Genitalbereich. Hatten Sie schon Erfahrungen mit solchen Patientinnen, also die vielleicht eben nicht unbedingt direkt eine Operation wollen aber einfach solche Beschwerden äussern?

IP1: |**<< Seltene KDS**|Ja eben eigentlich |**<< spezifisches Beispiel**|vor allem das eine Beispiel über das wir jetzt schon gesprochen haben.|*spezifisches Beispiel >>*| Und sonst, bin ich nicht so in Kontakt gekommen. |**<< Subklinische Unzufriedenheit**|Oder vielleicht kann sein, dass mal eine Frau was sagt, aber das ist dann mehr so, nicht dass es explizit, dass es das grosse Anliegen war. Tut mir leid. |*Seltene KDS >>*|*Subklinische Unzufriedenheit >>*|

OD: ((lacht)) Muss Ihnen nicht leid tun. Ist überhaupt kein Problem. Ähm und wenn jetzt die-se [zeigt auf Fallvignette] Patientin zu Ihnen kommen würde, wie würden Sie dann vorgehen?

IP1: (1). |**<< Anamnese**|Puh. Ja ich denk ich würde sie noch ein bisschen genau befragen. Also was, wie sie, also woher das überhaupt kommt die Idee, dass es nicht normal wär'.|*Anamnese >>*|**<< Gespräch**| Oder, also ich würde einfach noch mal genau sie befragen, wie sie sich darin fühlt und was genau sie stört und also einfach mehr darüber sprechen.|*Gespräch >>*| |**<< Normalität betonen**|Dann würde ich, vielleicht ich auch wieder, wie vorhin auch schon, erklären, dass es normal ist.|*Normalität betonen >>*| |**<< Fehlende Einsicht**|Aber ich mein das würde wahrscheinlich, das ist wahr-scheinlich, da red' ich wahrscheinlich gegen eine Wand, wenn ich ihr das sag. Wie so, wie alle anderen auch.|*Fehlende Einsicht >>*| Ähm, ja ist jetzt gar nicht so einfach da, darauf zu äh.|**<< Überweisung Psychotherapie**| Und wenn die Patientin zum ersten Mal zu mir kommt ist auch schwierig zu sagen: «Ah, gehen sie mal zum Psychologen». |*Überweisung Psychotherapie >>*|**<< Ernstnehmen**|Weil dann fühlt sie sich ja auch nicht ernst genommen.|*Ernstnehmen >>*| |**<< Visuell**|Dann, keine Ahnung, im Internet kucken ((lacht)), was es für unterschiedliche weibliche Genitalien gibt. Kann man sich Fotos anschauen oder so, also ich mein da gibt's natürlich Plakate und so weiter. |*Visuell >>*|Ähm. Ja. Ich bin jetzt gerade auch ein bisschen ratlos.|**<< Patientin muss selbst an sich arbeiten**| Ich denk hat viel mit Selbstwertgefühl und so weiter zu tun und dass, muss in ihr selbst wachsen und nicht

wo anders.

|*Patientin muss selbst an sich arbeiten >>*|OD: Gut, danke. Ja wir sind eigentlich schon bald am Ende. Es geht jetzt noch ein bisschen so um Fortbildung oder Hilfsmittel. Ähm, wie gross schätzen Sie das Interesse an einer Fortbil-dung in, also zu Körperdysmorphen Störung in der Gynäkologie ein?

IP1:|**<< Mittel Interesse an Fortbildung**| Ich denke es ist Typ abhängig, weil ähm, ob die-. Ich denk, dass schon Interesse da ist, aber wahrscheinlich nicht in der Breiten-, |*Mittel Interesse an Fortbildung >>*|**<< Niedergelassene**|wobei ich kann mir vorstellen, dass eigentlich schon so, vor allem in den ambulanten, also in den Praxen, die Gynäkologinnen, dass die da mehr Interesse haben könnten.|*Niedergelassene >>*| Im Spital ist es halt eher so, da geht's halt mehr so um operieren und, ähm und die neuste Krebsforschung und das und das und das und das. Aber solche The-men kann ich mir vorstellen ist schon Interesse da, eher bei niedergelassenen Gynäkologin-nen. Oder bei Gynäkologinnen die sowieso psychosomatische ähm, Sprechstunden oder ähm irgend so was gibt's ja auch.

OD: Und wie gross schätzen Sie das Interesse und den Nutzen an Hilfsmittel zur Diagnostik der KDS, also wie sie schon gesagt haben eben vielleicht so Tafeln oder so wo man verschie-dene ähm, Abbildungen sieht, oder auch so Screening Instrumente oder so?

IP1: Ob das Interesse da sein könnte?

OD: Ja. An so Hilfsmittel.

IP1: |**<< Grosses Interesse an Diagnostik**|**<< Grosses Interesse an Hilfsmittel**|Ja. Sicher. |*Grosses Interesse an Diagnostik >>*|*Grosses Interesse an Hilfsmittel >>*|

OD: Ok jetzt hab' ich eigentlich beide Fragen in einer gestellt. Gut ja dann sind wir eigentlich schon am Schluss. Möchten Sie noch etwas hinzufügen?

IP1: Nein, vielen Dank für das interessante Gespräch.

OD: Danke Ihnen.

Interview mit: IP2
Datum des InterviewIP2: 08.05.2019
Dauer: 36 min
Anonymisierte Begriffe: keine
IP-Nummer: 2

MS: Dann beginnen wir mit der Einstiegsfrage, und zwar: Mit welchen psychischen Problemen kommen denn Patientinnen zu Ihnen in die Praxis?

IP2: Also ganz grundsätzlich, meinen Sie?

MS: Genau, also grundsätzlich.

IP2: Jung bis alt und schwanger und nicht. Ähm ist natürlich ein sehr breites Feld und ich würde ein bisschen provokativ sagen, wahrscheinlich ein ganz ähnliches Spektrum wie es auch eine Psychologin hätte. Zum Teil aber nicht, ähm, als direkt erste Frage. |**<< Phobische Störung (F40) und andere Angststörungen (F41)**|Also in der Gynäkologie haben wir es sehr häufig mit, ähm, eigentlich Angst zu tun.|*Phobische Störung (F40) und andere Angststörungen (F41) >>*| |**<< Somatoforme Störungen (F45)**|Die sich äussert dann an irgendeinem körperlichen Symptom, an Beschwerden.|*Somatoforme Störungen (F45) >>*| Ähm. Wo man dann relativ bald einmal merkt, es geht gar nicht wirklich um den Schmerz der nicht erträglich ist, sondern es geht um die Angst dahinter, dass etwas nicht gut sein könnte. Also das ist was ganz Häufiges. Aber natürlich auch das alles andere, mit irgendwie weitem Sinne psychosomatischer Formenkreis, wenn man dem so sagen will. Also ich bin jetzt über 20 Jahre Gynäkologin und ich denke je länger je mehr man kann eigentlich was man früher mit Körper und Psyche aufgeteilt hat, das ist völlig absurd. Und deswegen find ich das auch super, so wenn man von den Disziplinen zusammensitzt und ja, schaut wo liegen eigentlich die Probleme. Aber ich kann Ihnen nicht jetzt eine, eine Liste von psychologischen Punkten, eben man könnte noch verschiedene Symptome oder so. Aber ich denke Angst ist schon etwas vom häufigsten.

MS: Mhm. Okay. Welche Rolle spielt denn die Zufriedenheit mit dem äusseren Erscheinungsbild bei Ihren Patientinnen?

IP2: Ähm, es ist halt, auch das generell ganz, ganz schwierig. Klar es ist ein häufiges Thema. Also, und zwar auch wieder in verschiedenen Lebensphasen, verschiedene Themen. Also es beginnt mit den Teenagern, einerseits Haut aber eben dann auch, ja wirklich recht häufig mit dem Intimbereich der nicht Vorstellungen entspricht, wie sie das haben. Ähm, also ich denke eben so Adoleszenz ist ein heikler, äh, Bruch. Für mich sind es vor allem immer die Umbruchsphasen. Der Frauenkörper verändert sich ja mehrere Male, während des Lebens. Und Adoleszenz ist so eine, äh spezielle Periode. Und während der Schwangerschaft dann erneut. Nach der Geburt nochmals. Also das ist dann ganz banal, Bauchumfang, Haut die anders ist, und so weiter. Und ähm, dann natürlich nochmals die Wechseljahre und das Älterwerden. Also, das ist dann auch, Adoleszenz und Wechseljahre. Und das sind dann häufig Mutter und Tochter gleichzeitig, äh. Wo's wirklich schwierig ist. Und die Frauen zum Beispiel kommen und sagen: «Ich hab' das Gefühl es ist ein Hormonproblem, weil ich rieche anders, meine Haut ist nicht mehr so straff». Und eigentlich denkt man: «Schauen Sie», ich sag das nicht aber wenn man alle Gleichaltrigen anschaut, dass, ihr Problem ist Älterwerden, aber ja. Man versucht es dann irgendwie an Hormonproblemen oder so, an etwas woran, äh wo man damit umgehen könnte, fest zu machen.

MS: Ok. Ähm. Nun hab' ich eine Fallvignette dabei. Ich bitte Sie diese einfach mal durchzulesen. Ähm. [Sucht Fallvignette 1]. So.

[MS legt Fallvignette 1 hin. IP2 liest Fallvignette 1 durch.]

MS: Meine Frage dazu wäre jetzt: Was fällt Ihnen hier auf, was für eine psychische Störung könnte diese Lea haben?

IP2: |**<< Begründung**|Ich mache keine psychischen Diagnosen. Weil ich finde ich bin keine Psychiaterin, keine Psychologin.|*Begründung >>*| Ähm, |**<< vermindertes Selbstwertgefühl und Selbstvertrauen**|aber, also es geht ja sicher einfach um ihr Selbstgefühl und ihre, ihre Identität und um die Zufriedenheit mit sich selbst,|*vermindertes Selbstwertgefühl und Selbstvertrauen >>*| also ich denke nicht das, dass jetzt objektiv, äh. Ich würde das jetzt nie so schreiben [zitiert Fallvignette]: «in Wirklichkeit ist sie attraktiv». Äh, anderen Leuten erscheint sie attraktiv. Aber es sind ja immer subjektive Eindrücke. Aber, so wie das tönt hat man ja das Gefühl sie hat, äh, ein Problem mit ihrem eigenen Selbstwertgefühl.

MS: Mhm, und woran machen Sie das fest? Also in welchen -

IP2: Es ist eigentlich sehr suggestiv geschrieben ((lacht)). [zitiert Fallvignette]: «Sie denkt jede Sekunde daran». Sie leidet, also der Leidensdruck. Sie hat ein soziales Vermeidungsverhalten, was auf eine Pathologie hin spricht. Und, ähm. Also wieso Selbstwertgefühl? Weil's ja eigene Beschreibungen sind über ihr äusseres Aussehen. Sagt jetzt nicht über, ob sie andere sogenannte innere Werte hat, die sie vielleicht mehr schätzt, aber vielleicht würde man da ähnliches finden. Aber, ähm, also es sind in dem Sinn ja keine objektiven, ähm Schönheitsmakel wie das jetzt vielleicht ein grosses Muttermal oder eine Warze auf der Nase wäre. Sondern subjektive Beschreibungen. Also, Haare können wahrscheinlich nie einfach hässlich sein. Das ist immer relativ, also -.

MS: Okay. Ich habe eine kleine Auflösung von diesem Fallbeispiel. Und zwar ist das ein Fallbeispiel einer Körperdysmorphen Störung. Ähm, haben Sie davon schon mal gehört?

IP2:|**<< KDS unbekannt**| Also. Nein, eben ich, ich kenn die ganzen Diagnosen wirklich nicht. Ähm, ich weiss, es ist mir schon bewusst, mit äh, mit den Dysmorphien, dass das zum Teil ganz extrem ist, dass man das ganz wirklich extrem wahrnimmt, dass die Nase gross ist und so weiter.|*KDS unbekannt >>*| Selbst hab' ich jetzt nie konkret damit zu tun gehabt. Und ich denk eben, weil da die Brüste sind und so, ja aber, nein, erzählen Sie. Es ist spannend.

MS: Ich habe jetzt mal die DSM-5-, Sie arbeiten wahrscheinlich mit dem ICD-10, oder?

IP2: Nein.

MS: Oder gar nicht?

IP2: Wir sind davon ganz befreit ((lacht)).

MS: Okay. Ich habe jetzt mal die hier dabei [zeigt DSM-5 Kriterien], weil die ein bisschen schöner beschrieben sind, die Symptome. Wie Sie eben gesagt haben, es ist eine übermässige Beschäftigung, darum steht auch jede Sekunde, mit einem wahrgenommenen Mangel oder Defekt. Der eben für andere nicht erkennbar ist oder nur geringfügig ist. Sie machen wiederholende Verhaltensweisen, wie sie überprüfen sich ständig im Spiegel, übermässige Körperpflege. Und sie fragen auch ständig nach bei Anderen: «Sieht das normal aus, oder ist das schön?». Oder vergleichen sich eben mit Anderen. Und es ist eben mit einem hohen Leidensdruck verbunden. Sowohl sozial, beruflich als auch in anderen Funktionsbereichen. Und die Bedenken dürfen eben nicht nur mit dem Körperfett oder Körpergewicht zusammenhängen also man muss auch eine Essstörung ausschliessen. Genau. Im ICD-10 gibt es das auch, das ist dann einfach [zeigt ICD-10 Kriterien] als Dysmorphophobie, wie Sie's gesagt haben, klassifiziert. Aber das kriegen Sie nachher auch. Genau. Gut. Jetzt haben Sie zwar die Frage schon ein bisschen vorne weg genommen. Die

nächste Frage wäre jetzt eben noch -. Ich habe noch eine ausgelassen ehrlich gesagt. Ähm. Wenn Sie jetzt bei diesem Fallbeispiel schauen, welche Informationen fehlen Ihnen denn noch. Oder was würden Sie diese Patientin noch fragen, wenn sie jetzt so zu Ihnen käme?

IP2: (1). Ich weiss jetzt nicht genau, äh, worauf Sie... Also, ähm, vielleicht ein bisschen Umweg, aber was wir eben noch häufig -. |**<< Labien erwähnt**|Wir sehen so andeutungsmässig Frauen natürlich einerseits wegen den Brüsten aber andererseits wegen den Labien, oder. Die Labien -, was zum Teil wirklich eine extreme Diskrepanz ist was, was sie selber denken und was nicht.|*Labien erwähnt >>*| Und häufig kommen sie dann sogar zu uns wenn sie schon x Operationen hinter sich hatten. Also die gehen ja dann häufig auch wirklich suchen sich plastische Chirurgie die irgendwie mehr zerstören das, als äh, hilfreich sind. Sie ist erst 13 [spricht Fallbeispiel an], «kleine hängende Brüste», also.

MS: Also jetzt ist sie 25.

IP2: Aha nein, jetzt ist sie 25.

MS: Aber seit sie 13 ist -

IP2: Ja genau, genau. Ja also muss man vieles Anderes noch fragen. Wir wissen jetzt, aber ich weiss nicht was Sie, was Sie jetzt von mir erwarten ((lacht)). Also ich -

MS: Gar nichts, gar nichts, ist nur eine offene Frage. Was Sie denken.

IP2: Also sie käme ja gar nicht zu mir, als Gynäkologin mit diesen Symptomen. So, oder. Wenn dann wäre die Frage an mich, ähm, ob sie eine Brustoperation haben könnte. Und ich würde sicher zuerst dann grundsätzlich mehr über diese Frau wissen wollen. Was macht sie sonst im Leben? Eben, hat sie schon, also, wie bewertet sie sich dann sonst? Was hat sie für ein soziales Netz? Ähm, gibt es Seiten die sie an sich auch gut findet? Und so weiter. Und, und sicher dann, eben ich mache keine Psychotherapie, absolut nicht aber, äh, dass man eher das Positive stützt als da sich zu sehr auf diese Defekte, scheinbaren, einlässt.

MS: Okay. Dann, Sie haben schon ein bisschen gesprochen von den Frauen, eben wegen den Operationen. Die Frage ist nun: Was sind denn Ihre Erfahrungen mit Patientinnen, die eben zum Beispiel äussern, dass Sie nicht zufrieden sind mit dem Aussehen, zum Beispiel eben der Brüste. Was haben Sie das schon für Erfahrungen gemacht?

IP2: Alles Mögliche.

MS: Alles Mögliche?

IP2: Also, eben. Ich bin selber sehr zurück -, oder, ähm, nicht nur zurückhaltend mit empfehlen, ich äh, warne die Frauen wirklich vor Operationen. Weil ich wirklich viel Erfahrungen habe wo sie dann eben erst im Nachhinein kommen und es irgendwann mal dann wirklich ein Problem ist. Also ich meine, Brust geht noch einigermassen aber wie gesagt bei den Labien ist das wirklich häufig, dass die wirklich nach einer Operation die Labien dann viel zu klein sind oder dann wirklich asymmetrisch, oder schmerzen mit den Narben. Bei der Brust wenn das ein guter Chirurg gemacht, ist das wahrscheinlich ein bisschen weniger das Problem aber auch da hab ich wirklich viele Frauen die nach Jahren kommen und sagen: «Es war für mich damals so ein grosses Problem, dass ich's gemacht hab, aber ich würd es nie mehr machen». Und das eigentlich bereuen nachher. Und einerseits sag ich ihnen das, ganz klar, dass es, dass die Chirurgie, dass ich nicht denke, dass die Chirurgie ihr Problem löst. Und wenn ich jetzt soweit wäre, dass ich das eben auch so hören würde, wie Sie das da sagen, dann, äh, erst recht natürlich. Aber, ähm, heutzutage denk ich sind auch, also die seriösen plastischen Chirurgen wirklich oftmals so, dass die Frauen dann auch von denen wieder zurückgeschickt werden. Nicht eben, es kommt auch

da sehr, gibt sehr grosse Unterschiede. Man muss, ich finde, das ist, das gehört eben auch zur Körperverletzung. Sie haben da gehabt, äh, Hautausdrücken und so, immer wieder korrigieren und ich finde das ist wirklich auch eine Körperverletzung dann jemanden so operieren.

MS: Ähm, dann ist die nächste Frage auf welche Herausforderungen treffen Sie mit solchen Patientinnen?

IP2: Zum Teil ist das, äh, sehr schwierig, also bisschen ähnlich, Sie haben vorher die Essgestörten auch angesprochen. Äh, weil die zum Teil sehr perseverieren auf diesen Problemen und sehr wenig einsichtig sind, dass es sich auch um etwas anderes, eben also, ich äh, ich würde jetzt nie einer Frau eine solche Diagnose mitteilen oder so. Sondern eben ich spreche eher von, ähm, eigenem Selbstwertgefühl oder sich selber akzeptieren können oder ähm, aus dem eigenen Leben einen sinnvollen Weg finden, eine sinnvolle Herausforderung finden. Und versuche sie dort dann irgendwie zu motivieren. In der Regel ist meine Aufgabe als Frauenärztin, finde ich, die Frauen so weit zu bringen, dass sie Einsicht haben darin, dass man auf einer anderen Ebene auch ansetzen sollte. Und das ist häufig nicht einfach. Und, ähm, ich versuche dann jeweils nicht so zu, also ich sage jeweils nicht: «Sie spinnen, schauen Sie sich doch an, ist alles normal». Weil dann, dann sehe ich sie einfach nicht mehr. Sondern ich versuche schon, äh, heraus zu spüren, wo ist ihr Leidensdruck und natürlich das sachlich zu, ähm, relativieren. Aber vor allem dann eben auch einfach weiterfragen und auf das Leiden einzugehen und versuchen, es geht darum, dass wir sie von diesem, eh, fast zwanghaften, Festhalten am Aussehen irgendwie etwas ablenken können. Und das finde ich die grosse Herausforderung, was häufig nicht einfach ist. Aber da bin ich immer ganz froh, wenn ich das schaffe, wenn eine Frau sagt: «Ja, nein wir versuchen jetzt das». Und ich denke für die Psychologinnen ist das sonst ja auch schwierig mit jemandem, also wenn jemand nur quasi gezwungen wird zur Psychologin zu gehen, dann weiss ich auch nicht wie einfach die Therapien dann sind.

MS: Also wäre dass dann ein Schritt von Ihnen, dass Sie die Frauen weiterschicken würden?

IP2: Ja, ja, ja.

MS: Zum Beispiel.

IP2: Also eben, sie kommen mit dem körperlichen Symptom, ich merke es geht um mehr als um etwas Körperliches. Also es geht in dem Beispiel jetzt wahrscheinlich sogar gar nicht um etwas Körperliches. Bei den Schmerzsyndromen ist das zum Teil dann noch etwas mehr vermischt. Ähm. Und, und dass wir da eine gemeinsame Ebene finden. Sie mit ihrem zwanghaften Körperbewusstsein und ich mit meiner Hypothese es geht um etwas anderes. Das wir da am Schluss merken, es gibt einen irgendwie einen gemeinsamen Nenner, wo das Leiden liegt und wie wir jetzt damit, wo wir ansetzen daran.

MS: Und was sind dann die Schritte, also was würden Sie dann machen?

IP2: Und dann würde ich sie wirklich zu einer Therapeutin schicken.

MS: Ja. ja.

IP2: Und natürlich immer auch mit der Möglichkeit, dass sie sich auch bei mir nochmals melden kann. Eben zum Teil gibt es ja, äh, zum Beispiel gibt es bei den Brüsten dann zum Teil, eine Brust ist viel grösser als die andere, oder so. Gewisse Sachen wo’s auch Sinn macht das in einem gewissen zeitlichen Abstand, das noch mal zusammen anschaut, dass sie sich auch von mir jetzt auch nicht einfach abgefertigt, ähm, fühlt. Das ist komischerweise ja zum Teil, oder mindestens am Anfang erleben sie das fast wie eine Niederlage, wenn die somatische Ärztin sie dann nicht will, sondern findet: «Du spinnst jetzt, du musst zur

Psychologin». Dieser Eindruck darf dann auch nicht entstehen.

MS: Ja, ähm, die Frage hatten wir eigentlich schon fast. Ist meine letzte: Wie wäre Ihr professionelles Vorgehen wenn sie bei einer Ihrer Patientinnen den Verdacht jetzt auf diese Störung, die wir gerade vorher gesehen haben, haben, was würden Sie da -?

IP2: Eben zuerst einmal mit der Patientin selbst, also ich würde ihr nicht einfach sagen: «Schauen Sie, das ist nicht so, ähm kann das nicht bestätigen, ich empfehle Ihnen eine Psychologin auf zu suchen». Sondern, ich würde sie wahrscheinlich einfach auch, äh, nochmals sehen, und vielleicht nochmals sehen und äh immer -. Es ist wirklich ganz häufig so, dass man dann bei zweiten, beim ersten Kontakt sondiert man so ein bisschen vor und drängt sie nicht in die Ecke, sondern -. Mein, mein Ziel ist immer Raum zu schaffen damit sie merkt, das ist ein Ort wo ich über Sachen reden darf, die nicht einfach sind und die mich belasten. Und ich habe, heute morgen oder gestern gerade jemanden gehabt, die hinkt immer. Und es ist offensichtlich kein körperliches Hinken, das sieht man schon am Hinken an. Und ich habe die jetzt auch schon zum dritten Mal gesehen. Ich bin einfach immer ganz höflich und neutral und sage nicht, weder: «Sie sind eine arme», noch -. Und heute erzählt sie mir das erste Mal: «Jetzt ist das Trauma wieder da». Also plötz-, das erste Mal wo sie wie auf eine andere Ebene geht. Und das ist so, mein Ziel. Eben einen Raum schaffen wo sie sich öffnen können und, und so lange dranbleiben bis das irgendwie gelingt. Und dann, kann ich sie weiter weisen.

MS: Mhm, ja.

IP2: Aber dann ist die, eine innere Motivation auch da.

MS: Danke.

OD: Gut dann mach ich mal weiter. Ähm, Sie haben schon ein bisschen angesprochen. Ähm, was sind Ihre Erfahrungen mit Patientinnen die Fragen zur Ästhetik oder zur Normalität ihres eigenen Genitalbereichs haben?

IP2: Ähm (2). |**<< Verzerrte Wahrnehmung**|Sehr häufig würde ich jetzt mal sagen, ist das wirklich eine Diskrepanz zu ihrem subjektiven Empfinden. Weil meistens stört's ja dann irgendetwas, sonst fragen sie das eher nicht. Und dem was ich objektiv finde.|*Verzerrte Wahrnehmung >>*| Also, es gibt ja einfach grosse Unterschiede und die einen stört das überhaupt nicht und andere stören Sachen wo's wirklich überhaupt nicht nachvollziehbar ist, von mir. |**<< Expertise nutzen**|Und ja, eben also, ich versuche als erstes immer wirklich so sagen: «Schauen Sie ich sehe so viele Frauen und i|**<< Vielfalt**|ch kann Ihnen sagen das ist so eine grosse Bandbreite.|*Expertise nutzen >>*| |*Vielfalt >>*|**<< Medien**|Die Norm ist nicht nur so wie sie das vielleicht in einem Heftli sehen oder so.|*Medien >>*| |**<< Normalität betonen**|Und es ist völlig normal». Das versuch ich schon, |**<< Ernstnehmen**|ich sag eben nicht: «Sie spinnen».|*Ernstnehmen >>*| Aber ich versuche ihnen aufzuzeigen, dass es normal ist.|*Normalität betonen >>*| |**<< Labien**|**<< Abnehmend**|Und eben das mit der Grösse der Schamlippe ist, ist was häufiges.|*Labien >>*| Interessanterweise würde ich jetzt sagen in meiner Sprechstunde in den letzten ein, zwei Jahren weniger häufig schon fast wieder als, als noch davor. Aber es kann jetzt durchaus auch ein Zufall sein.|*Abnehmend >>*| |**<< Schwangerschaft/Geburt**|Und das andere ist, hab ich jetzt aber auch länger nicht mehr gehabt, dass sind dann wieder eher Frauen die schon geboren haben, |**<< Andere Operationen**|die finden es klafft jetzt alles.|*Schwangerschaft/Geburt >>*| Und das ist irgendwie für den Mann nicht mehr schön, es ist alles zu locker. Und geht aber dann eigentlich ganz in eine ähnliche Richtung. |**<< Abratung von OP**|**<< Erwähnen OP**|Eine Operation macht das in der Regel nicht besser.|*Andere Operationen >>*|

|*Abratung von OP >>*|*Erwähnen OP >>*|OD: Aber dann würden Sie sagen, dass kommt eigentlich relativ häufig vor?

IP2: Ähm. |**<< Viele Fragen** |Also es ist jetzt nicht etwas Alltägliches, ähm. Eben also, wenn Sie mich jetzt vor zwei Jahren gefragt hätten, hätte ich wahrscheinlich gesagt, ich hab das fast einmal die Woche oder so.|*Viele Fragen >>*| |**<< Monatlich**|Aber im Moment würde ich sagen einmal im Monat. Also nicht ganz was Exotisches aber doch was, was man immer wieder sieht.

|*Monatlich >>*|OD: Ja. Gut. Und, ja das haben Sie auch schon ein bisschen gesagt, wie gehen Sie dann vor?

IP2: Ähm. |**<< Individualität**|Man muss natürlich schon auch ein bisschen unterscheiden oder. |*Individualität >>*|Also es gibt dann schon auch noch Unterschiede in der Nuance und |**<< Direkt OP**|ob sie schon mal eine Operation gehabt haben oder nicht. |*Direkt OP >>*|**<< Aufklärung**|Aber eben grundsätzlich denke ich es ist wirklich ganz wichtig, dass das und allein einfach auch bewusst ist die Norm wird ja immer enger. Das ist immer mehr gleich schon pathologisch in vielen Bereichen.|*Aufklärung >>*| |**<< Normalität betonen**|Und, und ähm, in diesem eben besonders auch und dass ich wirklich einfach versuche, äh, Ihnen möglichst bewusst zu machen, dass, dass es erstens normal ist |*Normalität betonen >>*|und |**<< Aufklärung OP**|zweitens Chirurgie da nicht einfach einen neuen Körper gibt oder was austauscht. Sondern es ist ein Eingriff in den Körper, es bleibt eine Narbe. Und die Gefahr, dass es nachher schlechter ist als vorher ist fast immer hoch.|*Aufklärung OP >>*| |**<< Sinnvoll**|**<< Medizinische Beschwerden**|Also eben, gibt natürlich gewisse Ausnahmen. |**<< Brüste**|Gibt Frauen die haben wirklich extrem eine Brust sehr gross und die andere fast nicht angelegt und so das ist dann ganz eine andere Situation natürlich.

|*Sinnvoll >>*|*Brüste >>*|*Medizinische Beschwerden >>*|OD: Und, fragen Sie auch manchmal bei Ihren Patientinnen nach, ob Sie mit der Ästhetik zufrieden sind?

IP2: |**<< Fragen nicht nach**|Nein. Weil ich sie gar nicht auf, darauf aufmerksam machen möchte, dass das ein Problem sein könnte.

|*Fragen nicht nach >>*|OD: Ähm. Ja, die nächste Frage haben Sie auch schon ein bisschen angesprochen. Was sind Ihre Erfahrungen mit Patientinnen die Schönheitsoperationen am Genitalbereich wünschen?

IP2: Ähm. Es gibt eben zwei-, |**<< Medizinische Beschwerden**|es ist vielen Frauen auch schon ein bisschen bewusst und viele sagen dann schon von Beginn weg, es ist zum Beispiel beim Velofahren klemmt das ein auf dem Sattel oder so, das ist etwas was manchmal kommt|*Medizinische Beschwerden >>*|. Ähm, |**<< Aufklärung OP**|und da ist eben meine Argumentation eben auch die Narbe kann schmerzen und.|*Aufklärung OP >>*| |**<< Aufklärung**|Eben also, ich weiss nicht, die Skizze sehen Sie nicht im Interview aber es ist, das Genital der Frau ist nämlich ein bisschen komplizierter als man sich das gemeinhin vorstellt. Ich zeige Ihnen das dann auch jeweils auf. |*Aufklärung >>*|[Holt ein Blatt und beginnt zu zeichnen]. [Zeichnet erst einen Kreis]. Also das wäre jetzt quasi so eine Vagina. Und dann ist die Klitoris hier oben. [Zeichnet Klitoris]. Und die Klitoris hat ja eine Vorhaut, ähnlich wie beim Mann. [Zeichnet Klitoris-Vorhaut]. Und diese Vorhaut geht dann direkt über in die kleinen Schamlippen und es geht immer um die kleinen Schamlippen. [Zeichnet Schamlippen]. Und dann gibt es da immer noch so eine Falte drauf auch [Zeichnet Falte auf Schamlippen]. |**<< Uninformiert**|Und wenn jetzt eine Frau relativ grosse kleine Schamlippen hat und dann haben die manchmal das Gefühl, ja kann man ja einfach da durchschneiden. |*Uninformiert >>*|[Zeichnet Schnitt durch kleine Schamlippen]. |**<< Kompliziert**|Aber nachher hat man so eine Stufe und das sieht dann schrecklich aus und das ist eben gar nicht einfach, dass man dann die Form man müsste dann quasi bis hier oben Mitte sezieren, dass man das wirklich eine schöne Kontinuität schafft, das ist schwierig. Und das müssen sehr erfahrene Leute machen. |*Kompliziert >>*|**<< Gelder**|Die Krankenkassen zahlen es meistens nicht. Ich habe letzthin eine gehabt die's bezahlt bekommen hat aber ich hab' sie jetzt noch

nicht gesehen und weiss noch nicht wie zufrieden sie ist. Ähm, das heisst man macht es möglichst billig.|*Gelder >>*| Das heisst nicht in Vollnarkose, sondern in Lokalanästhesie. Das Gewebe hier schwillt extrem schnell an. Also sie spritzen das 10 Milliliter, äh, Vidokain und es ist nachher einfach ein Ball. Da sehen sie überhaupt nicht mehr wo können sie das anpassen. |**<< Kompliziert**|Also so banal dass das ist, es ist eine schwierige ästhetische Operation |**<< Komplikationen** |und entsprechen wird es häufig nicht gut.|*Kompliziert >>*| Und das Hauptproblem ist, dass häufig zu viel entfernt wird. Und die kleinen Schamlippen haben ja auch eine Funktion. Die schützen den Eingang der Vagina. Und wenn die dann nicht mehr da sind dann ist einfach alles offen. Und die Harnröhre liegt auch noch hier [Zeichnet Harnröhre] drunter. Dann ist dann einfach ein wichtiger Schutz nicht da.

|*Komplikationen >>*|OD: Gut. Ähm, dann hab' ich auch noch ein Fallbeispiel dabei. Können Sie das noch durchlesen

[OD legt Fallvignette 2 auf. IP2 liest Fallvignette 2 durch.]

IP2: Das ist noch gut hat sie wenigstens einen guten Freund. ((lacht)). |**<< Viele KDS**|Also eben, also das ist jetzt was ich wirklich sehr häufig höre. Das könnte jetzt aus meiner Praxis direkt sein.

|*Viele KDS >>*|OD: Ja, das ist also einfach ähm, die Auflösung: Das wäre jetzt ein Beispiel von Körperdysmorpher Störung vom weiblichen Genitalbereich. Also es kann ja jedes Körperteil betroffen sein von dieser Störung und das wäre jetzt ein Beispiel des Genitalbereichs.

IP2: Genau das, wenn das habe ich jetzt nicht gewusst, dass Sie das schon so äh, mit einer Diagnose klassifizieren. Aber ich denke, ja äh, würde mich dann natürlich schon noch interessieren|**<< Zunehmend**| ich habe immer das Gefühl das in die-, eigentlich vor 20, 30 Jahren noch kaum gab, jetzt im Genitalbereich|*Zunehmend >>*|. Da ist auch so viel Aufmerksamkeit drauf. Also eben ich habe da nur schon ein bisschen äh, bei der Wortwahl. Ähm. [zitiert Fallvignette], «Die Schamlippen seien zu lang, so dass sie über die - » |**<< Normalität betonen**|da würd' ich schon sagen: «Nein! Das ist einfach manchmal so». Das heisst nicht, das ist nicht ein Argument, dass sie zu lang sind.|*Normalität betonen >>*| Und warum es sie seit einem Jahr belastet, die waren ja schon, also es ist unwahrscheinlich, dass sie von 20 bis 21 gross gewachsen sind, auch dass |**<< Veränderungen**|das kann dann wirklich in der Adoleszenz, sagen wir von 14-18 kann sich das verändern. Und es kann sich auch da wieder verändern sagen wir mit den Wechseljahren dann gibt's eine andere Fettverteilung.|*Veränderungen >>*| Aber das ist nicht unbedingt einsehbar, warum dass sie jetzt erst seit einem Jahr so ist. |**<< Verzerrte Wahrnehmung**|Und das mit den öffentlichen Umkleidekabinen ist auch ein bisschen absurd. Es würde da ja niemand so schauen.|*Verzerrte Wahrnehmung >>*| Und das Problem ist tatsächlich, dass es offensichtlich zum Teil eben Männer gibt die wirklich äh, solche Sachen meckern. |**<< Bemerkungen**|Also das, das hab ich relativ häufig dass sie finden «Mein Freund hat gesagt- , |**<< Uninformiert**|ich sehe-, ich sehe ja nicht so viele andere Frauen |*Uninformiert >>*|und mein Freund sagt, mit mir stimmt etwas nicht.»

|*Bemerkungen >>*|OD: Okay. Ja sie haben ja schon gesagt, dass Sie das kennen. Was sind denn so Ihre Erfahrungen mit Patientinnen die solche Symptome zeigen?

IP2: Wie das noch heraus kommt oder wie, äh ?

OD: Ähm ja, oder ja auch wie sie damit umgehen?

IP2:|**<< Visuell**|**<< Miteinbeziehen**| Ja, wir schauen das dann immer zusammen an auf dem gynäkologischen Stuhl wo man ja auch die Beine dann etwas gespreizt hat. Also man sieht es ja dann sehr gut. Ich habe ein Spiegel bei meinem Untersuchungsstuhl und wir schauen

das zusammen an.|*Visuell >>*|*Miteinbeziehen >>*| Und, eben, also, ich, das sehen sie ja jetzt hier nicht aber ja, ich wie gesagt äh, |**<< Aufklärung**|**<< Normalität betonen**|**<< Vielfalt**|betone dann wirklich, dass das normale viele Facetten hat und so weiter und, und rate ihnen oder warne sie wirklich aktiv davor, sich zu operieren zu lassen mit einer 21 sowieso. |*Aufklärung >>*|*Normalität betonen >>*|*Vielfalt >>*|Und wenn der Freund das auch noch findet, also sowieso. |**<< Subklinische Unzufriedenheit**|In meiner Erfahrung, glaube ich jetzt also die andere Fallvignette, hätte mich mehr beunruhigt als diese junge Frau, dass sie auch sonst ein Problem hat. Und eben sich selbst nicht annehmen kann, als wenn's nur wirklich das ist. Also ja, die sehe ich ja dann auch immer mal wieder.|*Subklinische Unzufriedenheit >>*| |**<< Überweisung an Chirurgie**|Es gibt schon die, die dann immer wieder kommen und sagen: «Also jetzt es ist mir jetzt einfach so wichtig». Und dann sag ich dann manchmal: «Ja aber wenn Sie's dann wirklich doch operieren wollen, sagen Sie's bitte mir und |**<< Gelder**|ich mache erstens eine Kostengutsprache |*Gelder >>*|und ich weise Sie zu jemandem der's kann. Und machen Sie nicht einfach irgendeine billige Operation». Aber diejenigen, die ich wirklich zuweise sind am Schluss ganz wenige.|*Überweisung an Chirurgie >>*| Und ich hab' ja in meiner Erfahrung auch nicht das Gefühl, |**<< Typ**|es sind schon, es ist ein bestimmter Typ Frauen. Und sind vielleicht schon Frauen die auch an anderen Orten dann manchmal kommen, ohne dass ich finde, die sind jetzt wirklich krank und so.|*Typ >>*| |**<< Selbst behandeln**|Aber nicht, dass ich wirklich finde die sind jetzt psychisch gestört. Also ich würde jetzt die glaube ich nicht einer Psychologin zuweisen.

|*Selbst behandeln >>*|OD: Okay. Ähm. Danke. Dann sind wir eigentlich schon bald fertig. Ähm, die letzten Fragen gehen so ein bisschen um Interesse an Fortbildung. Wie gross schätzen Sie das Interesse an einer Fortbildung, ähm, jetzt zu Körperdysmorphen Störung in der Gynäkologie ein?

IP2:|**<< Grosses Interesse an Fortbildung**| Das ist sicher, äh da. Also wir haben auch immer wieder solche Themen. Denn es sind viele wirklich betroffen auch davon und, und das ist so, ja ein Klassiker den man auch immer wieder bringen soll für auch die jungen Ärzte, dass einem das bewusst ist.

|*Grosses Interesse an Fortbildung >>*|OD: Und wie gross schätzen Sie das Interesse oder den Nutzen an Hilfsmittel zur Diagnostik einer Körperdysmorphen Störung, also zum Beispiel so ein Screening-Instrument oder so ein?

IP2:|**<< Kein Interesse an Diagnostik**| Da bin ich sehr skeptisch|*Kein Interesse an Diagnostik >>*| ((lacht)). Aber ich bin zu alt, wissen Sie ich, äh. |**<< Unpassend**|Ich ähm wehre mich ein bisschen dagegen, dass äh, dass jede Diagnose mit einer Checkliste und einem Score, äh belegt werden muss. Ich glaube die Wirklichkeit ist so häufig viel komplexer. |*Unpassend >>*|**<< Psychische Probleme im Hintergrund**|Und also ich glaube es ist sehr wichtig, dass das uns bewusst ist, dass eben nicht alles körperlich ist. Dass manchmal etwas anders dahinterstecken kann, dass wir auch hellhörig sind für solche Anliegen. Dass wir eben auch versuchen da hinter diesen vordergründig angebrachten Problemen noch weiter zu gehen.|*Psychische Probleme im Hintergrund >>*| Wie ich das vorher gesagt habe. Aber, ich meine es wäre viel einfacher wenn wir allen Assistenten sagen könnten okay und jetzt nehm' noch diesen Bogen und checkt ein -. Ich bin sehr skeptisch, ob das wirklich so viel weiterhilft. Ausser zu wissenschaftlichen Zwecken wo's natürlich völlig berechtigt ist. Persönlich würde ich das glaube ich nicht anwenden. Genau so wenig wie ich diese Schmerzskalen, das kennen Sie auch oder? Und es ist, es führt, also diese ganzen Formen jetzt so Problemkreise zwischen Körper und Psyche, sagen wir mal, die haben ja grundsätzlich eine Tendenz den eigenen Körper extrem mechanistisch zu sehen. Deswegen sprechen sie auch sehr an auf alles was man chirurgisch machen könnte. Die wären total begeistert von so einem Score. Und würden das auch sofort lernen ob sie da rein passen. Ich sehe immer mehr, kommen Frauen zu mir und sagen: «Dann hatte ich gestern Schmerzen auf der Skala von 10 war es eine 8» ((lacht)). Da bin ich nur schon, also ja, es ist äh, ja.

OD: Ähm. Und wie gross schätzen Sie das Interesse an Hilfsmittel für die Beratung von Patientinnen jetzt spezifisch für ähm Fragen zur Ästhetik des Genitalbereichs ein? Also es gibt ja so Tafeln oder so wo einfach verschiedene Genitalen abgebildet sind. So etwas.

IP2: |**<< Abhängig von Form**|Ja müsste man jetzt die Tafeln anschauen.|*Abhängig von Form >>*| Es ist etwas ganz Heikles, also ich habe da interessanterweise auch schon mit Studentinnen so vom wissenschaftlichen Zeichnen und so, und verschiedene so Projekte ein bisschen mitgema- äh begleitet, weil ich finde es ist ganz schwierig also wenn Sie Interesse haben kann ich Ihnen ein bisschen was zeigen, Sie haben vielleicht keine Zeit. Ich finde es ist ganz schwierig gute Abbildungen zu machen. Und auch da wieder, von mir aus, wenn ich Interesse hätte wären es nicht Tafeln, die Score 1 bis 2 und 3 und da ist's dann pathologisch und da ist es normal. Sondern wirklich mehr um die Breite der Norm zu illustrieren und dass müssten dann aber in dem Sinne auch nicht wirklich jetzt so frontal Aufnahmen äh. Quasi eine wissenschaftliche Vulva, sondern es muss dann mehr mit der, mit dem, ja eigenen Körper zu tun haben und es ist nicht einfach. |**<< Grosses Interesse an Hilfsmittel**|Also gute Tafeln grosses Interesse, schlechte Tafeln kann man nicht brauchen, gibt's schon genug.

|*Grosses Interesse an Hilfsmittel >>*|OD: Gut dann sind wir eigentlich fertig. Ausser Sie möchten noch etwas hinzufügen.

IP2: Nein.

Interview mit: IP3
Datum des Interviews: 09.05.2019
Dauer: 24 min
Anonymisierte Begriffe: keine
IP- Nummer: 3

MS: Dann beginnen wir doch mit der ersten Frage. Und zwar mit welchen psychischen Problemen kommen Patientinnen zu Ihnen in die Praxis? Was haben Sie da für Erfahrung?

IP3: Ähm eigentlich sehen wir die ganze Breite. |**<< Phobische Störung (F40) und andere Angststörungen (F41)**|Eigentlich bei jungen Frauen sind es häufig Angststörungen |*Phobische Störung (F40) und andere Angststörungen (F41) >>*|oder, ähm, |**<< Restkategorie Störungen**|einfach grundsätzlich Überlastung,|*Restkategorie Störungen >>*| Angst vor, ähm. Oder Zukunftsängste, nicht genügen zu können. Überlastung am Arbeitsplatz und ähm, je nach dem bei älteren Frauen sind es zum Teil auch krankheitsbedingte Sachen und dann sehen wir natürlich auch die psychisch kranken Leute, weil die Hausärzte auch oft die erste Anlaufstellen sind für psychische Erkrankungen und je nach dem wie offen die sind werden wir die auch weiter begleiten oder wir schicken sie in eine psychiatrische Behandlung. |**<< Depressive Episode (F32) und rezidivierende depressive Störung (F33)**|Aber da gibt es viel auch Depression|*Depressive Episode (F32) und rezidivierende depressive Störung (F33) >>*| ähm |**<< Nichtorganische Schlafstörungen (F51)**|viel, oft verknüpft mit Schlafstörungen|*Nichtorganische Schlafstörungen (F51) >>*| mit ähm ja. Einfach diese ganze Palette in diesem Bereich. |**<< Restkategorie Störungen**|Aber wir haben auch einige psychotische Patienten die halt weitere Behandlungen grundsätzlich ablehnen|*Restkategorie Störungen >>*| und wir sind dann so die einzige Anlaufstelle wo sie überhaupt noch kommen. Also von daher würde ich sagen ziemlich breit, vielfältig. Genau.

MS: Und Sie machen beides oder, Gynäkologie und auch Hausarzt-Tätigkeiten?

IP3: Genau.

MS: Für dieses Interview würden wir uns einfach auf den gynäkologischen Teil beschränken, wenn das geht. Dann ist die zweite Frage: Wie spielt denn die Zufriedenheit mit dem äusseren Erscheinungsbild, was spielt das für eine Rolle?

IP3: Also was ich sehe an Leuten die, die ähm psych-, oder mit ihren psychischen Beschwerden oder Problemen kommen auch in den gynäkologischen Kontrollen ist es oft, dass Patientinnen mit Übergewicht. Also sind oft Frauen die unzufrieden sind mit ihrem Gewicht und damit halt auch mit dem Erscheinungsbild. Bei jüngeren Frauen manchmal auch mit der Haut Probleme. Ähm, Akne die sie beeinträchtigt und stört ich, denke, dass sind so diese beiden Assoziationen die es häufig gibt.

MS: Ähm, Dann hab' ich nun ein Fallbeispiel für Sie. Ähm, ich würde Sie bitten, das einfach durchzulesen.

[MS legt Fallvignette 1 hin. IP3 liest Fallvignette 1 durch.]

IP3: Mhm.

MS: Ähm. Was fällt Ihnen zu diesem Fallbeispiel ein. Fällt Ihnen da irgendein psychisches Problem oder Störung ein oder was fällt Ihnen auf?

IP3:|**<< Körperwahrnehmungsstörung**| Okay, ja das ist ja eigentlich wirklich eine falsche Körperwahrnehmung.|*Körperwahrnehmungsstörung >>*| Ähm und es hat sicher eine psychische Ursache und ist ein Problem, dass ich hier vielleicht nicht in dieser Ausprägung

aber schon hin und wieder auch sonst sehe. |**<< vermindertes Selbstwertgefühl und Selbstvertrauen**|Das ich äh, ja dass einfach die Wahrnehmung durch halt ein schlechtes Selbstwertgefühl sehr beeinträchtigt ist und einfach nicht der Wirklichkeit entspricht.|*vermindertes Selbstwertgefühl und Selbstvertrauen >>*|

MS: Welche Stellen im Fallbeispiel, ähm sind Ihnen aufgefallen? Oder woran machen Sie Ihre Überlegungen fest?

IP3: Also es steht ja eigentlich genau so da ((lacht)). Genau, dass sie eigentlich keine dieser Merkmale hat oder dass sie eigentlich eine attraktive Frau ist, ich denke, das hat ja mit einer tieferen Störung zu tun, dass man das nicht adäquat einschätzen kann und dann kommt der soziale Rückzug der das ausgelöst und was natürlich dann die, diese negative Spirale verstärkt und weiter in äh, psychische Probleme führt.

MS: Wenn jetzt diese Lea zu Ihnen in die Praxis kommen würde, welche Informationen würden Sie sich noch, äh, wünschen von ihr?

IP3: Also mich würde eigentlich insgesamt ihre Anamnese interessieren. Ihre Familienkonstellation, wie sie aufgewachsen ist, ob sie Geschwister hat und dann auch wo sie sonst steht, beruflich in der Ausbildung. Was sie da überhaupt macht oder wozu sie auch Möglichkeiten hat also dass das denk ich das hängt dann auch sehr damit zusammen wie ihr Selbstwertgefühl ist und was sie da, oder wie stark sie da wirklich sozial auch eingeschränkt ist um sie dann auch weiter schicken zu können oder einfach eine gute Triage zu machen, was die weiteren Schritte wären.

MS: Ähm ich habe eigentlich eine Auflösung vom Fallbeispiel. Ähm ich weiss nicht vielleicht haben sie schon mal davon gehört. Ähm das wäre ein Beispiel für eine Körperdysmoprhe Störung, kennen Sie, haben Sie schon mal gehört? [Zeigt DSM-5 Kriterien]

IP3: |**<< KDS bekannt**|Mhm.

|*KDS bekannt >>*|MS: Eben wie im Beispiel eigentlich gestanden ist, ist eine übermässige Beschäftigung mit einem Mangel oder Defekt der eben für die Anderen nicht erkennbar ist oder nur geringfügig da ist. Ähm, sie haben wiederholende Verhaltensweisen, wie ständig überprüfen im Spiegel, übermässige Körperpflege. Sie vergleichen sich auch immer wieder mit Anderen. Ist eben ein sehr hoher Leidensdruck dann auch dabei. Beruflich, sozial und als Abgrenzung es darf sich dabei nicht um eine Essstörung handeln.

IP3: Ja, genau.

MS: Und es ist eben eine sehr seltene, also unbekannte Störung. Ungefähr 2% der Bevölkerung ist davon betroffen. Und das wäre jetzt einfach ein Beispiel gewesen dafür. Ich weiss nicht, arbeiten Sie mit dem ICD-10?

IP3: Eigentlich brauchen wir das nur bei den psychiatrischen Diagnosen, weil sonst wird es eigentlich gar nicht verlangt.

MS: Ja, einfach dass Sie's noch gesehen haben im ICD10 ist es dann unter Dysmorphophobie [zeigt ICD-10 Kriterien]. Jetzt wo sie ein bisschen die Symptome gesehen haben. Ähm, haben Sie denn schon irgendwelche Erfahrungen mit Patientinnen, die ähnliches berichtet haben?

IP3: In viel leichterer Ausprägung sehen wir das schon immer wieder. Gerade was wir vorher angesprochen haben. Vor allem auch die Haut ist so ein, ein Problem oder grad ähm, bei leichter Akne wo das völlig übertrieben und übergross dargestellt wird. Ähm, jedes kleinste, jede kleinste un- wie sagt man? Einfach dass es so gross wird, das ist so eine Variante. Und

sonst denk ich. Bei uns, ich habe nicht sehr viele Leute, die mit ähm die auch mit jetzt grad gynäkologischen Merkmalen starke Probleme haben, aber dass vermischt sich halt sehr stark jetzt bei uns. Hausarzt, Medizin, gynäkologisch das überschneidet sich in vielen Bereichen. Ähm, ja also in dieser Ausprägung habe ich jetzt eigentlich niemand der mir grad in den Sinn kommt.

MS: Dann würde dann auch diese Personen, die das vielleicht in leichterer Form haben. Was sind denn die Herausforderungen bei diesen Patientinnen?

IP3: Sie ernst zu nehmen, wenn ich das Problem vielleicht nicht sehe und herauszufinden warum das überhaupt so entsteht und, und was sie beschäftigt. Das finde ich einerseits schwierig. Aber anderseits finde ich es auch schwierig mich nicht darauf einzulassen und das zu verstärken sondern wirklich auch zu versuchen halt eine Aussensicht, eine neutrale Aussensicht zu behalten oder. Und das ist, denk ich eine Gradwanderung. Denn je neutraler man ist desto mehr denken sie ich nähme sie nicht ernst und je mehr ich mich darauf einlasse desto mehr unterstütze ich sie in ihrer Wahrnehmung und das finde ich gar nicht so einfach so in einer kurzen Viertelstunde irgendwie einen guten Zwischenweg zu finden. Das hängt aber auch stark damit zusammen wie gut ich die Patientin kenne. Und desto besser ich sie kenne desto einfacher wird es auch. Und was ich auch, ähm, eigentlich ganz spannend finde ist, wenn man so eine Vertrauensbasis hat, kann man das vielleicht mit etwas Humor auffangen. Also es gibt Leute die wissen genau, dass sie etwas übertreiben und die erkennen das mit der Zeit auch und dann kann man das irgendwie so ein bisschen humorvoll angehen und ich denk dann ist es ja auch noch nicht eine wirklich, eine schwere Pathologie. Aber ich denke, das ist schon, dass man das vielleicht früh auffangt oder merken kann grad in einer hausärztlichen oder gynäkologischen Sprechstunde. Da einfach eine Gegensteuer geben kann. Bei schwereren Ausprägungen, das das sind dann halt wirklich pathologische Verhaltensweisen wo ich denke da braucht es wirklich psychotherapeutische Betreuung und es geht uns da zu weit.

MS: Das wäre meine nächste Frage gewesen. Ähm, wie gehen Sie mit diesen Herausforderungen um? Was haben Sie da für Lösungen oder haben Sie da schon ein bestimmtes Vorgehen wie Sie das lösen können?

IP3: Äh, ja, Also Ich wiederhol mich jetzt wahrscheinlich ((lacht)).

MS: Macht nichts.

IP3: Ich denke es geht auch immer darum abzuschätzen, wie gravierend ist das wirklich. Wie wir am Anfang schon besprochen haben, was sind die Auswirkungen auf ihr Sozialleben, auf ihr berufliches Leben, wie stark schränken sie sich selber ein durch die, ja durch diese Wahrnehmung. Und wenn das noch nicht problematisch ist denk ich schon, dass wir, dass, dass ich das eigentlich auch ähm, zu einem gewissen Sinn versuche die Leute ein bisschen zu begleiten da drin. Und sie vielleicht aus auch aus irgendeinem Grund einfach regelmässig zu sehen und zu schauen, wo sie stehen und ähm aber auch offen darauf anzusprechen, dass vielleicht eine ähm Therapie auch eine Möglichkeit wäre, das wirklich adäquat anzuschauen. Grad bei jungen Frauen, versuch ich auch immer so ein bisschen darauf hin zu steuern, dass es auch eine Chance ist und eine Investition längerfristig, wenn sie das jetzt anpacken. Und von daher denke ich bin ich da relativ offen, sie wirklich auch vielleicht dazu zu bringen oder so ein bisschen anzusprechen, ob sie nicht eine Therapie in Anspruch nehmen möchten. Und wenn das absolut abgelehnt wird dann finde ich es eigentlich sinnvoll, dass man die wirklich begleitet und auch regelmässig sieht und grad gynäkologisch kommen sie ja sowieso regelmässig dann sind das ja gute Möglichkeiten, da immer wieder nach zu hacken und zu fragen und zu sehen wo sie stehen.

MS: Meine letzte Frage, aber Sie haben sie eigentlich jetzt schon beantwortet. Nämlich, eben wie wäre Ihr professionelles Vorgehen, wenn Sie bei einer Patientin jetzt den Verdacht

auf eine Körperdysmorphe Störung oder so hätten?

IP3: Also eben ich denke wirklich, wenn das etwas ist, was ich denke, dass ist relevant und es ist einschneidend für die Person dann würde ich versuchen sie irgendwie eine psychologische oder psychotherapeutische Stelle zu vermitteln und ja. Manchmal braucht es einfach auch Zeit oder, und manchmal braucht das auch einige Treffen und manchmal braucht das auch einige Jahre. Aber das wären so ein längerfristiges Ziel, auch wenn das nicht grad sofort geht.

MS: Dankeschön.

OD: Gut, dann übernehme ich jetzt. Was sind Ihre Erfahrungen mit Patientinnen die Fragen zur Ästhetik oder zur Normalität ihres eigenen Genitalbereichs haben?

IP3: Ähm. |**<< Typ**|**<< Unsicher**|Dass das grundsätzlich unsichere Patientinnen sind.|*Typ >>*|*Unsicher >>*| |**<< Bemerkungen**|Und oft wird diese Frage auch erst ausgelöst durch irgendeine Begegnung oder durch Bemerkungen, ähm. Oft fällt, fällt Frauen oder Mädchen lange nichts auf und dann macht irgendjemand eine Bemerkung und dann kann sie das wahnsinnig beschäftigen. |*Bemerkungen >>*|Ähm. |**<< Seltene Fragen** |Und dass sie, dass es jemand von sich aus einfach so, |**<< Jüngere**|also vor allem bei jungen Frauen, irritierend findet,|*Jüngere >>*| das, das erleb ich selten. |*Seltene Fragen >>*|**<< Medien**|Es ist meistens sie lesen etwas|*Medien >>*| oder sie hören etwas |**<< Bemerkungen**|oder sind halt wirklich konkrete Bemerkungen|*Bemerkungen >>*|. |**<< Medizinische Beschwerden**|Und dann gibt es aber schon auch die ganz eindeutigen Fehlbildungen und da finde ich es auch wieder erstaunlich, dass es oft lange nicht bemerkt wird. Ähm, oder erst wenn Probleme auftreten.|*Medizinische Beschwerden >>*| |**<< Vielfalt**|Was aber auch völlig verständlich ist, weil es ja auch keine absolute Norm gibt und die ähm, Normvaria- oder die Normbreite ziemlich gross ist.

|*Vielfalt >>*|OD: Ähm, und was ist Ihr professionelles Vorgehen, wenn solche Fragen kommen?

IP3: |**<< Visuell**|Dass wir das grundsätzlich |**<< Miteinbeziehen**|zuerst mal versuchen gemeinsam anzuschauen.|*Miteinbeziehen >>*| Einerseits zu objektivieren aber auch, äh, ich schau dass dann auch mit den jungen Frauen mit dem Spiegel an, dass wir zusammen schauen wo ist das Problem, was stört. |*Visuell >>*|**<< Normalität betonen**|Und nach einem vorerst objektiven Teil,|**<< Miteinbeziehen**| dass wir dann gemeinsam überlegen|*Miteinbeziehen >>*| ja ist das nun wirklich eine, eine ähm, wirklich ähm, eine Abnormalität oder ist es einfach eine Normvariante die absolut ähm, physiologisch ist. Und dann geht es ja auch immer drum rauszufinden, ähm, stört es wirklich oder ist es einfach nur eine Frage. Also manchmal möchten die ja einfach hören: «Das ist normal». Und dann ist es meine Herausforderung das auch ein bisschen zu spüren, |**<< Wenig Eingehen**|dass ich gar nicht viel darüber sage, sondern mich einfach kurz und bündig dazu äussere und sage, das ist normal.|*Normalität betonen >>*|*Wenig Eingehen >>*| Ich denk mal, wenn man dann zu weit ausholt oder zu weit versucht, das irgendwie normal zu reden, dann ist es sehr kontraproduktiv. |**<< Individualität**|Weil das ist manchmal ähm, also das ist für mich die Herausforderung zu merken möchten sie jetzt einfach ein «Ja, das ist normal» hören oder finden sie's wirklich nicht normal und dann braucht es ein bisschen mehr.|*Individualität >>*| |**<< Gespräch**|Da muss man ein bisschen weiter ausholen, dass man das dann aber nicht je nach dem ins Gegenteil verstärkt.

|*Gespräch >>*|OD: Ähm, fragen Sie manchmal selber bei den Patientinnen nach wie sie zur Ästhetik ihres Genitalbereichs stehen?

IP3: |**<< Fragen medizinisch**|Ähm, wenn mir etwas auffällt, dass ich denke das, das müsste man besprechen, dann kann es schon mal vorkommen aber eher selten, aber das sind dann

eher Sachen, wo ich dann denke, das ist vielleicht nicht normal oder ich müsste wissen ob, sich das verändert hat über die Zeit. Das kann dann auch mal Pigmentierungen sein oder Muttermale, wo's einfach irgendwie wichtig ist, dass man das wahrnimmt, weil das eine Stelle ist die man nicht einfach so für sich anschaut und ich denke, das ist dann auch irgendwie ein bisschen unsere Aufgabe da drauf aufmerksam zu machen, grad wenn es darum geht, dass man etwas beobachten soll. Abers sonst ähm, wenn ich denke, das ist im völlig normalen Bereich und hat keinen pathologischen Wert, dann eigentlich nicht.

|*Fragen medizinisch >>*|OD: Was sind Ihre Erfahrungen mit Patientinnen die Schönheitsoperationen am weiblichen Genitalbereich wünschen?

IP3: Ähm, |**<< Seltene OP**|ich hab' nicht sehr viele, die das wünschen und |**<< Entscheidung überlassen**|ich schieb das oder ich gebe da den Ball oft zurück. |**<< Überweisung an Chirurgie**|Also ich kann Ihnen Adressen angeben |*Überweisung an Chirurgie >>*|aber ich bitte sie oft sich da selber darum zu kümmern und auch zu informieren,|*Entscheidung überlassen >>*| |**<< Gelder**|ob das irgendwie übernommen wird von einer Kasse und das ist ja ähm also das braucht ja meistens eine ziemlich gute Indikation, dass da etwas übernommen wird|*Gelder >>*| und von daher, also wir sehen das nicht sehr oft.|*Seltene OP >>*|

OD: Ähm, ich habe jetzt auch noch mal ein Fallbeispiel, ich bitte Sie das mal durchzulesen.

[OD legt Fallvignette 2 hin. IP3 liest Fallvignette 2 durch.]

OD: Also das ist jetzt ein Beispiel einer Körperdysmorphen Störung eben des weiblichen Genitalbereichs. Also von der Körperdysmorphen Störung kann jedes Körperteil betroffen sein und das wäre halt ein Beispiel von einer Patientin die das am Genitalbereich hat. Ähm. Was sind Ihre Erfahrungen mit Patientinnen, die solche Symptome zeigen?

IP3: Mhm, |**<< Viele KDS**|das das hören wir ziemlich oft.|*Viele KDS >>*| Also das sind Frauen die häufig kommen. Ich glaub das war eine zeitlang auch in den Medien irgendwie ein Thema. Ähm und, |**<< Subklinische Unzufriedenheit**|also meine Erfahrung ist, dass die meisten mit denen man das wirklich auch bespricht und anschaut eigentlich damit umgehen können.|*Subklinische Unzufriedenheit >>*| |**<< Medizinische Beschwerden**|Und dann gibt es wirklich auch Frauen die halt wirklich ein, oder die wirklich auch Beschwerden haben. Und also grad Frauen die viel Rad fahren oder sonst Beschwerden haben, das also das kann vorkommen und dann muss man das besprechen und weiter anschauen.|*Medizinische Beschwerden >>*| |**<< Selbst behandeln**|Aber solange das sich im normalen Rahmen bewegt hatte ich eigentlich noch nie das Problem, dass sie das dann unbedingt auch behandeln wollten, sondern eigentlich wenn man das dann noch mal gemeinsam anschaut und bespricht war das jetzt bis her eigentlich in meiner Erfahrung kein grösseres Problem.|*Selbst behandeln >>*| |**<< Keine KDS**|Aber es gibt's natürlich, also ich weiss es also man hört es ja immer wieder. Aber ich kenn das jetzt nicht aus eigener Erfahrung.

|*Keine KDS >>*|OD: Ähm, darf ich fragen was heisst ziemlich oft? Wenn Sie das irgendwie -?

IP3: |**<< Jüngere**|Bei jungen Frauen, also die Frage ja, |**<< Labien**|dass die inneren Schamlippen die sind gross,|*Labien >>*| |**<< Monatlich**|das hören wir jeden Monat eins, zwei, drei Mal also.

|*Jüngere >>*|*Monatlich >>*|OD: Okay.

IP3: |**<< Subklinische Unzufriedenheit**|**<< Normalität betonen**|Das sind, das sind oft auch ganz neutrale Fragen, wie ich vorher erwähnt habe, oft geht es ja wirklich nur darum zu sagen, es ist normal und dann hat es ja auch keinen pathologischen Wert. Genau.

|*Subklinische Unzufriedenheit >>*|*Normalität betonen >>*|OD: Wenn jetzt genau diese Patientin [zeigt auf Fallvignette] zu Ihnen kommen würde, wie würden Sie dann mit ihr vorgehen?

IP3: (2). Ähm. (2). Also ich denke, das, das ähm, bräuchte ja zum das wirklich gemeinsam intensiver anzuschauen wie noch mal einen, einen zweiten Termin. Also ich denke in einer allgemeinen Kontrolle würde das den Rahmen wie sprengen. |**<< Anamnese**|Da etwas raus zu finden, woher das kommt. Aber ich würde, ich würde wirklich, also. Sie schreiben da, dass das ein neues Problem ist, dass das ein Jahr jetzt vielleicht so, ähm, sie beschäftigt und ich würde mal versuchen rauszufinden was hat das überhaupt ausgelöst, woher kommt diese Idee überhaupt. Die scheint vorher nicht da gewesen zu sein. Das scheint problematisch gewesen zu sein.|*Anamnese >>*| Und dann je nach dem halt da dran anzusetzen,|**<< Bemerkungen**| also hat sie das einfach gelesen oder gab es konkrete Bemerkungen die sie gehört hat.|*Bemerkungen >>*| Oder um was geht es überhaupt. Und, ähm, |**<< Gespräch**|wenn wir dann im Gespräch merken eigentlich hat sie etwas stark verunsichert aber vorher hatte sie gar kein Problem damit dann denk ich dann kann man das wirklich auch versuchen so normal anzuschauen|*Gespräch >>*| und damit um zu gehen und wenn, wenn das aber dann wirklich so darauf hinausläuft, dass das irgendwie |**<< Sexualität**|ein tiefer liegendes Problem ist vielleicht auch mit ihrer Sexualität |*Sexualität >>*|oder Erfahrungen die sie negativ geprägt haben, |**<< Überweisung Psychotherapie**|ich denk dann muss man versuchen rauszufinden, ob da wirklich auch, ähm, eine Therapie nötig ist.|*Überweisung Psychotherapie >>*| |**<< Traumatische Erfahrungen**|Damit man das anschaut und vielleicht liegt ja noch etwas ganz anderes dahinter, vielleicht steht da auch irgendwie, eine schlechte Erfahrung mit einem Freund, Partner oder auch irgendwie ein Missbrauch dahinter.|*Traumatische Erfahrungen >>*| |**<< Anamnese**|Aber das müsste man zuerst sorgfältig anschauen.

|*Anamnese >>*|OD: Gut. Ähm. Zum Schluss fragen wir noch ein bisschen über Ihr Interesse. Ähm. Wie gross schätzen Sie das Interesse an einer Fortbildung in diesem Bereich ein. Also ich meine Interesse an einer Fortbildung zu Körperdysmorphen Störung allgemein in der Gynäkologie?

IP3: Ähm, |**<< Grosses Interesse an Fortbildung**|das wäre sicher interessant so in einem, das wär so eine Nachmittags-Fortbildung, ähm, ich in, so in diesem Rahmen, das fände ich durchaus interessant.

|*Grosses Interesse an Fortbildung >>*|OD: Und wie gross schätzen Sie das Interesse und den Nutzen an Hilfsmittel zur Diagnostik der Körperdysmorphen Störung ein? Also so ein Screening-Instrument oder so etwas in der Art?

IP3: |**<< Wenig Interesse an Diagnostik**|Nein, das würde ich jetzt glaub ich nicht sehr hoch gewichten. |**<< Unpassend**|Weil das so individuell und weil die Normbreite so, so riesig ist und was einem stört, ähm, ich denk das ist so etwas individuelles, das schätze ich schwierig ein, so etwas überhaupt zu entwickeln.|*Unpassend >>*| |**<< Bewusstsein**|Screening, dass man so ein bisschen Tools hat, daran zu denken und vielleicht zu fragen oder mehr in diesem Sinne?

|*Wenig Interesse an Diagnostik >>*|NA|OD: Ja, oder auch grad so Symptome abfragen in der Art und dann kann man vielleicht besser einschätzen wie pathologisch das jetzt ist?

IP3: Ja genau, genau. Das denk ich aber das hängt ja dann eher mit dem Bewusstsein zusammen, dass man überhaupt hat. |*Bewusstsein >>*|**<< Unpassend**|Also ich bin nicht so Fan von irgendwelchen Checklisten oder Aufgaben, weil auch das Gespräch ist immer so individuell und die Leute bringen so viel mit.|*Unpassend >>*| Aber ich denk, das Bewusstsein wirklich auch ein bisschen zu haben, das wäre dann auch die Fortbildung oder, dass man dann überhaupt, ja ein bisschen hinhört oder auch Fragen stellt, wenn man denkt, es könnte

irgendwie ein Problem sein. Das wäre durchaus sinnvoll.

OD: Und wie gross schätzen Sie das Interesse für so Hilfsmittel für die Beratung von Patientinnen jetzt mit dieser Symptomatik [zeigt auf Fallvignette 2], also mit Körperdysmorphen Störung des weiblichen Genitalbereichs ein? Also es gibt zum Beispiel so Tafeln wo einfach verschiedene Genitalen abgebildet sind, dass man so eine Variation sieht und sieht, dass eben verschiedenartige Ausprägungen gibt, dass nicht alle gleich aussehen.

IP3:|**<< Grosses Interesse an Hilfsmittel**| Ja, das wäre sicher hilfreich jetzt im Zusammenhang mit solchen Fragen, |**<< Vielfalt**|dass auch diese Norm oder diese Varianz einmal aufzeigen kann. |*Vielfalt >>*|Ähm. Genau. Ja, das, das fände ich etwas sinnvolles.

|*Grosses Interesse an Hilfsmittel >>*|OD: Gut dann sind wir eigentlich schon am Ende. Möchten Sie noch irgendetwas hinzufügen?

IP3: Mh, nein.

OD: Danke vielmal.

Interview mit: IP4
Datum des Interviews: 10.05.2019
Dauer: 27 min
Anonymisierte Begriffe: keine
IP-Nummer: 4

MS: Die erste Frage ist: Mit welchen psychischen Problemen kommen denn Patientinnen zu Ihnen in die Praxis?

IP4: Das ist sehr vielseitig. In der Grundversorgung, also ich bin Internistin und biete auch gynäkologische Sprechstunden an und ich habe daneben eine psychosomatische Ausbildung und eine Sexualmedizinische therapeutische Ausbildung und |**<< Nichtorganische Schlafstörungen (F51)**|dann kann das psychische Probleme, das kann von Schlafstörungen |*Nichtorganische Schlafstörungen (F51) >>*|**<< Sexuelle Funktionsstörungen, nicht verursacht durch eine organische Störung oder Krankheit (F52)**|bis zu Sexualstörung bis zu Vaginismus bis zu Orgasmusstörung|*Sexuelle Funktionsstörungen, nicht verursacht durch eine organische Störung oder Krankheit (F52) >>*|. Äh, |**<< Restkategorie Störungen**|psychische Probleme, familiäre Probleme, Berufsprobleme, Burnout, Mobbing.|*Restkategorie Störungen >>*| Heute, es ist die ganze Palette.

MS: Und wenn Sie das auf die gynäkologischen Patientinnen beschränken, geht das?

IP4: Ja, äh,|**<< Sexuelle Funktionsstörungen, nicht verursacht durch eine organische Störung oder Krankheit (F52)**| häufig hör ich Libidomangel,|*Sexuelle Funktionsstörungen, nicht verursacht durch eine organische Störung oder Krankheit (F52) >>*| |**<< Restkategorie Störungen**|Beziehungsprobleme,|*Restkategorie Störungen >>*| (.) Ähm, |**<< Sexuelle Funktionsstörungen, nicht verursacht durch eine organische Störung oder Krankheit (F52)**|Vaginismus, gelegentlich, Orgasmusstörung häufiger|*Sexuelle Funktionsstörungen, nicht verursacht durch eine organische Störung oder Krankheit (F52) >>*|, ähm, ja Beziehungsprobleme allgemein nicht nur sexuell natürlich. Ähm ja. Erregungsstörungen (2). So ein bisschen um das ein bisschen zu fokussieren auf den gynäkologischen Aspekt. |**<< Restkategorie Störungen**|Oder natürlich dann Kinderwunsch, und der Kinderwunsch der setzt sich nicht ein. Ja.

|*Restkategorie Störungen >>*|MS: Und bei diesen Patientinnnen, also den gynäkologischen Patientinnen, welche Rolle spielt denn die Zufriedenheit mit dem äusseren Erscheinungsbild?

IP4: Oh, ist sehr wichtig. Ich denke die Selbstwahrnehmung und auch die Selbstachtung, auch der Selbstwert, auch das sexuelle Erleben hängt sehr stark, ist sehr stark gekoppelt mit dem äh, Wohlbefinden. Jedes Menschen, speziell der Frau jetzt. Mit und in ihrem Körper.

MS: Mhm.

IP4: (.) Das ist eine ganz zentrale, ja, ganz zentral.

MS: Okay. Ich habe jetzt eine Fallvignette für Sie.

IP4: Ja.

MS: Ich würde Sie bitten diese mal durchzulesen.

[MS legt Fallvignette 1 hin. IP4 liest Fallvignette 1 durch.]

IP4: Ja, okay.

MS: Was fällt Ihnen bei diesem Fallbeispiel auf. Kommen Ihnen hier psychische Störungen in den Sinn?

IP4: Ja ich denke sie ist, es beginnt mit 13, sie ist mittlerweile 25, sie macht ziemlich grossen sozialen Rückzug. Sie geht nicht mehr an die Uni. Das ist bei mir, läutet die Alarmglocke. Sie geht nicht mehr an, sie hat keine sozialen Aktivitäten. |**<< vermindertes Selbstwertgefühl und Selbstvertrauen**|Sie ist sehr unsicher. Sie ist, ja, sicher auch unzufrieden aber sehr unsicher.|*vermindertes Selbstwertgefühl und Selbstvertrauen >>*| Sie fragt ihre Mutter. Sie ist jede Sekunde mit ihren sogenannten Makeln beschäftigt. Und ich kenne jetzt hier keine Ressource, was hat sie dann sonst, was dreht sich denn sonst denn noch in diesem Kopf ausser diesen Gedanken um ihr |**<< vermindertes Selbstwertgefühl und Selbstvertrauen**|Selbstwert. Also ich würde sagen ist eine schwere Störung, liegt da vor.

|*vermindertes Selbstwertgefühl und Selbstvertrauen >>*|MS: Mhm, Mhm.

IP4: Das würde ich mit 25, das ist eigentlich das Selbstwert, ja immer noch, ja ich denke das ist eine schwere Störung. Würde ich mal sagen, weil es ist doch jetzt schon chronifiziert, 12 Jahre oder. Und sie ist jetzt nicht mehr in der Pubertät auch nicht in der Pospu-. Sie ist eine erwachsene Frau.

MS: Wenn jetzt diese Patientin bei Ihnen wäre, welche ähm, zusätzlichen Informationen würden Sie sich noch wünschen von ihr?

IP4: Ja ich gehe immer ähnlich vor. Man hat ja als Mediziner manchmal auch da mal eine ausführliche Anamnese. Ein Gespräch über die Krankheitsgeschichte über das jetzige. Bioanam- ganze bio, soziale, somatische Konzept oder? Und äh, eben was hat sie für Ressourcen? Was hat sie für Beziehungen? Was hat sie schon für Erfahrung? Was bringt sie mit an Lebenserfahrung oder? Lebt sie noch zuhause? Lebt sie bei der Mutter? Oder ist sie in irgendeiner WG oder wie, ja ich würde natürlich persönlich bei ihr, sozial. Ich würde auch familiär, ich da, das ist, das kann man nicht in einer Sitzung von einer halben Stunde oder einer Stunde. Das braucht eine längere Begleitung von dieser jungen Frau.

MS: Ja, mhm. Ich habe jetzt eine kleine Auflösung vom Fallbeispiel.

IP4: Gut ((lacht)). Schön.

[MS legt die DSM 5-Kriterien hin.]

MS: Das (.) Dieses Beispiel wäre ein Fallbeispiel von einer Körperdysmorphen Störung. Haben Sie davon schon mal gehört?

IP4: |**<< KDS bekannt**|Klar, klar. Sie haben ja jetzt schon ein paar Beispiele mit Körperdysmorphen Störungen unterteilt oder?

|*KDS bekannt >>*|MS: Genau. Das wären einfach die Diagnosekriterien sozusagen. [Zeigt auf DSM-5 Kriterien.]

IP4: [Liest DSM-5 Kriterien durch]. Jetzt soll ich durchlesen und welches für mich am meisten zutrifft?

MS: Nein, nein. Das sind wirklich die offiziellen Kriterien. Und es ist einfach kurz zur Erklärung des Störungbildes. Eben es geht darum, dass sie sich nur mit diesem Mangel beschäftigen. Diese aber für andere nicht sichtbar sind. Darum steht hier auch «In Wirklichkeit ist sie attraktiv» [Zeigt auf Fallbeispiel].

IP4: Die Mutter findet sie normal. Genau.

MS: Genau das geht einher mit vielen repetitiven Verhaltensweisen, stundenlang im Spiegel anschauen, überschminken, Haare zurecht zupfen. Aber eben auch vergleichen mit Anderen und immer wieder nachfragen bei anderen Leuten «Seh ich normal aus?» Und dann eben der hohe Leidensdruck. Sozial, beruflich und in anderen Gebieten. Und das ist einfach zur Abgrenzung, dass es nicht -

IP4: [Und beeinträchtigt, das ist ganz wichtig, nicht nur Leidensdruck. Und sie funktioniert ja eigentlich nicht mehr, weil sie geht nicht mehr raus. Sie ist wirklich eingeschränkt. Schwer, oder? Sozial, beruflich oder vielleicht auch in der Beziehungsaufnahme zu einem anderen Menschen oder einem Mann, oder, wenn sie bisexuell orientiert ist, oder das wissen wir alles ja nicht.

MS: Genau und einfach dass es keine Essstörung ist das wäre zur Abgrenzung.

IP4: Genau, klar. Kann auch noch dabei sein aber das müsste man alles genau dann von ihr erfahren.

MS: Genau. Ähm. Weiss nicht ob sie mit dem ICD-10 noch arbeiten.

IP4: Ja. Aber eigentlich in der Grundversorgung nicht so sehr. Ich glaube die Psychiater machen immer alles mit dem. Wir haben schon lang, wir haben so viele Patienten wir haben gar nicht die Zeit jedes Mal zu schauen wo befinden wir uns jetzt.

MS: Das nur zur kurzen Information ist es einfach anders klassifiziert.

IP4: Okay.

MS: Wenn sie jetzt dieses Fallbeispiel, ähm, das wir jetzt besprochen haben noch mal anschauen. Was für Erfahrungen haben denn Sie in Ihrer Praxis mit Patientinnen, die solche Symptome zeigen. Gibt es das überhaupt? Oder -?

IP4: Ja, ich würde schon sagen, ja. Ich glaube es ist so wie mit vielen so genannten Psychopathologien. Es gibt nicht nur weiss und schwarz. Es gibt sehr viele Übergänge. Was ist normal und was ist pathologisch? Es gibt nicht nur das eine oder das andere. Sondern ich glaube es gibt sehr viele Nuancen auch dazwischen. Oder auch Lebensphasen wo man vielleicht, Beziehungsabbruch. Eine Frau die gut funktioniert, Beziehungsabbruch, der Freund hat sie verlassen und plötzlich kommen natürlich Selbstwertfragen, man beschäftigt sich dann wieder mehr mit dem Äusseren. Und es kann dann auch ein bisschen in eine Körperdysmorphe Störung gehen aber es ist vielleicht noch im Rahmen des Normalen. Ich denke ich würde jetzt nicht sagen es gibt nur schw- schwarz und weiss. Und es gibt auch sehr viele Übergangsformen. Aber ich denke, wenn es so einschränkt. Ich denke, dann soll man schon achtsam sein, dass man das wahrnimmt. Dass diese Person schwer, äh, eine schwere Störung hat. Und eingeschränkt ist in ihrem Lebenslauf und ihrem, äh, Lebensqualität und auch ja in der Lebensausführung, oder? Ja.

MS: Was sind denn die Herausforderungen mit solchen Patientinnen, Was würden Sie sagen? (.) Für Sie?

IP4: Ich denke, äh, das Vertrauen, mal die Beziehung zuerst bilden. Das Vertrauen. Oder? Das kommt nicht mit einer Sitzung. Da muss man, ähm. Dann wenn man mal das Vertrauen, die Beziehung hat, ich denke, dann kann man dann auch zusammen arbeiten. Ich sage bewusst zusammen. Weil ich denke es ist eben, ist immer auch ein miteinander ein Weg gehen. Und sei das im verhaltenstherapeutischen Kontext immer wieder kleine Expositionsversuche. Also ihr das einmal wieder das Selbstwertgefühl zu erarbeiten ich

denke das ist wichtig und dann immer wieder kleine Expositionen. Dass sie möglichst gesunden kann und ihr Leben, äh, ja so gestalten kann, dass es ihr wohl ist. Und dass sie zum Beispiel wieder an die Uni oder einen guten Abschluss, dass sie auch eine Beziehung aufnehmen kann, dass sie ihr Leben in allen Facetten leben kann.

MS: Sie haben das jetzt schon ein bisschen beschrieben. Wie ist Ihr professionelles Vorgehen um mit diesen Herausforderungen um zu gehen? Haben Sie da ein -

IP4: [So wie ich soeben geschildert habe.

MS: So wie Sie es eben geschildert haben ((lacht)).

IP4: Wichtig ist eben das Vertrauen. Vertrauen, die Beziehung und dann kann man zusammen arbeiten. Und ich habe nicht immer, ich habe nicht, klar hat man Konzepte im Kopf und Ideen, aber (.). Ich bi-, ich kann nicht, ich gehe immer individuell auf ein Individuum ein. Nicht nur jetzt mit diesen, äh, Körperdysmorphen Störungen sondern allgemein, oder. Es ist eine therapeu-, ein therapeutischen Weg für die eine Person muss nicht heissen, dass es für die andere Person stimmt. Es muss das Individuum, also jetzt die Frau, schauen wo steht sie. Was hat sie für Lebenserfahrung, was hat sie für Ressourcen, wo hin könnte der Weg gehen, und versuchen gemeinsam mit ihr zu ermutigen, kleine Schritte, kleine therapeutische Schritte zu machen. Bis zu einer Genesung. Und dass ist eine tolle Herausforderung für mich als Ärztin.

MS: Ja, ja.

IP4: Sind auch tolle Geschichten. Aber es gibt halt auch Geschichten wo man sieht man dreht und dreht und dreht am Ort. Und, und oft muss man natürlich auch, weil wir haben nicht Kapazität alles selber zu behandeln, wir nehmen sehr oft Rücksprache oder arbeiten zusammen mit Psychiaterinnen und Psychiater oder einfach Körpertherapeutinnen oder Therapeutinnen. Also es kann ganz vielseitig sein. Ja, wir haben auch eine Psychotherapeutin im Team.

MS: Also würden Sie so einen Fall selbst behandeln oder würden Sie weiter verweisen?

IP4: Ich denke da, weil ich nicht die Kapazität hätte sie jetzt so engmaschig zu sehen und weil ich denke sie braucht eine intensive Therapie und sie doch sehr eingeschränkt ist, würde ich sicher sie auch noch in, mit einem Netz, dass sie mehrere, sicher zwei also Thera-, auch noch eine Therapeutin hätte. Wahrscheinlich eine, am ehesten eine Therapeutin in diesem Kontext.

MS: Ja. Okay.

IP4: Die sie vielleicht einmal, zwei Mal die Woche sehen kann. Und ich sehen sie vielleicht dann einmal im Monat, maximal zwei Mal im Monat oder.

MS: Okay. Meine letzte Frage, die haben Sie zwar auch schon beantwortet, ich sag sie einfach noch schnell. Wie wäre Ihr professionelles Vorgehen jetzt ein, den Verdacht auf eine Körperdysmorphe Störung hätten?

IP4: Also ich denke zuerst immer einmal Diagnose stellen, oder. Was liegt genau vor und das braucht man wirklich im Gespräch vielleicht gewisse Untersuchungen auch. Und, ähm, dann mal eben schauen, mit ihr zu besprechen was liegt eigentlich genau zu vor. Das mal zu nennen, dass es auf dem Tisch ist, oder? Und ihr auf der, Patientin oder diese Frau dann auch auf zu zeigen was für therapeutischen Möglichkeiten es gibt.

MS: Mhm.

IP4: Ja, das wäre so mein Vorgehen. Und dann individuell dann schauen, welche therapeutischen Schritte ist sie gewillt zu gehen. Und will man einschlagen.

MS: Danke vielmal.

OD: Gut dann mache ich mal weiter. Ähm. Was sind Ihre Erfahrungen mit Patientinnen die Fragen zur Ästhetik oder zur Normalität ihres eigenen Genitalbereichs stellen?

IP4: (.) Meine Erfahrung. |**<< Meisten Frauen zufrieden**|Ich denke die meisten Frauen sind sehr zufrieden|*Meisten Frauen zufrieden >>*|, mit ihrem -, ist das, bezieht sich die Frage auf das sexuelle Äussere, oder? Ich denke die, nur auf das sexuelle Äussere? Das Äussere des Genitaltraktes, oder?

OD: Ja, genau.

IP4: |**<< Meisten Frauen zufrieden**|Ich denke die meisten Frauen sind sehr zufrieden.|*Meisten Frauen zufrieden >>*| Und es gibt aber auch dort die ganze Palette von ein bisschen unzufrieden bis zu extrem verunsichert und |**<< Labien**|das Gefühl die Labien sind zu gross oder da ist etwas nicht in Ordnung,|*Labien >>*| das stimmt nicht und es ist auch wieder ähnlich wie vorhin, äh, ich glaube da gibt es auch eine ganze Bandbreite und auch fliessende Übergänge von -. Aber die meisten, ich würde sagen ja. In meiner Sprechstunde wahrscheinlich über 90, 95 Prozent, zum Glück, sind zufrieden.

OD: Ähm, und wenn es doch solche Fragen kommen, wie gehen Sie dann damit vor?

IP4: Ähm, |**<< Anamnese**|ich versuche herauszufinden was ist es genau?|*Anamnese >>*| |**<< Bemerkungen**|Oft wen stört es eigentlich, also kommt das von ihr, kommt das manchmal von, hat die Mutter mal etwas gesagt in jüngeren Jahren oder sonst irgendeine Beziehungsperson, muss ja nicht nur die Mutter sein. Was hat sie für Erfahrungen gemacht. Kommt das allenfalls von einem Freund, von einem frühen Freund, von einem Partner, |*Bemerkungen >>*|ähm. |**<< Gespräch**|Ja, und was ist es genau, was stört? |**<< Miteinbeziehen**|Und dann schauen wir uns das zusammen an und besprechen es.|*Gespräch >>*| |*Miteinbeziehen >>*|Und ist es, ja.

OD: Ähm. Fragen Sie selber bei Ihren Patientinnen manchmal nach, wie sie die Ästhetik ihres Genitalbereichs empfinden?

IP4: |**<< Fragen nach**|Ja, ich frage vielleicht genereller, nicht nur nach Gen- sondern sind sie zufrieden mit ihrem Körper. Oder sind sie zufrieden mit ihrem Äusseren? Oder mit ihrem sexuellen, mit ihrem, Äusseren ihres Sexualorgans, so, ja. Das kann sein, wenn ich den Eindruck habe, es drängt sich auf. Aber ich frage nicht jede Frau ganz systematisch.

|*Fragen nach >>*|OD: Gut. Ähm. Was sind Ihre Erfahrungen mit Patientinnen die Schönheitsoperationen am Genitalbereich wünschen?

IP4: Hmmmmm. Ja. |**<< Verzerrte Wahrnehmung**|Da gehen manchmal die Ideen von mir und von den Patientinnen wirklich sehr weit auseinander. |*Verzerrte Wahrnehmung >>*|Ja. Ähm. |**<< Viele OP**|Ja, da gibt es, gibt es auch viele ganz verschiedene Geschichten die mir so durch den Kopf gehen|*Viele OP >>*|, oder. |**<< Direkt OP**|Und oft, manchmal kommen sie natürlich schon operiert. Das ist dann kann man es nur noch feststellen und vielleicht ansprechen, oder. |*Direkt OP >>*|Und man kann es nicht mehr rückgängig machen. |**<< Ernstnehmen**|Ich bin sicher sehr zurückhaltend und versuche die Frauen viel ab zu holen.|*Ernstnehmen >>*| Warum? Oder? |**<< spezifisches Beispiel**|Eine Frau zum Beispiel, die hatte nie ein Problem und |**<< Schwangerschaft/Geburt**|**<< Sexualität**|dann hat sie ein Kind bekommen und dann war die Sexualität nach der Geburt des Kindes, eheliche

Sexualität ein bisschen, ähm, lag brach.|*spezifisches Beispiel >>*|*Schwangerschaft/Geburt >>*|*Sexualität >>*| |**<< Bemerkungen**|Und dann plötzlich kam der Partner auf die Idee das wahrscheinlich am Äusseren des Genitales der Frau liegt. Und dann muss man natürlich sagen, ja hallo dort liegt es nicht. |*Bemerkungen >>*|Und herausfinden, dass es wohl eine Beziehungsstörung ist. Viel eher. Und natürlich auch die ganze Situation mit dem Kleinkind und die schon ein bisschen die Eltern überfordert und die Nächte und so fort. Wo brauchen sie wirklich Unterstützung und ich denke die |**<< Abratung von OP**|Operation des Genitales ist da ein Nebenschauplatz und ich habe versucht die Frau ab zu raten.|*Abratung von OP >>*| |**<< Gespräch**|Ob sie es dann nachher gemacht hat, oder nicht. Ich habe nicht gehört, dass sie es gemacht hat also ich hoffe unser Ge- unsere Gespräche haben sie überzeugt, dass sie vielleicht sich andere Hilfe holt als eine Sexualoperation.

|*Gespräch >>*|OD: Ja eben, das haben Sie jetzt schon ein bisschen beantwortet. Wenn jetzt so eine Frage kommt zu einer Schönheitsoperation wie gehen Sie dann normalerweise vor?

IP4: Also wenn konkret die Frage kommt: «Ich möchte eine Schönheitsoperation durchführen»?

OD: Mhm, also am Genitalbereich.

IP4: |**<< Gespräch**|Also zu erst wieder das Gespräch.|*Gespräch >>*| |**<< Anamnese**|Herauszufinden, wie woher, was liegt vor? Ähm, auch vielleicht wichtig was wir nicht angesprochen haben, die Geschichte der Frau. |**<< Traumatische Erfahrungen**|Oder vielleicht traumatische Geschichten, vielleicht liegt etwas Traumatisches vor und ist das äussere Genitale nur ein Nebenschauplatz. |*Anamnese >>*|*Traumatische Erfahrungen >>*|**<< Untersuchung**|Und, ähm, dann denk ich eine Untersuchung|*Untersuchung >>*|, |**<< Visuell**|vielleicht auch mit dem Spiegel. Ich habe ein Spiegel, dass sich die Frau auch mal selber erkunden kann und anschauen kann|*Visuell >>*| und |**<< Normalität betonen**|ich denke auch wichtig die, äh, ein Feedback, dass die Frau, dass ihr Genitale gesund ist. Wir machen sicher eine Abklärung, Untersuchung. Äh, je nach dem was ansteht. Und dass sie etwas gesund ist, oder? Und dass das äussere Genitale, äh, normal ist. |*Normalität betonen >>*|**<< Selbst behandeln**|Und auch meistens denk ich braucht sie dann auch mal Zeit um das mal zu überdenken und mal schauen, dass man sich wieder trifft und schaut wo geht man jetzt weiter|*Selbst behandeln >>*| und, eh, ist das Problem für sie, hat sich das gelöst? |**<< Medien**|Waren das Zweifel die sich irgend-, vielleicht auch durch Medien, weil heute ist so viel in den Medien, ist das, sind das Zweifel, die jetzt weg sind.|*Medien >>*| Oder liegt noch viel-, in der Tiefe, liegen weiter Konflikte vor?

OD: Gut. Dann habe ich auch noch ein zweites Fallbeispiel dabei. Können Sie das mal durchlesen.

[OD legt Fallvignette 2 hin. IP4 liest Fallvignette 2 durch.]

IP4: Mhm, okay.

OD: Also das ist jetzt ein Beispiel einer Körperdysmorphen Störung des weiblichen Genitalbereichs. Also es kann jedes Körperteil betroffen sein der Körperdysmorphen Störung und hier geht es eben um den Genitalbereich. Und jetzt ist meine Frage, was sind Ihre Erfahrungen mit Patientinnen die solche Symptome zeigen?

IP4: |**<< Gespräch**|Also ich würde jetzt die Mia wenn sie jetzt kommt mit diesen Sorgen würde ich mal mit ihr sprechen.|*Gespräch >>*| |**<< Anamnese**|Und eben eine wie wir gesagt haben ein bisschen Anamnese oder, persönlich, ein bisschen ihre Geschichte erfahren. |*Anamnese >>*|**<< Untersuchung**|Und dann würde ich sie sicher untersuchen und dann, ähm, ja, |**<< Gespräch**|würden wir, müssten wir ins Gespräch kommen.|*Gespräch >>*| Ähm, ich müsste es auch anschauen oder. |*Untersuchung >>*|Äh und |**<< Normalität betonen**|ich

würde versuchen sie natürlich zu ermuntern oder ein Feedback zu geben, dass, äh, dass sie ein normales Genitale hat, oder.|*Normalität betonen >>*| |**<< Anamnese**|**<< Selbst behandeln**|Aber versuchen herauszufinden wie sie darauf kommt, oder. Und was dann so, ich mein sie lebt in einer Beziehung, oder.|*Anamnese >>*| |**<< Scham**|Und sie hat wohl ein gewissen, eine Einschränkung, ja sie schämt sich im, um zu ziehen, irgendwelche Garderoben. |*Scham >>*|**<< Exponieren**|Aber ich würde schauen wie kann sie vielleicht, am doch langsam sich ein bisschen exponieren,|*Exponieren >>*| kann sie doch, |**<< Strategien**|vielleicht gibt es gewisse Techniken sich umziehen, damit sie trotzdem zum Beispiel zum Sport oder zum Schwimmen gehen kann. Und so ein bisschen versuchen sie, äh, schauen dass sie nicht eingeschränkt ist in ihren Aktivitäten. Weil ich denke die Störung können wir nicht mit einem Gespräch aufheben, oder. Aber wie können wir ihr ein paar Tipps mitgeben, dass sie sich nicht eingeschränkt fühlt, |*Strategien >>*|weil sie scheint ja sonst, eben sie lebt in einer Beziehung. |**<< Beziehung**|Aber sie belastet natürlich auch die Beziehung mit ihrer Unsicherheit. Und ihrem Rückfragen und das müssten wir auch zusammen anschauen, das wäre sicher dann ein weiterer Schritt noch. Eine weitere Ebene oder, zuerst mal zu ihr und dann eben auch ihr zu Spiegeln was das natürlich in der Beziehung dann auslöst. Wie könnte sie vielleicht dort, ähm, ja, umgehen. Ja.

|*Beziehung >>*|*Selbst behandeln >>*|OD: Und haben Sie in echt jetzt auch schon solche Patientinnen erlebt?

IP4: |**<< Seltene KDS**|Ja, ja, mhm. Gibt’s. Aber eben nicht, ich würde sagen jetzt von vielleicht hundert Frauen die ich untersuche, |**<< 5 Prozent**|es sind wenige Prozente, also vielleicht fünf Prozent nur jetzt wirklich nicht einmal, nicht einmal. Ich würde sagen maximal fünf Prozent, also das wären fünf Frauen von hundert. Die wirklich dann schwer leiden. |*Seltene KDS >>*|*5 Prozent >>*|Ja. Mhm.

OD: Gut. Dann kommen wir noch zu ein paar Fragen zu so Fortbildungen und so.

IP4: Jetzt hab’ ich es nicht verstanden, zu?

OD: Fortbildungen.

IP4: Fortbildung, ja. Gut ((lacht)).

OD: Also wie gross schätzen Sie das Interesse zur Körperdysmorphen Störung im gynäkologischen Fachbereich ein?

IP4: Unter Ärztinnen?

OD: Ja.

IP4: Unter Ärzten?

OD: Ja.

IP4: |**<< Grosses Interesse an Fortbildung**|Ja ich denke schon, dass das Interesse gross ist. Vor allem eben wenn man, äh, Gynäkologie macht, Sexualmedizin, Psychosomatik, also ich meine in den Kreisen wo ich mich bewege. Dann bildet man sich natürlich auch auf diesen Gebieten fort. Also ich denke das ist schon da. Und ist auch ein Thema. Ja.

|*Grosses Interesse an Fortbildung >>*|OD: Und wie gross schätzen Sie das Interesse oder den Nutzen von Hilfsmittel zur Diagnostik ein, also so Screening-Instrumente oder so? Gibt’s da Bedarf?

IP4: Screening, also so Fragebogen?

OD: Ja, genau.

IP4:|**<< Wenig Interesse an Diagnostik**| Ja ich bin nicht unbedingt die eben da so sehr nach Sc-, nach irgendwelchen, äh, wie soll ich sagen, so Guidelines geht. |*Wenig Interesse an Diagnostik >>*|**<< Unpraktisch**|Ich denke die Medizin, es ist toll Guidelines, mal zu sehen oder mal zu lesen aber im Praxisalltag, es ist doch jede-. Jeder Mensch ist ein Individuum. Man hat schon, hat Erfahrung und lernt, oder? Aber ich weiss nicht, ob jetzt ein ähm|*Unpraktisch >>*| (.). Vielleicht im Rahmen einer Fortbildung, ja dass man das vielleicht vorstellt und was es gibt dann pickt sich jede Ärztin, jeder Arzt was er gerne hat in seinem Alltag, seinem Praxisalltag.

OD: Ähm. Wie gross schätzen Sie das Interesse an Hilfsmittel für Beratung von Patientinnen jetzt spezifisch mit Körperdysmorphen Störung des Genitalbereichs ein? Also es gibt zum Beispiel so Tafeln wo einfach verschieden Genitalien abgebildet sind oder so Sachen.

IP4: Mhm, mhm, dass man zum Beispiel so Bildmaterial hat und der Frau das zeigen kann was ist noch normal und so?

OD: Ja, genau.

IP4: (2). |**<< Kein Interesse an Hilfsmittel**|Haben wir nicht zu viel Bilder schon? ((lacht)). Ich denke es muss auch jede Person selber wissen, ich habe in gewissen Gebieten arbeite ich auch gerne mit Bildmaterial. Aber ich weiss jetzt nicht ob ich jetzt wirklich da (.) Bildmaterial möchte. |*Kein Interesse an Hilfsmittel >>*|Hab' ich mir gar noch nicht überlegt. Bisher habe ich mit dem Spiegel und mit der Person gearbeitet. Wirklich zugeschnitten auf die Person, und nicht mit anderen Bildern noch. Weil ich denke wir sind schon so voll im Kopf mit Bildern den ganzen Tag. Also das ist meine persönliche Meinung. Aber das kann sich auch, vielleicht nach der nächsten Fortbildung ((lacht)). Kann ich das doch noch einbauen, das Bildmaterial auf diesem Sektor ((lacht)).

OD: Gut dann wären wir eigentlich schon am Ende ausser Sie möchten noch etwas hinzufügen?

IP4: Nein, es ist gut so für mich.

OD: Gut, danke.

IP4: Bitte sehr.

Interview mit: IP5
Datum des Interviews: 16.05.2019
Dauer: 22 min
Anonymisierte Begriffe: keine
IP-Nummer: 5

MS: Dann starten wir. Und zwar ist die erste Frage mit welchen psychischen Problemen kommen denn Patientinnen zu Ihnen in die Praxis?

IP5: (6) |**<< Reaktionen auf schwere Belastungen und Anpassungsstörungen (F43)**|Also, es gibt äh, aktuell habe ich eine Patientin mit einer Anpassungsstörung.|*Reaktionen auf schwere Belastungen und Anpassungsstörungen (F43) >>*| Ähm, mit einer Schwangerschaft, wo sie von dem, wo sie sich vom Partner getrennt hat. Das ist ein Beispiel. Ähm (3). Also sie kommen weniger mit psychischen Beschwerden in meine Praxis eigentlich. Ja, sie suchen mich nicht deshalb aus. Sondern es stellt sich manchmal eine psychische Erkrankung heraus und, ähm, ja.

MS: Was wären das für, wüssten Sie dafür ein Beispiel.

IP5: |**<< Restkategorie Störungen**|Es gibt Frauen die in einer Lebenssituation sind, die vielleicht eine Erschöpfung haben|*Restkategorie Störungen >>*|, |**<< Depressive Episode (F32) und rezidivierende depressive Störung (F33)**|eine Depression|*Depressive Episode (F32) und rezidivierende depressive Störung (F33) >>*| |**<< Phobische Störung (F40) und andere Angststörungen (F41)**|oder eine Angststörung, die sie immer mehr, ähm herausfordert oder, oder betrifft und dann versuche ich, äh, sie weiter zu leiten an eine geeignete Stelle wo man sie, halt helfen kann.

|*Phobische Störung (F40) und andere Angststörungen (F41) >>*|MS: Ok. Ähm, welche Rolle spielt denn das äussere Erscheinungsbild bei Ihren Patientinnen? Die Zufriedenheit, also das Aussehen.

IP5: Also, meine Zufriedenheit?

MS: Nein, nein, von den Patientinnen. Also im Sinn von, ist das ein Thema bei Ihnen, sprechen Sie darüber? Kommen Frauen zu Ihnen und fragen Sie etwas?

IP5: Ich verstehe die Frage nicht ganz, aber also (2). Nein also ich verstehe die Frage nicht. Also die Frauen kommen zu mir, und -?

MS: Ist die Zufriedenheit mit dem Aussehen ein Thema, dass Sie anbringen?

IP5: Also es gibt Frauen, die sind nicht zufrieden mit ihrem Aussehen, ja. Und die würden dann, die sagen dann auch, dass sie mit gewissen Körperteilen vielleicht nicht so glücklich sind, oder dass sie finden, subjektiv, dass sie, dass es nicht gut ausschaut, aber ähm, das schon ja. Das kommt vor.

MS: Und welche Körperteile sind dann betroffen?

IP5: Ähm, dass ähm, sicher die Brust. Dass die Brust zu klein ist oder dass die Brust, ähm, zu stark, ähm, hängt oder dass es, äh, ja so etwas. Oder dass die Form nicht passt oder. Ähm zu gross gibt es ja auch. Dass sie auch Beschwerden haben deshalb.|**<< Labien erwähnt**| Dann gibt es auch, vom äusseren Genitale, dass, dass sie (.) die Schamlippen als zu gross oder zu prominent empfinden|*Labien erwähnt >>*| und dort sich nicht wohl fühlen oder das Gefühl haben, dass das vielleicht dem Partner oder der Partnerin nicht gefällt. Genau.

MS: Okay. Ich habe jetzt hier eine Fallvignette. Ich würde Sie bitten, diese kurz durch zu lesen.

[MS legt Fallvignette 1 hin. IP5 liest Fallvignette 1 durch.]

IP5: Ja.

MS: Wenn jetzt so eine Patientin zu Ihnen kommen würde, was würde Ihnen auffallen, oder - ?

IP5: Was würde mir auffallen?

MS: Ja.

IP5: Also (4). Das ist natürlich immer ein, ähm, eine, eine Frage des Betrachters. Was ist schön oder was ist nicht schön oder was-. Es gibt Menschen die stören sich an eben, an Sachen die sind gar nicht, die entsprechen sogar dem Ideal. Am Idealbild. Und andere Personen sehen das ganze anders als sie selbst. Also das ist genau der Fall eben, was ich Ihnen gesagt habe. Ich glaube da kann man, muss man schauen muss man explorieren, muss man schauen wie schlimm, also das scheint jetzt doch eine, äh, sehr einschränkende Geschichte zu sein. Da muss, da muss, da muss man sicher, äh, mit ihr das anschauen. Also man kann sicher bekräftigen, dass, wenn es so ist, dass es normal ausschaut und dass es alters entsprechend ist und dass, dass -. Aber das ist vielleicht, reicht das, führt da die Frau nicht raus, dass sie beruhigt ist, sie braucht etwas anderes ja. Sie braucht eine Therapie wahrscheinlich.

MS: Ja, die nächste Frage wäre, welche Informationen würden Sie sich noch wünschen von dieser Patientin (1), was würden Sie noch fragen?

IP5: (6). Also ich würde mich da nicht zu, ich würde das nicht zu stark ähm, explorieren bei ihr. |**<< Begründung**|Weil ich es jetzt da, ich sehe es nicht als meine Aufgabe, wenn sie das so, dann geht, dann ist es schon ein bisschen pathologisch, wenn sie das so anschaut.|*Begründung >>*| Dann würde ich sie weiterschicken an eine geeignete Stelle, an eine Psychologin oder, oder, dass sie das vielleicht aufarbeitet.

MS: Ich habe eine kleine Auflösung also das wäre, das Fallbeispiel wäre eine (4) wäre ein Beispiel einer Körperdysmorphen Störung, kennen, haben Sie davon schon mal gehört?

IP5: |**<< KDS bekannt**|Habe ich auch schon gehört.

|*KDS bekannt >>*|MS: Ich habe hier noch kurz die Kriterien. [Zeigt DSM-5 Kriterien]. Eben ist eine übermässige Beschäftigung, ist mit einem Makel der für Andere nicht sichtbar ist. Ähm, dann haben sie stundenlange, sich wiederholende Verhaltensweisen, wie vor dem Spiegel überprüfen, sie vergleichen sich ständig mit Anderen, rückversichern sich bei den Anderen: «Sieht das normal aus? ». Dann eben ganz wichtig eine hohe Beeinträchtigung und ein Leidensdruck. Sozial, beruflich aber auch in anderen Gebieten. Und ähm, es darf sich nicht um eine Essstörung handeln dabei. Das wären so die Kriterien.

IP5: Gut, habe ich etwas gelernt.

MS: Dann ist meine Frage, wenn wir jetzt das Fallbeispiel noch mal anschauen. Haben Sie denn Erfahrungen mit solchen Patientinnen, haben Sie das schon mal erlebt, oder kommt das eher nicht vor?

IP5: (2). Also so ausgeprägt würde ich sagen ist es nicht, erinner’ ich nicht, dass ich das -. Es gibt natürlich, es ist fliessend, jeder hat irgendwie, findet, hat irgendein Körperteil das er

nicht so mag oder hat das Gefühl das etwas nicht so gut ist, was andere nicht so empfinden. Das ist bis zu einem gewissen Grad auch normal. Aber das ist natürlich sehr einschränkend [spricht Fallbeispiel an]. Sie wird sich zunehmend isolieren und es wird immer schlimmer wahrscheinlich, könnt ich mir vorstellen. Oder so. Nein, sehe ich nicht so häufig ist nicht so, dass jetzt, äh, dass ich das jede Woche einmal sehe.

MS: Okay. Und wenn mal so eine Patientin bei Ihnen ist, was sind denn die Herausforderungen im Kontakt mit so einer Patientin?

IP5: (2). Also ich würde, also zum Beispiel wenn wir jetzt wieder zurück gehen zu irgendwelchen, wenn sie sagt ihre Schamlippen sind zu gross oder sie sind gar nicht oder so. Dann würde ich sie eher, ähm, davon stark abraten, dass sie sich da irgendwelche, ähm, Operationen, ähm, Operationen macht um das zu korrigieren es gibt, dann sollte man das nicht machen, das wäre der falsche Weg. Dann ähm so etwas wird sie nicht glücklich machen. Das ist kein Grund für eine Operation zum Beispiel. Aber es gibt natürlich dann wieder Ärzte oder Institutionen die das vielleicht machen weil, ja. Also da muss man sie ein bisschen schützen, dass man einen anderen Weg einschlägt.

MS: Also eine Herausforderung ist, dass sie dann eine OP wünschen und Sie sie abbringen müssen.

IP5: Vielleicht, genau. Also das gibt es immer wieder. Und, und wenn man das nicht macht dann gehen sie zum nächsten bis, bis einer ähm das macht für, für Geld, oder? Weil es geht dann am Schluss um, wenn der Arzt oder so, dass ist dann verlockend dass man sagt ich mach es dann kann ich etwas verdienen. Aber da muss man vielleicht ein wenig stark sein, beziehungsweise sollte das im Sinne der Frau sollte man das ablehnen oder sagen dass es nicht der richtige Weg ist das (.) eine falsche, dass sie irgendwie falsche eingestellt ist auf ihren Körper, genau.

MS: Ähm, Sie haben es jetzt schon eigentlich angetönt. Wenn Sie jetzt eine Patientin antreffen in Ihrer Praxis die jetzt solche Symptome zeigen würde, ähm wie wäre Ihr Vorgehen?

IP5:(3) Ich würde sie ähm empfehlen, dass sie sich (2) dass sie, dass sie sich mit einer Psychologin oder mit einem Psychologen bespricht, dass sie versucht, dass man das angeht, dass man schaut wo ist, wo ist, was kann man machen, dass man sich ein bisschen frei, befreit. Das ist meistens eben subjektiv, die denkt es ist wirklich so. Dass man sie versucht da zu challengen und raus zu nehmen aus dieser Anschauungsweise. Das wäre mein Vorschlag.

MS: Gut, danke. Das wäre mein Teil schon gewesen.

OD: Gut dann übernehme ich jetzt. Sie haben das schon ein bisschen angesprochen. Was sind Ihre Erfahrungen mit Patientinnen, die Fragen zur Ästhetik oder zur Normalität ihres eigenen Genitalbereichs stellen?

IP5: (3) Was sind meine Erfahrungen? Also das ist natürlich etwas eben was, was. |**<< Rollenbild**|Also Frauen haben wir ja in der Gesellschaft oder, oder in der -. Die Rollenverteilung, die Frau hat, sie muss gefallen und das ist irgendwie etwas uraltes, sie machen sich schön.|*Rollenbild >>*| Und dann, Und dann ist natürlich immer die Frage, bin ich hübsch genug und das ist etwas was, diese Rolle ist etwas bei ganz jungen, bei mittel jungen und bei älteren Frauen allgegenwärtig und das ist immer wieder etwas was sie auch Bestätigung zum Teil suchen. Oder, oder fragen oder sich beklagen. |**<< Viele Fragen** |Also das ist schon ein Thema, also immer und immer wieder. Es gibt auch, natürlich gibt’s auch Frauen die sind da relativ entspannt aber ich würde sagen jede Frau macht sich Gedanken in dieser Hinsicht. Das ist etwas was tief verankert ist.

|*Viele Fragen >>*|OD: Und wie ist ihr professionelles Vorgehen zu diesen Fragen. Also jetzt spezifisch zum Genitalbereich?

IP5: Also die (2), |**<< Vielfalt**|**<< Normalität betonen**|**<< Expertise nutzen**|also ich habe schon sehr viel gesehen|*Expertise nutzen >>*| und es gibt eine extrem grosse Bandbreite was normal ist und was, was gut ist. Oder. Und normal ist was, was ja. |*Vielfalt >>*|*Normalität betonen >>*|**<< Untersuchung**|Und dann geht's eigentlich darum heraus zu finden ob es tatsächlich medizinisch oder, oder |**<< Medizinische Beschwerden**|Beschwerden gibt für, für eh, es kann schon sein dass eh, |**<< Labien**|wenn die Schamlippen gross sind,|*Labien >>*| dass es dann Beschwerden gibt. Wenn sie, wenn die Frau sportlich zum Beispiel sehr aktiv ist. Beim Fahrradfahren. |**<< Sexualität**|Beim Geschlechtsverkehr kann es zum Teil auch störend sein. |*Sexualität >>*|Ist durchaus nachvollziehbar und einleuchtend. Da muss man schauen, ist es etwas was rein ästhetisch oder etwas was das empfinden stört. Oder ist es tatsächlich ein medizinisches Problem und dem entsprechend dann auch die Beratung. Ja.

|*Untersuchung >>*|*Medizinische Beschwerden >>*|OD: Fragen Sie manchmal bei Ihren Patientinnen nach wie sie zur Ästhetik ihres Genitalbereichs stehen.

IP5: |**<< Fragen nicht nach**|Nein.

|*Fragen nicht nach >>*|OD: Ähm,|**<< Erwähnen OP**| das haben Sie auch schon ein bisschen angesprochen. Was sind Ihre Erfahrungen mit Patientinnen die Schönheitsoperationen am weiblichen Genitalbereich wünschen?

|*Erwähnen OP >>*|IP5: (3) Was sind meine Erfahrungen? (.) In, also (2), also das ist äh, das ist eine offene Frage ((lacht)).

OD: Genau.

IP5: |**<< Einige OP**|Also gibt es, ja (.) Da kann ich nur beispielhaft sagen.|*Einige OP >>*| |**<< Komplikationen** |Also es gibt Erfahrungen die sind bedauerlich, dass, dass (.) |**<< Gelder**|Frauen vielleicht sogar ins Ausland gehen um dort günstige Operationen bekommen |*Gelder >>*|und und die dann am Schluss fast verstümmelt sind, dass sie |**<< Labien**|die Schamlippen fast ganz weghaben und dann kann man es tatsächlich ein Problem werden.|*Labien >>*| |*Komplikationen >>*|**<< Sinnvoll**|Es gibt aber auch sehr schöne, ähm, |**<< Brüste**|sehr schöne Operationen also sehr schöne Brust-Operationen ähm, die man sagen muss es sieht, sieht wirklich perfekt aus, und, und äh es ist gut. |*Brüste >>*|Also (.) das ist nicht unbedingt, ich würde das nicht einfach nur verteufeln oder schlecht machen es gibt sicher auch sehr, sehr gute ähm, gute Operationen. Und sinnvolle Operationen wenn jemand sehr stark unter einer zu kleinen Brust leidet oder einer nicht vorhandenen Brust und man einen Aufbau macht mit einer, |**<< Zufriedenheit**|*Sinnvoll >>*|mit einem Implantat kann das auch für die Frau positive auswirken für die Psyche, dass sie dann sich besser fühlt und und äh attraktiver und das gibt dann wieder Feedback für die Psyche das, das ja, finde ich gut. |*Zufriedenheit >>*|**<< Anamnese**|Man muss es im Detail anschauen was das Problem ist und wie sinnvoll das eine Massnahme ist.

|*Anamnese >>*|OD: Gut. Ja das haben Sie jetzt auch schon ein bisschen gesagt. Was ist dann Ihr professionelles Vorgehen, wenn Fragen zu Schönheitsoperationen kommen?

IP5: |**<< Individualität**|Also man muss einfach das individuell betrachten |*Individualität >>*|und, und |**<< Anamnese**|die Lebenssituation anschauen. |*Anamnese >>*|Ähm, |**<< Jüngere**|das Alter ist sicher auch entscheidend, dass nicht ganz junge, dass man das ein bisschen anschaut |*Jüngere >>*|und dann, dass man sie dann auch an die richtigen Kollegen weiterleitet die das, die das äh machen. |**<< Brüste**|Gerade Brustaufbau zum

Beispiel,|*Brüste >>*| |**<< Abneigung Chirurgie**|**<< Überweisung an Chirurgie**|dass man sie an ein seriöses, an einen seriösen Kollegen schickt, der äh nicht, sie gut berät. Also dass es nicht, ähm vielleicht muss man sie auch schützen, dass man nicht zu grosse Implantate macht, das kann ja auch negativ sein dann.

|*Abneigung Chirurgie >>*|*Überweisung an Chirurgie >>*|OD: Gut. Ich habe jetzt auch noch ein Fallbeispiel dabei. Wenn Sie das durchlesen könnten.

[OD legt Fallvignette 2 hin. IP5 liest Fallvignette 2 durch.]

IP5: Mhm.

OD: Ähm, das wäre jetzt ein Beispiel einer Körperdysmorphen Störung spezifisch vom Genitalbereich. Also es kann jedes Körperteil betroffen sein und hier würde es um den Genitalbereich gehen. Jetzt ist meine Frage, was sind Ihre Erfahrungen mit Patientinnen, die solche Symptome zeigen?

IP5: (3) |**<< Untersuchung**|Also wie gesagt man muss es anschauen. Also scheinbar ähm, scheint es scheinbar ist es normal die, die Schamlippen sind normal geformt und normal gross aber sicher muss man es anschauen wo, ob es jetzt wirklich so, so ist.|*Untersuchung >>*| Und dann müssen sie überlegen, was für die Patientin am besten ist.|**<< Überweisung an Chirurgie**| Ob es allenfalls eine kleine Korrektur ist, es kann ja auch sein, dass es wirklich äh mit einer kleinen Operation dann auch getan ist. |*Überweisung an Chirurgie >>*|**<< Simpel**|Schamlippen sind ja Hautfalten die kann man auch gut operieren. Sollte eigentlich kein Problem sein, es ist keine grosse Geschichte|*Simpel >>*| und |**<< Sinnvoll**|vielleicht profitiert sie auch wenn man ein bisschen reseziert, wenn man ein bisschen wegnimmt |*Sinnvoll >>*|und dann, dass sie das äh, ja. Ob es das ist. |**<< Psychische Probleme im Hintergrund**|Ob sie dann nachher etwas anderes hat oder vielleicht ist es wirklich, keine Ahnung müsste man anschauen.

|*Psychische Probleme im Hintergrund >>*|OD: Und ähm, also haben Sie auch schon solche Patientinnen angetroffen, kommt das manchmal vor bei Ihnen?

IP5: Ja das gibt es. Das gibt es schon.

OD: Können Sie ein bisschen eine Schätzung geben wie oft das vorkommt?

IP5: Wie häufig ich so etwas sehe. (.) |**<< Monatlich**|Vielleicht drei Mal pro Monat.|*Monatlich >>*| So. |**<< Alter**|Also sind dann vielleicht nicht einundzwanzig jährig. Ich würde sagen, dass gibt es auch bei ähm etwas reiferen Frauen, dass sie das, das sie sich darüber stören. Ja.

|*Alter >>*|OD: Gut. Ähm. Dann sind wir schon bald am Ende. Wie gross schätzen Sie das Interesse an einer Fortbildung jetzt zu Körperdysmorphen Störung in Ihrem Fachbereich ein?

IP5: (3) |**<< Mittel Interesse an Fortbildung**|Es ist sicher eine interessante Frage. Weil es, weil es gibt schon, gibt es schon. |**<< Verzerrte Wahrnehmung**|Wird schon wirklich immer wieder angetroffen, dass man es nicht nachvollziehen kann. |*Verzerrte Wahrnehmung >>*|Machen Sie eine Fortbildung?

|*Mittel Interesse an Fortbildung >>*|OD: Nein ((lacht)). Ist auch eine offene Frage. Wie gross schätzen Sie das Interesse oder den Nutzen and Hilfsmittel zur Diagnostik von Körperdysmorphen Störung ein? Also zum Beispiel in Form von Screening-Instrumenten ein?

IP5: (3). |**<< unnötig**|**<< Wenig Interesse an Diagnostik**|Also ich finde das jetzt nicht so eine schwierige Diagnose. Also ist natürlich eben graduell und, und muss man. Ich glaube

nicht, dass ich das brauche, weil man merkt ja, dass, man sieht ja die Kongruenz.|*unnötig >>*| Also ich würde sagen, gut (2), ich sehe natürlich nur eine. Die Person sehe ich nur für einen kurzen Moment, 20 Minuten, viertel Stunde, halbe Stunde. Dass ich dann das auf den Punkt bringe, dass ich das erkenne und sehe. Aber wenn das ein Thema ist und man das miteinander bespricht, dann würde ich sagen kann man das schon herausfinden. Dass es da, dass es ein Problem ist.

|*Wenig Interesse an Diagnostik >>*|OD: Ähm, wie gross schätzen Sie das Interesse an Hilfsmittel für die Beratung von Patientinnen. Jetzt spezifisch mit Körperdysmorphen Störung der, des Genitalbereichs ein?

IP5: |**<< Mittel Interesse an Hilfsmittel**|Also ist definitiv interessant.|*Mittel Interesse an Hilfsmittel >>*| |**<< praktisch**|**<< Bewusstsein**|Also das andere auch, das Screening natürlich ist interessant, dass man etwas ähm vielleicht auch dass ein bisschen mehr, dass man das ein bisschen kennt und auch ein bisschen die speziellen Punkte schneller erfahren kann, mit einem Hilfsmittel.|*praktisch >>*|*Bewusstsein >>*| Und auch dass auch, wie man schreibt allenfalls mit ihr besprechen kann

OD: Gut dann sind wir eigentlich fertig. Oder möchten Sie noch etwas hinzufügen?

IP5: Nein, ist gut ((lacht)).

Interview mit: IP6
Datum des Interviews: 16.05.2019
Dauer: 40 min
Anonymisierte Begriffe: keine
IP-Nummer: 6

MS: So, gut, dann können wir starten. Ich habe die Fragen einfach hier, damit ich nichts vergesse.

IP6: Gut.

MS: Ähm, die erste Frage wäre, ähm, also jetzt bezogen auf Ihre gesamte gynäkologische Erfahrung, ähm, mit was für psychischen Problemen kommen denn die Frauen zu Ihnen?

IP6: Also das teilt sich eigentlich in zwei Bereiche auf. |**<< Restkategorie Störungen**|Das eine ist natürlich die Frage Körperbild,|*Restkategorie Störungen >>*| das heisst, dass die Frauen entweder mit ihrer Brustform, Brustgrösse |**<< Labien erwähnt**|nicht so zufrieden sind oder im Genitalbereich,|*Labien erwähnt >>*| ähm, eben ein Schönheitsideal haben, dass die Schamlippen zu gross o- ja eben meistens eher zu gross sind, und sie dann eine bestimmte Vorstellung haben, wie sie das verändern wollen. |**<< Restkategorie Störungen**|Und wenn man das mehr auf den inneren Bereich bezieht, ist es halt häufig Probleme in der Sexualität. Und die sind natürlich sehr breit gestreut. Sei es, dass sie Probleme haben mit der Sexualität, weil sie vielleicht Medikamente nehmen müssen, die die Sexualität verändern. Da ist zum Beispiel ein Bereich, ähm, dass manche Frauen ja ‘ne verminderte Libido haben, weil sie ‘ne Pille nehmen und mit der Fragestellung kommen. Manche wenn sie Krankheiten haben wie beispielsweise eine Endometriose, das ist auch häufig mit Schmerzen im Unterleib verbunden, dass sie dann sagen, sie haben da Schmerzen oder sind auch ‘n Stück weit traumatisiert, weil sie noch nie ‘ne wirklich schöne Sexualität erlebt haben und darunter leiden, weil sie ja mit der Vorstellung einig ist, dass Sexualität was Schönes ist und sie es aber so nicht erleben können.|*Restkategorie Störungen >>*| Ähm, ein anderer Bereich ist natürlich so diese Kinderwunsch Frage, wenn ein unerfüllter Kinderwunsch da ist, dass das natürlich psychisch sehr belastend sein kann, weil das ja dann immer die Frage des Ursprungs ist, ist tatsächlich so, dass ‘ne verminderte Anzahl an Eizellen da ist, man schwieriger schwanger wird oder liegt es an Gründen, die man gar nicht definieren kann. Es gibt ja auch Frauen wo man es wirklich nicht rausfindet, warum sie nicht schwanger werden, obwohl sie den ganzen Abklärungsbereich haben. |**<< Reaktionen auf schwere Belastungen und Anpassungsstörungen (F43)**|Und wenn man in den onkologischen Bereich geht ist natürlich die Belastungssituation während der onkologischen Diagnostik und Therapie und natürlich immer die Frage nach Leben und Tod.|*Reaktionen auf schwere Belastungen und Anpassungsstörungen (F43) >>*| Das sind so die ganz grossen Hauptbereiche, wo sich natürlich stark sich in der Psyche widerspiegeln kann.

MS: Okay. Sie haben es zu Beginn schon angesprochen, welche Rolle spielt denn die Zufriedenheit mit dem äusseren Erscheinungsbild bei Ihren Patientinnen?

IP6: Es hat schon einen grossen Einfluss. Also man merkt, dass manche Probleme erst dadurch entstehen, dass sie mit dem Körperbild nicht zufrieden sind und das sich natürlich stark auswirkt, wiederum zum Beispiel auf die Sexualität und dass da manchmal so Teufelskreise sind, die entstehen. Dass man gehemmter ist deswegen, sich nicht so verhalten kann, nicht so kleiden kann wie man das möchte, dass wir dann Rückspiegelung auf Selbstwertgefühl, dass wenn man kein Selbstwertgefühl hat oder ein schlechteres Selbstwertgefühl hat, das natürlich sich wieder in der Kommunikation und Interaktion mit der Um- dem Umfeld widerspiegelt und verschlechtern kann. Also von daher glaube ich, dass das schon (2) meistens nicht auf eine einzige Sache zu beschränken ist, sondern das ist

halt, das sind Dinge, die stark ineinander sch- greifen und dann das eine das andere verschlechtern kann und eben anders herum dann auch wieder.

MS: Ja. Also sprechen Sie da öfters drüber mit -?

IP6: Wir sind natürlich da auch starke Ansprechpersonen, weil ich mein das ist, weibliches Körperbild hat sehr viel mit Gynäkologie zu tun und ähm, da ist die Hürde manchmal niedriger als wenn man jetzt zum Hausarzt geht wo man sagt, dass ist der Allgemeinmediziner und wenn ich den jetzt auf Körperbild anspreche, dann kann der damit nix anfangen, während da die Hemmschwelle bei uns natürlich niedriger ist, weil wir schauen uns die Brust an, wir schauen uns das äussere Genital an und da ist es viel näher, dann auch über die Themen zu sprechen. Und viele Fra- Gynäkologen oder Gynäkologinnen sind ja eben auch dann auch Frauen und dann ist die Hemmschwelle auch ein bisschen niedriger, also ich glaub bei Männern ist die Hemmschwelle da schon noch höher, sowas anzusprechen als jetzt bei uns Frauen.

MS: Mhm, okay. Ich habe jetzt eine Fallvignette dabei und würde Sie bitten, diese kurz durchzulesen.

[MS legt Fallvignette 1 hin. IP6 liest Fallvignette 1 durch.]

IP6: Mhm.

MS: Ähm, was für eine psychische Störung oder Probleme fällt Ihnen bei diesem Fallbeispiel ein?

IP6: |**<< Begründung**|Das ist natürlich als Gynäkologin schwierig, das in einem psychologischen Fachdeutsch erklären|*Begründung >>*| ((lacht)). |**<< vermindertes Selbstwertgefühl und Selbstvertrauen**|Aber ich glaub das fällt schon auf diese Körperbild, Selbstwertgefühl, in den Bereich|*vermindertes Selbstwertgefühl und Selbstvertrauen >>*|. Ich kenn da jetzt den Fachausdruck nicht dafür ((lacht)).

MS: An welchen Stellen machen Sie das fest? Also was sind die auffälligen Stellen für Sie?

IP6: Ja im Prinzip, das sind ja die Stichwörter Nase, Brust und Haare, das sind ja die weiblichen, also ein grosser Teil der weiblichen, ähm, äusseren Merkmale und da machen sehr viele ihre Definition dran fest, ob sie sich als schön oder hässlich empfinden und das wird natürlich dann auch ganz stark durch die Medien geprägt, was dort als Schönheitsideal, ähm, gerade propagiert wird und das hat ja über die Jahre auch ‘n starken Wechsel erfahren. Aber ich glaube das Äussere spielt in der heutigen Gesellschaft eine sehr grosse Rolle.

MS: Mhm. (3) Wenn jetzt, ähm, diese Patientin zu Ihnen kommen würde was würden Sie sich noch wünschen für Informationen von ihr?

IP6: Ja ich denke also das eine ist sicher, ähm, ob sie das zeitlich irgendwie einordnen kann, warum das gerade mit 13 angefangen hat, das ist ja in der Regel so das Alter, wo man dann, ähm, seine Periode das erste Mal bekommt und sich stärker mit dem Thema Weiblichkeit, Fra-, Frauenbild auseinandersetzt. Sicher auch so ein bisschen die Familienanamnese zu erfragen, wie sieht denn die Familie aus, gibt’s da irgendwelche Bereiche, ähm, das ist jetzt ein bisschen plakativ, wenn man das sagt, aber ist es vielleicht so gewesen, dass dort gerade die Trennung bei den Eltern stattgefunden hat oder das Mädchen bei der Mutter oder beim Vater geblieben ist und dann irgendwelche Ersatzrollen eingenommen hat oder dort auch ein Stück weit instrumentalisiert worden ist und dort vielleicht sehr viel Schlechtes über die Mutter plötzlich gehört hat. Solche Hintergrundinformationen wären sicher wichtig, auch um bissl rauszubekommen, wo sind die Bezugspersonen, die man vielleicht dann in weitere Gespräche miteinbeziehen kann. Wo sind vielleicht auch Berater im Hintergrund tätig, die

natürlich dann auch so ‘ne Meinungsbildung stark beeinflussen und es dann häufig gut ist, wenn man die dann mal mit in die Sprechstunde rein nimmt, um nicht immer nur so Sekundärinformationen zu bekommen oder auch der Person nur Sekundärinformationen zukommen zu lassen. Weil es ist ja dann noch mal was anderes, wenn die Person in der Sprechstunde selber sitzt und vielleicht die Aussagen des Arztes hört, als wenn dann die junge Frau nach Hause kommt und ihre Person, Bezugsperson erzählt «also der Arzt hat das und das gesagt», weil das ja häufig eigentlich nur ein Spiegel ist von dem, was sie gehört hat oder wahrnehmen konnte in der Situation.

[MS legt die DSM-5-Kriterien hin.]

MS: Mhm, okay. Ähm, ich habe eine kleine Auflösung. |**<< KDS bekannt**|Ähm, das wäre ein Fallbeispiel gewesen, ähm, einer Körperdysmorphen Störung. Haben Sie das schon mal gehört?

IP6: [Ja, okay. [Ja.

|*KDS bekannt >>*|MS. Ähm, das sind ähm, die, ja, die Diagnosekriterien. Eben es ist eine übermässige Beschäftigung mit einem Makel am Körper, der aber für Andere nicht erkennbar ist oder nur geringfügig. Ähm, die Personen machen wiederholende Verhaltensweisen wie Überprüfen im Spiegel, sie vergleichen sich mit Anderen, fragen immer wieder nach und es ist mit einem sehr hohen Leidensdruck verbunden in verschiedenen Bereichen und ähm, es ist keine Essstörung, das ist, genau, eine andere Störung. Genau und einfach wichtig, eben es ist ein Makel, aber die Anderen sehen das nicht, es sieht eigentlich normal aus. Und wenn Sie sich jetzt das Fallbeispiel nochmals anschauen, ähm, haben Sie schon mal solche Patientinnen getroffen? Haben Sie Erfahrungen schon gemacht, mit Leuten, die diese Symptome zeigen?

IP6: Also ich glaube nicht in der Ausprägung, dass jetzt eben so viele Körperbereiche mit Unzufriedenheit zusammenhängen. Aber wir haben natürlich häufig die Thematik der Brust. Also, dass die Frauen kommen und sagen, ihre Brust zu klein, zu gross, zu hängend. Und das eben häufig im jungen Alter dann auch schon wo wir uns sehr, sehr schwertun, ähm. Meistens kommen sie eigentlich mit dem Anliegen von einer Operation. Und ähm, das ist für uns eigentlich der letzte Schritt, meistens muss man eigentlich, äh, ganz viele andere Bereiche erstmal abarbeiten, weil den meisten nicht klar ist, dass meistens durch ‘ne Operation das Problem gar nicht behoben wird, weil gar nicht der körperliche Mangel, der sie, den sie so definieren, ja eigentlich Ursprung ist, sondern einfach ein Selbstbild oder Selbstwahrnehmung, die vielleicht gar nicht so vorliegt. Und das natürlich nicht weg ist, wenn man operiert, also, dass ist so, man hat dann die Vorstellung, beispielsweise bei einer zu kleinen Brust, «ja, wenn ich jetzt ein Implantat bekomme, dann verändert sich mein Selbstwertgefühl», aber das verändert sich dadurch nicht, die Brust wird grösser, das ist schon so, wo man hat dann Narben, die einen dann vielleicht sekundär plötzlich auch stören oder dann stört einen vielleicht die Brust nicht mehr, aber jetzt stört einen vielleicht der kleine Bauch, den man hat. Also das ist ja meistens der Auffänger aber eigentlich nicht die Ursache. Und ähm, oder eben grosse Thematik Schamlippenkorrektur. Das ist ja was völlig Neuzeitliches, dass man irgendein Bild hat, wie der Schambereich aussehen sollte. Hängt natürlich auch damit zusammen, dass wir eine sehr viel offenere Gesellschaft sind, ich mein, früher hat man den Bereich, ausser der Arzt, nie angeschaut, heute kursieren im Netz tausend Bilder, wie man da aussehen kann und sollte. Ähm, Pornografie ist viel ähm, gesellschaftlich akzeptierter und öffentlicher als früher gewesen ist, also das heisst, da sieht man auch viel mehr Bilder als man früher auf ‘nem einfachem Weg haben konnte und in der Regel sind mehr, ist es selten so, dass Frauen mit so vielen verschiedenen Bereichen kommen, sondern es ist schon eher so, dass sie sagen, es stört schon eher primär die Brustform oder der Genitalbereich. Und ähm, oder sie kommen tatsächlich erst mal über die Sexualität oder wenn man da darüber dann mit ihnen redet, dass man dann merkt, eigentlich ist die Sexualität gestört, weil das Körperbild gestört ist und wir da dann beispielsweise viel

eher dann über die Sexualtherapie mit der Patientin arbeiten und sagen, das ist die erste Anlaufstelle und nicht die Operation oder die Veränderung vom Körperbild, weil die Sexualität wird durch die Veränderung des Körperbild sich nicht verändern. Aber das muss man dann wie mit dem Patienten so Schritt für Schritt erarbeiten und einordnen, ähm, dass eben da der Arzt nicht was macht und dann ist das Problem weg.

MS: Ja, ja. Und das kommt öfters vor, dass es jetzt für Sie aus ärztlicher Sicht es normal ist aber die Unzufriedenheit der Patienten einfach nicht im Verhältnis steht –

IP6: [Also natürlich sehen wir auch Mädchen, die zum Beispiel eine unterschiedliche Brustentwicklung haben, also eine Fehlbildung in Anführungszeichen, das ist ja keine Krankheit in dem Sinne, wenn die Brustdrüse sich nicht so stark entwickelt aber wo man dann ganz klar sagen kann das ist tatsächlich ‘ne Belastung, weil wenn man’s anschaut, wenn man ‘n BH tragen will, das ja nicht nur mit Nacktsein sondern auch einfach schon wenn man was anziehen möchte schwierig. Wo man dann tatsächlich sagen kann, das hat ‘n gewissen körperlichen Krankheitswert, den man beheben kann und der dann wahrscheinlich auch ‘n bisschen dieses Körperbild verbessert, weil tatsächlich ein Makel nach einer gewissen Definition da ist aber ähm, es ist eigentlich häufiger, dass die Frauen mit ‘ner Vorstellung kommen, die man sagt, das ist Definition, ob das schön oder hässlich ist. Das hat nichts mit ‘nem tatsächlich vorliegenden Krankheitswert zu tun.

MS: Ja. Und jetzt bei solchen Patientinnen mit solchen Symptomen, was sind die Herausforderungen für Sie?

IP6: Sie zum Psychologen zu kriegen ((lacht)).

MS: Okay ((lacht)).

IP6: Ja, weil, das hat natürlich ganz schnell, also man muss sehr, sehr vorsichtig sein, wenn man dann diese Thematik anspricht, weil viele dann mit ‘ner Abwehrreaktion reagieren und dann sind, sind sie nicht mehr im Gespräch zugänglich, sondern dann haben sie so das Gefühl, «derjenige, mein Gegenüber nimmt mich nicht ernst und der will mich zum Psychiater und Psychologen- «, und da fängt ja schon die Definition an, viele kennen den Unterschied zwischen Psychologen und Psychiatern gar nicht, das wird alles in einen Topf geschmissen und dann haben sie sofort das Gefühl «das sind Menschen, die mir irgendwelche Medikamente verschreiben wollen, die sagen, ich hab ‘ne Störung aber ich nehm’ mich ja als normal wahr, ich hab ja tatsächlich ‘n Grund dafür, dass ich so empfinde und ich will nicht psychisch krank gemacht werden», was ich als normal empfinde und meistens muss man da erstmal wie ‘ne Beziehung zu dem Patienten aufbauen, indem man eben über sehr viel zusätzliches noch spricht, bevor man dann sagt «Ach, per Definition», also ich sag dann manchmal auch nicht, dass das jetzt ein Psychiater ist, zudem ich die Patienten schicke, sondern sozusagen, dass es wichtig ist, in solchen Entscheidungsfindungen, dass man da eine neutrale Person hat, die einen unterstützen kann, weil die Familie eben, ähm, oft einem zu nahe steht, um einen neutral zu beraten und ich als Arzt das nur in einem bestimmten Bereich kann aber eben, ähm, sicher gut ist, weil es auch mehr Folgen hat, solche Eingriffe, wenn man es psychologisch begleiten kann. Und da drüber kann man häufig die Patienten dann eher dazu bewegen, wenn man das eben nicht als Psychotherapie bezeichnet oder als psychiatrische Konsultation sondern als ‘ne Begleitung und Evaluationsgespräch, dass es wichtig ist, dass man von verschiedenen Seiten betrachtet und eben ja, je nach Patient und Vokabular da eben auch sehr sorgfältig aussucht, welche Begrifflichkeiten man wählt und das ist häufig mit so, nicht mit einer Konsultation getan.

MS: Ja, mhm okay. Ja genau, die nächste Frage wäre: Wie ist Ihr professionelles Vorgehen, um diese Herausforderungen ähm, ähm zu, also mit diesen Herausforderungen umzugehen? Da haben Sie schon gesagt, Sie nehmen sich Zeit, schauen das genau an, Sie sagen nicht

gleich, es ist ein Psychiater, Beziehungsaufbau haben Sie noch gesagt, ähm, kommt Ihnen noch etwas in den Sinn?

IP6: Ja auch teilweise Beratung mit den Fachkollegen, also jetzt gar nicht mal dass man unbedingt primär die Patientin dort hinschickt, sondern dass man sich auch ein Stück weit selber Rat holt, also wir haben, zum Beispiel jetzt bei ihr passt es jetzt nicht, aber wir haben einen sehr grossen Bereich der Psychoonkologie, wo's manchmal hilfreich ist, sich dort ein Feedback zu holen, «Du hör mal zu, ich hab die und die Patientin und im Gespräch hat sich der und der Problemkreis ein bisschen herauskristallisiert, siehst du da jetzt noch 'n Ansatzpunkt wenn ich sie das nächste Mal sehe, ähm, in welche Richtung das Gespräch gehen sollte, hast du vielleicht neutralere Ansprechpersonen, ähm, Beratungsstellen in dem Bereich, wo ich die Patientin mal hinschicken kann», ähm, also es ist wie so beides, das eine ist das Gespräch mit dem Patienten aber das andere ist natürlich auch das sich selber Rat holen bei den Kollegen, die dort mehr involviert sind und eben vielleicht manchmal auch 'n guten Tipp geben können, ähm, «Sprech doch das und das mal bei der nächsten Konsultation an, so ein bisschen als Vorbereitung für den nächsten Schritt».

MS: Mhm, okay. Ähm, dann noch meine letzte Frage, ähm ist, wie wäre denn Ihr professionelles Vorgehen, wenn Sie jetzt so einen Fall wie diese Lea bei sich hätten? Wie würden Sie da vorgehen?

IP6: Also ich glaube ich würde sie sicher noch genauer befragen was für 'ne Vorstellung sie denn von sich hat idealer weise. Das heisst, um das auch 'n bissl zu konkretisieren, ich mein, kleine hässliche, hängende Brüste, was bedeutet das? Findet sie das Volumen zu klein, ist es tatsächlich so, dass die Brust hängt oder hat sie 'ne Vorstellung-, manchmal frage ich die Patienten auch, ob sie mir Bilder mitbringen können, wie denn ihre ideale Brust aussieht. Ähm, weil man dadurch auch ein bisschen mehr ins Gespräch über diese Thematik kommt, was ist denn die ideale Brust? Gibt es die überhaupt? Ähm, wie sieht 'ne Brust im Vergleich zur Statur aus? Ich mein', wenn jemand 'n bisschen dicker ist, dann sieht 'ne sehr grosse Brust anders aus als wenn jemand sehr, sehr dünn ist und vielleicht stört jemand dünnes 'ne grosse Brust, während jemand, der ein bisschen dicker ist, findet das genau richtig. Und ähm, das hilft manchmal dort so ein bisschen besser ins Gespräch zu kommen und natürlich auch zu erfragen, was für 'ne Erwartung hat die Patientin denn jetzt an diese Konsultation, an das was ich machen soll oder wie ich das Problem lösen soll, um auch da ein bisschen nachhaken zu können, ist es ein Hilfesuchen, was zwar vordergründig mit 'ner Operation begründet ist, weil das am einfachsten ist oder ist es so, dass sie einfach gar keinen Ansprechpartner hat, eigentlich weiss, dass es was ist, was psychologisch eigentlich besser behandelbar wäre aber sie wie nicht weiss, wo sie da jemanden findet, der sie begleiten kann. Dass das dann erst über eine ärztliche Konsultation läuft aber eigentlich gar nicht das primäre Ziel hat, das sie sich operativ verändern lassen möchte. Ich glaub das wäre wahrscheinlich das, wie man da so 'n bisschen dran gehen würde. Meistens sage ich dann nach der Erstkonsultation «Lassen Sie sich das erst mal nochmals durch den Kopf gehen oder wir treffen uns in einem Monat noch mal, da können Sie vielleicht noch die Sachen sich ein bisschen aufschreiben, die Sie noch mitbeschäftigt oder eben Bilder mitbringen von dem, was Sie gerne möchten», um eben auch ein Stück weit diese Beziehung aufzubauen, weil ähm, von einmal sehen und sprechen, ähm, ist es nicht möglich all die Informationen zu bekommen, um weitere Schritte planen zu können und eben, sie dann einfach nur in Anführungszeichen weiterzuturfen ist meistens nicht die Lösung, weil sie brauchen meistens jemand, der sie ein Stück weit auch parallel begleitet und ähm, sie eben das Gefühl haben, dass sie ernst genommen werden, das ist glaube ich das A und O in so 'nem Ersttreffen, dass die Patienten sagen «derjenige, der mit gegenübersitzt, der hört mir zu und der nimmt mich ernst, also kann ich auch vielleicht das nächste Mal noch was erzählen, was ich dieses Mal noch gar nicht erzählen wollte».

MS: Mhm mhm, okay. Und dann schlussendlich würden Sie die Person weiterverweisen oder bei sich behalten?

IP6: Weiterverweisen.

MS: Okay. Gut, vielen Dank.

OD: Gut, dann mach ich mal weiter. Ähm, das haben Sie schon ein bisschen angesprochen, was sind Ihre Erfahrungen mit Patientinnen, die Fragen zur Ästhetik oder Normalität ihres eigenen Genitalbereichs haben?

IP6:|**<< Zunehmend**| Zum einen, dass es zunimmt, ähm, das ist früher viel, viel seltener gewesen,|*Zunehmend >>*| |**<< Jüngere**|dass das Alter immer jünger wird, dass die Mädchen sich immer früher damit beschäftigen und da auch Vorstellungen wagen zu artikulieren, wo ich dann, ähm, manchmal ihnen auch ganz klar sagen muss, sie haben noch gar nicht das Alter erreicht, wo so was überhaupt diskutiert werden kann, |**<< Erwähnen OP**|also zum Beispiel ästhetische Operationen vor dem 18. Lebensjahr sind in der Schweiz nicht zulässig, muss man ganz klar sagen.|*Jüngere >>*|*Erwähnen OP >>*| Sind-, ist vielen aber nicht klar, |**<< Gelder**|weil es gibt natürlich andere Länder, wo das ganz anders ist, wo man sehr früh in solche ästhetischen Operationszyklen hineintreten kann, wenn man genügend Geld hat. |*Gelder >>*|Ähm, ist zum Glück, ist ja auch rechtlich in der Schweiz sind dem gewisse Grenzen auferlegt, ähm, wenn man sich damit auseinander setzt, |**<< Abneigung Chirurgie**|ich weiss es ist trotzdem Ärzte gibt, die das zu jüngerem Alter machen.|*Abneigung Chirurgie >>*| |**<< Zunehmend**|**<< Jüngere**|Aber ich glaub die Tendenz ist, dass es mehr wird und dass die Frauen jünger werden, die mit solchen Fragestellungen kommen.

|*Zunehmend >>*|*Jüngere >>*|OD: Was ist denn Ihr professionelles Vorgehen zu den Fragen?

IP6: |**<< Untersuchung**|Also zum einen muss man sicher herausbekommen, ob tatsächlich irgendein Krankheitswert vorliegt.|**<< Medizinische Beschwerden**| Es gibt ganz, ganz wenige Frauen, da ist es tatsächlich so, die haben |**<< Labien**|sehr, sehr grosse Schamlippen beispielsweise|*Labien >>*| und da ist es tatsächlich ein Problem, dass sie sagen «Wenn ich Sport mache, wenn ich Fahrrad fahr, dann hab' ich immer ein Wundseingefühl» und das gibt's tatsächlich.|**<< Brüste**| |*Medizinische Beschwerden >>*|Oder eben, dass es Fehlbildungen von der Brust gibt, wo man sagen muss, da liegt tatsächlich 'ne Fehlbildung vor |*Brüste >>*|und|**<< Sinnvoll**| man kann nachvollziehen, dass da der Wunsch nach einer Korrektur vorliegt.|*Untersuchung >>*| Ähm, das ist sicher der eine Punkt, sich da ein Bild draus zu machen und wenn das eben nicht vorliegt, dann wird das n' viel schwierigerer Bereich, weil man dann natürlich ganz klar sagen muss, es liegt zum einen kein Krankheitswert vor, der definiert ist. |*Sinnvoll >>*|**<< Gelder**|Das zweite ist, dass es dann, wenn man tatsächlich irgendwann an den Punkt kommt, dass man das kosmetisch operieren lassen möchte, ganz klar wissen muss, dass das eine Kostenfrage ist weil es von der Krankenkasse nicht übernommen wird und wir dann auch nicht versuchen, da irgendwelche Gutachten so hinzudrehen damit die Krankenkasse das in irgendeiner Form übernimmt. |*Gelder >>*|Ähm, (2) und |**<< Aufklärung OP**|dann natürlich auch ganz klar gewisse Risiken zu artikulieren, also viele haben ja das Gefühl, das sind kleine Operationen, wenn's dann mal gemacht ist, dann ist gut, aber dass jede Operation mit 'nem Risiko verbunden ist, also man sich bewusst sein muss, ähm, sei es allein ein gewisses Narkoserisiko, dass man eigentlich aus einer gesunden Situation heraus ein Risiko eingeht, was nicht unbedingt sein muss. Das andere ist natürlich, dass es Folgen haben kann, so 'ne Operation, die einen krank machen aus 'ner gesunden Situation heraus, sei es Infektionen, die man nicht erwartet, die zwar nicht häufig sind, aber wenn sie auftreten, schwerwiegende Folgen haben kann.|*Aufklärung OP >>*| |**<< Brüste**|Zum Beispiel bei Brustoperationen |**<< Komplikationen** |kann es tatsächlich sein, dass man 'ne normale, gesunde Brust hat und nachher 'ne hässliche Brust hat, weil die Wundheilungsstörungen die Brustform verändern, chronische Schmerzen erzeugen können. Genau das gleiche ist im Genitalbereich, da entstehen, können Narben entstehen, die für immer bleiben, die für immer ähm, Sensibilitätsstörungen machen können,|**<< Sexualität**| die für immer die Sexualität

verändern können |*Sexualität >>*|und dann hat man eigentlich die gesunde Frau krank operiert.|*Brüste >>*|*Komplikationen >>*| |**<< Medien**|**<< Uninformiert**|Und auch das ist vielen nicht so klar, wenn sie primär mit dem Wunsch kommen, sondern sie sehen halt irgendwelche Bilder und sagen «ist 'ne kleine Sache und das macht man jetzt und danach geht's es mir psychisch besser».|*Medien >>*|*Uninformiert >>*| |**<< Aufklärung OP**|**<< Miteinbeziehen**|Und diesen ganzen Bereich muss man halt wirklich Schritt für Schritt mit dem Patienten besprechen und sagen «Haben Sie sich das mal überlegt?», auch das zu besprechen bevor man 'ne Entscheidung trifft. |*Aufklärung OP >>*|*Miteinbeziehen >>*|**<< Uninformiert**|Man merkt oft, dass die Informa-, also die Information, die die Patienten vorher haben, lang nicht so fundiert ist, wie man das eigentlich erwarten würde, dass jemand zum Arzt kommt, mit einem ausgeprägten Wunsch und er dann sagt «Ich hab' schon seit fünf Jahren den Wunsch, das operieren zu lassen». Da würde man ja annehmen, dass man sich seit fünf Jahren mit all diesen Bereichen beschäftigt hat, häufig haben sie das aber nicht.|*Uninformiert >>*| |**<< Unrealistisches Ideal**|Sondern es ist mehr so, sie haben sich mit einem Ideal beschäftigt und überlegt, wie sie zu diesem Ideal kommen, dass dieses Ideal erstens nicht realistisch ist, zweitens, vielleicht auch Probleme verursachen kann, denen sie nicht bewusst sind, das wird gerne ausgeblendet.

|*Unrealistisches Ideal >>*|OD: Fragen Sie manchmal auch bei Ihren Patientinnen nach, wie sie zur Ästhetik ihres eigenen Genitalbereichs stehen?

IP6: Nein. |**<< Psychische Probleme im Hintergrund**|Also, wenn sie das nicht äussern oder indirekt Zeichen aussenden, dass sie damit ein Problem haben, indem sie zum Beispiel immer wieder kommen und sagen, sie haben Jucken und Brennen und man kann da überhaupt nichts finden oder ähm, sie haben irgendwelche Schmerzen, die man aber nicht so richtig zuordnen kann, dann frage ich nicht konkret nach. |*Psychische Probleme im Hintergrund >>*|**<< Fragen medizinisch**|Also eben, es müssen gewisse Anzeichen vorliegen, dass ich sag', das könnte als Problemkreis dahinterstecken, wenn's von 'ner Patientin nicht selber kommt und dann sprech' ich's auch aktiv an. Aber wenn sie jetzt dazu gar nichts sagen, äussere ich's auch nicht. |**<< Medizinische Beschwerden**|Ausser ich sehe natürlich Veränderungen im Genitalbereich, die tatsächlich krankheitsbedingt sind, ähm, so Autoimmunerkrankungen, die zum Beispiel zu 'ner Verhärtung der Haut führen und ähm, da sprech' ich zum Beispiel aktiv die Sexualität an.|*Fragen medizinisch >>*|*Medizinische Beschwerden >>*|**<< Sexualität**| Ich frag' sie «Haben Sie Probleme bei der Sexualität?», |**<< Alter**|weil das dann manchmal ältere Frauen sind, die das gar nicht so ansprechen wollen und dann auch plötzlich froh sind, wenn man's anspricht.|*Alter >>*| Sie sagen «Ja, ähm, ich habe schon seit fünf Jahren keinen Geschlechtsverkehr mehr gehabt mit meinem Mann, weil es immer weh tut, aber ich hab' gedacht, da kann man nix ändern und da muss ich mit leben», dann ja, aber wenn sie nur zur Kontrolle kommen und da keinerlei Zeichen aussenden, dass sie 'n Problem haben könnten, dann nicht.

|*Sexualität >>*|OD: Gut, dann habe ich jetzt auch noch ein Fallbeispiel dabei, wenn Sie das mal durchlesen könnten.

[OD legt Fallvignette 2 hin. IP6 liest Fallvignette durch.]

OD: Ähm, das wär' jetzt ein Beispiel einer Körperdysmorphen Störung des Genitalbereichs. Also von der Körperdysmorphen Störung könnte jedes Körperteil betroffen sein und hier würde es um den Genitalbereich gehen. Ähm, was sind Ihre Erfahrungen mit Patientinnen, die jetzt solche Symptome zeigen?

IP6: |**<< Psychische Probleme im Hintergrund**|Also sehr häufig artikulieren sie nicht so sehr die Sorge um ihr Aussehen, sondern sie berichten primär Beschwerden,|**<< Gespräch**| weil das sehr viel einfacher ist ins Gespräch da drüber zu kommen, |*Gespräch >>*|als zu sagen «Ich hab' das Gefühl, das sieht nicht normal aus» oder es kommt dann eben ganz häufig «Also ich hab' ganz häufig da so ein Wundseingefühl» und da drüber versuchen sie

dann über, in den ästhetischen Bereich zu kommen.|**<< Seltene KDS**| |*Psychische Probleme im Hintergrund >>*|Es kommt seltener vor, dass sie direkt das ansprechen und sagen «Ich hab' das Gefühl, das sie, sie ist nicht normal und ähm, wie stehen Sie dazu?|*Seltene KDS >>*|» Ähm, manchmal ist es tatsächlich so, dass die dann sogar mit ihrem Partner kommen, was dann ein bisschen schwierig ist, weil die Kommunikation mit dem Partner dann häufig ein bisschen anders läuft als wenn sie alleine sind. |**<< Beziehung**|**<< Gespräch**|Ich denk, dann ist es, dann ist es manchmal gut, wenn man sowohl 'n Paargespräch|*Gespräch >>*| als aber auch die Situation sucht, wo man die Patientin alleine sprechen kann. Dass man zum Beispiel sagt «Ich würd jetzt gern noch mal untersuchen, wollen Sie solange kurz mal rausgehen, wir holen Sie dann nachher wieder rein», um natürlich gewisse Hemmschwellen in der Kommunikation zu vermeiden, wo man vielleicht Dinge nicht anspricht, weil's den Partner belasten könnte oder eben schon nervt, dass man da schon tausend Mal darüber geredet hat und das alles normal ist, ähm, ja. Also meistens läu-, kommt es erstmal über die Problemebene. |*Beziehung >>*|**<< Direkt OP**|Aber das hängt natürlich auch mit dem gynäkologisch sein, ich glaub wenn Frauen sich sehr damit beschäftigen und rein ästhetisch das Problem angehen wollen und da keine Hemmungen haben, die gehen dann zum ästhetischen Chirurg, der das in seinem Portefeuille anbietet, Genitalverschönerung und ähm, weil sie natürlich dann auch weniger ähm, Hürden zu durchlaufen haben. Weil ich mein, wenn die das anbieten, dann ist es auch primär der Fokus drauf was möchte die Patientin und wie können wir dahin kommen.|*Direkt OP >>*| |**<< Aufklärung OP**|Wenn sie über das Gynäko-, über den Gynäkologen kommen, dann wird viel mehr das im Fokus stehen, dass man sagt, ist es überhaupt notwendig und wenn ja, welche Folgen kann das haben. |*Aufklärung OP >>*|Das ist dann immer eine Frage des Zugangsweges. |**<< Gelder**|Mache erhoffen sich natürlich den Weg vom Gynäkologen, dass das dann von der Krankenkasse bezahlt wird, weil sie finanziell nicht die Möglichkeiten haben das primär über den ästhetischen Chirurgen laufen zu lassen, aber da hat man sicher viele Varianten.|*Gelder >>*|

OD: Und ähm, wie wäre jetzt mit dieser Patientin Ihr professionelles Vorgehen?

IP6: |**<< Vielfalt**|Also ich würde ihr sicher einmal erklären, wie gross die Varianz ist von dem Ge-, äusseren Genital |*Vielfalt >>*|und |**<< Aufklärung**|dass es gewisse Definitionen gibt, die natürlich auch ärztlich gemacht sind, wann man sagt, dass es überhaupt zu Problemen rein physikalisch führen kann und wann das eigentlich kein Problem ist. |*Aufklärung >>*|**<< Veränderungen**|Dann würde ich ihr sicher auch den Bereich berichten, dass ihr bewusst sein muss, dass das Genitale sich im Lauf des Lebens ständig verändert, das heisst natürlich, das sieht bei einem zwölf-, dreizehnjährigen Mädchen anders aus als bei jemand, der über zwanzig ist, dann verändert sich das ganz stark, |**<< Schwangerschaft/Geburt**|wenn man Kinder gebärt, da verändert sich das Genitale komplett,|*Schwangerschaft/Geburt >>*| das sieht nachher ganz anders aus und dann natürlich noch mal 'ne Phase der Veränderung, wenn man dann in die Wechseljahre kommt und die Hormone dann weniger werden, auch da gibt es noch mal eine Veränderung, das heisst, es ist kein Bereich, der statisch immer gleich ist, selbst wenn man den operiert, selbst dann verändert er sich nachher noch. Er verändert sich auch durch Gewichtszu- und -abnahme, das ist sicher ein Bereich, den man den Patienten auch bewusst sagen muss, vielleicht stört sie es jetzt aber wenn gewisse Ereignisse in ihrem Leben kommen, kann das sein, dass sie sich dann wiederum verändert|*Veränderungen >>*| und sie's dann nicht mehr so als störend empfinden und |**<< Miteinbeziehen**|dann ganz sicher mal die Frage, ob sie das Gefühl hat, dass 'ne |**<< Aufklärung OP**|Operation das Problem verändern würd' und ähm, dann kommt bei mir meistens relativ schnell, ob sie sich bewusst sind, dass ein operativer Eingriff Narben hinterlässt, dass die sichtbar sind, |**<< Medien**|dass das vielleicht nicht so aussieht, wie man das in den retuschierten Bildern im Internet gesehen hat ((lacht)).|*Medien >>*| |*Miteinbeziehen >>*|Ähm, |**<< Gespräch**|ja, ich glaub, das wären so ein bisschen die ersten Punkte, die ich ansprechen würde.

|*Aufklärung OP >>*|*Gespräch >>*|OD: Mhm, ähm, vorhin haben Sie gesagt, dass Sie immer

mehr solche Frauen antreffen.

IP6: Ja.

OD: Was heisst das, können Sie da irgendeine Zahl nennen, also wie oft kommen Frauen mit so Fragen?

IP6: ((überlegt)) (3). |**<< Brüste**|Also, wenn man's jetzt auf so spezifische Fälle runterbricht, dass das jetzt zum Beispiel nur die Schamlippen sind, die stören oder nur die Brust, die stört, dadurch, dass ich natürlich Brustschwerpunkt habe, seh' ich wesentlich mehr Frauen mit Brustproblemen als im Genitalbereich, das heisst, ich sehe wahrscheinlich mindestens (1) alle zwei Wochen eine, wenn nicht sogar jede Woche eine Patientin, die nicht zufrieden ist. Ist dann häufiger der Bereich, dass sie sagen ihre Brust ist zu gross, weil das häufig auch Zuweisungen dann von den Hausärzten sind, ähm, weil die Brust vergrössern ist natürlich mehr Schwerpunkt plastische Chirurgie, die sehen dort natürlich wesentlich mehr Frauen als wir Gynäkologen direkt,|*Brüste >>*| ähm, |**<< Jährlich**|**<< Einige KDS**|was den Genitalbereich angeht, denk ich, ich seh' alle drei Monate 'ne Patientin mit der Fragestellung und (1) also gerade Genitalbereich hab|**<< Zunehmend**| ich glaube ich erst die letzten fünf Jahre, ist's überhaupt Thema gewesen, davor hab ich glaub ich nie 'ne Patientin gesehen, die mit so 'ner Fragestellung gekommen ist. |*Zunehmend >>*|**<< Medien**|Also ich glaube, dass, da hat die mediale Welt schon 'n grossen Anteil dran.

|*Jährlich >>*|*Einige KDS >>*|*Medien >>*|OD: Ja, gut, ähm, wie gross schätzen Sie das Interesse an einer Fortbildung zur Körperdysmorphen Störung im gynäkologischen Fachbereich ein?

IP6: |**<< Niedergelassene**|Also ich glaub, dass das bei den Niedergelassenen wahrscheinlich schwerpunktmässig grösser ist das Interesse, als bei den Kollegen, die in der Klinik arbeiten, weil ähm, die natürlich häufig ein gefiltertes Patientengut haben. |*Niedergelassene >>*|Das haben wir an der Polyklinik, wo wir die Gynäkologen sind aber häufig gehen die erst mal zu einer niedergelassenen Gynäkologin oder zur Gynäkologin der Mutter oder - auch immer bevor die dann zu uns kommen. Und meistens sind ja auch schon gewisse Vorgespräche gelaufen, grad wenn es über eine Zuweisung von den niedergelassenen Gynäkologen läuft. |**<< Zusammenarbeit**|Und ich glaube, dass in dem Bereich dadurch, dass es schwieriger ist, das interdisziplinäre Zusammenarbeiten wahrscheinlich mehr Informationsbedarf ist, einfach was kann ich vielleicht schon als Gynäkologe für Schritte einleiten oder ähm, dem Patienten anbieten, während wir natürlich relativ schnell Zugang haben zu unseren psychologischen Kollegen, wo's sehr viel einfacher ist auch eben mal auf kurzem Weg 'nen Rat zu bekommen als jetzt für die Niedergelassenen.|*Zusammenarbeit >>*| |**<< Mittel Interesse an Fortbildung**|Also ich glaub das wär wahrscheinlich, ist mehr in dem Bereich von den Niedergelassenen, wobei auch wir machen Fortbildungen, |**<< Sexualität**|also gerade was Sexualität angeht, haben wir doch mindestens einmal alle sechs Monate irgend einen Teilbereich in unseren Fortbildungen und dann meistens eben als Teil von einem Gesamt|*Sexualität >>*|- ähm, also nicht als Einzelfortbildung aber zum Beispiel Probleme im Genitalbereich, wo dann eben die rein klinischen, pathologischen Dinge und dann eben die Sexualität, Körperbildstörung mit abgearbeitet werden, dass man wie so einen Gesamtüberblick bekommt.

|*Mittel Interesse an Fortbildung >>*|OD: Mhm. Und wie gross schätzen Sie das Interesse oder der Nutzen an Hilfsmitteln zur Diagnostik von Körperdysmorpher Störung ein? Also so in Form von Screeninginstrumenten zum Beispiel.

IP6: |**<< Grosses Interesse an Diagnostik**|Ich glaube, dass es sehr hilfreich ist|*Grosses Interesse an Diagnostik >>*|, |**<< Unpraktisch**|das Problem ist meistens, dass man 'ne gewisse Zeit braucht, damit man die benutzen kann, also man muss sich damit beschäftigen und das ist immer 'ne Frage des Zeitmangels. Ich kenn das in vielen anderen Bereichen,

inzwischen gibt's ja ganz, ganz gute Evaluationstools, sei es das Geriatric Assessment Tool, sei es ähm, Chemotherapieevaluationstool, sei es Cardiac, also die Herzbelastbarkeit von 'nem Patienten, ähm. Und ich weiss, dass das alles gibt und ich weiss auch wo ich das finden kann, anwenden tu ich das ganz, ganz selten. Und ich glaub, das ist so ein bisschen das Problem, dass das eigentlich gute Tools gibt, häufig dass man teilweise auch weiss, dass es die gibt, aber dass die Anwendung dann 'ne gewisse Zeitintensität benötigt, die man häufig in 'nem normalen Sprechstundenalltag nicht aufbringen kann oder will, also es ist ja immer im Rahmen des Wollens und nicht nur so, dass man ((lacht)) dass man es nicht kann, sondern man muss es einfach definieren, ähm, man sich dann fast die Zeit nehmen muss |*Unpraktisch >>*|und sagt, |**<< Objektivieren**|man macht 'n Termin zur Evaluation und dann wendet man das Tool an und dann hat man sicher ein objektiveren Eindruck als subjektiv, «ich hab mal gehört, dass und das passt da ganz gut rein und deswegen könnte es sein», ähm (2) aber ich glaube, die Zukunft liegt da drin, weil schon allein von den abrechenbaren Dingen im Praxisalltag das immer mehr so sein wird, dass man objektivierbare Tools benötigt, damit's eben nicht der allwissende Arzt ist, der da irgend 'ne Diagnose stellt, sondern man das auch objektivieren kann.

|*Objektivieren >>*|OD: Mhm. Und ähm, wie gross schätzen Sie das Interesse für Hilfsmittel zur Beratung jetzt von Patientinnen spezifisch mit diesem Problem, also mit Körperdysmorpher Störung des Genitalbereichs, ein?

IP6: |**<< Grosses Interesse an Hilfsmittel**|Also ich glaub, dass das sicher sinnvoll ist, wenn man ein gewisses Informationsmaterial aushändigen kann. |*Grosses Interesse an Hilfsmittel >>*|**<< Info zum mitgeben**|Also ich glaube, das hilft häufig im Gespräch, wenn man eben auch den Patienten irgendwelche Leitfäden auch aushändigen kann, dass sie wie Material nach Hause nehmen auch zur Reflektion von dem Gespräch, was man geführt hat und ich glaube, |**<< Medien**|es ist immer gut, weil sie ja dann doch dazu neigen, im Internet zu recherchieren und ähm, da findet man dann viel Gutes und viel Schlechtes und kann's nicht filtern und wenn man dann wie eine Informationsbroschüre hat, die evaluiert ist oder eben auch von Psychologen, Psychiatern mitgestaltet worden ist, ist es sicher sinnvoller als Begleitung für die Patienten und es ist auch sinnvoll, und es im Gespräch wiederum hilft.|*Info zum mitgeben >>*|*Medien >>*|

OD: Gut, dann wären wir am Ende ausser Sie möchten noch etwas hinzufügen?

IP6: Nein ((lacht)), ich glaub mir fällt jetzt aktuell dazu nicht mehr noch mehr ein.

MS: Danke vielmals.

Interview mit: IP7
Datum des Interviews: 24.05.2019
Dauer: 41 min
Anonymisierte Begriffe: Personennamen
IP-Nummer: 7

MS: Mit welchen psychischen Problemen kommen denn Patientinnen zu Ihnen in die Praxis? Gibt es das überhaupt?

IP7: Was, Probleme? Sagen Sie mir ein paar, die keine Probleme haben.

MS: Ja, was wären denn so die häufigsten oder –

IP7: Die häufigsten Probleme?

OD: Also psychische.

IP7: Psychische Probleme? Achso. Ja gut, |**<< Restkategorie Störungen**|Wechseljahrprobleme haben im Grunde genommen auch einen psychischen Anteil|*Restkategorie Störungen >>*|, oder? Einen grossen psychischen Anteil. |**<< Sexuelle Funktionsstörungen, nicht verursacht durch eine organische Störung oder Krankheit (F52)**|Libidostörungen|*Sexuelle Funktionsstörungen, nicht verursacht durch eine organische Störung oder Krankheit (F52) >>*|, |**<< Essstörungen (F50)**|Essstörungen|*Essstörungen (F50) >>*|, |**<< Restkategorie Störungen**|unerwünschte Schwangerschaftsstörungen, mit grossen psychischen Auswirkungen|*Restkategorie Störungen >>*|, |**<< Depressive Episode (F32) und rezidivierende depressive Störung (F33)**|Depressionen|*Depressive Episode (F32) und rezidivierende depressive Störung (F33) >>*|, zuhauf, (2), ja, das ist so ein Abriss.

MS: Mhm, ähm, welche Rolle spielt die Zufriedenheit mit dem äusseren Erscheinungsbild bei Ihren Patientinnen?

IP7: Schwierige Frage, ich habe sie erwartet. (3) Es ist unglaublich schwierig, den heutigen Normen zu entsprechen, (2) leider. Und ich staune, wie wenig gerade junge Leute akzeptieren können, wie sie sind. Ich erinnere mich natürlich auch an meine Jugend, auch ich war mal jung, habe aber nicht gelitten, oder. Und das Leiden, finde ich, hat zugenommen. Das Leiden an Unzulänglichkeiten, Akzeptieren der Inperfektion des menschlichen Körpers und des menschlichen Geistes natürlich. Das macht mich manchmal ein wenig traurig, weil jeder Mensch mit zwanzig schön ist, basta. Mit siebzehn noch schöner und mit vierzig noch schöner, oder? ( ) da geht's bergab.

MS: ((lacht)) Also würden Sie sagen, dass es zugenommen hat, dass man darüber spricht?

IP7: Ich würde eher so, nein, dass man unter diesem Druck steht. Darüber sprechen, das macht man eben nicht so. Aber unter diesem Druck steht man. Es kommen ja dann sicher noch detailliertere Fragen dazu, oder?

MS: Ja, vielleicht ((lacht))

IP7: Ich bin natürlich, ja, weil in meinem Alter ist man natürlich durch gewisse Normen durch. Also ich habe Normen jeder Form erlebt. Also die PERSONENNAME1 war ein kleiner Finger, flach wie ein Brett, kein Po, kein Busen, nix, nur dürr. Das war das Schönheitsideal. Und heute ist das pure Gegenteil das Schönheitsideal. Ein riesiger Busen und ein aufgepumptes Hinterteil, völlig grotesk, oder, dieser Wechsel? Und wenn man dann

hinschaut, sieht man, wie diese Normen wechseln und wie abstrus dies ist, was da den jungen Menschen für Normen aufgezwungen werden. Das ist im Verlauf der Geschichte ja nicht anders aber das ist in einem Leben passiert, oder. In einem bewussten Leben, zwischen den 60ern und den 2010er Jahren. Aber das ist ja eine halbe Predigt.

MS: Ich habe jetzt eine Fallvignette für Sie, die Sie gerne durchlesen dürfen.

[MS legt Fallvignette 1 hin. IP7 liest Fallvignette 1 durch.]

IP7: Ja, das ist genau das.

MS: Was für eine psychische Störung kommt Ihnen bei diesem Fallbeispiel in den Sinn? Oder Störungen?

IP7: Eine psychische Störung?

MS: Mhm.

IP7: |**<< Begründung**|Ja, also ein psychisches Problem, eine Störung ist das in meinen Augen nicht aber|**<< vermindertes Selbstwertgefühl und Selbstvertrauen**| mangelndes Selbstvertrauen|*vermindertes Selbstwertgefühl und Selbstvertrauen >>*| und (3) mangelnde (2) oder eben nicht akzeptieren zu können, wie man ist. Das ist ja nicht unbedingt eine Krankheit aber (3) ein bisschen unverständlich natürlich.|*Begründung >>*| Aber das darf man dieser jungen Frau, ja fünfundzwanzig jährige Frau, natürlich nicht sagen. Aber vielleicht am Anfang war das schwieriger, als ich selbst noch jünger war, jetzt da ich älter bin muss ich sagen, kann ich es vielleicht etwas besser machen, weil man es mir eher glaubt. Eine Frau, die nicht zufrieden ist, wie sie aussieht, zu fragen, warum sie nicht zufrieden ist mit ihrem Körper. Wo sie beispielsweise das Problem sieht, warum sie nicht zufrieden ist. Manchmal kommen dann interessante Antworten und sie sagt, der Vater habe schon immer gesagt, sie sei zu wenig schön, oder was auch immer. Oder von mir aus hat sie eine langjährige Beziehung, in welcher sie immer das Gefühl hatte, sie sei nicht attraktiv genug, weil sie in ihren Augen einen sehr attraktiven Freund gehabt hat. Oder, vielleicht kommt die Antwort von selbst und sonst muss man halt einfach ein bisschen darüber reden und probieren, sie auf die Antwort zu bringen.

MS: Welche Stellen im Text machen Sie stutzig? Sie haben vorher fehlendes Selbstvertrauen erwähnt, an welchen Stellen machen Sie das fest?

IP7: Ja, sie vermeidet soziale Aktivitäten, geht nicht raus. Sie leidet unter ihrem Makel (2), viel zu stark. Sie zieht sich zurück, das ist der Anfang vom Ende, bevor die Zerstörung kommt. Wenn sie das nicht ändern kann, wird sie krank. Dann hat sie dann eine Diagnose. Jetzt hat sie ja noch keine, aber sie ist auf bestem Weg dazu.

MS: Was würden Sie denn für eine Diagnose geben?

IP7: Nachher?

MS: Ja.

IP7: Ja das ist noch schwierig, ich bin kein Psychologe, (3) ich weiss nicht, wie man dem psychologisch sagt, eine Autoimmunitätsinkompetenz.

MS: Okay.

IP7: Ja ich meine, sie zieht sich zurück, |**<< Phobische Störung (F40) und andere Angststörungen (F41)**|sie wird phobisch, oder? Phobien, vor dem Leben.

|*Phobische Störung (F40) und andere Angststörungen (F41) >>*|MS: Mhm, okay. Ähm, Sie haben es vorhin schon ein bisschen angesprochen. Also wenn jetzt so eine Patientin bei Ihnen in der Praxis wäre, was würden Sie sie noch fragen? Welche Informationen würden Sie sich noch einholen, wenn sie sich jetzt so präsentiert?

IP7: Ich würde sie fragen, das Problem ist, wie kann ich unterscheiden, gut, ich habe ja sowieso nicht viel Zeit und muss diese herauspicken, bei denen ich den Eindruck habe, dass ich etwas bewirken kann. Sonst hat es ja keinen Sinn. Und das ist vielleicht ein bisschen unsere Kunst, aus diesen vielen menschlichen Facetten, die herauszupicken, die vielleicht etwas verbessern oder verändern kann. Und man kann sie ja mal fragen, man kann sie reden lassen, ich muss nicht reden, sie soll von sich erzählen, wie es ihr geht. Ich habe den Eindruck, sie sei nicht selbstsicher. Sie schaut mich nicht an, sie ist verklemmt, verscheucht, unsicher. Dann kann man sie ja einfach ein bisschen erzählen lassen, vielleicht kann man dann irgendwo aufspringen. Und dann wäre das Ziel, dass man die eine oder andere motivieren kann, wenn sie zugibt, dass sie leidet, dass sie das in einer psychologischen Beratung anschauen geht. Aber dafür muss man natürlich A, das Einsehen haben und B, eine psychologische, also das Einsehen und ein Wille, das zu machen, weil es mit Arbeit verbunden ist. Aber ich schicke solche, wenn ich kann, wenn ich sehe, dass es einen Sinn hat, gern zu einer Psychologin, die ich im selben Haus habe, also eine Gruppe von Psychologinnen. Die können dann mit ihr reden, warum sie diesen Knacks hat.

MS: Okay. Ähm, ich habe eine Auflösung. Es gäbe nämlich tatsächlich eine Diagnose dafür.

IP7: Sagen Sie es!

MS: Sie heisst Körperdysmorphe Störung.

IP7: [Ja, das hat man natürlich

MS: [Haben Sie das schon einmal gehört?

IP7: |**<< KDS unbekannt**|Nein, habe ich nicht gehört, aber das ist so.

|*KDS unbekannt >>*|MS: Klingt so ein bisschen danach, oder?

IP7: Nein, nein, das klingt, ist mir völlig –

MS: Also das hier wären die Kriterien. [MS legt die DSM-5-Kriterien hin.]

IP7: Körperdysmorphe Störung. Körperdysmorph. Das ist aber nicht, der Körper ist ja nicht dysmorph.

MS: Man meint, dass er es ist, oder?

IP7: Ja, ja aber für uns ist eine Dysmorphie, wenn etwas wirklich abartig ist. Und das ist sie nicht, sie hat eine psychisch Körperdysmorphe Störung.

MS: Das kommt jetzt nämlich hier gerade in den Diagnosekriterien. Und zwar geht es um eine übermässige Beschäftigung mit einem wahrgenommenen Makel. Und das heisst, er ist für Andere nicht erkennbar oder nur geringfügig vorhanden.

IP7: [Ja, ja, genau, das ist so.

MS: Das ist ganz wichtig.

IP7: Aber das müsste von mir aus gesehen in diese Diagnose rein. Weil es nicht eine Körperdysmorphe Störung ist. Eine Körperdysmorphe Störung, da wäre der Körper dysmorph. Es ist eine psychologische oder eingebildete Körperdysmoprhe Störung, weil es ist ja keine Störung.

MS: Mhm, aber ist natürlich schon im System der psychischen Störungen drin.

IP7: Ja, ja, eben, das muss man aber sagen. Dysmorpher Körper das ist ja, einer der wirklich dysmorph ist.

MS: Mhm, genau. Und was eben noch ist, sie halten sich mit sich wiederholenden Verhaltensweisen auf, die sehr viel Zeit brauchen. Also die ganze Zeit im Spiegel überprüfen, Dinge im Gesicht zurechtrücken, übermässige Körperpflege, Hüte tragen zur Tarnung und so weiter. Und sich immer wieder rückversichern bei den Leuten «Hey, sehe ich normal aus? Sieht das gut aus?» und vergleichen sich auch immer mit Anderen. Und das ist mit einem sehr hohen Leidensruck verbunden, eben wie bei unserem Fallbeispiel, sie können nicht mehr nach draussen, sie können keine Beziehung eingehen, Arbeitslosigkeit und so weiter. Und das ist einfach zur Abgrenzung, es handelt sich dabei nicht um eine Essstörung, das ist auch noch wichtig.

IP7: Ja, ja, es kann ja eine werden.

MS: Ja, man kann auch beides miteinander haben, genau.

IP7: Ja oder vom einen zum anderen.

MS: Genau. Und jetzt ist meine Frage einfach allgemein, ähm, haben Sie denn schon mal Erfahrungen gemacht, jetzt bei Ihnen in Ihrem täglichen Arbeitsleben, mit solchen Patientinnen? Kommt Ihnen da etwas in den Sinn?

IP7: Jede Menge.

MS: Jede Menge?

IP7: Bringen Sie mir eine junge Frau, aber nicht gerade fünfundzwanzig, sondern eben jünger.

MS: Jünger?

IP7: Also das Klientel, welches es betrifft, ist jünger. Weil mit zwanzig sollte man die nötige Reife haben, oder? Aber mit dreizehn bis sechzehn, siebzehn hat man die einfach nicht und dann wird einem von aussen gesagt oder auch von Anderen wie man auszusehen hat. Und diese Norm bestimmen nicht wir, die wird bestimmt von wem auch immer, heute würde man Influencer sagen, oder, wahrscheinlich? Die bestimmen, wie man auszusehen hat, die sagen den Anderen «Das ist ein Abartiger, der Dysmorphe», oder. Und dann wird sie ausgegrenzt. Und dann passiert das, was manchmal passiert. Wenn man dann die nötige, innere Sicherheit nicht hat, dann treibt man Leute zum Suizid, das ist theoretisch denkbar. Nur weil sie nicht so aussieht, wie man heute aussehen muss, aussehen sollte. Und das ist nicht nur das Aussehen, sondern auch das Anziehen, das Benehmen.

MS: Das heisst, dass Sie viele Patientinnen haben, die mit solchen Dingen zu Ihnen kommen?

IP7: Früher natürlich mehr, heute habe ich nicht mehr so junge. Aber ja, es gibt viele, leider, leider. Die nicht zufrieden sind mit ihrem Körper. Vielleicht nicht gerade in dieser grotesken

Ausführung aber. Es ist vielleicht eine von zehn Frauen zufrieden mit ihrem Busen, das ist leider so.

MS: Also das kommt oft vor?

IP7: Oft?

MS: Oft.

IP7: Oft, also ich kenne selten jemand, der sagt, es sei genau richtig. Also es gibt es natürlich schon nicht so selten aber die meisten haben etwas auszusetzen an der Nase.

MS: Ja. Was würden Sie denn sagen sind die Herausforderungen genau mit solchen Patientinnen?

IP7: Ja, da habe ich leider ein wenig resignieren müssen. Weil, man kann diesen Leuten nicht sagen, diesen Frauen, dass sie absolut in Ordnung sind. Weil sie das nicht so spüren. Sie finden einfach, dass es nicht so ist. Und ich möchte schon, manchmal gelingt es mir aber oft auch nicht, dass sie sich zum Beispiel den Busen operieren lassen oder dergleichen, das probiere ich prinzipiell, wenn daraus keine gesundheitliche Störung resultiert, ihnen auszureden, oder. Gerade eine Vergrösserung mit Implantaten. Weil wahre Schönheit kann man ja nur ausstrahlen, wenn man sie so wie man ist in Würde trägt. Weil wir alle werden älter, wir alle zerfallen gesundheitlich, oder, und niemand bleibt ewig schön. Aber eine schöne Ausstrahlung kann man eigentlich immer haben, auch im hohen Alter noch. Auch wenn man vollkommen mit Falten besetzt ist, oder. Ich meine PERSONENNAMEN2 oder ( ), die haben alle noch eine gewisse Ausstrahlung, oder? Oder der andere von «Der Pferdeflüsterer», der –

MS: [PERSONENNAMEN3?

IP7: PERSONENNAMEN3, der trägt seine Falten mit grösster Eleganz, oder. Aber ich meine, jeder andere würde sagen, der sieht aus wie ein völlig zerfallener Waschlappen, oder? Der besteht ja nur aus Falten. Aber man hat den Eindruck, dass er diese mit Würde trägt, ja für sein Alter. Oder PERSONENNAMEN4 auch, in meinen Augen. Oder es gibt natürlich auch Frauenbeispiele, grossartige Schauspielerinnen, wo man den Eindruck hat, dass die mit dem umgehen können. Ausgerechnet gerade Schauspielerinnen, die ja Influencer sind, oder. Aber dies übernimmt man dann nicht, es sind solche lächerlichen Gestalten, die jetzt, ich will den Namen nicht sagen, in der Zeitung herumgetragen werden, oder.

MS: Also würden Sie sagen, dass es schwierig ist, die Einsicht der Patientinnen zu gewinnen?

IP7: Ja, es ist schwierig, sie davon abzuhalten, wenn sie es im Kopf haben. Man kann immer einen Ansatz probieren und sagen «Von wo kommt diese Idee?», oder. Und es sagt sehr selten eine nachher, wenn sie wiederkommt und die meisten kommen wieder, und dann haben sie es halt gemacht und dann

MS: [also die Operation?

IP7: Ja, also ich sage ja nicht «Jetzt müssen Sie nicht mehr kommen», ich rate ihnen einfach davon ab. Es macht sie für mich nicht anders, ob sie einen Busen jetzt XL oder XS haben, das ist mir egal. Und ich bringe immer dieses lächerliche Beispiel: Beurteilen Sie den Mann auch nach der Länge seines Penis? «Nein, nein, was denken Sie?». Dann sage ich: «Ja aber, ist das ein riesiger Unterschied?». «Nein, nein das spielt mir keine Rolle, aber ich möchte es für mich machen, für meine Sicherheit». Ja das ist dann manchmal eine Leier und

dann sagen sie: «Ja, ich verstehe schon, dass Sie dagegen sind aber ich mache es für mich». Das ist dann halt so und dann sagen alle: «Ich bin so froh, dass ich es gemacht habe» ausser die paar wenige, bei denen es einfach in die Hose gegangen ist, oder. Das ist wie mit den Piercings, wenn es einen schönen Eiterabszess gibt und man da zwei Monate daran bastelt, bis es wieder gut ist und ein Kilo Antibiotika nehmen.

MS: ((lacht)). Ja, die nächste Frage wäre, ähm, wie ist Ihr Vorgehen, um mit diesen Herausforderungen umzugehen? Also da haben Sie schon gesagt, dass sie reden, versuchen, Ihre Sicht

IP7: [Versuchen, höflich, ja meine Sicht, ohne direktiv wirken zu wollen. Und sie probieren zu motivieren, zufrieden zu sein, so wie sie sind. Und, wenn sie dann natürlich direkt fragen, würde ich schon sagen, dass sie mit solchen Operationen gesundheitliche Risiken eingehen. A ist es eine Operation, eine Vollnarkose auf jeden Fall und immer auch mit Implantaten verbunden, also mit Fremdkörper, oder. «Ich will keinen Fremdkörper, ich will keine Spirale», aber zwei Gummibrüste, das darf's dann sein. Aus Silikon, das erwiesenermassen gesundheitliche Schädigungen haben können. Aber des Menschen Wille ist sein Himmelreich, oder? Das wissen Sie als Psychologin, oder?

MS: ((lacht)). Ähm, gut, also meine letzte Frage wäre noch, wenn Sie jetzt eine Patientin sehen, bei der Sie den Verdacht haben, die könnte jetzt so eine Störung wie wir sie vorhin angeschaut haben, haben, wie würden Sie da vorgehen?

IP7: Ich würde sie, das ist repetitiv, probieren zu motivieren, in dem ich ihr zuhöre, zu motivieren, ob sie genug Leidensdruck hat, um das psychologisch anschauen zu gehen. Und dann würde ich sie weiterweisen.

MS: Gut, kurz und knackig, sehr gut ((lacht)). Mein Teil wäre somit beendet.

IP7: Ja, oder was haben Sie erwartet?

MS: Wir erwarten nie etwas bei so Interviews, wir sind offen für alle Antworten ((lacht)).

IP7: Ja, Sie haben ja jetzt doch schon einige gehört, das wievielte bin ich?

MS: Das siebte.

IP7: Ja, also gut, dann haben Sie ja schon einen Haufen.

MS: Ja. Ja, Sie sind nicht der einzige, der das gesagt hat.

IP7: Ja, das ist natürlich ein Teil unserer Arbeit, oder, der psychologische Aspekt. Wir haben noch den somatischen Aspekt, da wird natürlich primär mal erwartet, dass wir die somatische Abdeckung machen und nicht die psychologische. Das ist für uns auch immer ein bisschen eine Herausforderung, weil wir nicht unbedingt immer gut ausgebildet sind. Wobei das einfach mehr Zeit braucht, als wir haben. Ja, das ist eine Ressourcenfrage. Man kann eine Libidostörung zum Beispiel nicht äh, die kommt meistens, wenn man die Türfalle schon in der Hand hat, oder. Übrigens ((lacht)), übrigens. Und dann ist man wirklich verkauft, also ich kann das nicht mit meinen Worten sagen, wann diese Maschine läuft, aber dann ist man dann echt am A-, wenn man die Türfalle schon in der Hand hält, Konzentration ist auch, «Übrigens, mit dem Mann geht es also auch nicht mehr so wie früher». Und dann gehen Welten auf. Das ist häufig. Weil mein Publikum, meine Klientel ist gewachsen mit mir, oder, kommen jetzt dann auch langsam in das Alter und dann nehmen diese Probleme zu oder. Verhütung ist nicht mehr das Problem, aber das.

MS: Okay, gut, danke vielmal.

IP7: Jetzt, gehen wir zur nächsten Vignette?

OD: Genau, ähm, zuerst mal ein paar Fragen ((lacht)). Ähm, was sind Ihre Erfahrungen mit Patientinnen, die Fragen stellen zur Ästhetik oder zur Normalität ihres eigenen Genitalbereichs?

IP7: (3) Nochmal von Anfang, nur die erste Einteilung, ich habe Frau PERSONENNAME5 schnell Guten Tag gesagt.

OD: ((lacht)). Kein Problem. Ähm, was sind Ihre Erfahrungen mit Patientinnen, die Fragen stellen zur Ästhetik oder zur Normalität ihres eigenen Genitalbereichs?

IP7: |**<< Labien**|Das betrifft bei mir eigentlich nur die grossen, also nur die Schamlippengrösse|*Labien >>*|, |**<< Medizinische Beschwerden**|welche manchmal ein Thema ist, die stört, sei es beim Biken oder sei es beim Reiten. Da sind wir natürlich in einem Grenzbereich zwischen Somatik und Psychologie|*Medizinische Beschwerden >>*|. Klar, riesengrosse Schamlippen ist ja ästhetisch, wobei das sehen ja nicht so viele Leute, aber da sind wir im Grenzbereich. |**<< Erwähnen OP**|**<< Sinnvoll**|Da gibt es somatisch echte Störungen, die man dann manchmal operativ angehen muss aber das ist eigentlich nicht meine Hauptfrage. |*Erwähnen OP >>*|*Sinnvoll >>*|**<< Keine Fragen** |Beim Rest habe ich keine Fragen, ausser es betrifft den somatischen Bereich aber psychologisch habe ich keine.

|*Keine Fragen >>*|OD: Okay. Und wie gehen Sie da vor?

IP7: |**<< Gespräch**|Ja, das sind längere Diskussionen, die man hat, |*Gespräch >>*|**<< Medizinische Beschwerden**|weil einerseits ist es ja eine spezielle Situation, es behindert einem im normalen Leben selber|*Medizinische Beschwerden >>*|, |**<< Scham**|es ist eine ästhetische Frage, sie genieren sich eventuell oder, wie wenn ein Mann das messen gehen würde. Aber sie genieren sich trotzdem vor sich selber und vor anderen Leuten, oder. |*Scham >>*|Und das andere, wenn sie natürlich ihren heissgeliebten Reitsport nicht ausüben können wegen dem, und|**<< Sinnvoll**| |**<< Überweisung an Chirurgie**|sie wegen dem einen Eingriff auf sich nehmen würden, dann muss man es auch machen, oder|*Überweisung an Chirurgie >>*|. |*Sinnvoll >>*|**<< Aufklärung OP**|Wir sind ja schliesslich da, den Leuten nicht zu sagen, was sie zu machen haben, sondern um ihnen zu helfen, wenn es geht. Und das geht, wenn man sie richtig orientiert, man muss sie orientieren, dass sie eine Anästhesie haben wird, dass es Narben gibt und dass nicht immer alles so schön aussieht, wie sie nachher meint, dass es aussehen müsse. Das ist einfach nicht möglich, man |**<< Kompliziert**|bringt das nie hundertprozentig symmetrisch|*Kompliziert >>*| hin und es gibt auch immer Narben. |**<< Überweisung an Chirurgie**|Narben sind immer Störfelder |*Aufklärung OP >>*|und|**<< Sinnvoll**| wenn man das sagt und sie will es trotzdem, dann müssen wir das wohl machen. Weil es ist |**<< Medizinische Beschwerden**|gesundheitlich ein Problem, das ist nicht nur reine Kosmetik. Und dann müssen wir halt.

|*Sinnvoll >>*|*Medizinische Beschwerden >>*|*Überweisung an Chirurgie >>*|OD: Fragen Sie manchmal bei Ihren Patientinnen nach, wie sie zu ihrem eigenen Genitalbereich stehen?

IP7: |**<< Fragen nicht nach**|Nein.|*Fragen nicht nach >>*|

OD: Und was sind, das haben Sie jetzt schon ein bisschen angesprochen, aber was sind Ihre Erfahrungen mit Patientinnen, die Schönheitsoperationen am Genitalbereich wollen?

IP7:|**<< Seltene OP**| Habe ich die wenigsten, |**<< Zufriedenheit**|**<< Labien**|nur so Labienverkleinerungen und die sind zufrieden.|*Zufriedenheit >>*|*Labien >>*| Aber das sind nicht viele, sonst habe ich niemanden mit Schönheitsoperationen, |**<< Andere**

**Operationen**|habe keine, so Schönheitsoperationen im weiteren Sinne, Verjungfräulich-Erneuerungsoperationen habe ich keine, weil da mache ich auch nicht mit.

|*Seltene OP >>*|*Andere Operationen >>*|OD: Ja. Wie sind Sie denn vorgegangen bei diesen wenigen Fällen, die Sie gehabt haben?

IP7: |**<< Gespräch**|Ja, ausdiskutiert.|*Gespräch >>*| Aber es sind dann viele, die sich nicht operieren und trotzdem durchs Leben kommen. Plötzlich arrangiert man sich damit.

OD: Also würden Sie dort auch eher versuchen, die Frauen davon abzuhalten?

IP7: |**<< Abratung von OP**|**<< Ernstnehmen**|Ja, das ist natürlich die Gratwanderung zwischen Abhalten und trotzdem Hilfe bieten zu wollen. |*Abratung von OP >>*|*Ernstnehmen >>*|**<< Individualität**|Man muss sich da halt durchschlängeln im Individualfall, man kann das nicht generell sagen. Jede ist individuell und das macht unser Leben ja zu so einem interessanten Bereich im menschlichen Bereich, weil wir alle so verschieden sind.|*Individualität >>*| Nur die Männer, die sind alle gleich. Macht euer Leben einfacher und meines ein bisschen komplizierter ((lacht)).

OD: ((lacht)).

MS: Soll ich das schreiben? ((lacht)).

IP7: Das können Sie ruhig schreiben, das sei ein Lieblingszitat von mir ((lacht)).

MS: ((lacht)). Das Lieblingszitat.

IP7: «Wir Frauen sind sehr verschieden aber wir Männer sind alle gleich.»

OD: Also, ich habe jetzt auch noch ein Fallbeispiel, wenn Sie das durchlesen könnten.

[OD legt Fallvignette 2 hin. IP7 liest Fallvignette 2 durch.]

IP7: Ja, ja. (3) ((lacht)). Da sind wir genau dabei, oder.

OD: Also, das wäre jetzt eben

IP7: [ein Beispiel, da eigentlich medizinisch nicht relevant ist.

OD: Ja.

IP7: |**<< Normalität betonen**|Ja. Also da beziehe ich klar Stellung ein. Und sage ihr halt, dass sie normal aussieht. |**<< Expertise nutzen**|Weil, das gesteht man mir dann zu, dass ich ein bisschen mehr Übersicht habe als sie, oder.|*Expertise nutzen >>*| Und damit sage ich ihr «Sie sind normal», Punkt. Und dann lasse ich sie damit. Weil das sage ich dann bestimmt, dass sie normal ist. Und ich gebe ihr den Rat, das zu akzeptieren. Und hoffe somit, dass das heilsam wirkt. |**<< Expertise nutzen**|Weil jemandem, der dieses Business 30 Jahre lang macht, kauft man dies wahrscheinlich ab, wenn man nicht ganz verbohrt ist.|*Expertise nutzen >>*| Und wenn es so ist, dann sage ich es. Klar, eindeutig, sie ist normal. Und sage dieses Wort noch ein paar Mal und gebe ihr den Rat, das so zu akzeptieren. Aber es soll ihr repetitiv in den Kopf gehen, dass ich ein paar Mal «normal» gesagt habe, damit sie es auch annimmt oder aufnimmt, das Wort. So wie es in der Reklame ja auch gemacht wird. Dass man möglichst in 21 Sekunden zehn Mal das Produkt zeigt. Dann probiere ich eben ihr in 21 Sekunden zehn Mal «normal» zu sagen ((lacht)).

|*Normalität betonen >>*|OD: Also einfach nur zur Auflösung das wäre ein Beispiel von

Körperdysmorpher Störung im weiblichen Genitalbereich

IP7: [vom unteren «Stübli»

OD: Genau. ((lacht)).

IP7: Also eine psychologisch Körperdysmorphe?

OD: Genau.

IP7: |**<< Verzerrte Wahrnehmung**|Eine Eingebildete. ((lacht)).

|*Verzerrte Wahrnehmung >>*|OD: Ja, genau. Die Frage ist, welche Erfahrungen haben Sie mit Frauen, die jetzt solche Symptome zeigen?

IP7: |**<< Subklinische Unzufriedenheit**|Eigentlich gute. Wenn sie wirklich normal sind, akzeptieren sie es, die allermeisten. Ich habe jetzt noch niemanden gehabt, die immer darauf zurückgekommen ist und wieder darauf zurückgekommen ist.|*Subklinische Unzufriedenheit >>*| |**<< Expertise nutzen**|Weil, die gehen irgendwie ein bisschen erlöst von dannen. Weil da muss man die Autorität ein bisschen wirken lassen. Wenn das eine 25-jährige Assistentin sagt, oder eine Unterassistentin, das hat nicht das gleiche Gewicht, wie wenn ich das sage. |*Expertise nutzen >>*|**<< Normalität betonen**|Weil das beinhaltet ja einerseits die Aussage, dass sie normal ist und |**<< Abratung von OP**|dass niemand, der einigermassen alle Tassen im Schrank hat sie operieren würde und da etwas verändern würde.|*Abratung von OP >>*| Es geht ja ein bisschen um das. Und dann sage ich auch, ich kenne niemanden und würde auch niemanden finden, der da etwas machen würde. Einfach weil es normal ist und noch mal normal ist. Und ich kenne niemanden in meinem seriösen, möglichst seriösen ((lacht)) Umfeld, der so etwas operieren würde, wenn sie ( ) machen würden. Nein, ich kenne niemanden, oder.|*Normalität betonen >>*| |**<< Verstümmelung**|Es gibt schon Spezialistinnen oder Spezialisten für Rekonstruktionen von Verstümmlungen, wie man es im afrikanischen Teil ab und zu sieht, aber das hat damit ja nichts zu tun so direkt, oder. Die sind wirklich verstümmelt, das ist etwas anderes.|*Verstümmelung >>*| |**<< Psychische Probleme im Hintergrund**|Und das ist ein psychologisches Problem, das ich eigentlich lieber, ob das überhaupt geht, dass müsst ihr mir sagen, |**<< Wenig Eingehen**|einfach zur Seite schieben und sagen «Nein, Sie sind normal» und fertig. «Akzeptieren Sie das, bitte». Da biete ich auch keine psychologische Beratung an.|*Psychische Probleme im Hintergrund >>*| Wenn jemand normal ist, braucht er auch keine psychologische Beratung, dann soll er das «gopfriedstutz» akzeptieren, soll sich mal einen Ruck geben und etwas anderes machen. Aber ((lacht)) das ist vielleicht ein bisschen arbiträr, das Ganze ((lacht)). Geht das?

|*Wenig Eingehen >>*|OD: Ich glaube eher nicht ((lacht)).

IP7: Eher nicht?

OD: Es kommt darauf an, wie tiefgründig, dass es ist aber, ja.

IP7: Hilft das nicht, wenn man diesen Frauen sagt, sie seien normal?

OD: Ja, ich denke, es kommt dann auf den individuellen Fall drauf an. Also jetzt bei ihr kann man es schwierig sagen, wie tiefgründig es ist. Aber es kann schon sei, dass viel mehr dahinter lieg.

IP7: |**<< Psychische Probleme im Hintergrund**|Ja, natürlich, wenn etwas mehr dahinter liegt, ist es ein Symptom. Aber dann hat sie vielleicht auch noch ein anderes Problem, oder. |**<< Traumatische Erfahrungen**|Klar kann man dann noch suchen gehen, wenn sie eine unglückliche Defloration oder Missbrauch entwickelt, dann ist es nicht nur dieses

Problem.|*Traumatische Erfahrungen >>*| Dann hat diese Person noch andere psychologische Auffälligkeiten und nicht nur ihre Schamlippen, die sie beschäftigen. Dann hat sie sonst noch einen «Chnurz». Und dann darf man schon nicht vergessen, dass es auch noch eine psychosomatische Auswirkung sein könnte oder im weiteren Sinne.|*Psychische Probleme im Hintergrund >>*| |**<< Subklinische Unzufriedenheit**|Natürlich, die ist normal, aber einfach eine psycho-, normale Dysmorphie, Störung ((lacht)). Nein, es könnte natürlich schon etwas dahinterstecken aber ich denke eben nicht immer an das Schlimmste. Weil die, die wirklich leiden, die kommen dann mit anderem. Wenn da wirklich etwas gewesen ist, im Sinne von einem Missbrauch zum Beispiel. Aber die haben dann schon mehr Bauchweh als Schamlippengrössenprobleme ((lacht)). Das ist dann häufiger.

|*Subklinische Unzufriedenheit >>*|OD: Gut, ähm. Wie gross schätzen Sie das Interesse an einer Fortbildung zur Körperdysmorphen Störung im Fachbereich Gynäkologie ein?

IP7:|**<< Grosses Interesse an Fortbildung**| Als eher gross. |*Grosses Interesse an Fortbildung >>*|**<< Untergebildet**|Psychologie ist in der Gynäkologie leider zu wenig gewichtet. Ich meine, man könnte, nachdem man einen operativen Gynäkologen kreiert, auch einen sprechstundenpsychologischen Aspekt kreieren können. Also eine Fachspezialistin oder -spezialisten für Sexualstörungen, für Körperdysmorphe Störungen in diesem Fall, wäre durchaus angebracht. Ich wäre sehr froh und das gibt es nur, wenn man jemanden kennt, der sich damit befasst, ein bisschen, dass man sie weiterweisen kann. Aber Spezialistinnen, ausgebildete Spezialistinnen für beispielsweise Libidostörungen oder eben solche dysmorphe Störungen gibt es nicht und das wäre ein echtes Bedürfnis, das kann ich euch also sagen. Und es-, auch eine interessante Mischung. Wir müssen das alles lernen, «learning by doing». Wir gehen in die Sprechstunde, sind ausgebildet operativ, also wir können alles zerschneiden und wieder zusammennähen aber sind dann völlig überfördert am Anfang mit diesen Problemen und müssen das alles irgendwie hinkriegen. Ich bin jetzt am Ende meiner Karriere, meiner ruhmlosen Karriere, aber man hätte viel Positives bewirken können, wenn man von Anfang an auf solche Probleme hätte eingehen können. |**<< Vertrauensperson**|Weil diese kommen vom ersten Tag an, das kann ich Ihnen garantieren. Am ersten Tag kommen die ersten psychologischen Probleme, die beim normalen Körper vorkommen, völlig somatisch normal sind aber psychisch herausgefordert.|*Vertrauensperson >>*| Also da hacke ich mir die rechte Hand ab, wenn das nicht so ist. Am ersten Tag kommen sie, erst recht am ersten Tag, weil dann kommen sie von anderen Ärzten, die ihnen nicht helfen konnten, kommen sie zum Neuen, der kann's jetzt, der ist neu, der ist jung, der kann das. Jedoch hat er überhaupt keine Ahnung, er hat erst recht keine Ahnung, weil er hat ja noch gar keine Erfahrung gemacht und hat eben keine Ausbildung. Und deshalb würde ich diese Frage mit einem Ausrufezeichen Ja bestätigen. Haben das die anderen sieben auch so gesagt?

|*Untergebildet >>*|OD: Nicht so ausgeprägt, aber schon.

IP7: Also, da ist ja mehr das alte Problem. Ein Chirurg ist ein Handwerker, wenn er noch ein bisschen psychologisches Geschick hat, ist er ein guter Handwerker oder er verkauft sich gut und macht die Leute nicht glücklicher. Das andere gibt es eben auch, seelisch ist die Baustelle grösser als die Gebärmutter. Das ist meine Erfahrung.

OD: Wie gross würden Sie das Interesse oder den Nutzen an Hilfsmitteln zur Diagnostik der Körperdysmorphen Störung einschätzen, also so

IP7: [Was für Hilfsmittel?

OD: So Screeninginstrumente, Fragebögen, solche Sachen.

IP7: |**<< Mittel Interesse and Diagnostik**|Ja, das kann in dem Sinne hilfreich sein, dass es Jüngeren die Normalität besser definieren kann. |*Mittel Interesse and Diagnostik >>*|**<<**

**Objektivieren**|Dass man den Leuten sagen kann «Schauen Sie, wir haben einen Fragebogen gemacht, und Sie auch da im Fragebogen normal oder wir messen es als normal, also ist es normal». Und dann kann man den Leuten ihre Normalität quasi einprügeln. Das ist jetzt psychologisch nicht ein wahnsinnig geschickter Aspekt aber ich würde das so machen.|*Objektivieren >>*| |**<< Expertise nutzen**|Und die Autorität so wirken lassen. Weil genau so, wie einem diese Autoritäten sagen, wie man auszusehen hat, kann man auch auf der positiven Seite sagen «Sie sind normal im Aussehen».|*Expertise nutzen >>*| Wie die Influencer sagen, sie sind nicht der Norm entsprechend, kann man auch sagen, sie sind der Norm entsprechend. Mit der gleichen Macht, wenn man sie hat. Wir haben sie eben, weil ja, man zieht sich nicht vor so vielen Leuten aus im Leben, vollständig nackt nicht ganz nackt aber. Ja, nicht so viel, im Normalfall ((lacht)).

OD: Und wie gross schätzen Sie denn das Interesse an Hilfsmittel für die Beratung spezifisch für Frauen, die Symptome der Körperdysmorphen Störung des Genitalbereiches zeigen?

IP7: |**<< Grosses Interesse an Hilfsmittel**|Ja, das geht ins selbe, ist die gleiche Frage wie vorher. |**<< Grosses Interesse an Fortbildung**|Ich würde eine Ausbildung in diese Richtung sehr begrüssen.|*Grosses Interesse an Hilfsmittel >>*| Dass man da, sei es, dass man da Praxiskurse macht, also für diejenigen, die in die Praxis gehen, in die Sprechstunde, diejenigen die an der Front sind, oder sei es ein Spezial-, so ein Subgruppentitel für operative oder einfach auch Sexualtherapie im weiteren Sinne oder psychologische Gynäkologie, ist ja Wurst, wie man dem dann sagt, die diesen Schnittpunkt besser abdeckt. Weil wir sind somatisch orientiert.|*Grosses Interesse an Fortbildung >>*| Klar, wir haben natürlich viel Somatik, wir müssen Kinder beurteilen im Bauch und möglichst hundertzehnprozentig sagen können, dass das Knöpfchen normal ist und das ist eine grosse Herausforderung. Aber das ist eben auch eine und wird es mit zunehmendem Alter noch mehr. Weil es kommen mehr psychologische Probleme zu den alten Knaben als zu den jungen. Man zieht sich seelisch auch lieber vor einem autoritären Typ aus als vor einem Kollegen. Also das ist meine Erfahrung, ich denke, dass das stimmt. Aber ähm, auf dem gleichen Level geht das schwieriger. Also wenn man sich an eine Autorität lehnen kann, also manchmal sucht man auch den Kumpel, aber das funktioniert nicht. Der Arzt ist nie ein Kumpel. Es muss immer eine Führung sein, eine fachliche oder menschliche oder autoritäre Führung. Sonst geht man nicht zum Arzt, wenn er mit einem Kumpel reden will, für das hat er das Telefon.

OD: Ja, wir wären -

IP7: Am Ende.

OD. Ja am Ende, ausser Sie wollen noch etwas hinzufügen?

IP7: Ich habe glaube, ich genug geredet.

OD: Gut.

Interview mit: IP8
Datum des Interviews: 04.06.2019
Dauer: 22 min
Anonymisierte Begriffe: keine
IP-Nummer: 8

MS: Mit welchen psychischen Problemen kommen denn Patientinnen zu Ihnen in die Praxis oder in die Klinik?

IP8: In die Klinik. Psychische Probleme |**<< Restkategorie Störungen**|meistens ähm sekundär oder, aufgrund chronischer Schmerzsyndrome im Beckenbodenbereich, sei es nach Geburten oder ähm, operativen Eingriffen|*Restkategorie Störungen >>*| und ähm |**<< Reaktionen auf schwere Belastungen und Anpassungsstörungen (F43)**|weiters psychische Probleme jetzt ähm reaktiv beispielsweise oder hier im Brustzentrum insbesondere nach Krebserkrankungen|*Reaktionen auf schwere Belastungen und Anpassungsstörungen (F43) >>*| |**<< Restkategorie Störungen**|oder ähm, aufgrund familiärer Situationen oder die häufig dann sich die Frauen schon gegenüber der Frauenärztin eher öffnen oder. Weil wir natürlich solche Fragen auch direkt ansprechen. Sexualität beispielsweise oder Körperempfinden,|*Restkategorie Störungen >>*| genau.

MS: Was würden Sie sagen, welche Rolle spielt denn ähm die Zufriedenheit mit dem äusseren Erscheinungsbild bei Ihren Patientinnen?

IP8: Extrem. Oder, es gibt ja alle unterschiedlichsten Körperformationen und die Zufriedenheit korreliert nicht mit dem Schönheitsideal ((lacht)). Aber was wir insbesondere bei unseren ähm, Frauen mit Brustkrebs sehen oder dass die zunehmende Wichtigkeit der Rekonstruktion oder, dass die körperliche Rekonstruktion auch eine psychische Wiederherstellung ist, oder. Ähm, wir führen nie mehr ausser bei sehr alten Patienten oder auf ausdrücklichen Wunsch Mastektomien durch, das heisst komplette Amputationen der Brust, wir rekonstruieren eigentlich immer direkt oder führen in den meisten Fällen, sagen wir bei zwei Drittel bis achtzig Prozent eine ähm, eine brusterhaltende Operation durch.

[Kurzer Unterbruch aufgrund eines Telefonates.]

IP8: Sorry, ähm, nimmt's wieder auf?

MS: Ja.

IP8: Also und eben, dass wir Rekonstruktionen machen, oder. Und ähm so die Frauen wirklich, das ist belegt in Studien oder, dass die psychische, ähm, Zufriedenheit und auch die psychische Integrität wieder vermehrt hergestellt ist, dass die Lebensqualität deutlich verbessert wird, oder. |**<< Labien erwähnt**|Dass ist bei Brusteingriffen natürlich offensichtlicher als bei Genitaloperationen, die im Bauchraum sind oder in der Scheide drin, die man nach aussen nicht sehen kann.|*Labien erwähnt >>*|

MS: Okay, ich habe jetzt eine Fallvignette für Sie. Ich würde Sie bitten, diese kurz ähm, durchzulesen.

[MS legt Fallvignette 1 hin. IP8 liest Fallvignette 1 durch.]

MS: Gut? Ja. Ähm, welche psychische Störung oder welche Probleme fallen Ihnen bei diesem Fallbeispiel ein?

IP8: Ja es sind, sind, was stellt sich die Frau vor unter der Norm, oder. Was sind, ist für sie

normal, und es ist, es ist wirklich nicht nur, sind nicht nur die Brüste, sondern auch die Haare, die Nase, also es ist so ein bisschen körperübergreifend die Problematik, oder, was wir häufig sehen. Also es zählt denn, dass die Frau nicht nur mit der Brust unzufrieden ist oder mit den zu grossen kleinen Labien, also den Schamlippen, sondern dass es da mehrere Orte gibt, oder. Woher diese, das ist wahrscheinlich multifaktoriell bedingt oder, woher diese ähm, Probleme kommen. Kommen sie von einem Schönheitsideal, sind sie medial gesteuert oder ähm, |**<< vermindertes Selbstwertgefühl und Selbstvertrauen**|hat sicher mit dem Selbstvertrauen zu tun, oder, und mit dem Umfeld natürlich auch|*vermindertes Selbstwertgefühl und Selbstvertrauen >>*|.

MS: Welche Stellen sind Ihnen besonders ins Auge gesprungen?

IP8: Ähm, (3). Ja das dauernde oder, das heisst, es ist ähm, von morgens bis abends wahrscheinlich beschäftigt sie das, jede Sekunde oder, es ist schon sehr, sehr häufig ((lacht)) und es ist nicht nur ein Adjektiv, wie's beschrieben wird, sondern es ist gross und krumm, es ist klein und hängend, also es sind immer, ist immer noch, nicht nur ein Problem, womit sie nicht zufrieden ist. Und auch der Druck von aussen wahrscheinlich, oder, sie ähm, «was denken die anderen über mich?» oder, es gibt ja viele, die nicht ihr eigenes Leben leben sondern das der Anderen ((lacht)) oder der Vorstellung der anderen. Bestätigung suchend, oder, bei der Mutter.

MS: Wenn jetzt diese Patientin zu Ihnen kommen würde, was würden Sie sich denn noch für Informationen von ihr wünschen?

IP8: Also sie kommt zu mir wegen ihren ähm, gesundheitlichen Problem, das sie korrigiert haben möchte, oder?

MS: Also sie kommt genau so, wie hier dargestellt mit diesem Anliegen, ihr Äusseres gefällt ihr nicht.

IP8: Ja wichtig ist natürlich die ganze Geschichte der Patientin, oder, was hatte sie bisher für operative Eingriffe, wie sind die familiären Verhältnisse, lebt sie in einer Paarbeziehung, ist der Freund genau so unzufrieden mit dem Aussehen oder so. Und ähm, das sind lange Gespräche, oder. Ich meine hier eine Ope-, hier irgendwo beginnen operativ zu korrigieren, das wird nicht in einer glücklichen Patientin enden, oder, das ist, wenn alles nicht passt, oder. Manchmal kommt eine Frau mit einer Brustunterentwicklung, die hat tubuläre Brüste und ähm, das ist eine Fehlentwicklung der Brust oder, die muss man ernst nehmen, auch wenn sie sechzehn ist, oder. Ähm, aber ähm, da muss man ganz gut prüfen und das Gespräch führen einfach um zu sehen, wie schwerwiegend das Problem ist oder ob es wirklich etwas gibt, das operativ korrigiert werden muss oder eben gesprächstherapeutisch, genau

MS: Mhm. Ich habe eine kleine Auflösung davon. Und das Fallbeispiel das wäre ein Beispiel für eine Körperdysmorphe Störung.

[MS legt die DSM-5-Kriterien hin.]

MS: Haben Sie davon schon mal gehört?

IP8: |**<< KDS bekannt**|[Jawohl, ja, mhm.

|*KDS bekannt >>*|MS: Genau, also eben, es geht um die übermässige Beschäftigung mit einem wahrgenommenen Makel, das heisst, er ist für andere eben nicht erkennbar, oder nur geringfügig vorhanden. Und eben, sie vergleicht sich die ganze Zeit mit anderen, ähm, schaut sich vor dem Spiegel an, ähm, hat sehr grosses Leiden, ist natürlich beeinträchtigt, sozial, beruflich und so weiter. Und das ist einfach das Kriterium, das nicht erfüllt sein darf,

eine Essstörung. Weil es um Körperfettverteilung geht, genau. Also wenn Sie das jetzt mal gehört haben, ähm, haben Sie denn schon Erfahrungen gemacht mit solchen Patientinnen, die jetzt solche Symptome zeigen?

IP8: Ja, die sind gar nicht so selten. Häufig kommen sie übers Brustzentrum, das sind weniger die Leute, die über die gynäkologische, allgemeine Sprechstunde kommen, das ist wie Zahnarzt, man möchte den Abstrich und dann möchte man wieder zwei Jahre Ruhe oder, aber ähm, die Brust ist schon etwas sehr Zentrales bei den Frauen und dann, wenn wir solche Frauen sehen, dann sehen wir sie im Brustzentrum.

MS: Das heisst sie kommen mit normal aussehenden Brüsten-

IP8: [Genau.

MS: Und sind nicht zufrieden.

IP8: Genau, sind nicht zufrieden, möchten eine Korrektur und dann ist da aber noch «Ja, wurden Sie schon operiert?» «Ja, ich habe mir die Nase korrigieren lassen, ich habe dieses und jenes korrigieren lassen» ((lacht)). Dann ähm, fällt das schon auf, dass das so eine Körperdysmorphe Störung sein kann.

MS: Ja, also Sie kennen das?

IP8: Kennen, ja.

MS: Was würden Sie sagen, wie oft sehen Sie eine Patientin?

IP8: Zwei mal pro Jahr.

MS: Zwei mal pro Jahr?

IP8: Genau. Und ich mach an zwei Tagen die Woche die Sprechstunde.

MS: Ja, okay, mhm. Ähm, dann passt diese Frage gut, ähm, auf welche Herausforderungen treffen Sie denn mit-, also im Kontakt mit solchen Patientinnen?

IP8: Ja die Herausforderung, die Patientin, ihr eigenen, ihr eigenes Körperbild in ein anderes Licht zu rücken, das ist sehr schwierig, oder. Ich mein wir kennen die Leute, wir haben dreissig Minuten Termin und die bringen ihr ganzes Leben mit, oder. Also das ist schwierig, da ähm, weiss ich auch nicht, ob wir die richtigen dann sind, die das aufarbeiten sollten oder, wir sollen die Frau insbesondere beruhigen, manchmal hilft es, andere Brüste zu zeigen, genau und zu vergleichen, also ja, nicht ähm, mit Photoshop modifizierte Brüste, sondern wirklich real life, oder. Und auch das Körperbild und die Brustform, was ist normal, was ist nicht normal, sie vielleicht auch wirklich ernst zu nehmen und zu sagen «Ja, da haben Sie Recht in diesem Punkt, aber in diesem Punkt haben Sie nicht Recht, das sind die normalen Brustmasse», da kann man auch oder, so das gilt als schön, oder, es hängt nicht, oder die Brustwarze ist nicht zu gross und so, dass sie wie diesen Blick bekommen «Ah, das ist ja eigentlich gar nicht so abnorm, wie ich das wahrnehme».

MS: Okay. Und fehlende Einsicht, ist das auch ein Problem, das Sie antreffen?

IP8: Ja, ob die immer ehrlich sind oder, wenn man sie so zum ersten Mal sieht und berät und, ist noch schwierig einzuschätzen, ob sie das gleich einsehen oder ob man einen Gedankenanstoss gemacht hat, dass sie in eine andere Richtung zu denken beginnen, ist noch schwierig, wenn man sie nur einmal sieht. Weil wenn solche Probleme sind, oder so schönhei- ästhetische Probleme, dann schicken wir sie schon zu den Plastikern, die sehen

sie dann im Langezeitverlauf.

MS: Okay, Ähm, die zweite Frage ist ein bisschen ähnlich und zwar, wie ist Ihr professionelles Vorgehen, um mit diesen Herausforderungen umzugehen?

IP8: Eben, sicher eine ganze medizinische Anamnese durchzuführen, die Patientin ernstnehmen und auch aussprechen zu lassen, die Sorgen und Ängste ansprechen und auch wissen, was ist sie für ein Typ, oder. Ist sie ganz pragmatisch oder eben eher nicht, meistens eher nicht, oder. Und ähm, ja sich halt da ein bisschen einzufühlen, wie tickt sie, wie denkt sie, oder. Wenn sie ihren eigenen Stil haben, wollen ein Möbel verkaufen, das ihnen nicht gefällt, das wird nie funktionieren, oder. Man muss sie ein bisschen auf die andere Seite stellen, das ist so ein bisschen das. Und dann ähm, meistens einen medizinischen Teil, damit die Leute sehen, sie werden ernst genommen, man macht einen Ultraschall, es sieht alles gut aus, man macht Fotos, man misst was aus, oder, und am Schluss kam man das von der rein medizinischen Seite her dann tiefer gehen und sagen, es sieht eigentlich alles in Ordnung aus, ja, ein bisschen so, auch Ernstnehmen ist das wichtigste, oder weil sie sind vielleicht die erste Anlaufstelle, wo sie sich offenbaren oder ihrem Problem öffnen.

MS: Ja, mhm. Ähm, genau, die letzte Frage haben Sie eigentlich schon beantwortet, ähm, wie Sie vorgehen würden, wenn Sie jetzt merken, dass es wahrscheinlich nicht ähm, also dass das Aussehen normal ist. Das was Sie eigentlich jetzt gesagt haben. Sie haben aber gesagt, Sie sehen diese Patientin nur einmal, schicken Sie sie dann woanders hin oder-

IP8: [Ja, wenn es nur um ästhetische Korrekturen geht, oder, dann zu dem plastischen Chirurgen. Und ähm, was noch so ein bisschen typisch ist, ist wenn sie mit der Mutter kommen, oder. Das ist auch etwas, das man dann versucht, oder die Chance besteht, dass dann die Mutter zwar beim Erstgespräch dabei ist aber wenn es um die Untersuchung geht, haben die häufig abgemacht, dass die Mutter dann rausgeht und das ist der Moment ((lacht)), indem man die Patientin wirklich dann für sich alleine hat ohne die Nabelschnur ((lacht)). Und dann öffnen sie sich meistens.

MS: Mhm, okay. Ähm, haben Sie schon mal an Psychologen weitergeleitet?

IP8: Ja, wir arbeiten ständig mit Psychologen zusammen, insbesondere mit der Psychoonkologie, bei unseren Brustkrebspatientinnen oder ähm, wir haben auch ein psychologischen Dienst für chronisches Schmerzsyndrom, ( ) pain syndrome, so sind so Spezialgebiete, wo die Leute austherapiert sind und dann an diesem Board, an diesen Konferenzen dabei sind aber die Psychologinnen sind bereits bei uns am Tumorboard dabei, speziell für die Krebspatientinnen und wir bieten aktiv jeder Patientin mit Brustkrebs das an und ähm, die Frauen die bisher gekommen sind mit dieser dysmorphen, Körperdysmorphen Störung, die haben häufig schon einen Psychiater.

MS: Ah, das haben sie sogar schon, okay.

IP8: Mhm.

MS: Ja, das wollte ich nämlich noch fragen, was Sie mit diesen zwei Frauen pro Jahr machen.

IP8: Fragen sie ja nicht immer, aber frag sie «Haben Sie noch mehr Ärzte?», «Möchten Sie, dass ein Brief versendet wird?» dann sieht man, ah, das ist ein Psychiater oder «Was haben Sie für Medikamente?» ja da kommt, genau, dann kommt man drauf. Man fragt die Leute nicht direkt so «Haben Sie einen Psychiater?», dann denken sie, «Das ist schon die zehnte, die kommt und sagt, ich werde auf die Psyche reduziert» und das ist nicht ganz fair.

MS: Aber ist also interessant, dass Sie sagen, dass die meisten schon jemanden haben, ja. Okay, gut, danke.

OD: Gut, ähm, was sind Ihre Erfahrungen mit Patientinnen, die Fragen zur Ästhetik oder eben zur Normalität ihres eigenen Genitalbereichs stellen?

IP8: |**<< Kultur**|Da, das ist wirklich ganz interessant, weil das geht ja wirklich zurück auf die Kultur. Also es ist ja von der Kultur her nur schon interessant, oder. |**<< Intimrasur**|Früher hat man, oder die Europäerinnen haben sich im Genitalbereich sehr wenig rasiert, oder. Also wie ich Gynäkologie begonnen habe war fast jede Frau behaart und jetzt sind knappe zwei Prozent behaart.|*Intimrasur >>*| Und ähm, Südländerinnen, Türkinnen oder eben die südlichen Regionen die sind, die waren schon immer gewachst und komplett rasiert. Auch Afrikanerinnen, die sind immer komplett rasiert und durch das wird das viel auffälliger, das Genital wurde gar nicht so wahrgenommen vorher, oder, da waren Haare drum rum. |**<< Labien**|Und äh, es ist normal und physiologisch, dass die kleinen Schamlippen ein bisschen die grossen Schamlippen überragen, oder und für viele ist das unästhetisch, oder.|*Labien >>*| |**<< Verstümmelung**|Ähm, jetzt woher kommt die Genitalbeschneidung, oder? Weil's unästhetisch aussieht, weil die gedacht haben, das ist eine Vermännlichung des weiblichen Genitals und äh, führen diese Verstümmelungen durch, oder, in weiten Teilen Afrikas, oder. Das ist so ein bisschen der kulturelle Hintergrund,|*Kultur >>*| das ist nicht rein, das ist schmutzig, sieht nicht gut aus. |*Verstümmelung >>*|**<< Zunehmend**|Und ähm, aufgrund des Rasierens ist dann auch bei uns so ein bisschen der Trend gekommen, das sieht nicht schön aus, das sieht aus, keine Ahnung, oder, wie das verglichen wird, oder einfach nicht ästhetisch und |**<< Erwähnen OP**|dann kommen Anfragen zu Reduktionsplastiken, die Labienreduktionsplastiken, oder das wegschneiden zu lassen|*Erwähnen OP >>*| und ähm, das ist wirklich oft die Frage, |*Zunehmend >>*|oder, was die Frauen stellen, so «Ist das normal? Sind sie zu gross?» oder manchmal sind sie so gross,|**<< Medizinische Beschwerden**|**<< Sexualität**| dass es beim Sex stört, dass die reingezogen werden, oder, da ist's 'ne andere Sache, aber das war früher nie das Thema und jetzt häufiger.

|*Medizinische Beschwerden >>*|*Sexualität >>*|OD: Ja, okay. Also, wie häufig würden Sie sagen?

IP8: |**<< Viele Fragen** |**<< Wöchentlich**|Die Anfragen bei uns, sicher zwei, drei pro Woche, jetzt in der Sprechstunde.|*Viele Fragen >>*|*Wöchentlich >>*| |**<< Abratung von OP**|Aber wir lehnen ab, wir raten davon ab und das macht auch die Schweizer Gesellschaft für Gynäkologie und Geburtshilfe, dass die wirklich davon abraten hier Narben zu setzen in diesem hochsensiblen Bereich, oder. Wenn da mal Narben gesetzt sind, dann ist die Empfindung nicht mehr dieselbe, irgendeinen Sinn hat das, oder, und da kosmetische Eingriffe durchzuführen, das ähm, lehnen wir hier ab.|*Abratung von OP >>*| |**<< Aufklärung OP**|Also das Befürworten, |**<< Überweisung an Chirurgie**|wenn eine Frau darauf insistiert, dann wird das schon gemacht,|*Überweisung an Chirurgie >>*| aber äh, nach einem guten und aufklärenden Gespräch häufig so auch akzeptiert.

|*Aufklärung OP >>*|OD: Ähm, ja, was ist denn Ihr professionelles Vorgehen, wenn solche Fragen kommen?

IP8: |**<< Vielfalt**|**<< Normalität betonen**|Auch wieder, |**<< Bereits in Benutzung**|wir haben eine ganz interessante, äh, Gips-,äh, ein Gipsposter. Da wurden Gipsabdrücke von ähm, weiblichen Genitalien gemacht und da gibt es alles was die Welt hergibt, von kleinen Schamlippen über sehr grosse,|*Bereits in Benutzung >>*| einfach ganz unterschiedlich, oder, und dann sagt man, das ist alles normal.|*Vielfalt >>*|*Normalität betonen >>*| |**<< Intimrasur**|Also so wirklich vergleichend oder, weil auch das Anatomiebuch hilft oder, dass das völlig normal ist, dass die, dass die kleinen Schamlippen grösser sind und dass das halt äh, so zur Geltung kommt, wenn man das Genital rasiert.

|*Intimrasur >>*|OD: Fragen Sie manchmal aktiv bei Ihren Patientinnen nach, wie sie zur Ästhetik ihres Genitalbereichs stehen?

IP8: |**<< Fragen nicht nach**|Nein, nein.

|*Fragen nicht nach >>*|OD: Ähm, dass haben Sie schon ein bisschen beantwortet, aber ich stelle
die Frage trotzdem. Was sind Ihre Erfahrungen mit Patientinnen, die jetzt direkt nach Schönheitsoperationen ihres Genitalbereichs fragen?

IP8: |**<< Abratung von OP**|Eher ablehnen oder abraten davon. Weil Vernarbungen in diesem Bereich, die machen keinen Sinn, oder, also, mhm.

|*Abratung von OP >>*|OD: Gut, dann habe ich jetzt auch ein Fallbeispiel.

[OD legt Fallvignette 2 hin. IP8 liest Fallvignette 2 durch.]

OD: Also das wäre jetzt ein Beispiel einer Körperdysmorphen Störung des weiblichen Genitalbereichs. Ähm, also es ist keine separate Diagnose so zu sagen, aber bei der KDS kann jedes Körperteil betroffen sein und hier wär's eben der Genitalbereich.

IP8: |**<< Beziehung**|Es ist interessant, was der Freund dazu sagt, den stört es nämlich meistens nicht ((lacht)).

|*Beziehung >>*|OD: Genau ((lacht)). Ja, was sind Ihre Erfahrungen mit solchen, oder Patientinnen, die so ähnliche Symptome zeigen?

IP8: |**<< Sexualität**|Ja sie haben schon dann häufig wirklich ein Problem mit dem Geschlechtsverkehr, oder, |**<< Scham**|wenn man sich so schämt, weil Sexualität ist multifaktoriell, dass man das organische, die Veranlagung dafür oder, dass man die anatomischen Strukturen hat, dies dazu braucht, orgasmusfähig zu sein, aber äh, wenn man sich nicht hingeben kann|*Scham >>*|, da gar nichts funktioniert oder, das ist wenn so eine Störung vorliegt, die haben kein gutes Sexleben oder. |**<< Abratung von OP**|Da muss, operative Korrekturen helfen da nicht.

|*Sexualität >>*|*Abratung von OP >>*|OD: Ja, wie gehen Sie dann vor, wenn solche Patientinnen kommen?

IP8: |**<< Überweisung Psychotherapie**|Wir haben eine Sexualtherapeutin, die wir sie über-, wo wir sie überweisen, oder, dann äh, das zu besprechen, sich selber kennenzulernen und auch so mögen zu lernen, Austausch mit anderen Frauen und ja. |*Überweisung Psychotherapie >>*|

OD: Ähm, gut, dann, wie gross schätzen Sie das Interesse an einer Fortbildung jetzt allgemein in den, im Fachbereich Gynäkologie zur Körperdysmorphen Störung ein?

IP8: |**<< Grosses Interesse an Fortbildung**|Das ist sicher interessant oder, weil das ein grosser,|*Grosses Interesse an Fortbildung >>*| oder |**<< Vertrauensperson**|viele Frauen suchen den Zugang ja nicht über den Psychologen oder Psychiater, sondern über die Frauenärzte oder, weil's halt um den Gynbereich geht, jetzt wenn die dysmorphe Störung in Bezug aufs Genital |**<< Brüste**|oder auf die Brüste besteht|*Brüste >>*|,|*Vertrauensperson >>*| ist es sicher wichtig, so in Fortbildungsveranstaltungen, dass dieser Teil auch aufgegriffen wird. |**<< Untergebildet**|Häufig da auch eine Unterversorgung in der Weiterbildung besteht, oder.

|*Untergebildet >>*|OD: Genau, ähm und wie gross würden Sie das Interesse oder den Nutzen

an Hilfsmittel zur Diagnostik der Körperdysmorphen Störung einschätzen? Also so ein Screeninginstrument zum Beispiel.

IP8: |**<< Grosses Interesse an Diagnostik**|**<< Objektivieren**|Das ist sicher äh, das ist sicher sehr interessant, weil das erleichtert's auch, oder standardisiert's oder. |*Grosses Interesse an Diagnostik >>*|*Objektivieren >>*|**<< praktisch**|Da kann man dann wirklich, äh, da muss man dann nicht speziell ausgebildet sein, sondern man hat wie ein Hilfsmittel, wie den Mini Mental State bei den Demenzerkrankungen oder Demenzkranken, das kann jeder Unterassistent beauftragt werden, oder, das herauszufinden. Das ist sicher gut.

|*praktisch >>*|OD: Und ja, Sie haben jetzt schon erwähnt, dass Sie ähm, eben so ein, wie haben Sie das, die Gips, das Gips-Bild haben

IP8: [Ja. So Modelle, ja, mhm.

OD: Ja, das wäre ja wie ein Hilfsmittel zur Beratung von solchen Patientinnen

IP8: [Ja, absolut.

OD. Ähm, ja, Sie sind jetzt aber die erste, die so was erwähnt haben, wie schätzen Sie so allgemein jetzt in der Gynäkologie das Interesse an so einem Hilfsmittel ein, oder den Nutzen?

IP8: |**<< Grosses Interesse an Hilfsmittel**|Ich glaube schon gross, oder, weil wirklich in der Weiterbildung das nicht so, der Fokus nicht so drauflegt|*Grosses Interesse an Hilfsmittel >>*| oder, |**<< Untergebildet**|'ne Unter- , zu wenig Wissen da ist, oder, mhm und da das schon hilft oder, in der Beratung.|*Untergebildet >>*| Und manchmal Frausein, oder. Ich denke schon, dass das äh, ein Punkt ist. |**<< öffnen sich eher Frauen gegenüber**|Es gibt Frauen, die öffnen sich schon Männern gegenüber aber äh, das, die Nachempfindung ist vielleicht schon, ist schon Vorteil, mhm.

|*öffnen sich eher Frauen gegenüber >>*|OD: Gut, ja, das wär's eigentlich schon gewesen, ausser Sie möchten noch etwas hinzufügen?

IP8: Nein, ist gut, danke.

OD: Gut, danke vielmal.

Interview mit: IP9
Datum des Interviews: 06.06.2019
Dauer: 26 min
Anonymisierte Begriffe: Spitalname
IP-Nummer: 9

MS: Ja gut, ähm, dann beginne ich gleich mit der ersten Frage und zwar mit welchen psychischen Problemen kommen denn ähm, Patientinnen zu Ihnen in die Praxis?

IP9: Äh, gut, ist die Frage ob man's schon, |**<< Depressive Episode (F32) und rezidivierende depressive Störung (F33)**|also eben depressiver Formenkreis|*Depressive Episode (F32) und rezidivierende depressive Störung (F33) >>*|, ähm, |**<< Phobische Störung (F40) und andere Angststörungen (F41)**|Ängste|*Phobische Störung (F40) und andere Angststörungen (F41) >>*|, mh, |**<< Nichtorganische Schlafstörungen (F51)**|Schlaflosigkeit|*Nichtorganische Schlafstörungen (F51) >>*| zählt auch zu, nicht als Diagnose, aber genau. Selten wirklich psychiatrische Erkrankungen, sondern mehr so, mh, und ob man eine Depression diagnostizieren würde in so einem Fall, ist die zweite Frage, |**<< Depressive Episode (F32) und rezidivierende depressive Störung (F33)**|depressive Verstimmung|*Depressive Episode (F32) und rezidivierende depressive Störung (F33) >>*| von mir aus, |**<< Phobische Störung (F40) und andere Angststörungen (F41)**|Zukunftsängste, grundsätzlich Ängste, ja.

|*Phobische Störung (F40) und andere Angststörungen (F41) >>*|MS: Mhm, äh und welche Rolle spielt denn ähm, das äussere Erscheinungsbild bei Ihren Patientinnen? So die Zufriedenheit vor allem?

IP9: Das äussere Erscheinungsbild?

MS: Also mit dem Aussehen. Sprechen Sie da darüber oder ko-

IP9: [Ob ich die Leute anspreche darüber und weil ich das Gefühl habe es sie sind depressiv und schauen sich selber nicht oder was?

MS: Nein, einfach allgemein das äussere Erscheinungsbild. Also kommen Patientinnen zu Ihnen, die unzufrieden sind, wie sie aussehen? So meine ich das.

IP9: Aha, okay, jetzt. Ähm, ich denk ((lacht)) ist denn das eine Trickfrage? Ich glaube nicht, dass ich, fast gar nie einen Termin abgemacht habe, weil die Leute kommen wegen ihrem äusserem Erscheinungsbild. Dass das bei der Brustkontrolle, bei der Untersuchung im Gespräch ein Thema sein kann, ja. Dass sie finden, wenn ich sie wäge «Ach, ich bin zur schwer» oder dass wenn man die Brust untersucht sie sagen «Mh, die ist nicht schön, ich möchte sie operieren». Aber vielleicht, wenn dann Brust, das sind also paar ganz wenige. Und dann haben die sich schon informiert und sagen, sie wollen die Brust operieren, ob ich sie überweisen soll. Aber dass sie wirklich mit dem Auftrag kommen, selten.

MS: Und sie sprechen auch nicht an?

IP9: Mh, nein, also ich find nicht, weil ich nicht finde, dass man irgendetwas korrigieren sollte, wenn nicht irgendwo ein Leidesdruck da ist. Höchstens wenn es medizinisch sinnvoll wäre, ja, aber das ist in der Regel ja nicht.

MS: Mhm, okay. Ähm, ich habe jetzt eine Fallvignette für Sie.

IP9: ((lacht)). Fangfrage.

MS: ((lacht)). Ich würde Sie bitten, diese kurz durchzulesen.

[MS legt Fallvignette 1 hin. IP9 liest Fallvignette 1 durch.]

IP9: Mhm.

MS: Ähm, welche psychische Störung fällt Ihnen zu diesem Fallbeispiel ein?

IP9: Äh, weiss auch nicht, |**<< Körperwahrnehmungsstörung**|Körperwahrnehmungsstörung|*Körperwahrnehmungsstörung >>*| oder so. |**<< Depressive Episode (F32) und rezidivierende depressive Störung (F33)**|Wahrscheinlich ein bisschen depressiv,|*Depressive Episode (F32) und rezidivierende depressive Störung (F33) >>*| ähm, |**<< vermindertes Selbstwertgefühl und Selbstvertrauen**|Selbstwertmakel |*vermindertes Selbstwertgefühl und Selbstvertrauen >>*|oder Mangel, keine |**<< Essstörungen (F50)**|Ahnung ob sie noch eine Essstörung|*Essstörungen (F50) >>*| hat, hätt' ich jetzt so im Hintergrund. Ähm ja, genau.

MS: Worauf basieren Sie Ihre Annahmen? Welche Stellen zum Beispiel?

IP9: Ähm, ja also einerseits die gro-, also, das äussere Erscheinungsbild, das nicht gut, also die kleinen, hängenden Brüste, ist ja immer die Frage, hat sie abgenommen und sind die Brüste drum so. Die hässlichen Haare, die Haare ((lacht)) in der Regel wenn man sich nicht schaut, oder ähm, oder auch stark abnimmt, hätte ich jetzt gedacht, sind die Haare drum auch nicht so gut und dass das heutzutage einfach ein grosses Thema ist, dass man einen grossen Busen haben muss und dünn sein muss und drum sonst nicht an die Partys und so geht, also das gängige Erscheinungsbild in der Welt, also seit sie dreizehn ist, ist natürlich krass, vor allem dass das schon so früh angefangen hat. Ähm, einfach dass sie schon so lange leidet und die Gedanken ständig darum kreisen, da wäre so die depressive, ähm, und Selbstbildstörung, weil da steht, dass sie eigentlich eine attraktive Frau ist und man davon ausgehen würde, dass, also ich davon ausgehen würde, dass man objektiv sagen müsste, objektiv ist sie nicht hässlich, nur sie hat das Gefühl, sie sei hässlich. |**<< Depressive Episode (F32) und rezidivierende depressive Störung (F33)**|Vielleicht irgendwie sowas noch, also und der soziale Rückzug auch eher depressiv|*Depressive Episode (F32) und rezidivierende depressive Störung (F33) >>*| oder keine Ahnung, |**<< Phobische Störung (F40) und andere Angststörungen (F41)**|Ängste|*Phobische Störung (F40) und andere Angststörungen (F41) >>*|, irgendwie sowas.

MS: Mhm, ähm, wenn jetzt diese Patientin zu Ihnen kommen würde, was würden Sie sich noch für Informationen von ihr wünschen?

IP9: Mh, ja also ich würde gern ein bisschen erfahren wollen über wer sie ist, was sie macht, wie eben also wahrscheinlich so das soziale Umfeld bisschen explorieren, die Familiensituation explorieren, mal fragen, was sie schon alles gemacht hat, wo sie schon überall war, warum sie das findet. Ähm, einfach dem Ganzen mehr nachgehen, was ist eigentlich das Problem oder, und dann ist natürlich die weitere Frage ja, aha, aber wo, also jetzt rein in der Dynamik, was lauft zuhause, wieso fragt sie die Mutter immer, was ist dort irgend, hat sie Geschwister. Ähm, selbstverständlich würde ich wissen wollen, hat sie eine normale Periode, waren die Brüste schon immer so, haben sie gewechselt, waren die Haare schon immer so, ist das erst gekommen mit der Zeit. Die Nase, die phu, auf die würd' ich wahrscheinlich nicht gross eingehen, weil ich denk, dort ist nicht so, also das ja. Dann vielleicht ein bisschen herausfinden, was sie findet, was ihr Weltbild, was für sie wichtig ist, wie sie auf die Idee kommt, dass sie äh, mit wem sie sich vergleicht, ähm, ja. Genau, was ich gesagt habe, ob sie eine Periode hat, da wäre natürlich im Ganzen drin eine Untersuchung. Ich denk', wenn ich sie sehen würde, dann würde man ja auch das beantworten können eher, oder. Das ist jetzt ein bisschen schwierig mit der kurzen Fallvignette. Genau, ja, dann

geht's dann weiter, vielleicht würde man noch gewisse Sachen untersuchen, Labor oder sonst was aber das sind ja wie dann die weitergehenden, vielleicht auch ob sie einen Partner hat, wenn ja, nicht, oder ob sie eine Partnerin hat oder wie sie sich überhaupt orientiert, ähm, geschlechtlich, ja, irgendwie so. Wie's in der Schule läuft, gelaufen ist, wie es im Job ist. Ja. Oder, oder, oder…

MS: Ja ((lacht)), okay. Ähm, ich habe jetzt eine Auflösung. [MS legt die DSM-5-Kriterien hin.] Denn das Fallbeispiel ist ein Beispiel einer Körperdysmorphen Störung. Haben Sie davon schon mal gehört?

IP9: |**<< KDS unbekannt**|Ja, genau, das wäre für mich so Körperwahrnehmungsstörung|*KDS unbekannt >>*|.

MS: Genau, Sie haben es eigentlich schon recht gut erklärt.

IP9:|**<< Begründung**| In der Regel ähm, geben wir keine ähm, wirklich psychiatrischen, ich hüte mich, psychiatrische Diagnosen zu geben, weil ich nämlich nicht Psychiater bin.|*Begründung >>*|

MS: Aber Sie hätten an so etwas gedacht?

IP9: Jaja. Und |**<< Zwangsstörungen (F42)**|Zwangsstörung |*Zwangsstörungen (F42) >>*|oder da geht die |**<< Essstörungen (F50)**|Essstörung|*Essstörungen (F50) >>*| auch so ein bisschen in die -

MS: Genau, das wäre ein Ausschlusskriterium, also dass es bei dieser Störung eben nicht um eine Essstörung geht, sondern eben, wie Sie eigentlich schon gesagt haben, übermässige Beschäftigung, es ist ein Makel, der für Andere gering erkennbar ist oder gar nicht. Dann eben diese wiederholenden Verhaltensweisen wie ständig im Spiegel anschauen, Rückversicherung.

IP9: Genau, das ist so das zwanghafte.

MS: Genau. Und eben dann der hohe Leidensdruck und die Beeinträchtigung, man kann nicht mehr raus, ist sozial und beruflich eingeschränkt.

IP9: Also was in dem Kontext ja ein grosses Thema ist, ist das Rasieren im Intimbereich. Also das kommt ((lacht)) das, das diskutiere ich häufig. Weil einfach immer das Gefühl ist, man muss absolut blank sein, und das ja, mhm.

MS: Wenn wir jetzt nochmal diese Patientin anschauen und Sie jetzt das im Hinterkopf haben, ähm, haben Sie schon Erfahrungen gemacht mit solchen Patientinnen? Kennen Sie das oder kommt das eher nicht so häufig vor?

IP9: Ähm, nein, würd' sagen ich hab' schon ein paar. Also mit kommt gerade eine in den Sinn, aber die ist psychisch wirklich auch sonst noch, also da sind noch andere, ich weiss nicht, ob sie noch ein ADHS hat oder irgendwas, sie ist auf jeden Fall in psychiatrischer Betreuung. Ähm, da habe ich sie eben nach Jahren hingekriegt ((lacht)). Genau, und die ist eben kürzlich gekommen wegen den Brüsten.

MS: Wegen den Brüsten?

IP9: Ja, genau.

MS: Und die sehen eigentlich normal aus, aber sie findet

IP9: [Nein, sie hat effektiv kleine, hängende Brüste, sie hat auch stark abgenommen. Also ich versteh bei ihr, wieso das ist. Aber ja. Aber alles andere finde ich auch, sie ist eine wunderschöne Frau ((lacht)), ja.

MS: Ja, also die Frage wäre einfach, was sind Ihre Erfahrungen mit Patientinnen, die solche Symptome zeigen?

IP9: Hm, hartnäckig, langwierig, ähm, Hauptthema gute Bindung, gute Beziehung aufbauen, versuchen, anzubinden, langsam ranzugehen, immer daran bleiben, es sind ja mehrheitlich die Jungen und die ja eigentlich nicht zum Arzt gehen, nicht gehen wollen, nicht kommen und dass man da wie versucht, niederschwellig auch Angebote zu machen, dass, ich muss gestehen, ich arbeite manchmal mit Psychologen zusammen und ich lasse die Patientinnen dann aufbieten, weil man das Eisen wieder einmal kontrollieren sollte oder so, einfach, damit ich sie wieder sehe, damit man einfach eine Kontinuität hat und nach und nach und nach einfach abholen kann, wo das ist. Es braucht ja ganz lange, bis die wirklich sagen, was das Problem ist, wenn sie überhaupt selber schnallen, was das - oder verstehen, was das Problem ist. Ähm, ja und dann versuche ich sie psycho-, psychotherapeutisch anzubinden, wenn's irgendwie geht. Und ähm, auch jedes Mal wieder halt mit dem versuchen, dass sie nicht operieren, sie zurückhalten, manchmal halt auch mit «Sie sind zu jung, drum können wir es noch nicht» also mehr so. Und dass sie sehr viel Liebe und ein Zuhause brauchen. Also es ist effektiv, häufig habe ich das Gefühl, dass die einfach keinen Rückhalt haben sozial, irgendwo zuhause nicht, niemand versteht sie und sie eigentlich durch das gern mit der Zeit und das sieht man ja auch, dass sie wie lieber kommen, weil sie sich irgendwo auch wohl fühlen, zuhause fühlen. Also das ist für mich der Hauptding, dass ich wie das Gefühl habe, dass es denen wie den Boden geben, das Gefühl geben, «Okay, wenn ich irgendetwas hab, dann komm ich hier hin» und wenn ich irgendetwas nicht sicher bin, dann frag ich, damit sie nicht genau Dummheiten machen wie die Nase operieren obwohl sie schön wär. Das ist so mein Ziel bei solchen ((lacht)) Patienten und äh, es sind doch viele, die kommen wieder und haben dann die Sache nicht, und es geht ja nicht wie auch ein Rübertragen in einer schwierigen Zeit. Also ich denke, wenn sie dann mal fünfundzwanzig sind oder älter, häufig wird es besser. Ja nicht immer aber ähm, genau, da haben sie wie andere Sachen, all die Lehrerprobleme und solche Sachen sind häufig in der Phase. Ja, ein bisschen so.

MS: Das haben Sie jetzt eigentlich schon erwähnt, ich erwähne es trotzdem nochmal, die nächste Frage wäre nämlich, auf welche Herausforderungen treffen Sie im Kontakt mit diesen Patientinnen. Da haben Sie gesagt fehlende Einsicht zum Teil, es braucht sehr viel Zeit, gute Beziehung –

IP9: Ja, Vertrauen ist ein sehr grosses Thema, sind wie scheue Katzen, oder. ( ) Sie ein bisschen was und dann kommt sie und mit der Zeit merken sie: «Aha, da tut mir niemand weh, wenn ich hier hin komme». Also sehr viel Geduld, sehr viel Zeit und sehr viel, genau, Beziehungsarbeit. Und ja, fehlende Einsicht, Ängste auch wirklich, Ängste vor Sachen, Ängste vor medizinischen, häufig geht ja, dass sie operieren wollen einher mit ganz grossen Ängsten und Zweifel wo sie sagen: «Ja, nein» das würden sie nicht machen aber operieren das würden sie. Also, irgendwo auch Doktor Google beantworten, auch immer ein Thema. Also die kommen dann mit weiss ich was aus dem Internet und ich frag dann: «Was haben Sie gelesen?» Also schauen wir es zusammen an. Ja, weil so ist es heute, oder.

MS: Äh und meine letzte Frage haben Sie auch schon angesprochen und zwar ist die, wie wäre Ihr professionelles Vorgehen, wenn Sie jetzt eine Patientin haben, die diese KDS-Symptomatik hat? Da haben Sie schon erwähnt, Sie würden ähm, sie weiterschicken wahrscheinlich?

IP9: Ja, genau, also probieren, oder. Manchmal gehen sie ja nicht einfach, sie sind ja nicht einsichtig, dass es einen Psychologen braucht oder einen Psychiater brauchen und genau

drum wär es häufiger aufbieten, Beziehung aufbauen, Sachen anschauen, erklären, ganz viel Aufklärung und wirklich, dass die kommen und Fragen stellen und, und, dass die merken, es geht um, dann langsam versuchen, genau, die psychiatrische oder psychologische Anbindung äh, aufzugleisen. Irgendwie so.

MS: Ja.

IP9: Dann aber trotzdem mitbetreuen, also ich mach gerne so eine Doppelbetreuung, damit das Medizinische auch abgefangen ist, dass sie nicht eben an die Schönheitskliniken gehen, die ja einfach operieren, wenn man genug Geld hat. Oder häufig das machen so.

MS: Das heisst, Sie haben häufig Leute, die operieren möchten?

IP9: Ja, nicht häufig, aber es kommt vor. Oder die kommen und sagen: «Ja, ich hab' da ein Termin, ich geh nach Österreich, geh nach Deutschland, ich geh irgendwo in den Balkan und mach diese Operation, ich brauche einen vor-, präoperativen, sind aber ältere Frauen, bei mir auf alle Fälle, sind mehr die vierzig plus minus. Die Jungen schon auch aber dort hab' ich mehr, bei den Älteren hab' ich weniger zu sagen, bei den Jungen versuch ich wirklich, Einfluss zu nehmen.

MS: Gut, danke, dann wäre das mein Teil schon gewesen.

OD: Ähm, dann mache ich grad weiter. Ähm, was sind Ihre Erfahrungen mit Patientinnen, die Fragen zur Ästhetik oder zur Normalität ihres eigenen Genitalbereichs stellen?

IP9: |**<< Zunehmend**|Ähm, wesentlich mehr als früher.|*Zunehmend >>*| Also ich denk, als ich angefangen habe, war das kein Thema ((lacht)) und jetzt ist so, ah ja genau, |**<< Erwähnen OP**|man kann diese Operation und das machen.|*Erwähnen OP >>*| |**<< Kultur**|In der Übergangsphase waren es vor allem die Russinnen, die alle kommen und gesagt haben: «Ja, man muss doch da jung aussehen unten und ich mach diese Operation».|*Kultur >>*| Äh, jetzt vermehrt,|**<< Jüngere**| also ich hatte auch schon ganz junge Frauen, |*Jüngere >>*|die kommen und quasi sagen, eben, ist das normal, kann das sein, dass man es wie anschaut. |**<< Labien**|Ist meistens ein Thema der kleinen Schamlippen und ähm, mittlerweile |**<< Vielfalt**|sage ich ihnen, sie sollen|*Labien >>*| «Die göttliche Ordnung» schauen ((lacht)), wenn sie den Film schauen, gibt's so eine Szene wo sie die ganzen entscheiden, wie ihr Genitale aussehen soll, ob's der Schmetterling oder Tiger oder sonst was ist, oder, einfach zum Sagen, es gibt eine Diversität,|*Vielfalt >>*| |**<< Normalität betonen**|Normalität ist ein wahnsinnig breiter Begriff |*Normalität betonen >>*|und |**<< Aufklärung OP**|operieren finde ich immer ähm, muss man sich ja fragen, was macht man kaputt, man nimmt ja die Nerven am äusseren Ende weg |*Aufklärung OP >>*|und |**<< Intimrasur**|das nur weil es und, da komme ich wieder aufs Rasieren, seit man rasiert, sieht man das mehr, wenn man nicht rasiert, dann sieht man das eben gar nicht. Und das machts natürlich schwieriger für die Jungen.|*Intimrasur >>*| |**<< Bemerkungen**|Und dass alle Männer mitreden in dem Bereich kommt auch dazu. |*Bemerkungen >>*|Früher hatten sie nichts zu sagen, jetzt ist so, ah, dieser Schnitt und diese Frisur, das ja, das nein, also, genau. |**<< Viele Fragen** |**<< Zunehmend**|Also wesentlich mehr ein Thema als es früher war|*Viele Fragen >>*| und vielleicht fünf Jahre sicher, ob's zehn Jahre sind, da bin ich nicht ganz sicher,|*Zunehmend >>*| |**<< Intimrasur**|also das mit dem Rasieren hat ja schon länger angefangen, aber dass es wirklich so, ja, so öffentlich, ist, das hätte ich jetzt gesagt, wahrscheinlich die letzten fünf Jahre.

|*Intimrasur >>*|OD: Und, wenn Sie sagen, mehr als früher, wie häufig ungefähr kommt das vor?

IP9: Hm, also dass jemand kommt, wirklich weil sie wissen will, ob alles in Ordnung ist oder dass man's bespricht während einer Kontrolle?

OD: Beides.

IP9: |**<< Jährlich**|Also, dass jemand kommt, all Halbjahr.|*Jährlich >>*| |**<< Wöchentlich**|Und dass man's bespricht, vielleicht einmal, zweimal in der Woche, im Schnitt. |*Wöchentlich >>*|Es kann Wochen geben, da sag ich das Gleiche fünf Mal am Tag und andere Wochen, wo's weniger ein Thema ist. Es kommt auch ein bisschen drauf an bei uns, wie viel Gynäkologie dann drin ist oder, das ist so, genau. Aber an einem Gynitag kommt es sicher häufiger vor ((lacht)).

OD: Und was ist dann Ihr professionelles Vorgehen zu solchen Fragen?

IP9: |**<< Aufklärung**|Ähm, Aufklärung, |*Aufklärung >>*|also einfach, dass ich der Meinung bin, dass eben, |**<< Normalität betonen**|dass es eigentlich keine Vorschriften gibt, dass die Normalität eine grosse Bandbriete hat |**<< Abratung von OP**|und dass wenn's nicht stört, nicht krankhaft ist und drum keinen Grund gibt, es zu operieren.|*Abratung von OP >>*| |*Normalität betonen >>*|**<< Gespräch**|Eben sie ermuntern, doch auch mit den Kolleginnen darüber zu reden,|*Gespräch >>*| |**<< Expertise nutzen**|ich bring dann irgendwie Prozentzahlen, keine Ahnung ob sie immer stimmen, aber dass, also einfach, ganz, also so und so viel Prozent Frauen gibt, die haben das genau gleich, oder ich habe Patienten einfach aus meinen eigenen|*Expertise nutzen >>*| ( ), so und so viele Patienten haben da so und die anderen haben das so und ermuntere die Frauen auch, selbstsicherer zu sein. Wenn sie Haare haben wollen, sollen sie diese Haare lassen. |**<< Beziehung**|Und dass und vielfach ist ja so das Partnersuchthema oder, «Ich werde ja niemals einen finden, wenn», |**<< Sexualität**|«der wird dann nicht mit mir schlafen, wenn ich nicht rasiert bin»|*Beziehung >>*|*Sexualität >>*|, ja hallo, oder. |**<< Gespräch**|**<< Visuell**|Also einfach so, auch dort, genau, solche Themen ansprechen |*Gespräch >>*|und das anschauen, mit ihnen mit dem Spiegel das Genitale anschauen, erklären, was was ist eben, |**<< Aufklärung**|**<< Normalität betonen**|das meine ich mit Aufklärung und wirklich auch ermuntern, |**<< Patientin muss selbst an sich arbeiten**|sich selber gern zu haben und das als Normalität anzunehmen|*Aufklärung >>*|*Normalität betonen >>*|.|*Visuell >>*|*Patientin muss selbst an sich arbeiten >>*| Genau.

OD: Ähm und fragen Sie manchmal auch bei Ihren Patientinnen nach, wie sie zur Ästhetik ihres Genitalbereichs stehen?

IP9: |**<< Fragen medizinisch**|Selten. Also eben, ich denk, wenn irgendwas nicht gut wär', was ich das Gefühl habe, dann würde ich fragen. Aber ich würde jetzt nie jemand, der grosse Schamlippen hat, fragen «Stört Sie das, oder was finden Sie?», nein.|*Fragen medizinisch >>*|

OD: Ja. Ähm, das haben Sie jetzt schon ein bisschen beantwortet aber ich frage trotzdem nochmal. Ähm, was sind Ihre Erfahrungen mit Patientinnen, die jetzt eben direkt nach Schönheitsoperationen am weiblichen Genitalbereich fragen?

IP9: Die fragen?

OD: Oder wünschen.

IP9: Ähm, |**<< Direkt OP**|also die, die schon wissen, dass sie das machen wollen, die haben sich meistens, wissen die schon, wohin sie gehen und haben das alles schon,|*Direkt OP >>*| |**<< Kultur**|eben insbesondere die Russinnen, die haben, die wissen schon, wo sie hingehen.|*Kultur >>*| |**<< Jüngere**|Dann hab' ich solche, die wirklich ähm, sagen wir junge Frauen, |**<< Labien**|lange innere, grosse innere Schamlippen oder das stört mega,|*Jüngere >>*| oder irgendwie so Zipfel, also wenn's genau, |**<< Schwangerschaft/Geburt**|wenn's so Sachen hat, die rausstehen, auch nach einer Geburt oder so.|*Labien >>*|*Schwangerschaft/Geburt >>*| |**<< Gespräch**|In der Regel besprich ich das mit ihnen, |**<<**

**Aufklärung OP**|sag ihnen meine Bedenken|*Gespräch >>*|,|*Aufklärung OP >>*| |**<< Ernstnehmen**|hol mal ab, was ist, oder warum sie das wollen, ob es stört, warum, was und |**<< Überweisung an Chirurgie**|dann weis ich die weiter.|*Ernstnehmen >>*| Ähm, da entweder ans SPITALNAME in die Sprechstunde oder an ein, zwei niedergelassene Gynäkologinnen, die auch operativ tätig sind.|*Überweisung an Chirurgie >>*| Die eine ist eben nicht operativ tätig, |**<< Zusammenarbeit**|dann haben sie wie eine second opinion und nochmals jemand wo ich weiss, dass sie auch nicht einfach operiert und aber sehr eine gute Person ist, also die kann die Leute auch abholen.|*Zusammenarbeit >>*| Im SPITALNAME bin ich der Meinung, sind sie auch sehr zurückhaltend und dort immer auch zur Sprechstunde zuerst, damit nochmals das besprochen wird. |**<< Zusammenarbeit**|Ich sag ihnen dann: «Operation, das kann ich sowieso nicht entscheiden, das müssen Sie nochmals besprechen». Da kann ich mich herausnehmen|*Zusammenarbeit >>*| und so, ähnlich wie bei den Abtreibungen, eine zweite Person ranlassen, zum nochmals die Situation anschauen. Genau. |**<< Überweisung an Chirurgie**|**<< Sinnvoll**|Wenn's klar etwas ist, das man einfach wegnehmen muss, dann meld' ich sie direkt zur Operation an.|*Überweisung an Chirurgie >>*|*Sinnvoll >>*| |**<< Wenig Eingehen**|Aber immer durch dass das wir weiterweisen, genau, müssen wir da nicht so stark Stellung beziehen.|*Wenig Eingehen >>*|

OD: Gut, ähm, ich hab' jetzt auch noch ein Fallbeispiel dabei. Ich bitte Sie, das mal durchzulesen.

[OD legt Fallvignette 2 hin. IP9 liest Fallvignette 2 durch.]

IP9: Ja, ((lacht)). Genau, oder, das ist genau das, die inneren Schamlippen.

OD: Ähm, also das wär' jetzt ein Beispiel einer Körperdysmorphen Störung des weiblichen Genitalbereichs. Also die Körperdysmorphe Störung kann jedes Körperteil betreffen und hier wäre es eben der Genitalbereich. Ähm, was sind Ihre Erfahrungen mit Patientinnen, die solche Symptome zeigen?

IP9: |**<< Viele KDS**|Viele, jetzt genau in dem Bereich,|*Viele KDS >>*| find ich, sind im Gegensatz zum anderen Beispiel, oder beim anderen ist's wie klarer, |**<< Uninformiert**|das ist ja wie nicht klar, dass das, weil sie nicht wissen, was normal ist.|*Uninformiert >>*| Und genau drum find ich bei sowas, |**<< Aufklärung**|hab' ich mit Aufklärung und Erklärung und Beruhigung viel mehr Erfolg als beim anderen Beispiel.|*Aufklärung >>*| |**<< Jüngere**|Genau, also eben genau die ganz Jungen|*Jüngere >>*| oder |**<< Beziehung**|eben auch die, es geht ja genau darum, wenn der Freund sagt, er sagt immer « ja, es ist normal»,|*Beziehung >>*| auch das brauchen oder. |**<< Vielfalt**|Die Männer, die fragen immer nach der Penisgrösse, ja toll, oder, wieso sollten wir nicht unterschiedliche Schamlippen haben wie die unterschiedliche Penisgrössen haben? Die gehen gar nicht davon aus, dass das anders, sind immer die Frauen.|*Vielfalt >>*| |**<< Gespräch**|Und dann mal auch, eben ermuntern mit den Freundinnen reden, ermuntern,|*Gespräch >>*| so einen Film zu schauen, ermuntern, das mal im Internet anzuschauen, das machen sie ja in der Regel ja sowieso. |**<< Selbst behandeln**|Genau, also da hab' ich die besseren, also aus meiner Erfahrung mindestens, sind das solche, die man viel besser abholen kann, die viel weniger dem nachgehen,|*Selbst behandeln >>*| |**<< Subklinische Unzufriedenheit**|auch eben für mich, ich hätt' jetzt das noch gar nicht als Körperdysmorphe Störung ((lacht)) diagnostiziert, |**<< Uninformiert**|sondern einfach als ähm, Mangelinformation |*Uninformiert >>*|und ähm, ein Problem, |**<< Unsicher**|vielleicht ein Selbstwertproblem, das ja in dem Alter häufig noch ein Thema ist und Unsicherheit, so, genau.

|*Subklinische Unzufriedenheit >>*|*Unsicher >>*|OD: Ähm, also, was wäre jetzt Ihr Vorgehen dazu, einfach aufklären?

IP9: [|**<< Visuell**|**<< Miteinbeziehen**|Genau, ich würd' mit ihr das anschauen, |**<< Untersuchung**|also ich würd's mir selbstverständlich zuerst anschauen,|*Untersuchung >>*|

dann würde ich es mit ihr anschauen, im Spiegel,|*Visuell >>*|*Miteinbeziehen >>*| |**<< Gespräch**|das besprechen,|*Gespräch >>*| erklären, |**<< Anamnese**|was sind denn genau so die Themen|*Anamnese >>*|, äh, |**<< Intimrasur**|Rasieren, ja, nein, dann sieht man's nicht, das Thema, man ist nicht weniger attraktiv, wenn man das hat, das Thema,|*Intimrasur >>*| wichtig ist, |**<< Beziehung**|dass der Freund, oder, genau, darum sag ich, das Thema ist immer «ah nein, ich find nie einen Freund, wenn ich solche Schamlippen hab», die hat sie jetzt aber.|*Beziehung >>*| Auch ermuntern eben der andere zu fragen, |**<< Aufklärung**|erklären, wieso das überhaupt ein Thema ist heutzutage, bisschen so, genau.|*Aufklärung >>*| |**<< Medizinische Beschwerden**|Und sie auch fragen, wo und was sie stört, oder ob es sie stört, ob sie irgendwie immer wund sind, ob sie Probleme hat damit, genau.|*Medizinische Beschwerden >>*| |**<< Aufklärung**|Indem Sinn aufzeigen, dass es eine normale Funktionalität hat, in der Annahme, dass das so ist. Mhm, irgendwie so, ja|*Aufklärung >>*|.

OD: Gut. Ähm, wie gross würden Sie ein Interesse an einer Fortbildung zur Körperdysmorphen Störung jetzt so im allgemeinen, gynäkologischen Fachbereich einschätzen?

IP9: Mir? Oder bei den Leuten?

OD: Ja, so allgemein.

IP9: Also, dass Patienten, dass man quasi sagen kann «Ah, dort gibt es eine Infoveranstaltung, gehen Sie dorthin -

OD: [Nein, nein, für jetzt

IP9: [Ärzte

OD: Ja, genau.

IP9: Also wie gross schätze ich das, den Wunsch oder das Interesse ein bei meinen Kollegen, dass sie an so einer Veranstaltung teilnehmen?

OD: [Ja, also Sie selber und Ihre Kollegen.

IP9: Ähm, |**<< Mittel Interesse an Fortbildung**|ich find's ein spannender Bereich, ich denk, es ist immer wieder, also es ist ja, gut es ist eben nicht die Körperdysmorphe Störung|*Mittel Interesse an Fortbildung >>*|, sondern sowas, was ist die Genitalverstümmelung ein Thema eigentlich mehr. Ähm, fänd ich super, wenn's mal kommen würde in der, insbesondere wenn man die Gynäkologen ein bisschen äh, die operativ tätig sind ((lacht)), informieren würde und wahrscheinlich am besten ist an einer Grossveranstaltung wo man wegen anderem geht und dann gibt's halt eine Vorlesung über das, weil eine Einzelvorlesung, da kommen die wahrscheinlich nicht. Ähm, würde ich u-, also wenn's passt vom Zeitlichen her, fände ich das sehr ein spannendes Thema.

OD: Mhm. Und wie gross würden Sie das Interesse oder den Nutzen an Hilfsmittel zur Diagnostik der Körperdysmorphen Störung einschätzen?

IP9: Im Sinne von Checklisten?

OD: [Also so, genau, also Screeninginstrumente, also es gibt welche auf Englisch, die man jetzt so, wahrscheinlich

IP9: [Die man den Leuten gibt oder die ich habe?

OD: Äh, beides.

IP9: So wie das Depressionsschema, da wo man -?

OD: [Genau, ja.

IP9: Ähm, |**<< Wenig Interesse an Diagnostik**|also ehrlich gesagt, würd' ich's wahrscheinlich durchlesen, in ein Fach tun und denken ich hab' das und nachher nicht mehr daran denken, |*Wenig Interesse an Diagnostik >>*|sondern selber, also in der Regel brauch ich die Sachen so nicht, |**<< Bewusstsein**|aber zum ab und zu mal Nachschauen, ah genau, hab ich an alles gedacht,|*Bewusstsein >>*| ja. Ähm, ja, ich bin nicht so ein Checklisten-Mensch. |**<< Unpraktisch**|Aber grundsätzlich eine gute Sache aber man hat so viele Papiere heutzutage, also es kommt jeder mit irgendeiner Checkliste, dann und äh, ja.|*Unpraktisch >>*| Man fragt's oder man fragt's nicht. Entweder man denkt dran oder nicht, aber genau, zum's mal haben und da wäre wahrscheinlich die Weiterbildung eben sinnvoller.

OD: Genau. Ähm und noch die letzte Frage. Wie gross würden Sie das Interesse für Hilfsmittel für die Beratung ähm, für Patientinnen jetzt mit dieser Symptomatik, also mit Körperdysmorpher Störung des Genitalbereichs einschätzen?

IP9: Modell?

OD: Ja genau, oder wie Sie gesagt haben Bilder oder so.

IP9: |**<< Grosses Interesse an Hilfsmittel**|Ja, das fänd' ich super, das habe ich nämlich nicht.|*Grosses Interesse an Hilfsmittel >>*| Das einzige, was ich hab', ist das da da und da sind die Schamlippen nicht dargestellt. [Zeigt 3D-Modell des inneren Genitals.] Es ist zwar sonst supi, man kann's aufmachen, man kann's zumachen, man kann das Spekulum reinhalten aber ja, das fänd' ich also super. Genau das Plakat, das sie in der «Göttlichen Ordnung» haben, sowas wer cool. Also ja, da grosses Interesse.

OD: Gut, dann wären wir eigentlich schon am Ende, ausser Sie möchten noch irgendetwas hinzufügen?

IP9: Mh. Nein, also nicht zur Frage, zum Interview.

OD: Gut, danke.

Interview mit: IP10
Datum des Interviews: 06.06.2019
Dauer: 17 min
Anonymisierte Begriffe: ausländischer Stadtname, Spitalname, Ländername
IP-Nummer: 10

MS: Ja dann beginne ich gleich mit der ersten Frage und zwar ähm, mit welchen psychischen Problemen kommen denn Patientinnen zu Ihnen in die Praxis?

IP10: Ich habe viele |**<< Nichtorganische Schlafstörungen (F51)**|Schlafstörungen|*Nichtorganische Schlafstörungen (F51) >>*| und |**<< Depressive Episode (F32) und rezidivierende depressive Störung (F33)**|Depressionen|*Depressive Episode (F32) und rezidivierende depressive Störung (F33) >>*|. Was hab' ich noch? Ich habe auch |**<< Essstörungen (F50)**|Essstörungen|*Essstörungen (F50) >>*|, aber das häufigste ist eigentlich Gefühlstiefs, sagen wir's so. |**<< Depressive Episode (F32) und rezidivierende depressive Störung (F33)**|Depressive Verstimmungen.

|*Depressive Episode (F32) und rezidivierende depressive Störung (F33) >>*|MS: Mhm, ähm, welche Rolle spielt denn die Zufriedenheit mit dem äusseren Erscheinungsbild bei Ihren Patientinnen?

IP10: Das ist ganz verschieden, ich muss grad – (1) eigentlich nicht ein so grosses. Jetzt verglichen mit anderen Ländern, wo ich gearbeitet habe. Also es kommt immer wieder vor, dass irgendwas sie stört im Äusseren aber jetzt zum Beispiel Essstörungen habe ich ganz selten.

MS: Ja. Und was stört die Leute am Körper oder -?

IP10: [|**<< Labien erwähnt**|Also jetzt habe ich gerade eine die stört die Grösse der Schamlippen|*Labien erwähnt >>*|, findet sie einfach zu gross. Oder dann natürlich das Gewicht, das Übergewicht. Aber auch das eher selten muss ich sagen, eher ziemlich selten. Ich habe sehr viele selbstbewusste, junge Frauen.

MS: Okay.

IP10: Mhm, erstaunlich ((lacht)).

MS: ((lacht)) Gut.

OD: ((lacht)) [Das ist schön.

MS: Ja. Okay. Ähm, ich habe eine Fallvignette dabei und würde Sie bitten, die kurz durchzulesen.

[MS legt Fallvignette 1 hin. IP10 liest Fallvignette 1 durch.]

IP10: Ja. (3) Okay.

MS: Mhm. Ähm, welche psychische Störung fällt Ihnen zu diesem Fallbeispiel ein?

IP10: Ich denke das ist eine (.) falsche -, eine |**<< Körperwahrnehmungsstörung**|Wahrnehmungsstörung|*Körperwahrnehmungsstörung >>*|, oder.

MS: Mhm.

IP10: Obwohl ich jetzt nicht weiss, wie sie wirklich aussieht. Sie ist einfach sehr kritisch ihrem Äusseren gegenüber, |**<< vermindertes Selbstwertgefühl und Selbstvertrauen**|ein tiefes Selbstbewusstsein.|*vermindertes Selbstwertgefühl und Selbstvertrauen >>*| |**<< Begründung**|Aber welche psychische Störung, kann ich jetzt nicht sagen.|*Begründung >>*| Sie scheint ja nicht, dass sie auch versucht, etwas zu ändern aktiv, |**<< vermindertes Selbstwertgefühl und Selbstvertrauen**|sie ist einfach unsicher.

|*vermindertes Selbstwertgefühl und Selbstvertrauen >>*|MS: Mhm. Ähm, worauf basieren Sie Ihre Annahmen, also zum Beispiel welche Stellen sind Ihnen aufgefallen?

IP10: Dass sie dann vermeidet, deswegen soziale Aktivitäten wie Partys, Dates, sie geht nicht mehr regelmässig an die Uni, also sie scheut jetzt den soziales- ihr soziales Umfeld. Viele Leute werden geplagt durch Unsicherheiten während des, genau, also während des Teenageralters, wenn man älter wird. Aber wenn man denn Vermeidung hat, dann ist es find ich schon, ziemlich schlimm.

MS: Mhm. Wenn jetzt so eine Patientin zu Ihnen kommen würde, was würden Sie sich noch für Informationen bei ihr einholen?

IP10: Ich würde sicher mal das Gewicht machen, ich würde anschauen wie der BMI ist. Ich würde fragen, wie in der Familie, wie sie eingebett- , also was sie für ein Zuhause hat. Sicher auch mal Drogen, Alkohol, einfach das ganze Bild. Schule, Freunde, Freundeskreis.

MS: Mhm. [MS legt die DSM-5-Kriterien hin.] Ähm, ich habe jetzt noch eine kleine Auflösung. Ähm, das Beispiel wäre ähm, ein Beispiel einer Körperdysmorphen Störung.

IP10: Okay.

MS: Haben Sie davon schon mal gehört?

IP10: |**<< KDS unbekannt**|Von dem Ausdruck nicht, nein.|*KDS unbekannt >>*|

MS: Ja. Mhm. Also es geht eben um eine übermässige Beschäftigung mit einem wahrgenommenen Makel, das heisst, er ist für Andere eben nicht erkennbar oder nur geringfügig. Ähm, das geht mit vielen wiederholenden Verhaltensweisen einher, im Spiegel überprüfen, bei Anderen nachfragen: «Sieht das normal aus?». Ähm, und dann eben, das haben Sie gesagt, dass hat ähm, die Person leidet sehr, sie sind beeinträchtigt, sozial, beruflich und so weiter. Und eben als Abgrenzungskriterium, es ist keine Essstörung. Also es geht nicht um das Körperfett oder die Körperfettverteilung.

IP10: Okay, mhm, mhm.

MS: Genau. Wenn wir jetzt die Patientin nochmals anschauen und das im Hinterkopf haben, haben Sie schon einmal solche Patientinnen gehabt, haben sie da Erfahrungen schon damit gemacht?

IP10: Nein, ich kenne eher Leute im Umfeld die das haben als Patienten ((lacht)).

MS: Ahja? ((lacht)).

IP10: Nein, Sie müssen natürlich auch wissen, wir haben nicht so viele Gyn, also so viele Gyn habe ich jetzt auch nicht, einfach Frauen allgemein. Aber ich habe auch sonst viele junge Frauen. Dass sie wirklich dann das Umfeld meiden, nein, so schlimm habe ich das noch nie gesehen in der Praxis.

MS: Mhm. Ähm, dann die nächste Frage ist, ich weiss nicht, vielleicht können Sie das nicht so gut beantworten, aber ich frag trotzdem. Ähm, auf welche Herausforderungen treffen Sie denn mit Patientinnen die jetzt zum Beispiel so, äh, eine Symptomatik haben?

IP10: Also, wenn sie jetzt mit dem

MS: [Oder

IP10: kommen?

MS: [Genau oder, sagen wir hypothetisch, was denken Sie, was wären die Herausforderungen bei solchen Patientinnen?

IP10: [Das geht natürlich sehr ins Psychologische rein. Und das ist für mich eine grosse Herausforderung, da ich nicht psychologisch -, da arbeite ich sehr gerne mit Psychologen zusammen. Und ähm, ich habe auch eine, da kann ich eigentlich jederzeit zuweisen und das ist dann für mich häufig eine Zusammenarbeit mit einer Psychologin zusammen. Eben wenn es dann schon zum Vermeidungsverhalten kommt dann ist es für mich so «red flags». Das ist mehr als nur eine normale Unsicherheit, da hat jeder von uns irgendwas, das stört.

MS: Mhm. Wenn jetzt so eine Patientin zu Ihnen kommen würde, genau mit diesen Problemen, wir würden Sie denn da konkret vorgehen? Im Erstgespräch oder -

IP10: [Ich würde wahrscheinlich mal fragen, was wünschen Sie sich denn, was stört Sie genau. Ich mein, wenn die dann zu mir kommt und ihre Nase operieren möchte oder weiss was, was weiss ich da noch alles machen, dann würd' ich natürlich auch mal darüber sprechen, dass es sich dann wahrscheinlich nicht ändert mit der Nasen-Operation. Und dann würde ich sie wahrscheinlich schon als zweites zum Psychologen schicken.

MS: Mhm. Geht das einfach?

IP10: Ja jetzt, mit einer geht es sehr einfach, weil sie eine MPA hat. Muss ich sagen, macht's viel einfacher. Ich kann dort anrufen und einen Termin vereinbaren sofort und jederzeit. Die meisten Psychologen sind nicht erreichbar. Und dann ist es für mich, ich gebe sehr gerne den Patienten, wenn sie vor mir sitzen, gerade einen Termin mit.

MS: Ja, okay. Und wie ist es für die Patienten selber? Nehmen die das sehr gerne a-

IP10: [Also die müssen natürlich wollen. Also ich frage: «Habt Ihr, möchtet Ihr mal mit einem Psychologen sprechen? Ich finde das jetzt ein Problem, das würde ich gerne mit einem Psychologen zusammen mit Ihnen anschauen». Und ich habe selten Leute die sagen: «Nein». Also, meine oft, häufigste Zusammenarbeit ist mit Psychologen.

MS: Ja. Und Sie begleiten dann einfach weiter?

IP10: Ja. Und die Psychologen und ich schreiben uns dann E-Mails und sie sagen uns dann was sie noch für Vorschläge brauchen, also jetzt. Psychologen sind ja nicht spezialisiert auf Medikamente aber trotzdem kennen sie sich sehr gut aus. Dann gibt sie mir Vorschläge, was sie noch machen würde. Oder sie macht Verhaltenstherapie oder was jetzt für sie in diesem Fall anstehen würde. Ich denke aber, da braucht es wahrscheinlich schon (.) tieferes oder?

MS: Mhm. Gut, ähm, das wär's schon gewesen mit meinem Teil.

IP10: Gut.

MS: Danke.

OD: Ähm, dann mache ich grad weiter. Ähm, was sind Ihre Erfahrungen mit Patientinnen, die Fragen zur Ästhetik oder zur Normalität ihres eigenen Genitalbereiches stellen?

IP10:|**<< spezifisches Beispiel**| Das hatte ich gerade heute, ist noch lustig, dass ich das gerade hatte. |*spezifisches Beispiel >>*|**<< Gespräch**|Ähm (.), ich habe jetzt bei -, mit ihr kurz diskutiert,|*Gespräch >>*| |**<< Gelder**|die hat überhaupt kein Geld. Habe ich gesagt: «Schauen Sie, ich mein so ein Eingriff, das kostet einfach Geld, das bezahlt die Krankenkasse nicht». |*Gelder >>*|**<< Erwähnen OP**|**<< Kultur**|Ich weiss eigentlich, ich habe in AUSLÄNDISCHER STADTNAME gearbeitet und da hat jede zweite wollte ihre Schamlippen verkleinern, die kleinen Schamlippen.

|*Erwähnen OP >>*|*Kultur >>*|OD: [Oh, okay –

IP10: |**<< Labien**|Da hat es wie so gang und gäbe, ein Riesenthema, die zu grossen kleinen Schamlippen.|*Labien >>*| |**<< Unrealistisches Ideal**|Was die Grösse sein muss, |**<< Medien**|da hat irgend nach dem Vorbild Porno,|*Medien >>*| mädchenhaft oder und dann wird man eine erwachsene Frau und das sieht plötzlich ganz anders aus.|*Unrealistisches Ideal >>*| Ähm, was ist die konkrete Frage nochmals? ((lacht)).

OD: ((lacht)). Einfach was Ihre -

IP10: [Wie ich damit umgehe?

OD: Nein, was Ihre Erfahrungen sind.

IP10: Mhm. (3) |**<< Seltene Fragen** |**<< Meisten Frauen zufrieden**|Muss sagen, echt wenig Frauen kommen mit Problemen.|*Seltene Fragen >>*|**<< Intimrasur**|*Meisten Frauen zufrieden >>*| Es geht auch um die Rasur. Ich meine |**<< Kultur**|dort hab' ich nur rasierte, vollrasierte Frauen gesehen.|*Intimrasur >>*| In der Schweiz sieht man alles, zwischen gar nichts bis alles weg.|*Kultur >>*| Also, ich glaube man ist da auf eine Art weniger getrimmt durch so ein Schönheits-, ich weiss nicht ob ihr das anders seht, |**<< Jüngere**|aber ich finde die jungen Frauen, die ich hier in der Stadt Zürich sehe, haben da weniger ein Vorbild, «so muss es sein und anders bin ich hässlich». Das hab' ich, seh' ich wenige.|*Jüngere >>*| |**<< Medizinische Beschwerden**|**<< spezifisches Beispiel**|Also bei ihr war das jetzt heute konkret auch Schmerzen. In den Jeans. Es tut weh, weil das zu gross ist, oder. Aber nicht: «Ich finde mich hässlich, wenn ich mich nackt im Spiegel sehe», das nicht.

|*Medizinische Beschwerden >>*|*spezifisches Beispiel >>*|OD: Mhm, okay. Ähm, die nächste Frage ist jetzt, wie gehen Sie damit um?

IP10: |**<< Untersuchung**|**<< spezifisches Beispiel**|Also mit ihr habe ich jetzt abgemacht, heute hatte sie noch die Mens, beim nächsten Mal schaue ich das an und objektiviere das, ist es wirklich, es kann auch mal sein, |**<< Medizinische Beschwerden**|dass die Schamlippen wirklich zu gross sind, dass es wirklich stören kann beim Velofahren und so weiter, die kleinen.|*Untersuchung >>*|*Medizinische Beschwerden >>*| Und dann kann man darüber reden,|**<< Sinnvoll**| ob man sie verkleinert, ja. |**<< Abratung von OP**|Aber wenn ich das anschaue und ich muss sagen, das finde ich jetzt überhaupt nichts, was man medizinisch operieren muss, dann würde ich ihr davon abraten.|*Sinnvoll >>*|

|*Abratung von OP >>*|*spezifisches Beispiel >>*|OD: Mhm. Ähm, fragen Sie manchmal bei Ihren Patientinnen nach, wie sie zur Ästhetik ihres Genitalbereichs stehen?

IP10: |**<< Fragen nicht nach**|Nein.|*Fragen nicht nach >>*|

OD: Ähm, ja jetzt haben Sie schon ein bisschen über Operationen geredet, aber ich frage trotzdem noch mal. Ähm, was sind Ihre Erfahrungen bei Patientinnen, die jetzt direkt nach Schönheitsoperationen am Genitalbereich fragen? Oder die solche Op-

IP10: [|**<< spezifisches Beispiel**|**<< Zusammenarbeit**|Jetzt heute konkret habe ich mir wirklich überlegt, ich müsste mich zuerst erkundigen, wer das überhaupt gut macht. Weil geht das in -, würde ich das gerne ins SPITALNAME schicken, ich schicke sehr gerne ins SPITALNAME, weil dort eben sehr diese Frauenthemen ein grosses Thema ist.|*Zusammenarbeit >>*| |**<< Jüngere**|*spezifisches Beispiel >>*|**<< Abratung von OP**|Weil die ästhetische Chirurgie, also ((seufzt)), da bin ich, ich würde keine junge Frau einfach an einen Schönheitschirurgen schicken. |*Jüngere >>*|*Abratung von OP >>*|Weil eben, es fängt dann unten an, dann kommen die Brüste und dann am Schluss ist die Nase und die Lippe auch operiert.|**<< Kultur**| |**<< Seltene OP**|Das -, aber da habe ich sehr wenige Patienten, jetzt hier in der Schweiz, wirklich ganz anders als in LÄNDERNAME damals.|*Kultur >>*|

|*Seltene OP >>*|OD: Ja und aber wie würden Sie jetzt da vorgehen?

IP10:|**<< Zusammenarbeit**| Konkret würde ich jetzt anrufen ins SPITALNAME und fragen: «Macht ihr das? Macht Ihr Plastik? Verkleinert ihr kleine Schamlippen? Habt Ihr auch so oft Anfragen?»|**<< Alter**|**<< Schwangerschaft/Geburt**|*Zusammenarbeit >>*| Ich weiss, dass es bei den älteren Frauen fast ein grösseres Problem ist nach den Geburten.|*Alter >>*|*Schwangerschaft/Geburt >>*| Weil man da ganz anders aussieht und plötzlich ist alles weit und hässlich und nicht mehr so schön. |**<< Seltene OP**|Aber auch da habe ich ganz wenige Patienten die danach fragen.|*Seltene OP >>*| |**<< Andere Operationen**|Als ich in AUSLÄNDISCHER STADTNAME arbeitete, hat der Chef das gemacht, der Verkleinerungen gemacht. Von der Vagina, aber nicht von der Vulva. |**<< Schwangerschaft/Geburt**|Also im Prinzip ähm, oder, Schlauch-, verengt, nach drei, vier Spontangeburten|*Schwangerschaft/Geburt >>*|, oder.|**<< Sexualität**| |*Andere Operationen >>*|Es ist auch teilweise sexuell gesehen, ist es gar nicht mehr das gleiche wie früher.|*Sexualität >>*| |**<< Zusammenarbeit**|Also konkret würde ich im SPITALNAME anfragen: «Macht Ihr das?», dann würde ich sie dorthin schicken, um sie kurz zu sehen, zur Beurteilung: «Ist das etwas, das Ihr überhaut machen würdet». |**<< Abneigung Chirurgie**|Ich würde keine Schönheitsklinik anschreiben.

|*Zusammenarbeit >>*|*Abneigung Chirurgie >>*|OD: Ja. Mhm, gut. Ähm, ich habe jetzt auch noch ein Fallbeispiel, wenn Sie das kurz durchlesen könnten.

[OD legt Fallvignette 2 hin. IP10 liest Fallvignette 2 durch.]

IP10: Ja. Das ist eben so ein Fall ((lacht)), in AUSLÄNDISCHER STADTNAME jeder zweite, genau.

OD: Was ((lacht)), ähm ja genau, das ist jetzt ein Beispiel einer Körperdysmorphen Störung des Genitalbereichs. Also von der Körperdysmorphen Störung könnte jedes Körperteil betroffen sein und hier geht's um den Genitalbereich. Ähm, ja, was haben Sie für Erfahrungen mit solchen Patientinnen?

IP10:|**<< Seltene KDS**| Wenig. Wirklich wenig, muss ich sagen.|*Seltene KDS >>*| Ich weiss einfach, dass es das gibt und|**<< Unsicher**| |**<< Sinnvoll**|teilweise hilft dann eigentlich ein kleiner Eingriff, damit sie sich einfach selbstsicherer fühlen.|*Unsicher >>*|*Sinnvoll >>*| |**<< Scham**|Weil wenn das ein Leidesdruck ist für diese Person, sie schämt sich in der Umkleidekabine, sie schämt sich mit dem Freund, sie wird immer mehr Komplexe deshalb haben, wieso nicht dann diese Verkleinerung machen, muss ich sagen.|*Scham >>*| |**<< Gespräch**|Da würde ich mit ihr darüber sprechen und jetzt in dem Fall,|*Gespräch >>*| wenn ich -, |**<< Überweisung an Chirurgie**|da würde ich sie ganz klar ins SPITALNAME schicken und da machen die auch nicht zu viel. Also da bin ich mir dann sicher. |*Überweisung an*

*Chirurgie >>*|Ich weiss aber ehrlich konkret jetzt gar nicht, ob sie es überhaupt machen dort oben. |**<< Überweisung an Chirurgie**|Aber ich weiss einfach, dass das immer ein grösseres Thema wird mit der Zeit|*Überweisung an Chirurgie >>*|. |**<< Subklinische Unzufriedenheit**|Ich glaub ich würde der, ich mein ihr Freund sagt, es sei alles, es ist wie gesagt eine Körperdysmorphe Störung, und ich glaube, ich kannte eine andere mit dem Bauchnabel und man hat am Schluss diesen Bauchnabel operiert und es war einfach alles gut. Obwohl ich den Bauchnabel absolut okay fand. Für sie war das aber über Jahre ein Riesenthema. Und es, oder das ist ein kleiner Schnitt, also da hab' ich schon auch gesehen, da schneidet man so wenig weg, dann ist eigentlich für die Patientin alles wieder okay.

|*Subklinische Unzufriedenheit >>*|OD: Gut. Ähm, wie gross würden Sie das Interesse an einer Fortbildung jetzt zur Körperdysmorphen Störung einschätzen. So allgemein im gynäkologischen Fachbereich?

IP10: |**<< Grosses Interesse an Fortbildung**|Ich denke ziemlich gross.|*Grosses Interesse an Fortbildung >>*| |**<< Verstümmelung**|Wir sehen natürlich viele mit Genital Mutilation bei uns oder, weil wir die ganzen Asylanten haben.|*Verstümmelung >>*| |**<< Kultur**|Das ist ja interessant oder, man macht's hier se-, freiwillig und dort wird's gemacht. |*Kultur >>*|Fand's jetzt wunderschön, dass jetzt gestern in 20-Minuten mal so eine Frau auf der Titelseite war, weiss nicht, ob Sie's gesehen haben?

OD: [Nein, hab' ich jetzt nicht ge-

MS: [Mhm, doch, ja, hab's gesehen.

IP10: Das ist jetzt wirklich fortschrittlich. |**<< Grosses Interesse an Fortbildung**|Also ich fände es interessant. Ich weiss nicht, wie ihr das seht, oder die anderen Gynäkologen das öfter sehen,|**<< Seltene KDS**| ich seh's nicht oft, Patienten, die das stört, ich habe es nicht viel.|*Grosses Interesse an Fortbildung >>*|

|*Seltene KDS >>*|OD: Ähm, ja, wie gross würden Sie das Interesse oder den Nutzen von Hilfsmitteln zur Diagnostik der Körperdysmorphen Störung einschätzen? Also jetzt, es gibt zum Beispiel auf Englisch so Screeninginstrumente, die jetzt wahrscheinlich bald übersetzt werden, oder solche Sachen.

IP10:|**<< Grosses Interesse an Diagnostik**| Also in der Jugendgyni gibt's ja auch schon so ein Tool, einfach so ein Akronym, wo man dann fragt «Home, Sex, Drugs» und so weiter. Das find ich ein sehr interessantes Tool, dass man einfach nicht vergisst, irgendeinen Aspekt zu erfragen. |**<< praktisch**|Und wenn es so ein Tool gäbe, wo man jetzt ziemlich kurz innerhalb von zwei, drei Fragen so etwas findet, finde ich das sehr hilfreich. |*praktisch >>*|Wie mit der Depression oder gibt es ja auch ein kurzes Tool, zwei Fragen. Und dann kann man eigentlich schon einschätzen, ist es jetzt schwer oder nicht.|*Grosses Interesse an Diagnostik >>*|

OD: Und wie gross würden Sie das Interesse an Hilfsmittel für die Beratung jetzt von Frauen die solche Symptome zeigen, also die mit der Ästhetik ihres Genitalbereichs unzufrieden sind, einschätzen? Also so, es gibt vielleicht so Bilder oder einfach solche Sachen.

IP10: Mhm. Wie meinen Sie jetzt Bilder? Was ist normal, oder? Das ist ja dann halt schwierig.

OD: [Ja, genau. Also

MS: [Es gibt doch so Plakate wo verschiedene, äh ja, Genitalien, genau, abgebildet sind. Oder die vom SPITALNAME hat sogar Gipsabdrücke, die sie zeigen können und solche Sachen.

IP10: [Wirklich? (3) |**<< Mittel Interesse an Hilfsmittel**|Ja, wenn ich mehr Patienten hätte, könnte ich vielleicht darü-, |**<< Visuell**|**<< Normalität betonen**|ich denke jetzt aktuell für mich, ich google dann immer. Wirklich, man sieht da alles und dann kommen irgendwie zehn Bilder und dann zeige ich den Patienten: «Schauen Sie, es kann von so bis so aussehen, oder», das ist einfach alles in der Norm.|*Visuell >>*|*Normalität betonen >>*| Ja, genau. (3) Ja, aber so gute Bilder, ästhetisch schöne, ist natürlich schwierig, oder. Fänd' ich auch hilfreich, aber jetzt nicht top.|*Mittel Interesse an Hilfsmittel >>*|

OD: Mhm, gut, dann wären wir schon am Ende, ausser Sie möchten noch etwas hinzufügen?

IP10: Nein, ist gut ((lacht)).

MS: Danke vielmals.

Interview mit: IP11
Datum des Interviews: 28.06.2019
Dauer: 31 min
Anonymisierte Begriffe: Autorinnenname, Stadtname, Ausländischer Stadtname, Preise, Ländernamen
IP-Nummer: 11

MS: Gut dann beginn ich mit der ersten Frage, ich habe die hier. Mit welchen psychischen Problemen kommen denn die Patientinnen zu Ihnen in die Praxis?

IP11: Also die Patientinnen kommen primär nicht mit psychischen Problemen. Aber viele Gründe, warum sie kommen haben psychische, psychische Hintergründe. |**<< Vertrauensperson**|Also ich würde meinen als Gynäkologe ist man ein Vertrauensarzt.|*Vertrauensperson >>*| So sehe ich meine Aufgabe, und äh, ich hab ein Ohr offen für alles was die ganzheitliche Erfassung ist. Also es kann jemand mit Symptomen kommen, Bauchweh, und am Schluss ist es eine |**<< Somatoforme Störungen (F45)**|psychosomatische Geschichte,|*Somatoforme Störungen (F45) >>*| wenn ich nichts anderes finde. Ich geh immer auch auf Beziehungen ein ich frage wo sie stehen. Wenn ich eine Frau kennen lerne will ich immer wissen was ist sie von Beruf wie steht sie mit, wie ist sie in den Beziehungen, wird immer thematisiert. Für mich gehört das zu diesem Bild. Aus einer Krankengeschichte sieht man immer, ich habe so Zeichen wo ich seh', ist es das erste, zweite Mal, dritter Freund oder was auch immer, es interessiert mich. Ja. Und mit welchen Problemen kommen sie? Psychischen Problemen. Zum Teil |**<< Depressive Episode (F32) und rezidivierende depressive Störung (F33)**|Depressionen|*Depressive Episode (F32) und rezidivierende depressive Störung (F33) >>*|, es gibt natürlich verschiedenen Gründe. Ein Gynäkologe ist im Prinzip, guter Gynäkologe oder Gynäkologin sollte eigentlich psychologisch geschult sein, oder? Die meisten sind es auch, ich mein, also innerlich, hoffe ich.

MS: Welche Rolle spielt den die Zufriedenheit mit dem äusseren Erscheinungsbild bei Ihren Patientinnen?

IP11: Zufriedenheit mit dem Äusseren?

MS: Mhm, mit dem Aussehen.

IP11: Da erleben Sie alles Mögliche. Sie, es kommt darauf an was man anschauen will. Wenn das Thema Schema, wie fühl ich mich und so weiter. Es gibt ja Frauen die wollen sich zum Beispiel die Brust vergrössern lassen. Obwohl sie wunderschöne Brüste haben. Ähm, und bei denen ist es extrem schwierig davon ab zu raten. Ich probiere immer wieder da zu bremsen oder zu sagen wozu brauchst du das, sogar wenn der Freund das nicht nötig, wenn sie aber die Kollegin und ich will das, was auch immer. Und dann hat man keine Chance. Und man realisiert dann die hat dann andere Probleme, die lenkt dann ab, die möchte nicht darauf eingehen. Ähm, dann sage ich einfach ganz häufig, Sie müssen sich das ganz gut überlegen, weil das ist, ist nicht von mir aus, ausser sie haben Probleme. Ich meine da gibt's junge Mädchen mit Asymmetrien oder dann schau- kämpf ich auch dafür, dass das bezahlt wird und so weiter. Dass die Krankenkassen das zahlen. Also klar wenn's ein Grund gibt selbst verständlich. Und heute, ähm, wo man sich Unten rasiert ist das auch eine ganze Geschichte, oder? Das wird auch ausgenutzt, die wissen gar nicht wie eine normale Frau aus sieht die haben irgendwelche Bilder. |**<< Labien erwähnt**|Und meinen sie müssen die Vulva, was da korrigieren und so weiter.|*Labien erwähnt >>*| Dann habe ich Bilder wo ich zeigen kann was normal ist und was nicht. Und sage was Sie da haben ist ganz normal, ausser Asymmetrien oder was auch immer. Ähm, ja. Ich mache sogar manchmal Eingriffe, wenn es sie psychisch stört massiv dann mache ich es, so. Aber ich verlange nicht diese horrenden Preise die dann auf dem Markt verlangt werden.

MS: Ich habe jetzt eine Fallvignette für Sie. Und würde Sie bitten diese kurz durch zu lesen.

[MS gibt IP11 Fallvignette 1.]

IP11: Also eine Patientin, Fall.

MS: Genau.

IP11: [Liest Fallvignette 1 durch.] Genau, was ich vorher erzählt habe (5). Ok, Ja.

MS: Ähm meine Frage dazu ist jetzt, welche psychische Störung, Störungen kommen Ihnen denn zu diesem Fallbeispiel in den Sinn?

IP11: Ja diese, das Mädchen hat ganz |**<< vermindertes Selbstwertgefühl und Selbstvertrauen**|grosse Selbstzweifel.|*vermindertes Selbstwertgefühl und Selbstvertrauen >>*| Und die sind, aus meiner Erfahrung bedingt, kommt aus der Kindheit. |**<< Körperwahrnehmungsstörung**|Dass sie nicht richtig gespiegelt wurden in ihrem Körper abgeholt wurden, die stecken nicht in ihrem eigenen Körper, das ist eine psychische Störung, sie will sich irgendwie von aussen anders sehen als sie ist und die Frage ist auch ob sie sich überhaupt richtig wahrnimmt.|*Körperwahrnehmungsstörung >>*| Also das wäre ja der Idealfall. Mit der würde ich jetzt eine Standortbestimmung machen und dann mit diesen anderen Themen als nur gerade mit ihrem Körper. Wie sie sich überhaupt sieht und fühlt und ja.

MS: Ähm, worauf basieren Sie Ihre Annahmen, also welchen Textstellen?

IP11: (2). Also sie hat negative, also ihre krumme Nase, und ich weiss das meine Mutter unter einer krummen Nase gelitten hat und dass man sie hoch genommen hat in der Pubertät, in der Pubertät kommt darauf an was da gelaufen ist. Wurde sie vielleicht gehänselt etc. Und hat deswegen eine falsche Wahrnehmung und, und das vielleicht. Und die hängenden Brüste okay, das ist natürlich äh, relativ. Eine 25-jährige hat sicher keine hängenden Brüste. Aber das nützt, wenn sie das Gefühl hat die hängen dann muss man vielleicht genau darauf eingehen was sie damit meint. Ja. Also einerseits muss man sie schon abholen, um zu sehen wo es jetzt drückt. Aber, ja. Und die hässlichen Haare, da kann man helfen. Also es gibt Sachen da kann man etwas machen. Selbstverständlich, auf das eingehen, ja.

MS: Wenn jetzt diese Patientin zu Ihnen, kommt, welche Informationen würden Sie sich noch von ihr einholen?

IP11: Von der Patientin selber?

MS: Genau, Genau, wenn sie jetzt einfach -

IP11: Also ich frage mich, wie sie, wie sie, warum sie das so wahrnimmt. Und, äh, ich würde es zusammen anschauen mal primär und sagen ja gut. Sag ich, ich sehe natürlich auch andere Brüste und ich kann sagen, dass das vielleicht nicht so schlimm ist wie sie das Gefühl hat. Ja. Aber es ist, so empfindet sie das, das kann man nicht einfach in Frage stellen. Dann würde ich mit ihr sagen, wie gesagt, ich würde sagen ja vielleicht müssen wir mal darüber reden. Wie, wie, wie sie überhaupt dazu kommt, dass sie sich so sieht wie sie angeblich ist. Weil, offensichtlich in diesem Fall stimmt das nicht ganz überein. Sie hat ein negatives Bild von sich selber, es geht darum heraus zu finden woher, dass das kommt. Das findet man manchmal schon raus.

MS: Ich habe jetzt eine kleine Auflösung zu diesem Fallbeispiel. |**<< KDS unbekannt**|Ähm

und zwar ist das ein, ist die Fallvignette ein Fallbeispiel für die Körperdysmorphe Störung. Haben Sie davon schon mal gehört?

IP11: (2) Nein.

|*KDS unbekannt >>*|MS: Nein.

IP11: Ja.

MS: Ähm, genau. Das sind kurz die Kriterien, [Gibt DSM-5-Kriterien an IP11]. Eben es geht um eine übermässige Beschäftigung, wie im Fallbeispiel, und zwar von einem oder mehreren wahrgenommenen Mängeln oder Defekten, die aber eben für Andere nicht erkennbar oder nur geringfügig sind, das ist ganz wichtig. Ähm, die Leute leiden unter wiederholenden Verhaltensweisen, sie schauen sich stundenlang im Spiegel an, sie wollen ihre Makel abdecken, immer wieder nachfragen, sie vergleichen sich mit Anderen und es ist eben mit einem sehr hohen Leiden und Beeinträchtigungen verbunden.

IP11: Ja, es geht in die Zwangsstörung, also wenn's extrem ist, ja.

MS: Genau, und einfach als Abgrenzungskriterium, es ist keine Essstörung. Genau.

IP11: Ja es hat wahrscheinlich gewisse Parallelen. Ich habe jetzt gerade so eine, die macht Spitzensport und Gymnasium und hat einen BMI von was weiss ich. Wahrscheinlich auch Wahrnehmungsstörung.

MS: Wenn Sie sich jetzt diese Kriterien anschauen und auch das Fallbeispiel, ähm haben Sie denn Erfahrungen mit Patientinnen die mit so einer Symptomatik zu Ihnen kommen?

IP11: Ja das haben wir. Also ich nenn jetzt das nicht so, aber jaja, klar. Aber das geht ja darum, dass man, dass man, ja muss man versuchen rauszufinden, wo das Problem liegt oder. Und gewisse sind gar nicht für das zugänglich, die bringen eine andere Lösung, die wollen einfach, dass etwas gehandelt wird. Das wird ausgenutzt. ( ) in dem Bereich. Da gibt's Kollegen die verdienen sich dumm und dämlich.

MS: Also Sie meinen Operationen?

IP11: Ja. Es hat aber, es gibt äh, äh, ja plastische Chirurgen, die das einbeziehen. Aber das sind wenige, die psychologische Abklärungen machen und so weiter. Wenn es da was zu verdienen gibt.

MS: Ja, das wäre meine nächste Frage gewesen, nämlich was denn die Herausforderungen sind im Kontakt mit diesen Patientinnen für Sie?

IP11: (5) Ja das ist das Problem, diese Zwangsstörungen sie überhaupt, ähm, überhaupt an sie zu gelangen. Die haben ein Kommunikationsproblem, da kommt man nicht dazu. Das ist eine Ohnmacht, also ein Ohnmachtsgefühl, das sie in einem hinterlassen. Die verstehen, oder die will es nicht verstehen oder ich komm nicht ran. Das gibt's dann schon manchmal, das ist nicht einfach. Oder sie springen ab. Weil man nicht gerade so denkt, wie sie denken. Die wollen nur ihre Bestätigung ihrer Störung. Das ist, sie sollten, die Kunst die trotzdem zu behalten, ja. Und an die, an die Grundproblematik zu kommen. ( ) sagen sie ja meistens nicht, primär.

MS: Wie ist denn Ihr professionelles Vorgehen mit diesen Herausforderungen um zu gehen?

IP11: Ja ich, ich nehm' sie mal primär ernst. Oder? Und versuch sie auf der somatischen Schiene da, ja mal abzuholen und verstehen, dass sie das vielleicht sehen. Und das

zwanghafte, das kann man nicht denen einfach so sagen, das ist zwanghaft, das bringt ja nichts. Die Frage ist dann, äh was kompensiert sie da. Was will sie. Und das sind häufig Beziehungsstörungen, die im Hintergrund sind. Eben ich mache, wenn sie einverstanden sind mache ich eine körperpsychotherapeutische Standort Bestimmung. Da mach ich Entwicklungspsychologie meistens und arbeite mit dem Körper und andere Sachen. Sich sonst berühren und am Boden wahrnehmen und so weiter, und so. Aber ich würde meinen, achtzig Prozent wollen es gar nicht.

MS: Wollen es gar nicht. Und die gehen dann weiter?

IP11: Jaja, die bleiben auf dem, oder lassen sich operieren, das gibt es auch. Das kann ich auch nicht ändern.

MS: Genau, meine letzte Frage, das haben Sie alles schon ein bisschen angetönt, ist: Wie wäre denn jetzt Ihr professionelles Vorgehen, wenn jetzt eine Patientin zu Ihnen kommt bei der Sie Verdacht, nachdem Sie jetzt die Kriterien gesehen haben, bei der Sie Verdacht auf eine Körperdysmorphe Störung haben, wie würden Sie da vorgehen?

IP11: Wenn ich jemanden wüsste. Der sich auf das spezialisieren würde, oder? Würde ich meinen, das ist, ich habe leider nicht so wahnsinns gute Erfahrungen mit Psychiatern und Psychologen, bin sehr häufig enttäuscht. Ich habe selber mehrere Leute die schon jahrelang in Therapie sind und dann kommen sie hierher und es gab schon quasi, könnt Ihnen Geschichten erzählen. Ähm ja, ich weiss es nicht, also es geht ja darum, dass sie, der Leidensdruck muss genug hoch sein, dass sie das überhaupt einsieht, dass sie eine Therapie braucht. Das ist das Problem, oder? Und wie man das hinkriegt, das ist nicht einfach. Also mich dünkt das ist der Schlüssel, ja. Und primär zum Psychologen schicken werden die sagen: «Geht's noch, bin ja völlig, wo ist das Problem», oder? Die wollen das ja nicht wahr haben oder. Ähm, eben probieren eine Brücke zu schlagen wenn's geht, dass sie dann irgendwo, ja. Oder noch Fremdanamnese einholen, gibt's natürlich auch noch. Wenn die Mutter da ist, dass man vielleicht so jemand mit einbezieht. Das gibt einem ein Bild, was da im Hintergrund auch noch mitläuft. Ja. Weiss nicht ob es Leute, Kollegen gibt die sich da auf das, spezialisiert sind?

MS: Dazu kommen wir noch.

OD: Ja.

IP11: Gut.

MS: Gut, danke vielmal.

OD: Gut, dann mach ich mal weiter. Ähm, was sind Ihre Erfahrungen mit Patientinnen, die Fragen zur Ästhetik oder zur Normalität ihres eigenen Genitalbereichs haben?

IP11: Ah, jetzt kommen wir zu dem Thema.

OD: ((lacht)).

IP11: Also was sind die Fragen, oder-?

OD: Was sind Ihre Erfahrungen mit -?

IP11: Meine Erfahrungen?

OD: Ja genau, mit Patientinnen die Fragen dazu haben.

IP11: Was ich schon vorher gesagt habe. |**<< Individualität**|Das ist sehr unterschiedlich, ich mein sie ähm. |*Individualität >>*|**<< Vergleichen**|Es gibt Leute die kommen, weil sie (3) sagen wir in der Sauna Andere gesehen haben, das Gefühl haben. Oder sie vergleichen sich, das ist ja häufig dann, wenn das kommt.|*Vergleichen >>*| |**<< Veränderungen**|**<< Jüngere**|Gerade so, ja, nach der Pubertät kommt das häufig so, so Fragestellungen.|*Veränderungen >>*|*Jüngere >>*| |**<< öffnen sich eher Frauen gegenüber**|Teilweise macht das auch meine Frau und nicht ich. Ich gebe das meistens meiner Frau weiter. [IP11 führt mit Ehepartnerin eine gynäkologische Praxis.] Also ich glaube meine Frau bespricht so, in dem Alter mehr.|*öffnen sich eher Frauen gegenüber >>*| |**<< Labien**|Und sonst, wie gesagt, mit diesen Labien, oder. |**<< Visuell**|Besonders wenn man's mit Spiegel anschaut, genau anschaut nochmals uns sagt, das sind die grossen Labien, wenn sie natürlich, sie sind jetzt relativ schlank und dann kommen diese kleinen Labien mehr hervor|*Visuell >>*| und.|*Labien >>*| |**<< Aufklärung OP**|**<< Erwähnen OP**|Aber dann mach ich auch, dann erklär ich ihnen das, wenn man das operiert dann sieht es vielleicht schöner aus, aber es gibt Narbenbildungen, das kann man nicht immer verhindern.|*Aufklärung OP >>*|*Erwähnen OP >>*| |**<< Unrealistisches Ideal**|Und es ist weniger geschützt und dann muss, ästhetische Vorstellungen die gegeben werden, die man so quasi als normal sieht, ist gar nicht normal.|*Unrealistisches Ideal >>*| |**<< Expertise nutzen**|Muss man sie einladen, dass sie mal alle Frauen -, ein Tag lang bei mir zu sehen und unten rein schauen, oder?|*Expertise nutzen >>*| |**<< Uninformiert**|Die AUTORINNENNAMEN hat mal so ein Buch geschrieben, da mit dem, ich habe mich mit der Sexualkunde am Anfang ziemlich engagiert gehabt, die ist in STADTNAME und hat so ein Buch rausgegeben wo sie mit Bleistiftzeichnungen im Prinzip verschiedenen Sexual- der Frauen, zeigen was normal ist quasi. Die haben falsche Vorstellungen. Ja.|*Uninformiert >>*|

OD: Genau, das haben Sie jetzt schon ein bisschen beantwortet, aber wie ist dann Ihr Vorgehen, wenn solche Fragen kommen, dann machen Sie einfach Aufklärung, oder?

IP11: |**<< Gespräch**|Ja wir schauen es an und ich frage genau was sie meint, was jetzt da nicht gut ist und so.|*Gespräch >>*| |**<< Medizinische Beschwerden**|Weil das kann ja wirklich und das kann ja sein, dass es, wo sie sagen beim Fahrradfahren stört es und so. Sage ich: «Ja gut, das übernehmen sie dann von Anderen, dass sie sagen beim Fahrradfahren stört es, aber wissen Sie ich habe einen Hoden, das stört ja viel mehr eigentlich» ((lacht)).|*Medizinische Beschwerden >>*| |**<< Strategien**|Ja man muss ein bisschen relativieren und sagen: «Ich weiss jetzt nicht ob das wirklich, müssen Sie ein bisschen büscheln, oder andere Unterhosen.»|*Strategien >>*| |**<< Unsicher**|Da ist die Frage, darf ich so sein wie ich bin, oder? Das sind grundlegende Fragen und das ist auch die Toleranz mit der ich aufgewachsen bin oder nicht. Das hat sehr viel mit dem zu tun.|*Unsicher >>*|

OD: Fragen Sie manchmal bei Ihren Patientinnen nach, wie Sie zur Ästhetik ihres Genitalbereichs stehen?

IP11: «Gefällt Ihnen Ihr Genital?»?

OD: Ja.

IP11:|**<< Fragen nicht nach**| Nein, das habe ich jetzt noch nie gefragt.|*Fragen nicht nach >>*|

OD: Gut.

IP11: |**<< Viele Fragen** |Nein, wenn ich's Gefühl-, also verstehen Sie, ich habe sehr viele, ich mein ich kann Ihnen erzählen.|*Viele Fragen >>*| |**<< spezifisches Beispiel**|**<< Vertrauensperson**|Ich habe eine Patientin, die ist schwanger geworden, war bei einer Gynäkologin und hat gesagt sie hat ein bisschen Angst vor der Geburt und dann ist sie auf das gar nicht eingegangen. Dann hat sie gewechselt, weiss nicht warum sie zu mir

gekommen ist. Und beim ersten Gespräch habe ich schon gemerkt, okay, vielleicht ist da was, oder? Beim Untersuchen und da habe ich gefragt, vielleicht ist da mal etwas vorgefallen und so. Und das kam jetzt raus, die wurde missbraucht von fünf bis zwölf vom Vater. Und bei der Scheidung, ging sie zum Vater, weil der der einzige war der wenigstens mit ihr lieb umging. Die Mutter war so kalt. Also, grässliche Geschichten, oder? Und dann haben wir das mit dem Ehemann der das vorher nur gehört hat, könnte Ihnen die Krankengeschichte geben, ich habe da dreissig Seiten geschrieben.|*spezifisches Beispiel >>*|

|*Vertrauensperson >>*|OD: Wow.

IP11: |**<< Psychische Probleme im Hintergrund**|Und dann geht's darum. Wenn das dann der Grund ist. Das braucht schon viel voité oder das man merkt, dass da vielleicht etwas dahintersteckt oder.|*Psychische Probleme im Hintergrund >>*| Also ich meine es ist sehr wichtig, also es kann einfach ein Zeichen sein, wenn man das Gefühl hat etwas stimmt nicht, bei mir.

OD: Ja, genau. (2). Was sind denn Ihre Erfahrungen mit Patientinnen die jetzt direkt nach Schönheitsoperationen des Genitalbereichs fragen?

IP11:|**<< Seltene OP**| Komischerweise habe ich nicht viele.|*Seltene OP >>*|**<< Sinnvoll**| Ja also wenn sie Fragen würden, dann sage ich, ist es von mir aus berechtigt oder nicht.|*Sinnvoll >>*| |**<< Selbst behandeln**|Und wenn es wirklich berechtigt ist würde ich, dann mach ich's|**<< Gelder**| aber nicht für diese tausend Franken sondern, ich mache es einfach.|*Selbst behandeln >>*| Operiere das für die PREIS Franken die ich kriege.|*Gelder >>*| |**<< Simpel**|Das ist keine Kunst, ich mein das ist verrückt was da. Das ist ein Riesenbusiness, Riesenbusiness.|*Simpel >>*|

OD: Wann wäre es denn berechtigt?

IP11: |**<< Medizinische Beschwerden**|**<< Sinnvoll**|Eben, Asymmetrien, gibt's zum Beispiel, oder? Eh, es gibt schon Gründe. Oder nach irgendwelchen Eingriffen, nach Geburt. Sachen die stören, gibt's auch. Die ganzen ( ) gibt's auch, die bringen ein Problem. Tampon nicht rein, oder kleine Sachen die man operieren muss das ist-. Aber sind keine kosmetischen Eingriffe in dem Sinne.

|*Medizinische Beschwerden >>*|*Sinnvoll >>*|OD: Gut. Also wie ist dann, wieder wie ist dann Ihr Vorgehen, wenn eine Patientin eine, sozusagen kommt und sagt ja sie würde gerne eine Schönheitsoperation haben?

IP11: |**<< Abratung von OP**|Ja dann versuche ich ihr das so auszureden. |*Abratung von OP >>*|Und wenn sie -. Und ich sage selber: «Ich würde das bei Ihnen jetzt nicht operieren, es tut mir leid, ich würd's nicht machen.» |**<< Entscheidung überlassen**|Das ist ihre Wahl, wenn sie gehen will, ist okay.|*Entscheidung überlassen >>*| |**<< Komplikationen** |Sage ich: «wenn sie Komplikationen haben, können Sie dann zu mir kommen». Gibt's ja auch.|*Komplikationen >>*|

OD: Ja. Gut. Dann habe ich jetzt auch noch mal ein Fallbeispiel, dass Sie lesen können.

IP11: Jawohl.

[OD gibt IP11 Fallvignette 2.]

IP11: [Liest Fallvignette 2 durch.] Jawohl.

OD: Ja also, das ist jetzt ein Beispiel einer Körperdysmorphen Störung des Genitalbereichs. Also ist jetzt nicht so eine spezifische Diagnose aber es kann einfach auch dort vorkommen.

IP11: |**<< Psychische Probleme im Hintergrund**|Ja das ist einfach ein Ausdruck von dem was sie erleben. |*Psychische Probleme im Hintergrund >>*|Was diese Körperdysmoprhe Störung.

OD: Genau. Was sind Ihre Erfahrungen mit Patientinnen die solche Symptome zeigen?

IP11: Ich glaube ich habe schon, schon beantwortet. Also im Prinzip ist es. |**<< Ernstnehmen**|Geht darum ein zu gehen, ja tatsächlich die [Schamlippen] sind ein bisschen grösser, stimmt oder. Also nicht verneinen, nicht verniedlichen.|*Ernstnehmen >>*|**<< Aufklärung**| Und sagen ja aber es hat eine Funktion. Dann erkläre ich da, dass das ein Schutz ist.|*Aufklärung >>*| |**<< Intimrasur**|Und früher hat man ein Busch gehabt, heute rasieren sie sich. Das hat man gar nicht gesehen, das war unter den Haaren, oder?|*Intimrasur >>*| |**<< Zunehmend**|Deshalb boomt das so, weil man das anschaut, oder. |*Zunehmend >>*|**<< Medien**|Und dann werden sehr viel, die ganze Porno Geschichten und so,|*Medien >>*| |**<< Unrealistisches Ideal**|sehen sie das, da werden die Frauen ausgesucht, die natürlich nicht, die halt so eher fast androgyne eh Genitalien, eh nicht so, |**<< Vielfalt**|gibt's ganz verschiedene Ausdruck.|*Unrealistisches Ideal >>*| |*Vielfalt >>*|**<< Fehlende Einsicht**|Das kann man denen in dem Sinn nicht ausreden, dass sie das nicht schön findet, wenn sie etwas anderes schön findet, das ist schwierig. Weiss nicht wie das professionell umstimmen wollt.|*Fehlende Einsicht >>*| (3). Ja es geht darum, dass, |**<< Psychische Probleme im Hintergrund**|meistens ist es ja ein Symptom. Ich würde sagen die haben auch sonst ein Problem mit sich selber, grundlegende Geschichte, oder|*Psychische Probleme im Hintergrund >>*|?

OD: Also würden Sie da auch einfach, |**<< Gespräch**|erklären –

IP11: Jaja, auf alle Fälle und sagen, die sind jetzt für mich. «Das Sie das stört, das kann ich vielleicht verstehen, dass – dann nimm doch einfach 20 Kilo zu dann ist die grössere Schamlippe grösser dann ist es, aber Du willst nicht so dünn, so dick sein, oder?» |**<< Aufklärung**|Muss ich sagen, der Körper hat für gewisse Sachen vorgesorgt, die kleinen Schamlippen sind da um die Öffnung zu zuhalten. |*Aufklärung >>*|**<< Schwangerschaft/Geburt**|Ich könnte Sie einladen und sagen es gibt Frauen die nach der Geburt, dass eben nicht zu sind und die haben viel mehr Probleme. |*Schwangerschaft/Geburt >>*|Die Scham- die das nicht richtig zu macht und probiert. |**<< Fehlende Einsicht**|Wenn die Zwanghaft sind dann hat man keine Chance. Ja.|*Gespräch >>*|

|*Fehlende Einsicht >>*|OD: Gut. Ähm. Wie gross würden Sie schätzen wäre das Interesse an einer Fortbildung zur Körperdysmorphen Störung, allgemein in Fachbereich der Gynäkologie?

IP11: |**<< Grosses Interesse an Fortbildung**|Das ist super, das müsste man so einfach als Seminar machen. Austausch unter Kollegen.|*Grosses Interesse an Fortbildung >>*| «Wie machst es Du?». So etwas. |**<< Abneigung Chirurgie**|So könnte man das angehen und dann gleich noch diese Ästheten noch einladen, die davon profitieren, weil mich, ich hab', mir kommt immer Wut dann rauf, die nutzen das aus.|*Abneigung Chirurgie >>*| Ich ging mal, ich wollte wissen wie das ist, ich ging dann nach AUSLÄNDISCHER STADTNAME an eine Fortbildung. Habe mich da eingeschrieben, dachte ich super, äh, habe den Flug schon gebucht, das war eine dreitägige Ausbildung in kosmetischer Operationen und ich wollte wissen, was machen die dort. Und als ich mich einschreiben wollte hätte das PREIS2 Franken gekostet. Und dann habe ich gesagt: «Nein. Habe aber den Flug schon gebucht». Dachte ich, gut fahre nach AUSLÄNDISCHER STADTNAME und geh einfach nicht in den Kurs aber geh einfach schauen, wer geht dort hin. Habe ich mich dort angemeldet und bin ein bisschen später gekommen als die Anderen und habe an der Reception geklopft und das war so eine hollywood-mässige Klinik, selbstverständlich nett. Und dann habe ich gesagt ist der Ding da, und dann kam dieser Chef da, selber und sprach mit mir. Ich habe dann mit ihm

sehr offen geredet und gesagt: «Ich bin aus der Schweiz. Was Ihr da anbietet und ich habe meine grössten Zweifel da, was Ihr da macht». Oder?. «Aber ich würde gerne sehen wer da mitmacht.» Da hat er mich eingeladen mitmachen. Und dann bin ich mit. Und da waren Frauen, eingekleidete von, und ja islamische Ärztinnen und ja LÄNDERNAME. Und dann haben sie gesagt, sie, also der war schön seriös. Er musste viele Eingriffe, die dann schief gingen korrigieren. (3) Aber eben PREIS3 Franken, weil man eben verdient, oder? Und dann wurde gleichzeitig geschult, wie mache ich mein Auftritt. Homepage, Facebook, alles, Instagram, wie bringe ich die Leute hin. Marketing. Also der halbe Tag war Marketing.

OD: Wow. (.) Wie gross würden Sie das Interesse oder den Nutzen an Hilfsmittel zur Diagnostik einer körperdysmorphen Störung einschätzen? Also so ein Screeninginstrument zum Beispiel?

IP11: (2). |**<< Mittel Interesse and Diagnostik**|Müsste man sehen, müsste man ausprobieren. Weil wir machen das im Prinzip unbewusst.|*Mittel Interesse and Diagnostik >>*| Also wir nennen es nicht Körperdysmorphe Störung aber, ja. Wir haben nicht diesen Stempel, ja. |**<< Fehlende Einsicht**|Es ist schon frustrierend, das zwanghafte, es ist schwierig mit denen. Es ist eine Zwangsstörung, die hast du nicht gleich umgebrochen. Zu was zählt ihr das? Zwangsstörung, was ist das im Prinzip, ist es? Weil das ist ja nicht eine (2)?

|*Fehlende Einsicht >>*|OD: Schon eine psychische Störung.

IP11: Ja, aber keine Persönlichkeitsstörung aber es sind so, schon gewisse –

OD: Ist eine eigene Kategorie, sozusagen.

IP11: |**<< Medikamente verschreiben**|Jaja. Man könnte da nicht einfach Medikamente abgeben.

OD: Ja es gibt schon Medikamente die helfen aber meistens bringen Medikamente nur etwas wenn man's in Kombination mit einer Psychotherapie nimmt.

|*Medikamente verschreiben >>*|IP11: Ja.

OD: Noch die letzte Frage: Ich glaube Sie haben erwähnt, dass Sie das eigentlich schon brauchen, aber jetzt allgemein wie gross würden Sie das Interesse an Hilfsmittel für die Beratung von Patientinnen mit Körperdysmorphen Störung, spezifisch des Genitalbereichs einschätzen?

IP11: |**<< Überweisung Psychotherapie**|Also ich würde - . Das Beste wäre in Gruppen, die in Gruppen nehmen. Ich würde Gruppentherapie machen.|*Überweisung Psychotherapie >>*| Das wäre wahrscheinlich das Beste weil das nimmt, geht dann weg von mir selber und ich habe Kolleginnen die das gleiche haben und ich kann mich austauschen und man merkt dann «Ah, erstens bin ich nicht alleine». Wahrscheinlich hätte das, von mir aus gesehen, den besten Impact. Ja. Das wäre eine Idee, dass man so was machen würde. |**<< Gelder**|Und der Krankenkasse, dass jede oder überhaupt müsste man jede |**<< Abneigung Chirurgie**|ästhetische Operation so, zu so was hingehen, was da alles passiert, können sie sich nicht vorstellen.|*Gelder >>*|*Abneigung Chirurgie >>*| Aber es hat natürlich auch mit dem ethi- . |**<< Kultur**|Es sind auch kulturelle Unterschiede. Es sind in LÄNDERNAME2, da lässt sich jede zweite da die Brust aufspritzen oder was auch immer, oder? Und ähm die macht das auch, da kann man nicht sagen es ist eine Störung sondern kulturelles Verfahren. Mache ich ja, Mode, wie ich das T-Shirt anziehe. Mache ich mir die Brüste, ist völlig-, nein es ist so.|*Kultur >>*| |**<< Subklinische Unzufriedenheit**|Also das kann man nicht nur als Störung sehen.|*Subklinische Unzufriedenheit >>*|

OD: Gut dann wären wir eigentlich schon fertig, ausser Sie möchten noch etwas hinzufügen.

IP11: Ja ich habe Freude gehabt, sind Sie vorbeigekommen.

OD: Danke ((lacht)).
